# Proceedings of the 2017 Advancing the Science of Community Engaged Research (CEnR) Conference

**DOI:** 10.1186/s12919-019-0164-y

**Published:** 2019-04-19

**Authors:** Al Richmond, Sergio Aguilar-Gaxiola, Eliséo J. Perez-Stable, Usha Menon, Chanita Hughes-Halbert, Karriem S. Watson, Regina Greer-Smith, Courtney Clyatt, Jonathan N. Tobin, Consuelo H. Wilkins

**Affiliations:** 1 Community Campus Partnerships for Health, Raleigh, NC 27605 USA; 2 Clinical Internal Medicine, School of Medicine, University of California, Davis, Sacramento, CA 95817 USA; 3 National Institute on Minority Health and Health Disparities, Bethesda, MD 20892-5465 USA; 4 University of South Florida, Tampa, FL 33612 USA; 5 Medical University of South Carolina, Charleston, SC 29425 USA; 6 University of Illinois at Chicago Cancer Center, Chicago, IL 60612 USA; 7 President of Healthcare Research Associates, LLS, Chicago, IL USA; 8 Patient-Centered Outcomes Research Institute, Washington, DC 20036 USA; 9 Clinical Directors Network, Inc., New York, NY 10018 USA; 10 Vice President for Health Equity, Vanderbilt University Medical Center, Executive Director, Meharry-Vanderbilt Alliance, 1005 Dr. D.B. Todd Jr. Boulevard, Nashville, TN 37208 USA

**Keywords:** Research and Innovative Approaches, Community Engaged Research, Partnerships, Diversity, Health Equity

## Abstract

**Background:**

To address an urgent need to advance the field of community engaged research, faculty at Vanderbilt University Medical Center and Meharry Medical College organized the national meeting "Advancing the Science of Community Engaged Research (CEnR): Innovative & Effective Methods of Stakeholder Engagement in Translational Research, Washington, DC September 14-15, 2017 (See Additional file 1). These meetings brought together a diverse group of stakeholders to share community engaged research evidence and practical knowledge for implementing new and enhancing existing research programs. The conference series’ goals were: 1) to expand the scientific basis for the community engaged research field by convening researchers, community partners, patient advocacy organizations, and others to share innovative methods and strategies; 2) to engage community representatives and patient advocates in the development of new approaches in community engaged research by meaningfully involving them in the planning, as speakers and presenters, and as conference participants; and 3) to catalyze innovative community engaged research using interactive meeting methods that promote learning, support collective problem solving, and encourage new conceptual frameworks. These conferences have advanced community engagement across the translational research spectrum in biomedical research. For the 2017 meeting, described here, the overarching theme was *Innovative and Effective Methods of Stakeholder Engagement in Translational Research.*

**Methods:**

The forum was attended by over 210 participants. This conference used novel approaches to fulfill its objectives of participant diversity, meaningful stakeholder engagement, and eliciting varied distinct perspectives to advance the science of community engaged research. Innovative strategies for the conference included: Think Tanks focused on emerging community engaged research topics or topics in need of urgent attention. These dynamic group sessions provided for freely sharing ideas with the purpose of creating change and facilitating new research collaborations. Learning Labs offered unique opportunities to gain practical knowledge regarding innovative methods in community engaged research. Learning Labs also facilitated the wide broadcast of locally successful engagement methods with the goal of speeding the uptake and implementation of community engaged methods. Travel Scholarships were provided for twenty community and patient representatives to participate in the conference. The lack of travel funds was a significant barrier to stakeholder participation in prior community engaged research meetings. The scholarships expanded the role of community and patient representatives in setting research priorities and promoting methods development. Meaningful Engagement meant that community members and patients participated in decision making on all aspects of the conference planning, including the selection of themes, topics, and speakers, and were fully integrated into the conference as speakers, panelists, and moderators.

**Conclusions:**

Community and stakeholder engagement can directly impact research by enhancing clinical trial design, increasing relevance, and increasing recruitment, accrual and retention (Staley K.: Exploring Impact: Public 53 Involvement in NHS, Public Health and Social Care Research – INVOLVE.; 2009, Johnson et al Clin Transl Sci 8:388-54 390, 2015, Joosten et al Acad Med 90:1646-1650, 2015). The 2017 Advancing the Science of Community Engaged Research meeting, *Innovative and Effective Methods of Stakeholder Engagement in Translational Research* facilitated meaningful engagement of diverse stakeholder groups including racial and ethnic minorities, community and patient representatives, and junior investigators. Of 210 attendees, 72 completed the evaluation, and, of those, 36% self-affiliated as community members, and 21% as patient/caregiver advocacy, faith-based, or tribal organization members. This conference 1) represented a step toward expanding the scientific basis for the community engaged research (CEnR) field; 2) catalyzed innovative community engaged research; and 3) enhanced the reach and impact of the scientific developments emerging from pioneering work in community engagement.

**Electronic supplementary material:**

The online version of this article (10.1186/s12919-019-0164-y) contains supplementary material, which is available to authorized users.

## Background

Researchers and stakeholders need a forum to rapidly disseminate community engaged research evidence and to gain practical knowledge for implementing new, and enhancing existing, research programs. Because community engaged research is an approach that transcends disciplines and can be used in any phase of translational research, traditional scientific meetings that are discipline or subject area-focused likely draw just a limited number of community engaged researchers. A national community engaged research conference was held annually from 2008 to 2014. These meetings, led by Duke University Medical Center and supported by cooperative agreements with NCATS and NCRR, provided a key learning opportunity for community engaged researchers and were the primary opportunity for thought leaders and stakeholders to convene. The meetings were also a discussion forum around the “Principles of Community Engagement”, a 2011 HHS publication developed in partnership with the CDC [1]. The conference series ended in 2014, leaving a gap in national meetings focused on community engaged research. With a critical need to expand the scientific basis of community engaged research, [2] this left researchers and stakeholders with limited opportunities to share novel approaches, identify areas in need of further inquiry, and deliberate on key issues in the field.

Faculty at Vanderbilt University Medical Center and Meharry Medical College organized national meetings on “Advancing the Science of Community Engaged Research (CEnR)” in 2016 and 2017. These meetings brought together a diverse group of stakeholders to share community engaged research evidence and practical knowledge for implementing new and enhancing existing research programs. The conference series’ goals were: 1) to expand the scientific basis for the community engaged research field by convening researchers, community partners, patient advocacy organizations, and others to share innovative methods and strategies; 2) to engage community representatives and patient advocates in the development of new approaches in community engaged research by meaningfully involving them in the planning, as speakers and presenters, and as conference participants; and 3) to catalyze innovative community engaged research using interactive meeting methods that promote learning, support collective problem solving, and encourage new conceptual frameworks. These conferences have advanced community engagement across the translational research spectrum in biomedical research. For the 2017 meeting, described here, the overarching theme was *Innovative and Effective Methods of Stakeholder Engagement in Translational Research.*

A number of regional meetings by the Medical College of Wisconsin, University of Alabama- Birmingham, and Northwestern University have offered opportunities to engage regional stakeholders and implement approaches locally, but typically without emphasizing advancing the field of community engagement. Several national meetings that focused on community engagement itself appealed more to investigators who sought to gain capacity in community engagement but did not meet the needs of the broad range of community engaged research stakeholders. The biannual Community-Campus Partnerships for Health (CCPH) International Conference has brought together hundreds of stakeholders to focus primarily on partnerships and collaboration with less emphasis on research. The American Public Health Association’s Annual Meeting has drawn audiences of more than 12,000 attendees; however, the research focus is public health rather than community engaged research. The Patient-Centered Outcomes Research Institute (PCORI) held its first Annual Meeting in 2015. While the PCORI meeting actively engaged patients and community representatives, the conference was exclusive to patient-centered and comparative effectiveness research. In the 2015 Association for Clinical and Translational Science Annual Meeting, only one of the more than 30 sessions focused on community engaged research.

The Advancing the Science of Community Engaged Research conference series was distinctively different and separate from the prior series of community engaged research meetings and was planned with input from leaders of the earlier series to build on successful elements and gain insights from lessons learned. The 2017 Advancing the Science of Community Engaged Research meeting, *Innovative and Effective Methods of Stakeholder Engagement in Translational Research,* held at the American Association of Medical Centers (AAMC) in Washington, DC, extended the success of the 2016 meeting to teaching and learning about methods development and comparative effectiveness in the field, and facilitated meaningful engagement.

## Methods

### Overview

The 2017 Advancing the Science of Community Engaged Research conference brought together leaders in community engaged research, community and patient representatives, researchers across the clinical and translation research spectrum from basic scientists to community based researchers, plus faculty and staff from community engaged research programs of Clinical and Translational Science Awardees (CTSAs) and Research Centers in Minority Institutions (RCMIs), governmental agencies, and the private sector (payers, industry). The conference attracted thought leaders from a range of translational research disciplines and stakeholders – including community leaders and patient advocates with interest and experience in research

### Conference Format

The conference was a 1.5-day meeting with a combination of plenary sessions, (See Additional file 2) breakout sessions (Learning Labs and Think Tanks), a poster session, and networking opportunities. The 1.5-day format is commonly used for in-person scientific meetings and allowed attendees more than 10 hours of learning activity time. The format and agenda provided ample opportunity for attendees to network, convene new and existing interest groups, and have mentoring meetings. The “at-a-glance” view of the schedule is shown below.

### Structure of Conference Sessions

The learning activities were presented in the following formats:

*Plenary Sessions.* These general sessions covered issues critical to the science of community engaged research with presentations from distinguished speakers. Plenaries included 1-2 keynote speakers or a panel.

*Think Tanks.* These dynamic breakout sessions focused on timely and emerging issues relevant to the science of community engaged research and were moderated jointly by a community or patient representative and a researcher or academic stakeholder. With this structure, different perspectives were shared, participants engaged in collective problem solving, and ideas were feely exchanged with the goal of advancing community engaged research.

*Learning Labs.* These “how to” breakout sessions were forums for sharing locally successful innovative methods in community engaged research with participants from across the nation. Practical information to guide implementation of community engaged research approaches was delivered. Researchers and other stakeholders shared best practices and lessons learned in an interactive setting. Four concurrent sessions were held on day 1 and day 2 (8 total).

*Poster Session.* In the poster session, researchers, community partners, students, and travel scholars presented cutting-edge research and works in-progress.

### Session Content Development

The Organizing Committee played a critical role in determining the program content and was responsible for planning the conference schedule, selecting themes, and identifying plenary speakers and panelists. The Committee also guided promotion of the conference and stakeholder engagement. To ensure that the conference would be relevant to a broad audience, the Organizing Committee represented diversity in gender, race, ethnicity, age, disciplines, and stakeholder type. Members of the Organizing Committee for 2017 were:

Consuelo H. Wilkins, MD, MSCI (Chair); Executive Director, Meharry-Vanderbilt Alliance; Co-Director, Meharry-Vanderbilt Community-Engaged Research Core

Charles P. Mouton, MD, MS (Co-Chair); Professor of Family and Community Medicine, Vice Dean for Academic Affairs, Professor of Family Medicine, University of Texas Medical Branch

Philip Alberti, PhD; Senior Director of Health Equity Research and Policy, Association of American Medical Colleges

Karen Calhoun, MA; Community Health Coordinator, City Connect Detroit, and Michigan Institute for Clinical and Health Research

Chinenye Anyanwu, PharmD, MPH: Engagement Officer, Patient-Centered Outcomes Research

Rhonda G. Kost, MD, Clinical Research Officer, The Rockefeller University Center for Clinical and Translational Science

Lloyd Michener, MD; Professor and Chair, Department of Community and Family Medicine, Duke University Medical Center; PI of prior seven National Community Engagement Conferences

Eruera “Ed” Napia, EdD: Youth Programs and Special Projects Manager, Urban Indian Center of Salt Lake

Maria Pardos de la Gándara, MD, PhD: Postdoctoral Fellow, The Rockefeller University

Tricia Piechowski-Whitney, MPH, MSW, MA: Administrative Program Director, The University of Michigan, MICHR

Al Richmond, MSW; Executive Director, Community Campus Partnerships for Health (CCPH); Founding member, Community Based Public Health Caucus

Jaye Bea Smalley, MPA; Director, Global I & I Patient Advocacy and Life Cycle Management

Louisa A. Stark, PhD; Research Associate Professor, Department of Human Genetics, University of Utah; Co-Director, Community Outreach and Collaboration Core, Center for Clinical and Translational Science, University of Utah School of Medicine

Alvin “Hal” Strelnick, MD; Assistant Dean of Community Engagement; Professor of Clinical Family & Social Medicine; Program Director, Hispanic Center for Excellence; Albert Einstein College of Medicine

Rev. Neely Williams, MDiv: Chief Executive Officer, Community Partners’ Network

Melvin Thompson, MBA: Executive Director, Endeleo Institute

### Abstract Review Committee

The Organizing Committee identified individuals for a 15-member Abstract Review Committee, which comprised researchers, community representatives, patients and other stakeholders with knowledge and experience in research. The Abstract Review Committee reviewed abstracts submitted for the Learning Lab and the Poster Session. One third of the Abstract Review Committee comprised community members and patients. Drs. Wilkins and Mouton from the Organizing Committee screened all abstracts and assigned up to ten abstracts to each reviewer. An orientation for abstract reviewers was provided via web conference. At least two reviewers evaluated and scored each abstract. The Abstract Review Committee met virtually to review abstract scores, discuss abstracts with discordant scores, and select abstracts to be presented.

### Stakeholder Engagement

Because meaningful and substantive engagement of stakeholders is a fundamental principle of community engaged research we used best practices to ensure that community members and patients: 1) were involved in organizing the conference, 2) participated as speakers, discussion leaders, and attendees, 3) received appropriate compensation for work on committees, 4) had travel expenses paid if involved in the conference program, 5) had accessibility requirements accommodated, and 6) received information on family care resources. Plenary sessions were video recorded and posted on the conference website. When possible, access to plenary sessions was provided via free streaming video to allow virtual participation.

### Travel Scholarships

Costs and inconveniences of travel hamper community member and patient involvement in research conferences that able-bodied and travel-supported academic investigators attend with relative ease. Institutions were encouraged to provide travel support to community members and patients engaged in their research programs. The Meharry-Vanderbilt Alliance, with funding support from NCATS and NIMHD, provided 20 travel scholarships for community members and patients. [Table 1] The scholarship application included brief questions regarding experience and interest in community/patient engaged research, commitment to advancing community engaged research, and goals for attending the conference. Priority was given to stakeholders actively engaged in research.

**Table 1:** Travel scholarship Awardees by Institution and Affiliation

Travel Awardee	Institution	Affiliation
Ysabel Duron	Latinas Contra Cancer	Community Organization
Luther Evans	Flint, Michigan Community Leader	Minority-serving community non-profit
Jeanette Gonzalez	Gilda's Club Chicago	Patient/Caregiver advocacy organization
Tim Grimes	Resident, Kansas City	Patient advocate
Candace Henley	The Blue Hat Foundation	Minority-serving community non-profit
Abel Hopson-Suvlu	Arctic Slope Native Association	Tribal organization
Kimberlee Hyman	Campbell University	Faith-based organization
Tam Lutz	Native CARS/NPAIHB	Tribal organization
Ila McDermott	Community Advisory Board, Nashville Tennessee	Community organization
Jane Nguyen	Boat People SOS	Minority-serving community non-profit
Geraldine Peacock	Apostolic Faith Church	Faith-based organization
Prisciliana Quijada	California Department of Public Health-Office of Binational Border Health	Public health program
Natasha Ray	New Haven Healthy Start	Community organization
DeWaun E. Robinson	Artistic Visions Enterprise	Community organization
Antolin Rodriquez	Promotor de Salud / Community Health Worker	Community organization
Nicky Smith	The Bronx Community Research Review Board & The Community Engaged Research Academy	Public health program
MD Taher	DREAM Coalition, NYU Center for the Study of Asian American Health	Minority-serving community non-profit
John Taylor	Real Life Poets	Community organization
Cathy Vue	Asian Services in Action, Inc.	Minority-serving community non-profit
Tatiana Webb	Community Faces of Utah	Community organization

#### Promotion and Communication

The conference was promoted by email, mail, and social media. To reach our target audience, we specifically invited members of the following groups:

*Collaboration/Engagement Domain Task Force* – a CTSA task force focused on advancing the translation of research by engaging stakeholder communities and enabling team science. Members represent each of the 64 CTSAs and includes three community members.

*RCMI Translational Research Network (RTRN) Community Engagement Cluster* – a cluster focused on engaging communities to foster collaborative solutions for improving minority health and reducing ethnic and geographic disparities in health. Members are community engaged researchers at minority-serving institutions.

*Community-Campus Partnerships for Health (CCPH)* – a national non-profit organization that promotes health equity and social justice by supporting collaborations and partnerships between communities and academia.

*AAMC Research on Care Community (ROCC) –* a community of researchers, clinicians, and educators who share an interest in integrating research into care delivery.

*The National Patient-Centered Clinical Research Network (PCORnet)* – a national network of researchers, patients, and health systems focused on patient-centered outcomes research. (http://www.pcornet.org/).

*Partnership for the Advancement of Community Engaged Research (PACER)* – a special interest group of the Association of Clinical and Translational Science; comprised of researchers, community engaged research program managers and staff, and community members.

### Process Evaluation Results and Metrics

**Fig. 1:**
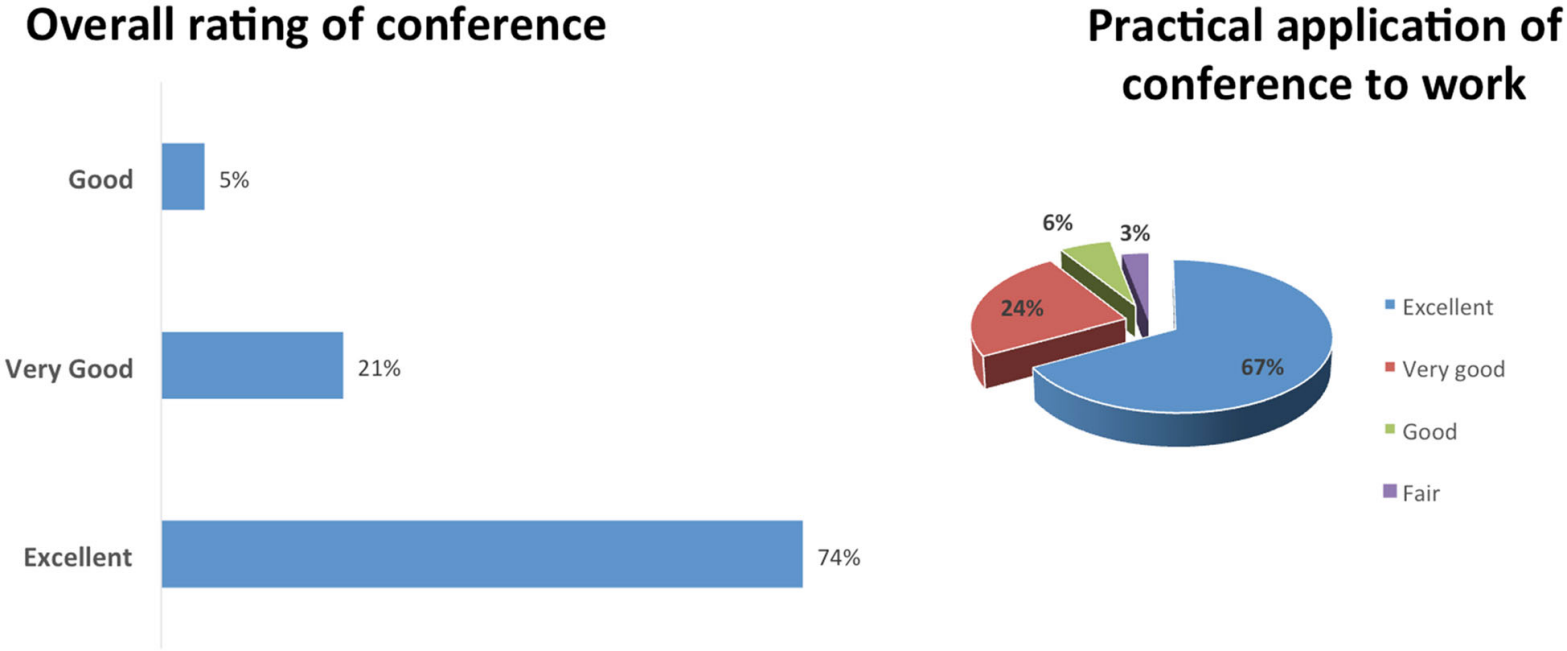
Overall Conference Metrics

A process evaluation was conducted of the conference operations, logistics, and fidelity to the mission and purpose of advancing community engaged research (CEnR). The evaluation response rate was 34% from conference attendees (72/210). The conference was rated “excellent” by 74% of respondents and 67% of respondents felt the conference scored excellent as a practical application of their work in community engaged research (Fig. 1).

The majority of the respondents (67%) scored the conference proceedings as “excellent” in discussing and describing approaches to improving community engagement in research. Overall, half of the respondents (51.4%) rated the conference as “excellent” in discussing and describing evidence-based methods, metrics, and tools to measure the impact of community engaged research. Notable topics for future conferences were provided, with several themes emerging in class and ability status as barriers to healthcare intersecting with racism; cross-sector CEnR collaborations in housing, business, and community development hospitals; and engaging the next generation/high school students to assist in changing community mindsets through youth.

#### Conference Website

A homepage (https://www.vumc.org/meharry-vanderbilt/advancing-science-community-engaged-research-conference ) on the Meharry-Vanderbilt Alliance website, included all pertinent conference information and was updated regularly by the communications coordinator. Webpages included conference location, agenda, registration information, and links for abstract submission. The website also included information regarding travel, hotel accommodations, accessibility information, and availability of family care.

#### Conference Mobile App

**Fig. 2:**
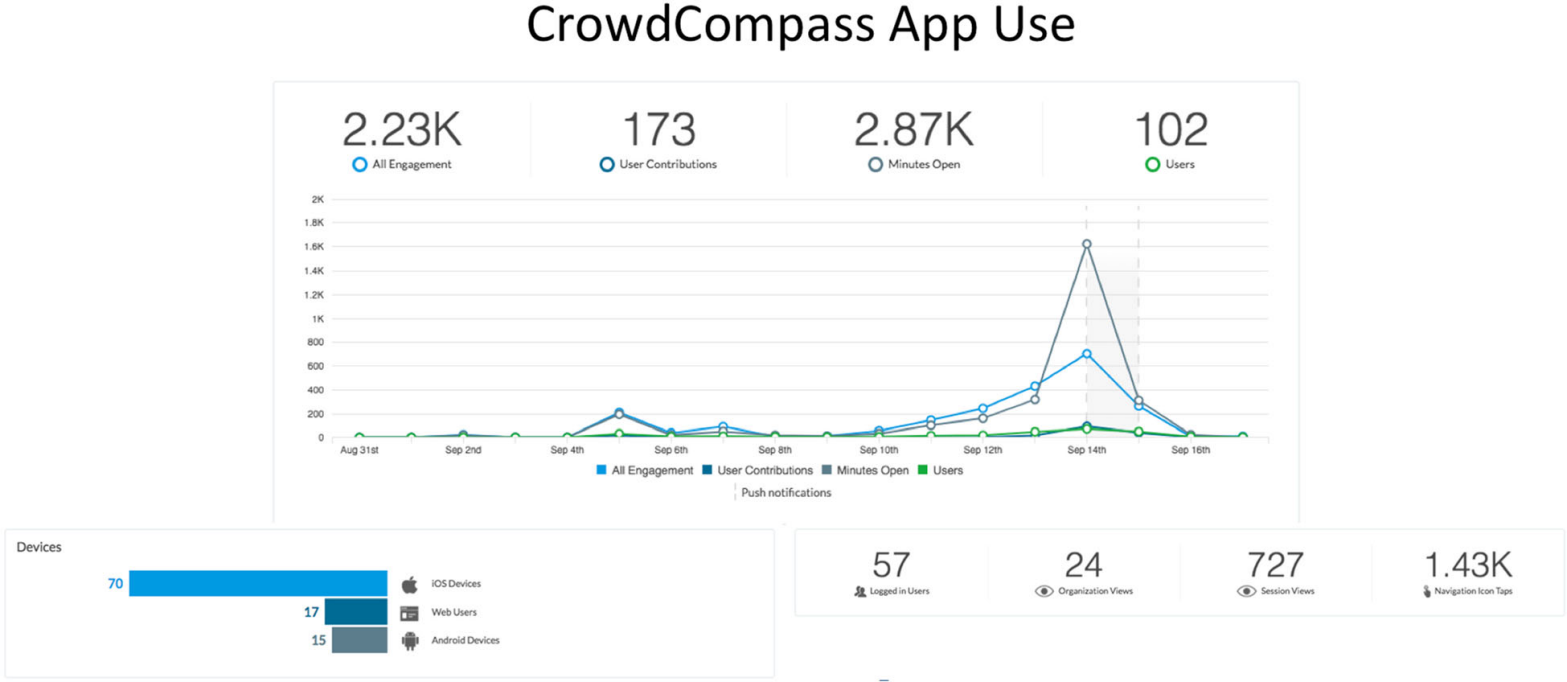
CrowdCompass App use

A mobile application, Crowd Compass provided the conference schedule, detailed speaker bios, maps of the conference location and hotels, and other conference materials. The app increased opportunities for attendees to engage via social media. Most attendees used smartphones and laptops during the conference and easily accessed digital information via the conference app (Fig. 2). At its peak on day one of the conference, the app was utilized 2,230 times by 102 users, further reinforcing the return of value on providing an app for accessibility and real-time information updates. The app hosted 173 user interactions, including instant messages, evaluation tools, and Continuing Medical Education (CME) materials.

### Proceedings

#### Advancing the Science of Community Engaged Research 2017 Conference

**Welcome and Overview**

**Consuelo H. Wilkins, MD, MSCI**

Executive Director, Meharry-Vanderbilt Alliance, Vice President for Health Equity, Vanderbilt University Medical Center

Nashville, Tennessee

Associate Professor of Medicine, Vanderbilt University Medical Center and Meharry Medical College

**Charles P. Mouton, MD, MS**

Vice Dean for Academic Affairs, Professor of Family Medicine, University of Texas Medical Branch

**Erurera "Ed" Napia, EdD**

Program Manager for Sacred Paths Youth Services and Special Projects, Urban Indian Center of Salt Lake

#### Opening Plenary Session: Innovative National and Statewide Community Engagement Initiatives to Improve Health

Panelist 1: *Dismantling Structural Inequality through Partnerships*

**Al Richmond, MSW,** Executive Director, Community-Campus Partnerships for Health

ccph.executivedirector@gmail.com

A description of the Community Campus Partnerships for Health was provided. This organization is grounded in the scholarship of service learning, community-based participatory research, and the role of partnerships. Originally, the individuals in this organization were primarily individuals in allied health professions, including physicians, nurses, dentists, and others who desired to take their academic skills and really work in communities to improve the health of those communities. These graduate/post-graduate/postdoctoral students, along with the Founding Executive Director, Sarena Seifer, felt the need to create this organization out of that spirit of advancing community-engaged research, but also out of the service aspect of that work. Over the last 20 years this leadership organization sought to more fully understand and promote partnerships as a platform for social justice and health equity.

Out of this visioning and thinking process, five pillars emerged: 1) Leading; 2) Convening; 3) Partnering; 4) Training; and 5) Disseminating. These pillars synthesize and catalyze who CCPH is as an organization. CCPH leads for social justice, convenes for change, partners for collective impact, and trains to enhance learning and best practices. CCPH disseminates for impact. The pillars are foundational ways in which the CCPH carries out the mission as an organization to advance social justice and health equity. The CCPH view on health is very broad. The view is to change the conditions of the environment in which people live, work, study, pray, and play. Communities play a central role in their own well-being.

The work of CCPH is fully vested in partnerships. Some are long-term partnerships and others are emerging. The lens of partnerships and the power of them was discussed with reference to the images of torches and racially-charged propaganda that occurred on or near the campus of the University of Virginia on August 12th and 13th, images of Nazi, Germany, and the fire-bombing that occurred at the 16th Street Baptist Church in Alabama. These images were referenced by the presenter along with conversations that he had with his grandmother as a child, who talked about the night rides that occurred in her community in South Carolina, as the members of the Ku Klux Klan rode through black neighborhoods with torches burning crosses. The images on the campus of University of Virginia highlighted a deep divide in the nation, as we struggle with a history of racism and antisemitism. While laws and legislation have passed to address these issues, the structural inequalities remain. The shocking, horrific, and deeply disturbing ideas of white supremacy and a racial hierarchy in this country as related to the campus of the University of Virginia on August 12th and 13th were discussed. This event brought further attention on academic institutions viewed as purveyors of thought and intellectual pursuit. The modified mission of the University of Virginia that was adopted in 2014 was read "The University of Virginia is a public institution of higher learning, guided by a founding vision of discovery, innovation, and development of the full potential of talented students from all walks of life". The work of the CCPH challenges the status quo to work in partnership to dismantle systems of structural inequality in this country. Dr. Camara Jones, the 2016 President of the American Public Health Association, was discussed as someone leveraging her position and platform to elevate racism as impacting the health of people in this nation. Dr. Camara Jones’s national campaign against racism was highlighted where racism is seen as a system of structure and opportunity and assigning value based on the social interpretation of how one looks. "Race" unfairly disadvantages some individuals and communities, unfairly advantages other individuals and communities, and saps the strength of the whole society through the waste of human resources. Dr. Camara Jones work [3] was cited as a primer when looking at dismantling structural inequality and understanding racism in our nation.

The presenter, Al Richmond (Community-Campus Partnerships for Health, USA) began an in-depth description of the "Structural inequalities: An On the Ground View Summer Intensive" convened by the CCPH. The intensive was a gathering of over 50 leaders from across the country from academic settings, community-based organizations, and faith-based organizations, during two days in the Summer of 2017 to deepen their understanding of structural inequality in this nation. This Summer intensive was held on the campus of a historically black college in Durham North Carolina (North Carolina Central University) to allow people who live outside of the deep south to experience familiarity with historically black colleges and universities (HBCUs). The rationale behind establishing HBCUs was revealed… these colleges and universities were not established simply because black people wanted to go to their own school, but they were established because they had no other choice. The truth of this school choice was simply stated: this nation had a system that did not permit black people to attend colleges and universities with white people. The location of Summer intensive was concentrated on the campus of historic Stagville, a plantation in Durham, North Carolina. Richmond noted that the visits to Stagville were intentional to expose people to what the genesis of structural inequality looks like. Some portions of the this intensive were in the deep woods on a plantation that at one time covered over 60,000 acres with thousands of enslaved people to illustrate that when left unchecked, this is what structural inequality looks like. Richmond emphasized that in his work, when talking about people who worked on a plantation, they are not referred to as slaves, as that is considered dehumanizing. They are referred to as enslaved people.

The definition of structural racism was provided as a system in which public policies, institutional practices, cultural representations, and other norms work in various, often reinforcing ways, to perpetrate racial group inequity. The history of social activism in this country among college students was acknowledged as an example on how to look at structural racism. Images from North Carolina of students sitting in Woolworth's in Greensboro were displayed. An image of PHD's, pre-health dreamers, individuals who were fighting for their right to remain here in the United States was then displayed. Lastly, images of students and communities working together at Standing Rock, standing together around social justice and health equity were displayed. The historic role of these images was touched upon and the responsibility of balancing research and communities and understanding the real issues that are impacting communities.

The CCPH principals of partnership were shared, highlighting “Transformative Experiences” and how we need to be engaged in creating transformative experiences for the people interpersonally, institutionally, and then as a community.

Panelist 2: *Community-Based Approaches to Improving Mental Health Equity in Underserved Communities*

**Sergio Aquilar-Gaxiola, MD, PhD**

Director, University of California-Davis Center for Reducing Health Disparities

saguilargaxiola@ucdavis.edu

Sergio Aguilar-Gaxiola (University of California-Davis, USA) opened his presentation with a concerted focus on the “treatment gap” in California. Sobering data were presented; between 50-90% of people with serious mental disorders have not received appropriate mental healthcare in the past year. Aguilar-Gaxiola presented the metrics of a collaborative study conducted in the Central Valley in California with people of Mexican origin where he found that of 1,000 people in need of services, one out of three of the U.S.-born people of Mexican origin utilized services, one out of six of the immigrants, and less than one in 10 of the migrant agricultural workers. Aguilar-Gaxiola stated that not much progress had been made, further emphasizing quotes from in-depth interviews with those who have suffered a decade or more with severe mental illnesses and no mental health care.

Evidence from previous studies intensified this issue. Aguilar-Gaxiola showed statistics that up to 75% of Latinos who get services once did not return for a second time. Aguilar-Gaxiola continued to show the sequelae of health circumstances that intensify over time for people with severe mental illness, pausing to note their average life expectancy is truncated by 25 years. Aguilar-Gaxiola shifted his presentation to detail the magnitude of mental illness and the trajectory over the next two decades, showing that, in the United States, about 50% of the population is going to suffer from one or more mental illnesses, including substance abuse.

A question was posed to the audience, "Why the treatment gap?" and some conclusions drawn from the research thus far were offered. Stigma was recognized as one of the major obstacles to seeking care. At the community level, lack of culturally and linguistically appropriate services was cited as a barrier.

Aguilar-Gaxiola shared with the audience multiple programs that he implemented in the state of California and continues to focus efforts on programs at the local, state, and national levels.

The first initiative Dr. Aguilar-Gaxiola shared. he termed a “Robinhood initiative”. is called the “Mental Health Service Act”, a law passed 12 years ago to increase funding for mental health programs. The mechanics of this law were revealed. Those who make one million dollars or more were taxed, effectively 46,000 Californians. The presenter shared that in 2016, the tax collected surpassed two billion dollars and constituted roughly 17% of the operating money for the counties.

An innovative community-initiated program was showcased as an example called the "California Reducing Disparities Project." The two phases of this program were discussed along with the budgetary allocation of millions of dollars to focus on the disparities of five populations; African Americans, Asian and Pacific Islanders, Latinos, LGBTQ, and Native Americans. This program benefited from 60 million dollars invested from the Mental Health Services Act to fund approximately 40 projects. The inception of this program was derived from "community-defined evidence" implemented by community-based organizations. The presenter continued to describe the programs, noting that resources are allocated for five years to raise the level of evidence for their continuation.

Aguilar-Gaxiola shifted his presentation to address the second phase of the California Reducing Disparities Project. As described, the second phase was an effort to fund promising practices and systems change recommendations to address disparities specifically in historically underserved populations.

A local level project in Solano County was brought to the audience’s attention. Aguilar-Gaxiola remarked that this project was innovative as it was the first county project to design a multi-phase innovation, training, and transformation project with culturally and linguistically-appropriate mental health services. The focus of this project was noted to improve access to mental health services and increase workforce diversity. Aguilar-Gaxiola quickly debriefed the audience on the three phases of the Solano County project. The first phase was formulated to bring together these three groups of stakeholders – the community leaders , the community-based organizations, and the counties. Aguilar-Gaxiola noted that the second phase of the project was to bring stakeholders together and train them with a curriculum that has an evaluation component. The training was described as developing quality improvement projects where specific action plans will be implemented in the third phase.

#### Special Plenary Session: Community Engagement in Minority Health Research to Reduce Disparities

**Eliseo J. Pérez-Stable, MD**

Director, National Institute for Minority Health and Health Disparities

eliseo.perez-stable@nih.gov

Dr. Eliseo Pérez-Stable (National Institute for Minority Health and Health Disparities, NIHMD, USA) was introduced by Dr. Meryl Sufian, PhD, a Program Officer with the National Institute for Minority Health and Health Disparities (NIMHD) in Integrative Biological and Behavioral Sciences. Dr. Pérez-Stable’s bio was read noting his career highlights of being the Director of the National Institute of Minority Health and Health Disparities for just two years leading research in smoking cessation in Latinos for over 30 years, both in the United States and Latin America. Dr. Pérez-Stable was a Professor of Medicine at the University of California, San Francisco. He earned his Bachelor's Degree in Chemistry from the University of Miami, his M.D. also from the University of Miami, and he completed his primary care internal medicine residency and research fellowship at UCSF.

The history of the National Institute for Minority Health and Health Disparities (NIMHD) was detailed from its origins where the NIMHD started in 1990 as an office of the NIH Director as a consequence of Dr. Sullivan [*then Secretary of the U.S. Department of Health and Human Services.* A critical step came about in 2000, led by representative Stokes [*Representative Louis Stokes, D, OH*] in Congress, the NIMHD was elevated to a center with the ability to fund its own grants. A requirement emerged in 2010 as part of the Affordable Care Act that all the agencies in Health and Human Services have an Office of Minority Health. The Office of Minority Health exists in the Secretary's office, defines health disparity populations and advances research and policy priorities that are important for reducing health disparities in the United States.

The NIMHD structure and mission were presented, where the NIMHD was elevated to an institute with the dual mission of an interest in minority health and health disparities and to distinguish those two areas: Minority health was defined as everything related to racial-ethnic minority groups in the U.S., whether the outcomes are good or not good. Health disparities were defined as outcomes that are worse in disadvantaged groups and to use that knowledge to develop interventions to reduce the disparities.

Pérez-Stable, as the Director of NIMHD noted he was empowered to decide who is a health disparity population and mandated to consult with the Director of the Association of Health Research and Quality. In October 2016 sexual gender minorities were declared a disparity population for NIH research purposes. The dearth of data on long-term outcomes for sexual gender minorities has escalated this as a priority for the research community. Pérez-Stable offered the challenge of measuring the concept of being “poor” and his personal preference to use education as a simple, one-question item. Pérez-Stable stated that this is an imperfect measure but fairly robust across the lifespan in adults, as opposed to income, which varies quite a bit. A visual example was then provided to show how research does not do a very good job at addressing social class in human research as a powerful predictor of health.

The NIMHD framework displays possible mechanisms leading to health disparities was depicted with variables of individual behavior, coupled with, racism and stress indicators, adverse conditions, the adverse effects of childhood adversity (effects on adult health) and the effect on biological processes. The elements in this framework were heightened with the empirical evidence on the importance of the "built environment" or the "physical environment." Pérez-Stable stated how the importance of where you live - broken windows, trash on the street, safety, sidewalks - are factors that scientists have begun to incorporate into public health science and behavioral science. Mobile technology was referenced as a factor in the cultural environment. Pérez-Stable posed the question for thought - how do you blend all these together?

The need for the NIMHD to be in the research space focused around minority health and health disparities was emphasized and the ability to ameliorate disparities in the clinical setting in the laboratory of doctor/clinician/patient communication. Reference to the NIMHD framework on the website was made to express the interest and the complexity, of the research the NIMHD is focused on. The framework was further qualified as a guide not an explanatory or causal model.

The next topic of discussion was presented as diversity in science. Statistics were cited to emphasize the need for diversity in science. In 2015, somewhere around 12% of medical school graduates were either Latino or African-American. Pérez-Stable noted that by adding in American Indians and Pacific Islanders, the percentage is barely bumped up a half percent. This scenario was also described in the in the biomedical scientific workforce, where about 7% of principal investigators at the National Institute of Health are either black or Latina.

Diversity of research participants in scientific research studies was broached [4] where Dr. Pérez-Stable issued the message that, to advance knowledge including diverse samples is vital. Pérez-Stable noted one way to facilitate inclusion was community engagement where scientists have to go out and engage the community and reciprocally, the community has to be willing to engage with meeting in a common ground.

Pérez-Stable informed the audience that social justice is good science and common sense mandate inclusion because 40% of the population, by 2020, will check one of those boxes that is not White. He identified resources and feedback systems to make NIH studies be more accountable. Dr. Pérez-Stable went further to identify a system in place that updated and monitored minority inclusions in funded grant proposals. He stated his preference to have a reward for a scientist who does an extremely good job of having diverse samples.

Powerful examples were displayed showing the big effects of race-ethnicity observed and supported by empirical data. [5, 6] Smoking cigarettes was shown and the differential effects of nicotine metabolizing to cotinine at different cut-points if someone was Mexican, Latino, White or African-American. The values of what defines a biochemical smoker were showcased as a research tool. Body Mass Index (BMI) was shown as another example where a screen of 23 works for Asian/Pacific Islanders for diabetes, because at a lower BMI they have higher metabolic risk. Other provocative examples were shown from large scale studies in minority populations and presentations of chronic diseases.

The infrastructure at NIMHD was further illustrated to show the three functional branches: biological behavior sciences related to mechanisms and etiology (not basic science), community health and population science, and clinical and health services research. A couple of examples of studies funded in the field of community-engaged research were detailed.

Pérez-Stable switched focus to talk about racism and discrimination as a model of chronic stress using various dimensions to illustrate the point; interpersonal, structural racism, historical, cultural and institutional practices. Dr. Pérez-Stable urged the audience to acknowledge these in their research and that racism is internalized in a way that would affect health through biological responses which may be unmeasurable.

A list of biological measures that minority health and health disparities research scientists are trying to address was presented for thought, with reference to studies of cardiovascular reactivity and the importance of telomere sleep as an important biological function of the human body.

Dr. Pérez-Stable presented some encouraging data, showing from 2000 to 2014 African American life expectancy increased by 4% annually [7, 8]. He referred to visual data where for people over 65 a crossover was revealed and African Americans do a little bit better in mortality than their white counterparts after 65 [9]. 

Pérez-Stable ended the presentation with some of his opinions where he stated policies to reduce health disparities in the healthcare setting landed on expanding access and coordination of care. Pérez-Stable expressed his view that the patient-centered medical home was an effective way of creating expanded access with information systems. Pérez-Stable concluded with lessons he learned from a manuscript by Dr. George Mensah [10] on how to use population data to identify hot spots in public health to target the most vulnerable. Vulnerable was defined as the elderly, minorities or poor. Pérez-Stable also summoned the researchers in the audience to recognize the resilience that exists in communities and to learn from communities, by actually going into the community and employ the members, engage the leaders and create partnerships.

#### Plenary Session II: Precision Engagement- Approaches to Involving Diverse Populations in Precision Medicine – A Panel Discussion

Moderator: **Lloyd Michener, MD**, Duke University

Panelist 1: **Usha Menon, PhD, RN, FAAN,** Associate Dean for Research & Global Advances, University of Arizona College of Nursing (currently at University of South Florida): *From the Frontline: What does Precision Medicine Mean for Me?*

umenon@health.usf.edu

Dr. Menon (then of University of Utah, currently at University of South Florida, USA) opened her session by discussing the importance of engagement to the National Institute of Health’s (NIH) *All of Us* project, a multi-year study that intends to recruit 1 million participants that, as a group, represent the diversity in the United States. The term engagement was defined in the context of the work of *All of Us* as education, consultation, communication, extension, partnership, participation. She discusses how engagement cannot be effective if it stops with outreach, because that is unidirectional. Menon stated her approach as wanting to make engagement a bidirectional conversation fostering the norm of inclusion. The concept of precision medicine was discussed as it was defined from the focus groups convened in the initial inception of the All of Us research program. It was revealed that people wanted to be included, that they wanted their voices included at levels beyond just putting them on an advisory board and asking their opinion every three to four months. The current approaches of the *All of Us* program were highlighted to reorient toward maximizing diversity through targeted inclusion, reaching hard-to-reach populations, and using very thoughtful inclusion and exclusion criteria

The Arizona Board of Regents Tribal Consultation Policy was showcased an example of fostering inclusion working within the constraints of what a Board of Regents put forth in governing engagement with the American Indian community. Menon noted, before communicating with the tribes about any kind of research, all 22 tribes were invited to the table to have a conversation. The example was brought up to reflect the needs and preferences and priorities as well as opportunities to interact with potential participants. Setbacks with the community were discussed and the author Lewis Grizzard was referenced [11]. Menon acknowledged that setbacks happen and how to learn from mistakes and go back to the community to apologize. It was posed as to whether there was a formula for engaging with communities starting with understanding cultural competency. This approach was linked to precision medicine and the *All of Us* research program strategies and program brochures. Several strategies were presented and discussed at length, visual strategies, evidential strategies, linguistic strategies, sociocultural strategies. A reference was made to using visual strategies - colors, images, graphics and that people want to see people who look like themselves. Evidential strategies were discussed with the strategy of enhancing the perceived relevance of the health issue. Menon clarified that is not one health issue as the topic precision health with a million+ cohort that will generate hundreds of studies on hundreds of issues and many diseases and health conditions. Linguistic strategies were presented as strategies translating into the language of choice for that individual. This strategy was further explored in regard to vernacular and idioms that people use within their culture to be appropriate to use with all audiences. Sociocultural strategy was cited as recognizing a group's cultural values, beliefs, and behaviors, and understanding that they should be recognized, reinforced, and leveraged to give context rather than to assume that there is a formula.

Menon cautioned the audience that they are setting a precedent, not just for themselves but for other community engaged professionals, coming behind them. The key message the presenter conveyed was the difference between equality and equity and discussed how a focus on equity can keep this large NIH project from creating more health disparity than already exists.

Panelist 2: **Chanita Hughes-Halbert, PhD,** Associate Dean for Assessment, Evaluation and Quality Improvement, Medical University of South Carolina: *Academic-Community Partnerships for Precision Medicine Academic-Community Partnerships for Precision Medicine* hughesha@musc.edu

Dr. Hughes-Halbert ( Medical University of South Carolina, USA) opened her discussion on the landscape around precision medicine and academic community partnerships within the context of the MUSC [*Medical University of South Carolina*] Transdisciplinary Collaborative Center in Precision Medicine and Minority Men's Health.

Hughes-Halbert revealed the motivation of her transdisciplinary research for focusing on minority men from a landscape review of the research in this area and how minority men were underrepresented with respect to the larger health disparities. She describes the NIMHD-funded center Transdisciplinary Collaborative Center (TCC) in Precision Medicine and Minority Men's Health, that focuses on transdisciplinary research in the health areas that most affect these men in the United States - namely heart disease, stroke, diabetes, and HIV. The Center includes investigators from many disciplines, including medical oncologists, geneticists, bioinformaticians, nursing science, and individuals with expertise in dissemination and implementation science.

The infrastructure of this TCC was further detailed naming several different types of academic institutions of the University of Pennsylvania, Hampton University, a leading a TCC in Minority Men's Health, The University of Texas Health Sciences Center in San Antonio for its focus on minority health from the perspective of Hispanic populations. Hughes-Halbert noted inclusivity of academic institutions but deliberately engaging diverse types of community-based organizations, the Low Country Area Health Education Consortium, the National Black Leadership Initiative on Cancer and the Hope Institute based out of Baltimore, Maryland, which focuses on ethical, legal, and social issues with respect to inclusion and diversity in clinical trials. Lastly, the partnership with the Southeastern Health Equity Council was mentioned.

Hughes-Halbert shifted her discussion from the TCC infrastructure to unique stressors that minority men face, both acute stressors and chronic stressors. The roles stress and stress reactivity and their respective impact on the initiation and progression of disease were interrogated. Hughes-Halbert remarked that the investigators at the TCC were interested in establishing markers to understand biological functioning. This was noted as the overarching framework of the TCC, to understand the allostatic process and how it influences disease initiation and progression. The decision as a center to focus on prostate cancer as a condition that had a significant clinical and public health impact and also a condition that was relevant for community stakeholders and individuals in the community was discussed. Prostate cancer as the TCC disease focus was further highlighted as it had biological, clinical, and public health relevance to other chronic conditions. Several publications from the center were highlighted including research on utilization of prostate cancer screening among African American men. The findings of this paper were provocative and found that, although there was a recommendation that men engage in an informed decision-making process, this did not play terms of how men were making decisions about prostate cancer screening. Other research out of the TCC was showcased, the continuum from screening to diagnosis to treatment revealing differences in quality of life following prostate cancer diagnosis and how the differences are influenced by men's sociocultural factors, spotlighting religiosity and temporal orientation as important indicators on how men react to being diagnosed with prostate cancer.

Hughes- Halbert early work focusing on genetic counseling and testing for inherited breast cancer risk was presented in the vein of her psychosocial behavioral research on understanding intentions to donate to Biobanks and intentions to participate in precision medicine research, Two examples were shown noting a lot of variation. Hughes-Halbert noted about 23% of those are "very likely," to be willing to participate or donate to a Biobank , until you ask them or give them more information about the attributes of the study, what it would actually involve in terms of their participation, it is much less likely with only 31% saying they would participate in a study that was sponsored by the government where they had to give a biospecimen or complete a survey about their personal and health history and that they would not get results back. Hughes-Halbert mused, that if individuals were provided more information about the research, less of a willingness to participate emerged . Hughes-Halbert noted she was really excited to see some of the work to engage populations in the *All of Us* research program.

Hughes-Halbert went on to share the organization of the TCC and the roles and challenges that have been experienced. The responsibilities of the Consortium Core topped the discussion on this being the overarching infrastructure that provides direction , sets the priorities and governance. The Consortium Core was noted to implement strategies and develop a research program that addresses the priorities that have been identified with respect to precision medicine. Hughes-Halbert continued to discuss other elements of the TCC including the implementation core and the data integration core. The implementation core was charged with developing materials and methods for determining readiness and capacity of different stakeholders across regions to implement and adopt precision medicine approaches and to clinical care and public health practice. The Data Integration Core was slated to integrate the data generated through all TCC projects for translation into effective strategies for precision medicine. So, as we have taken a step back, we are now in our second year of our center. Hughes-Halbert then discussed integrating all of the activities mentioned earlier on engaging the academic and community partners effectively and meaningfully in the TCC by setting the tone through the overarching framework. The development of strategies was presenters with thought on how they could be implemented into care - public health practice, clinical care. Hughes-Halbert stated that ultimately the work of the TCC should be translated that down into evidence-based recommendations or policies that are implemented in healthcare systems and prevention programs. A definition of precision medicine was presented which Dr. Hughes-Halbert noted really resonated with the academic and community members of the TCC consortium core and the constituents in each of their four activities and center projects.

The TCCs established goals for community engagement were presented. Goal highlights were listed as: 1) to provide information that people can use to increase their knowledge and awareness level of precision to develop their capacity to apply it to the healthcare or clinical practice decisions ; 2) develop and engage different stakeholders to make informed decisions about their participation in precision medicine research and/or referring members or patients for participation in studies; and the ultimate goal 3) to ensure that the use of precision medicine doesn't result in exacerbating racial and ethnic disparities in healthcare and outcomes.

One last engagement strategy was shared that the presenter noted they were very excited about; an Evidence Academy that focuses on knowledge translation for the purpose of developing tailored implementation strategies. Dr. Hughes-Halbert went on to describe the Evidence Academy as structured around sort of a co-learning process where individuals present their understanding, bring everyone to the same baseline understanding about a particular issue, and then work to develop best practices that are realistic for their particular type of setting. The Evidence Academic dissemination approach was illustrated through its regional implementation teams. The team were further detailed as including healthcare providers, community public health officials, and individual patients.

Panelist 3: **Karriem S. Watson, DHSc, MS, MPH,** Senior Research Scientist, University of Illinois at Chicago Cancer Center, Director of Community Engagement and Implementation Sciences: *Advancing Trust to Engage Diverse Participation in Research: Community Dialogue and Partnerships*

kwatson@uic.edi

In this presentation, Dr. Karriem S. Watson (University of Illinois at Chicago Cancer Center, USA) discussed the value of dialogue, conversation, and listening as he unpacked the precision engagement concept. Watson introduced himself as son of an Apostolic Deacon and a COGIC [*Church of God in Christ*] parents. He referred to the formal title of his presentation as "Advancing Trust to Engage Diverse Participation in Research: Dialogue and Partnerships, and then noted in the tradition of the black church, the sub-title, would be, "Who goin' check me, boo?" The genesis of Dr. Watson’s work was described with the concepts of advancing trust through his academic institution of the University of Illinois Cancer Center. He identified himself as a community-engaged researcher very passionate about precision medicine, precision health, and precision engagement, Dr. Watson noted that he lived in Chicago, and explained the concept of, "Who goin' check me, boo?" as the community partners checking you through dissemination the message.

Watson continued to speak on partners at the table, discussions of precision medicine and the lost art of conversation and listening. A story of a community partner and breast cancer survivor, Miss Rose Marie was shared and her interactions with grant agencies such as the DOD, (the Department of Defense) which elevated her from being a survivor to being an advocate. Watson made reference to Dr. Robert Winn, the cancer center Director of the University of Illinois Cancer Center and the movement to change the name his office from The Office of Community-Engaged Research and Implementation Science to The Office of Community Involvement and Implementation Science effectively advancing “engagement” to “involvement”.

Watson continued his presentation and his role on the *All of Us* research program [12] where he illustrated how he capitalized and leveraged the work done in Chicago in the communities around precision medicine. Watson detailed the interactions he had with community members through the Community Advisory Boards at the UIC-CCTS [13] where he was “checked” and advised to not start conversations with black men in Chicago and just ask them to donate their saliva. Watson sagely noted he was redirected to inform community partners that if he can connect saliva with West African ancestry, knowing black men’s West African ancestry gives a greater risk of prostate cancer, then science might be one step closer to addressing the mortality gap in African-American men.

The importance of beginning to catalog best practices such as the approach above was discussed. Examples of community member quotes were provided. A concern voiced by community members was conversation fatigue, the same thing being said in different venues without moving the conversation forward. Researchers must be better at noting who has been saying this and learning what they heard.

Watson described picture examples of two different groups, both community, and both initiated by community stakeholders. Watson noted they have partnered with industry and academic partners with similar conversations of trust, and have convened community members with successful outcomes. In May 2017, Community Campus Partnership for Health( CCPH) partnered with University of Illinois-Chicago ( UIC) Cancer Center and Northwestern University Cancer Center for Community Dialogue, and in July 2017, they partnered with Sister’s Network Chicago.

Instead of trying to invent new conversation, the presenter believed researchers may be able to be a part of those conversations with the community. Watson quoted the great granddaughter of Henrietta Lacks, Veronica Robinson: “Chicago, everything I’ve seen today, Mile Square, you are keeping the community engaged, you are exactly what we are about.” Data were presented showing 659 nine people registered for the Community Dialogue, and there were more than 500 attendees. There were three official welcome letters from federal officials: Senator Dick Durbin, Congressman Danny Davis and Congresswoman Robin Kelly. Representatives from those elected officials’ offices attended and joined in a private lunch, along with UIC leadership, several Patient Brigade members and city/academic/medical institutions including Illinois Medical District, Rush, Northwestern University, Cook County HHS, Harvard/Duke, Howard Brown, Elevate, AIDS Foundation of Chicago, City of Chicago and Pfizer. Descriptive and demographic data were displayed on the respondents, 76% were women and 24% were male.

Data on the final Likert fortified the Community Dialogue efforts. The question was, “Will conversations and town halls like the one today will continue to advance research?” Of the respondents, 81% strongly agreed, 16% agreed, 1% disagreed and 2% strongly disagreed.

Watson quoted Robert A. Winn, MD from a Chicago Magazine interview: “If loving science is wrong, then I don’t want to be right. But in that science, there is a framework of social justice. Science devoid of social justice may even be considered bad science.”

The theme that emerged from the Community Dialogue was noted as “trust” and this was echoed in quotes the presenter shared from community members: “Continue to promote community awareness and engagement. Trust will come when people know who you are and what you do. Make things are clear and easy to understand as possible. Appeal to the public interests. Thank you for all the great work that you do!!!” [sic]. Watson then quoted another community member “In conferences like this, healthcare providers should step up, acknowledge and take responsibility and assure the communities that it won’t happen again. Watson quoted a community Timothy Francis Jones AKA DJ Tim Buck2 a participant on a kidney cancer clinical trial who did not survive his fight: "Your struggle is my struggle, and I'm here to fight with you. To the ones with too much pride, and don't like going to the doctor because they are too proud and can handle it all, I have high hopes that my journey and story brings you back down to earth.” Watson noted that although Timothy did not survive his participation furthered the science towards a cure for kidney cancer. Lastly, the presenter honored the life of Dr. Cynthia “Cee” Barnes-Boyd and her legacy.

#### Plenary Session III: Data as the Driver-and Passenger- for the Community Engaged Research Vehicle – A Panel Discussion

Moderator: **Melvin Thompson, MBA**, Executive Director, The Endeleo Institute

Panelist 1: *Big Data Research Ready Communities in Chicago*

**Regina Greer Smith, MPH, LFACHE**

President, Healthcare Research Associates, LLC

healthcareresearch@sbcglobal.net

Ms. Greer-Smith ( Healthcare Research Associates, LLC, USA) opened the session by noting her one-person organization called Healthcare Research Associates of which she is the President, CEO, Chief Engagement Officer, self-titled "engagement activist." Greer-Smith presented the journey of her advocacy work through her 20 years in the south side and south suburbs of Chicago in partnership with the Southland Ministerial Health Network. Greer-Smith highlighted 2012 as pivotal year when her collaboration with the Patient Centered Outcomes Research Institute (PCORI) merged with the Southland Ministerial Health Network. Greer-Smith noted to the audience her incredulousness that an organization funded by the Affordable Care Act sought patients to be the center of research combined with a strong focus on African-American communities and communities of color and underserved people. The conversations shared between Ms. Greer-Smith and her partners in the faith-community were revealed. Greer-Smith recounted where she sat with the pastors and they reminisced about the health fairs they convened to bring awareness to the burdens in their communities. Greer-Smith stated that she took these conversations further with the pastors, noting that PCORI supported the pastors as partners in designing and participating in the research. Greer-Smith paused to note this was where the name of their group, 'Pastors4PCOR' was born. Greer-Smith then walked the audience through the process through which her group, Pastors4PCOR, gathered community health data to serve as a resource for research.

Greer-Smith continued elaborating on the development of “Pastors4PCOR” through its 501c3 status, its collaboration with Dr. Rebecca Johnson at Osher Center of Northwestern University for fostering relationships to build capacity for a robust research infrastructure in the community for faith-based communities to engage in health research. Various photos were displayed of Pastors4PCOR in action. The organizational structure was described as a faith-based, community-based, two academic medical centers, and policy institute.

September 2015 was identified as the beginning of the research training for members of Pastors4PCOR, who were then referred to as "Research Ministry Ambassadors," Ms. Greer-Smith then detailed Pastors4PCOR roadmap starting with a five-module curriculum to train Research Ministry Ambassadors how to get involved with “big data” in research.

The focus of the talk then shifted to module 3, step 3 from the roadmap which honed in on "Big Data." Three goals were set for the Big Data Training: Goal 1 was described as identifying where the big data about health outcomes and relative factors comes from and for Pastors4PCOR to feel competent engaging big data projects on their own data. Goal 2 was set as determining what the priorities were in the faith communities, identifying where the data came from and ultimately developing a survey to identify the health conditions and factors which were priorities in our faith communities. Goal three was to ascertain the tools to conduct the survey on health conditions and factors impacting our faith-based communities.

Greer-Smith provided an exemplar for how community members can learn “big data” and share that with researchers who can study and impact the health of that community. Examples were cited from a survey on the health burdens and disparities in each of the zip codes in Chicago subsetting to the PCORI-style big data. PCORI projects in Chicago were reviewed showcasing a stroke program between Northwestern University and Rush University. The PCORI's Patient-Powered Research Network (PPRN's) in Chicago were highlighted for their features that invite patients to join including the Healthy Heart Alliance, a large pragmatic study "ADAPTABLE" and the "ABOUT" network.

Greer-Smith elaborated on Goal 2, developing a survey to be shared with the community. This process was described as devising survey templates based on the faith community health conditions and priorities The presenter emphasized that Pastors4PCOR went directly to the churches to support them to develop survey messaging. Greer-Smith laid the foundation on this process with the dissemination plan of the survey development for the Research Ministry Ambassadors to deliver it to their respective congregations during Sunday service and Bible study including an evaluation component. The most compelling results to one of the process evaluation questions “what are the resources your faith-based community needs to address the health conditions and factors you identified”? were shared as education, programs, and research ... places and facilities needed in the communities (considered neighborhood factors), information and communications, access to affordable health, finances, and people. In the closing, the presenter stated a testament to community-driven data collection, “We got our own data, we did it our own way, and now we can share it with the world.”

Presentation 2: *Data as the Passenger: The Importance of Identifying What Data Matters to the Community*

**Courtney Clyatt, MA, MPH**

Program Officer, Patient-Centered Outcomes Research Institute

cclyatt@pcori.org

Ms. Courtney Clyatt( Patient-Centered Outcomes Research Institute) opened her presentation with an introduction to the audience of two programs developed with the Patient Centered Outcomes Research Institute (PCORI) engagement team that provide funding to ensure that community voices also inform research; the Eugene Washington Engagement Awards that encourage active integration of community members in research and the Pipeline to Proposal Awards which provide funding to build capacity for community engaged research teams to develop proposals with sound scientific rigor and robust patient engagement.

The PCORI vision was shared where patients and the public have information they can use to make decisions that reflect their desired health outcomes Clyatt operationalized this concept further as engaging patients, clinicians, and caregivers in the entire research process from topic prioritization to proposal review, Merit Review Program, then dissemination and implementation of results. Clyatt detailed the PCORI vocabulary stopping to define comparative effectiveness research as comparing two treatments or two diagnostic tools, to help patients figure out which one will work better for them.

Two PCORI funding programs that support projects that bring together communities, including patients, caregivers, and clinicians were at the crux of this presentation. The Pipeline to Proposal Awards, established to build a national community of patients, stakeholders, and research partnerships that have the expertise and the drive and the passion to involve their communities in the research development process. An example program Pipeline program was presented out of New Jersey with a focus on sickle cell anemia and a identifying patients biggest issues as dealing with pain and the medication that they must take. The "Cystic Life," patient-led project was then detailed for its focus on cystic fibrosis and looking at cardiovascular exercise as a treatment option. Clyatt introduced the audience to the clinician-lead project #LCSM on lung cancer and lung cancer treatment after surgery where the research question was sourced from a Twitter chat .

Clyatt dissected the PCORI research process strongly emphasizing the pre-planning stage and the need to include the community in capacity-building . Clyatt articulated the likelihood that patients and the community partners are engaged in the beginning of the research process will ease the dissemination of research and participation in the implementation of study findings.

Clyatt closed with this thought, that if the data system doesn’t reflect the full patient experience, living with a specific health issue, then that data system is not fully integrated and does not have the patient perspective.

Panelist 3: *Little Data, Big Data: Translational Research Partners Across the Spectrum*

**Jonathan N. Tobin, PhD**

President/CEO, Clinical Directors Network, Inc.

jntobin@cdnetwork.org

The results of Dr. Jonathan N. Tobin’s (Clinical Directors Network, Inc, USA) research project were discussed first with reference to the methods to allow insight on translational research partnerships. Two cases were presented; a study in “small data” (a few participants engaged in a behavioral intervention implemented by community health workers) and a study in “big data” (an observation in electronic health record data over 10s of thousands of pregnant women of the life course and trans-generational patterns of metabolic disease). Tobin framed his presentation using the translational research spectrum spanning T0 to T7, noting the current presentation ends with T4 translation to community with population level impact. The infrastructure contributing to the research studies was acknowledged, the practice-based research network infrastructure of the Clinical Director's Network, and the CTSA infrastructure, The Rockefeller University Center for Clinical and Translational Science. Common elements between these two infrastructures were highlighted in terms of training and in terms of research, with the real differences appearing in the focus of the institutions. The practice-based research network focus [14] features clinical outcomes and comparative effectiveness research and training investigators who are practicing clinicians, The Clinical Translational Science ( CTSA) engagement award, was shown to have a focus on patients becoming investigators and colleagues and the CTSA academic training focuses encompasses clinical scholars, postdocs, medical students, physician scientists.

Kost’s publication, "The Community-Engaged Research Navigation Process," [15] served as a centerpiece to introduce the audience to the CEnR-Navigation Process ( CEnR-NaV) an open cycle that allows investigators and teams to join in the research enterprise at any at stage and move into a collaboration that produces community engagement, community-engaged research, and eventually comparative effectiveness research, and patient-centered outcomes research. This model embodied unique additions to actually embedding T0 or mechanistic questions within the framework of comparative effectiveness research and clinical outcomes study.

The first case study was presented as "The Patient-Centered Comparative Effectiveness Study of Home-Based Interventions to Prevent Community-Associated MRSA Infection Recurrence", referred to as "CAMP" throughout the presentation. The variety of stakeholder involved in this project was illustrated: patients, caregivers, family members of patients, community health workers, practicing clinicians, academic and laboratory investigators, statisticians, and informaticians, several large public hospitals in New York City and several large federally-qualified health centers, Rockefeller and CBN. During his presentation, Dr. Tobin observed that translational research critically depends on listening closely to the needs of all stakeholders — priorities, obligations, and driving incentives — which entails deep engagement. He emphasized the need to define outcomes across the full biomedical research spectrum, from the biological, through the clinical and patient-centered, and to the health services public health measures. Deep engagement in grant writing was provided as an example using an approach that involves discussion teams consisting of a laboratory investigator, a health services researcher, a clinician, and a patient. Topics the groups discuss include patient engagement, patient recruitment, incorporating patient needs, defining patient outcomes. One monthly meeting of case presentations by a clinician struck the presenter. A case where a patient was very upset with the clinician because she has had yet another recurrent MRSA infection despite incision and drainage and antibiotics. This case presentation intensified as the patient’s sister was visiting and came down with the same infection. This example was provided as a galvanizing experience for the group to try to understand the predictors of recurrence. This case presentation was then tied to the CAMP observational cohort data where, 40% of patients, in spite of most of them receiving and following CDC-defined treatment guidelines, were developing recurrent infections if they had methicillin-resistant Staph or methicillin-sensitive Staph infections. This puzzling situation evolved into a two-armed randomized control study ( RCT) to examine the comparative effectiveness of promotoras and community health workers training patients to implement the ICU protocol for preventing MRSA versus usual care, where the participating sites across New York City and Westchester County received the CDC guidelines care. The intervention was further detailed to describe the experimental group, which received materials related to decolonization (removing evidence of the bacteria from parts of the body that are not infected) and decontamination (removing the bacteria from surfaces in the household to reduce the overall risk of infection). Surveillance measures were collected in the households of patients 278 randomized patients including 13 high-touch surface areas. Swabs were collected of participant nares, axilla, and groins to determine who in the household was colonized in addition to the patient. Now, I am going to tell the story through the results.

The preliminary key findings of the CAMP study were presented. Study data revealed 65% of study participants with the skin and soft tissue infection diagnosed microbiologically to be caused by Staph aureus are also colonized in one or more locations and 33% of the people they share their home with are colonized. A striking result was noted, 60% of the households are contaminated and have evidence of Staph aureus on the high-touch surfaces .

The big data case was presented next “ Obesity, Cardiometabolic Risk, and Adolescent Pregnancy: Building a De-Identified Electronic Clinical Database to Examine Biological and Social Determinants of Nutritional Status, Pregnancy, and Birth Outcomes”. The parent project of this study was described as a randomized clinical trial of 14 hospitals and Federally Qualified Health Centers (FQHCs) in New York City to usual care prenatal care (which is individual care) or group prenatal care, using a program called "Centering." The intervention group, (the group prenatal care) had significantly reduced the incidents of pre-term birth, low birth weight, and small for gestational age for these young mothers. Parent study results illustrated younger mothers in the intervention group gained less weight during pregnancy and returned to their pre-pregnancy weight quicker than women in the control condition. The parent study results piqued the investigators’ interest to design the current big data study with the research question to leverage the resources of the electronic health records

(EHR) to limit focus on to measures part of routine clinical care to reduce the burden of clinical research. The big study goal was to build a de-identified, multi-site EHR database to demonstrate this feasibility. The same population from the parent study, young women age 12-21 was investigated, abstracting their EHR to characterize the care and conditions that they experienced during the preconception, prenatal, postnatal, and early pediatric phases. The big study data component was 55,000 records from seven of the eight clinical sites.

The preliminary results of the cardiometabolic analysis were presented on blood pressure trends by obesity status. The presenter noted strikingly that each systolic and diastolic blood pressure measurement in the first visit statistically significantly monotonically increased moving from underweight through the obese. These data suggest that the obese women are just within the range of normotensive, noting that the incidence of hypertension will be faster and sooner among the obese and the overweight. The data were presented as a breakdown of underweight, normal weight, overweight, and obese, the definition of hypertension( HTN) was based on three measures from the chart on three different occasions. The prevalence of HTN was statistically significant among overweight women at 11%, 19% among obese women. The presented emphasized that this was two to four times higher than the prevalence of normal weight women. The data were referred to as an emergent disturbing picture of the cardiometabolic life course. The cholesterol, total cholesterol, triglycerides, blood glucose, and hemoglobin A1c, data trends were then presented and were all highly-statistically significant with a pattern virtually identical to blood pressure and hypertension.

The data presentation delved further with review of the incidence of low birth weight in the cohort of women, the focus of the intervention study. The trends revealed the incidence of low birth weight as inversely proportional to the weight of the mothers. It was clearly illustrated that birth weight was inversely associated with maternal weight and potentially related to a cycle of low birth weights, growth failure, low weight in teens, contributing to low weight in adults, and a repeated cycle over and over again. Tobin commented on the other end of obesity life cycle spectrum, where all of the large birth weight babies were born to the obese and overweight mothers. The cyclical nature of this relationship where maternal overweight and obesity contributes to child overweight and obesity, in part due to dietary patterns, feeding environment. The whole picture was presented holistically, demonstrated from the electronic health records, extracted from routine clinical care leading the investigators to raise the question of these multigenerational effects on the cardiometabolic curve. Tobin speculated that following these babies for 20 years or so; the same association in the grandchildren that are going to be born to these babies in the next two decades the cardiometabolic associations and cardiometabolic risks clearly illustrates a lifespan model of these multigenerational effects that indicating that clinician in this instance are not just treating individual patients. They are treating families and communities and generations. This is absolutely essential work for us to try to intervene and intervene using the most effective methods, and soon, in order to avoid the continuation of these disparities.

Tobin reflected back on some of the principles that gave rise to his projects. Critical elements were highlighted in the engagement of the clinicians and the patients in defining the measures and the study parameters and in really understanding what kinds of questions can be structured from these observations.

The key components in the above approach were the model of embedding mechanistic questions in outcome studies to ensure that there are variables and hypotheses that are related to each end of the translational spectrum.

Tobin summarized the critical issue as listening to each stakeholder's needs, priorities, their obligations, and understanding their rewards systems. A part of this is seen as eliciting and including the specific priorities for measurement and assessment to ensure that the outcomes are meaningful. The presenter stated the need to be able to define the outcomes across the full spectrum, starting with the biological, moving into the clinical and patient-centered, and then into health services measures and public health measures. He concluded that this approach allowed research to embed biological measures within well-characterized clinical populations.

#### Advancing the Science of Community Engaged Research 2017 Conference Learning Labs

*Maximizing Value of Stakeholder Engagement: Tips and Tools from Stakeholder Engagement Consulting on Nine PCORI-Funded Studies*

Presenters

**Gay Thomas** *(University of Wisconsin-Madison, WINRS)*

**Betty Kaiser** *(University of Wisconsin-Madison, WINRS)*

Learning Objectives

1. Identify orientation activities that prepare stakeholders to effectively participate in the project.
2. Recognize elements of a stakeholder meeting agenda that can yield constructive feedback for the research team.
3. Describe key strategies to sustain stakeholder engagement across the project lifespan.

*Mile High Community Engagement: Developing a Training Pipeline for Community Based Participatory Researchers in Colorado*

Presenters

**Victoria Francies** *(University of Colorado Denver, Colorado CTSI)*

**Mary Fisher** *(University of Colorado Denver, Colorado CTSI)*

**Montelle Tamez** *(University of Colorado Denver, Colorado CTSI)*

Learning Objectives

1. Describe how the pipeline within community engagement for researchers and community members can enhance CBPR research practice, and increase community participation and capacity.
2. Learn how to incorporate the roles of Community Research Liasions and create Immersion Programs for Community Engagement

*Helping Community Members Claim Their Power: Building Capacity to Partner with Research Institutions*

*Presenters*

**Yvonne Joosten** *(Vanderbilt University Medical Center, CERC)*

**Tiffany Israel** *(Vanderbilt University Medical Center, CERC)*

**Alexis Gorden** *(Sickle Cell Foundation of Tennessee)*

Learning Objectives

1. Identify potential roles for patients and other community stakeholders as active partners with research institutions.
2. Develop knowledge of essential elements for increasing community capacity to take on meaningful roles with research institutions.
3. Identify strategies to address institutional barriers to meaningful community engagement.

*Promotores (Community Health Workers) as Partners in Research: Lessons Learned and Recommendations*

Presenters

**Katrina Kubicek** *(University of Southern California)*

**Alma Garcia** *(University of Southern California)*

Learning Objective

Participants will identify ways in which Promotores (lay community workers) can form part of the research team.

*Strategies for Engaging the Community in Creating Patient-Centered Research Questions*

*Presenters*

**Shivonne Laird** *(Patient-Centered Outcomes Research Institute)*

**Courtney Clyatt** *(Patient-Centered Outcomes Research Institute)*

Learning Objectives

1. Understand what makes a good patient-centered research question.
2. Learn what type of information can be used to inform a patient-centered research question, and how anyone (including patient and community groups) can collect this information.
3. Learn how researchers can use data to make their research questions patient-centered or community-relevant.
4. Discuss ways patients and community members can engage with researchers, and vice versa, to ensure research questions are relevant.

*Engaging Diverse Communities to Understand How Precision Health Research Can Address Disparities*

Presenters

**Lisa Goldman-Rosas** *(Stanford University)*

**Rhonda McClinton-Brown** *(Stanford University)*

**Jill Evans** *(Stanford University)*

Learning Objectives

1. Discuss the barriers and facilitators of developing and implementing precision health research in diverse racial/ethnic communities.
2. Identify best practices for developing community-university partnerships for precision health research.
3. Understand how to develop and implement research to engage diverse communities in precision health research.
4. Identify best practices for working with researchers from diverse disciplines to incorporate community engagement in their research.
5. Become familiar with existing resources for increasing communities' capacity for engaging in precision health research.
6. Discuss diverse communities' understanding and perception of precision health research and related best practices for implementation of precision health research.

Implementing a Community / Patient Scientist Academy to Engage Underrepresented Populations in Research

Presenters

**Kate Stewart** *(Translational Research Institute, University of Arkansas for Medical Sciences)*

**Anna Davis** *(Translational Research Institute, University of Arkansas for Medical Sciences)*

Learning Objectives

1. List the two main objectives of the community / patient scientist academy.
2. Articulate at least three key concepts covered in the academy.
3. Describe at least two interactive exercises used to engage participants in the academy.

*Sharing Research Results with Those Who Need Them: Engaging with Community Partners to Plan Effective Disseminations*

Presenters

**Rachel Hemphill** *(Patient-Centered Outcomes Research Institute)*

**Lisa Stewart** *(Patient-Centered Outcomes Research Institute)*

**Vanessa Ramirez-Zohfeld** *(Northwestern University)*

Learning Objectives

1. Learn about a variety of methods for working with community partners to plan and prepare for effective dissemination of study results to end-users.
2. Generate ideas for a dissemination plan for a research case study.
3. Identify challenges for getting study results to end-users and share potential solutions and lessons learned.

*Best Practice Strategies for Engaging Community Stakeholders and Patients as Partners in Research*

Presenters

**Tilicia Mayo-Gamble** *(Georgia Southern University)*

**Velma McBride Murry** *(Vanderbilt University Medical Center, CERC)*

Overall Learning Objective

Participants will be able to identify effective strategies for engaging community stakeholders and patients as partners in research with an emphasis on expectations for challenges and strengths.

*The Forgotten Stakeholder: Partnering with University Administrators to Create Compensation and Recognition Mechanisms that Support Efficiency, Fairness and Sustainability in Community Engagement*

Presenters

**Lori Carter-Edwards** *(University of North Carolina-Chapel Hill)*

**Ginny Lewis** *(University of North Carolina-Chapel Hill)*

Learning Objectives

1. Define efficiency, fairness and sustainability in CEnR stakeholder engagement from the perspectives of:
  1. health providing/seeking communities
  2. academic researchers
  3. research grant administrators
  4. university administrators
2. Discuss categories of university mechanisms for recognition and compensation of non-employee stakeholders in health research, as well as other non-financial compensation benefits for stakeholders.
3. Develop strategic plans to build and strengthen efficiency, fairness and sustainability in stakeholder engagement initiatives that utilize one or more of the university mechanisms for recognition and compensation of non-employee stakeholders in health research.

*Development, Implementation and Evaluation of a Community Engaged Board: Best Practices for Strategies for Maximizing Success*

Presenters

**Alicia Matthews** *(University of Illinois at Chicago)*

**Amparo Castillo** *(University of Illinois at Chicago)*

**Emily Anderson** *(University of Illinois at Chicago)*

Learning Objectives

1. Describe the role of community engagement advisory boards in clinical and translational research.
2. Describe five contributions of community engaged advisory boards to improving research outcomes.
3. Discuss best practices in the formation and development of community engaged advisory boards.
4. Identify strategies for building skills and capacity among community engaged advisory board members.
5. Develop methods for evaluating the contributions of community engaged advisory boards to research teams.

#### Advancing the Science of Community Engaged Research 2017 Think Tank Discussions

**Community Health Needs Avenues to Assessments to Impactfully Serve Communities**

**Moderators**

**Karen Calhoun, MA,** City Connect Detroit, and Michigan Institute for Clinical and Health Research

**Lloyd Michener, MD** Department of Community and Family Medicine, Duke University Medical Center

**We Want You! Engaging Stakeholders in Early Translational Research**

**Moderators**

**Rhonda Kost, MD,** The Rockefeller University Center for Clinical and Translational Science

**Neely Williams, MDiv** Chief Executive Officer, Community Partners’ Network

**Mission not Impossible: Deploying CEnR to Achieve Health Equity**

**Moderators**

**Claudia Barajas,** Vanderbilt-Ingram Cancer Center

**Charles P. Mouton, MD, MS,** Department of Family Medicine, University of Texas Medical Branch

Is Money the Missing Link? Sustaining Community Partnerships

Moderators

**E. Hill De Loney, MSW** Community Based Organization Partners (CBOP)

**Louisa Stark, PhD,** Community Outreach and Collaboration Core, Center for Clinical and Translational Science, University of Utah School of Medicine

Digital Divide? Innovative Approaches to Disseminating CEnR Findings

Moderators

**Al Richmond, MSW,** Community Campus Partnerships for Health (CCPH)

**Jaye Bea Smalley, MPA,** Global I & I Patient Advocacy and Life Cycle Management

## Conclusion

During the past two decades, community and stakeholder engagement have emerged as essential approaches to accelerate the translation of research into practice and translational research programs are expected to ensure community engagement in all phases of research [16]. Many research programs have developed and implemented successful community engaged research programs, leading to a growing body of literature in this field. Effective community engaged research methods and best practices are not currently being distributed through research programs at a pace consistent with the demands. Researchers and stakeholders need a forum to rapidly disseminate community engaged research evidence and to gain practical knowledge for implementing new, and enhancing existing, research programs. The 2017 Advancing the Science of Community Engaged Research meeting, *Innovative and Effective Methods of Stakeholder Engagement in Translational Research* facilitated meaningful engagement of diverse stakeholder groups including racial and ethnic minorities, community and patient representatives, and junior investigators. Of 210 attendees, 72 completed the evaluation, and, of those, 36% self-affiliated as community members, and 21% as patient/caregiver advocacy, faith-based, or tribal organization members. Researchers and stakeholders need a forum to rapidly disseminate community engaged research evidence and to gain practical knowledge for implementing new, and enhancing existing, research programs. This conference 1) represented a step toward expanding the scientific basis for the community engaged research (CEnR) field by convening a diverse group of researchers, community partners, patient advocacy organizations, and other stakeholders to share innovative methods and strategies; 2) catalyzed innovative community engaged research by using presentation and discussion formats that facilitated interactive learning, collective problem solving, and new conceptual frameworks; and 3) enhanced the reach and impact of the scientific developments emerging from pioneering work in community engagement.

## Additional files

Additional file 1: 2017- The 2017 Advancing the Science of Community Engaged Research (CEnR) Innovative & Effective Methods of Stakeholder Engagement in Translational Research Conference Program. (PDF 8420 kb)

Additional file 2: Transcripts of full meeting reports, annotated by slide set numbers to corresponding presenters PowerPoint Presentations. (PDF 116000 kb)

## Abbreviations

BMI: Body Mass Index
CAMP: The Patient-Centered Comparative Effectiveness Study of Home-Based Interventions to Prevent Community-Associated MRSA Infection Recurrence
CBN: Center for Biomedicine and Nutrition
CCPH: Community Campus Partnerships for Health
CDC: Center for Disease Control
CEnR: Community Engaged Research
CEnR-NaV: Community Engaged Research Navigation Process
CME: Continuing Medical Education
COGIC: Church of God in Christ
CTSA: Clinical and Translational Science Awards
DOD: Department of Defense
EHR: Electronic Health Records
HTN: Hypertension
ICU: Intensive Care Unit
MUSC: Medical University of South Carolina
NCATS: National Center for Advancing Translational Sciences
NIH: National Institutes of Health
NIMHD: National Institute for Minority Health and Health Disparities
PHD: Pre-Health Dreamers
RCT: Randomized Control Study
TCC: Transdiscplinary Collaborative Center
UCSF: University of California, San Francisco
UIC: University of Illinois-Chicago
UIC-CCTS: University of Illinois-Chicago – Center for Clinical Translational Sciences
