# Supplementary material for: Proceedings of the 2017 Advancing the Science of Community Engaged Research (CEnR) Conference
Source: BMC Proc. 2019 Apr 19;13(Suppl 3):3. doi: 10.1186/s12919-019-0164-y (PMC6474049; doi:10.1186/s12919-019-0164-y)
Supplement: Supplementary file 1 — 2017- The 2017 Advancing the Science of Community Engaged Research (CEnR) Innovative & Effective Methods of Stakeholder Engagement in Translational Research Conference Program. (PDF 8420 kb) [file 12919_2019_164_MOESM1_ESM.pdf]

CE$_n$R

Advancing the Science of Community Engaged Research

INNOVATIVE & EFFECTIVE METHODS OF STAKEHOLDER ENGAGEMENT IN TRANSLATIONAL RESEARCH

September 14-15, 2017 • Washington, DC

Hosted by **Vanderbilt University Medical Center and Meharry Medical College**

Greetings Colleagues!

It gives us great pleasure to extend a warm welcome to the 2017 Advancing the Science of Community Engaged Research (CEnR) Conference!

Community engaged research fuels accelerated translation of clinical and bench discoveries into effectively implemented care practices that improve health. As you may know, successful, newly-developed community-engaged research methods are not advancing the field at a pace consistent with the demand for innovative and effective best practices and practitioners.

This conference provides a forum for cutting-edge approaches to meaningfully engage a broad range of stakeholders: including researchers, community members, patients, industry and governmental agencies, to share novel approaches, identify areas in need of further inquiry, and deliberate on key issues that will aid in the advancement of CEnR.

The Plenary Sessions are designed to provide critical perspectives and emerging evidence on the science of CEnR. The Learning Lab Sessions will provide "how to" opportunities for small-group learning on specific issues and practices, and the Think Tanks will advance discussions and problem-solving for issues that can impede or accelerate community engaged research.

This conference promises to be enlightening and thought-provoking as we continue to explore effective methods of engaging stakeholders in research and the dissemination of the scientific developments.

Special thanks to our conference organizing committee for their leadership and guidance in making this program a success, and the leadership of Vanderbilt University Medical Center and Meharry Medical College. Appreciation is also extended to the Association of American Medical Colleges for their outstanding facilities and hospitality during this meeting.

Best wishes,

Conference Co-Chairs

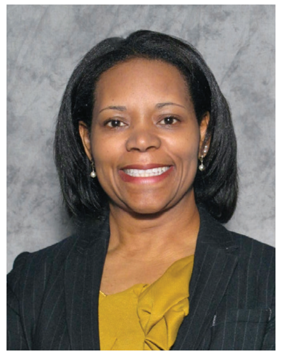

Consuelo H. Wilkins, MD, MSCI
Executive Director,
Meharry Vanderbilt Alliance

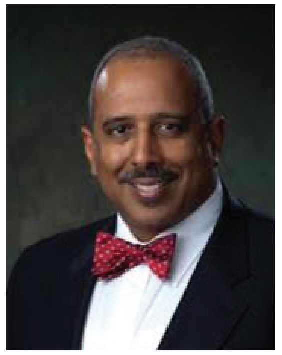

Charles P. Mouton, MD, MS
Vice Dean for Academic Affairs
Professor of Family Medicine
University of Texas Medical Branch

Organizing Committee

Consuelo H. Wilkins, MD, MSCI
Meharry-Vanderbilt Alliance
(Chair)

Charles P. Mouton, MD, MS
University of Texas Medical Branch
(Co-Chair)

Philip Alberti, PhD
Association of American Medical Colleges

Karen Calhoun, MA
City Connect Detroit and Michigan ICHR

Chinenye Anyanwu, PharmD, MPH
Patient-Centered Outcomes Research Institute

Rhonda G. Kost, MD
The Rockefeller University

Melvin Thompson, MBA
The Endeleo Institute

Lloyd Michener, MD
Duke University

Eruera "Ed" Napia, EdD
Urban Indian Center of Salt Lake

Maria Pardos de la Gándara, MD, PhD
The Rockefeller University

Tricia Piechowski-Whitney, MPH, MA
University of Michigan, MICHR

Al Richmond, MSW
Community Campus Partnerships for Health

Jaye Bea Smalley, MPA
Boehringer Ingelheim

Louisa Stark, PhD
University of Utah

Alvin "Hal" Strelnick, MD
Albert Einstein College of Medicine

Rev. Neely Williams, MDiv
Mid-South Clinical Data Research Network

Meryl Sufian, PhD
National Institute on Minority Health and Health Disparities

All events and times are subject to change. Please view conference website advancingcenrconference.com and mobile app for the latest schedule and details.

Welcome!

Welcome to the Association of American Medical Colleges (AAMC)!

Founded in 1876, the AAMC is a not-for-profit association dedicated to transforming health care through innovative medical education, cutting-edge patient care, and groundbreaking medical research. Its members comprise all 145 accredited U.S. and 17 accredited Canadian medical schools; nearly 400 major teaching hospitals and health systems, including 51 Department of Veterans Affairs medical centers; and more than 80 academic societies. Through these institutions and organizations, the AAMC serves the leaders of America's medical schools and teaching hospitals and their nearly 160,000 faculty members, 83,000 medical students, and 115,000 resident physicians.

To fulfill the AAMC's mission to "improve the health of all." authentic, meaningful partnerships between scientists and their community stakeholders are essential. The wisdom and expertise of local residents and patients are crucial to developing solutions to health and health care inequities and to improving the health and wellbeing of the communities the AAMC's members serve.

We are honored to host you as you come together this week to collaborate, share, and strive to improve the health of our nation.

Sincerely,

Ross McKinney, Jr., M.D.
Chief Scientific Officer, AAMC

Funding for this conference was made possible (in part) by 1 R13 TR 001694 - 01 A1 from the National Center for Advancing Translational Sciences (NCATS) and the National Institute on Minority Health and Health Disparities (NIMHD). The views expressed in written conference materials or publications and by speakers and moderators do not necessarily reflect the official policies of the Department of Health and Human Services; nor does mention by trade names, commercial practices, or organizations imply endorsement by the U.S. Government.

Vanderbilt®, Vanderbilt University Medical Center®, V Oak Leaf Design®, Monroe Carell Jr. Children's Hospital at Vanderbilt® and Vanderbilt Health® are trademarks of The Vanderbilt University

Schedule at a Glance

Thursday, September 14 • 7:30am - 6:00 pm

7:30 – 8:45 am	Registration/Breakfast
8:45 – 9:00 am	Welcome and Overview
9:00 – 10:15 am	Opening Plenary Session<br>*Innovative National and Statewide Community Engagement Initiatives to Improve Health*
10:15 – 10:30 am	Break
10:30 am – Noon	Learning Labs I
Noon – 1:00 pm	Networking Lunch
1:00 – 2:00 pm	Keynote Presentation<br>*Community Engagement in Minority Health Research to Reduce Disparities*
2:00 – 3:15 pm	Plenary Session II<br>*Precision Engagement: Approaches to Involving Diverse Populations in Precision Medicine*
3:15 – 3:30 pm	Break
3:30 – 4:30 pm	Think Tank Discussions
4:30 – 6:00 pm	Poster Session and Reception

Friday, September 15 • 7:30am - Noon

7:30 – 8:30 am	Registration/Breakfast
8:30 – 9:30 am	Plenary Session III<br>*Data as the Driver -- and Passenger -- for the Community Engaged Research Vehicle*
9:45 – 11:15 am	Learning Labs II
11:15 – 11:45 am	Closing Session<br>*Engaging Patients and Communities in Translational Science*
11:45 - noon	Grab & Go Box Lunch

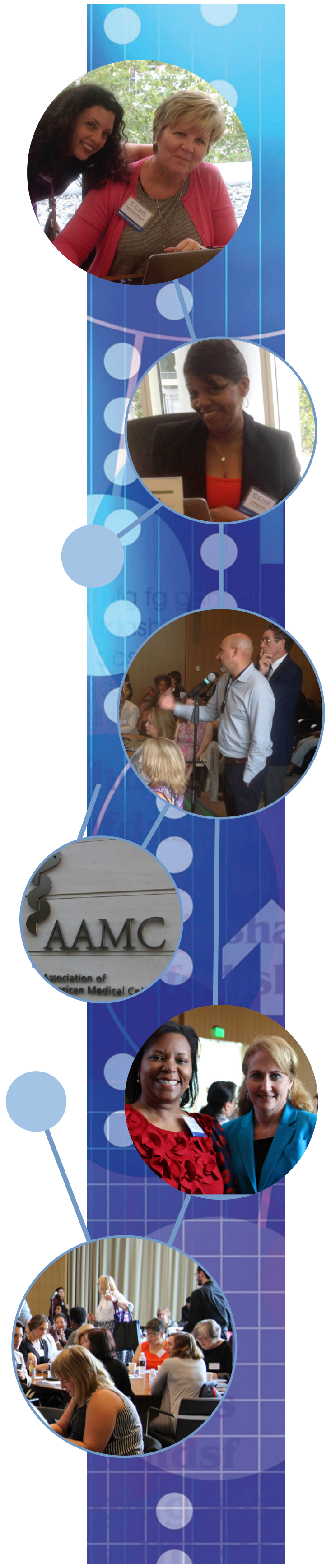

Thursday, September 14

7:30–8:45 am
Registration/Breakfast
2nd Floor Servery, Outside room LC-200

8:45–9:00 am
Welcome and Overview

LC-200

Consuelo H. Wilkins, MD, MSCI
Executive Director, Meharry-Vanderbilt Alliance, Associate Professor of Medicine, Vanderbilt University Medical Center and Meharry Medical College

Charles P. Mouton, MD, MS
Vice Dean for Academic Affairs, Professor of Family Medicine, University of Texas Medical Branch

Erurera "Ed" Napia, EdD
Program Manager for Sacred Paths Youth Services and Special Projects, Urban Indian Center of Salt Lake

9:00–10:15 am
Opening Plenary Session

Innovative National and Statewide Community Engagement Initiatives to Improve Health

Moderator: Sergio Aguilar-Gaxiola, MD, PhD
Director, University of California-Davis Center for Reducing Health Disparities

This opening plenary is a multi-stakeholder panel discussion with a researcher and community member presenting perspectives regarding key issues, opportunities and/or challenges regarding the science of community engaged research.

LC-200
Dismantling Structural Inequality through Partnerships

Al Richmond, MSW
Executive Director, Community Campus Partnerships for Health (CCPH)

LC-200
Advancing the Community Health Worker Workforce in Research Partnerships

Olveen Carrasquillo, MD, MPH
Chief, Division of General Internal Medicine, Professor of Medicine and Public Health Sciences, University of Miami
University of Miami CTSA Community Engagement and Cultural Diversity Program Director

10:15–10:30 am
Break
2nd Floor Servery, Outside room LC-200

10:30 am–noon
Learning Labs, Part I (Six Concurrent Sessions)

Learning Lab "how to" breakout sessions serve as forums for disseminating innovative methods in community engaged research. Learning Labs deliver practical information to guide implementation of community engaged research approaches and provide an interactive opportunity for researchers and other stakeholders to share best practices and lessons learned.

Thursday, September 14 (continued)

LC-200 *Maximizing Value of Stakeholder Engagement: Tips and Tools from Stakeholder Engagement Consulting on Nine PCORI-Funded Studies*

Gay R. Thomas (UW-Madison School of Nursing WINRS)

Betty Kaiser (UW-Madison School of Nursing WINRS)

LC-220 *Mile High Community Engagement: Developing a Training Pipeline for Community Based Participatory Researchers in Colorado*

Victoria Francies (University of Colorado Denver, Colorado CTSI)

Mary Fisher (University of Colorado Denver, Colorado CTSI)

Montelle Tamez (University of Colorado Denver, Colorado CTSI)

LC-230 *Helping Community Members Claim Their Power: Building Capacity to Partner with Research Institutions*

Yvonne Joosten (Vanderbilt University Medical Center, CERC)

Tiffany Israel (Vanderbilt University Medical Center, CERC)

Alexis Gorden (Sickle Cell Foundation)

LC-240 *Promotores (Community Health Workers) as Partners in Research: Lessons Learned and Recommendations*

Katrina Kubicek (University of Southern California)

Alma Garcia (Promotore)

LC-320 *Strategies for Engaging the Community in Creating Patient-Centered Research Questions*

Shivonne Laird (Patient-Centered Outcomes Research Institute)

Courtney Clyatt (Patient-Centered Outcomes Research Institute)

LC-330 *Engaging Diverse Communities to Understand How Precision Health Research Can Address Disparities*

Lisa Goldman–Rosas (Stanford University)

Rhonda McClinton-Brown (Stanford University)

Jill Evans (Stanford University)

Noon–1:00 pm **Networking Lunch**
2nd Floor, Servery Room, Outside LC-200

1:00-2:00 pm **Special Session: Community Engagement in Minority Health Research to Reduce Disparities**

Eliseo Pérez-Stable, MD
Director, National Institute for Minority Health and Health Disparities (NIMHD)

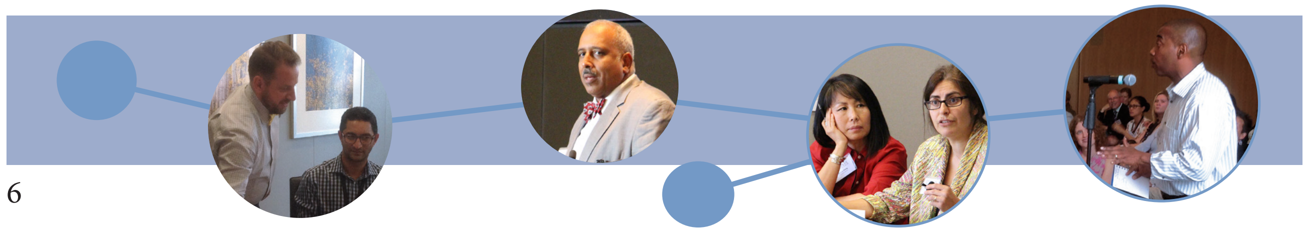

Thursday, September 14 (continued)

2:00–3:15 pm **Plenary Session II**

LC-200 **Precision Engagement: Approaches to Involving Diverse Populations in Precision Medicine**

Moderator: Lloyd Michener, MD
Duke University

This plenary session features speakers discussing progress in developing metrics, instruments and tools to measure the impact of community engaged research. The speakers will highlight gaps in metrics and provide perspectives on strategies needed to advance the science of the field.

LC-200 *From the Frontlines: What Does Precision Medicine Mean for Me?*

Usha Menon, PhD, RN, FAAN
Associate Dean for Research & Global Advances,
University of Arizona College of Nursing

LC-200 *Academic-Community Partnerships for Precision Medicine*

Chanita Hughes-Halbert, PhD
Associate Dean for Assessment, Evaluation and Quality Improvement,
Medical University of South Carolina

LC-200 *Advancing Trust to Engage Diverse Participation in Research: Community Dialogue and Partnerships*

Karriem S. Watson, DHSc, MS, MPH
Senior Research Scientist, University of Illinois at Chicago Cancer Center,
Director of Community Engagement and Implementation Science (OCERIS)

3:15–3:30 pm **Break**
2nd Floor Servery, by LC-200

3:30–4:30 pm **Think Tank Discussions (Five Concurrent Sessions)**

Think Tanks are focused on emerging community engaged research topics. These interactive group sessions are intended to stimulate dialogue, generate new perspectives and facilitate discussions that transcend disciplinary and academic/community boundaries.

LC-220 *Community Health Needs Avenues to Assessments to Impactfully Serve Communities*

Moderators:
Karen Calhoun, MA
Lloyd Michener, MD

LC-230 *We Want You! Engaging Stakeholders in Early Translational Research*

Moderators:
Rhonda Kost, MD
Neely Williams, MDiv

Thursday, September 14 (continued)

LC-240
Mission not Impossible: Deploying CEnR to Achieve Health Equity

Moderators:
Claudia Barajas
Charles Mouton, MD, MS

LC-320
Is Money the Missing Link? Sustaining Community Partnerships

Moderators:
E. Hill De Loney
Louisa Stark,PhD

LC-330
Digital Divide? Innovative Approaches to Disseminating CEnR Findings

Moderators:
Al Richmond, MSW
Jaye Bea Smalley, MPA

4:30–6:00 pm
Poster Sessions and Reception
2nd & 3rd Floor Foyer

The poster session provides researchers and stakeholders a forum to present cutting edge research and works-in-progress. Hosted by the Meharry-Vanderbilt Alliance and the Association of American Medical Colleges.

Friday, September 15

7:30–8:30 am
Registration/Breakfast
2nd Floor, Servery, outside LC-200

8:30–9:30 am
Plenary Session III

LC-200
Data as the Driver—and Passenger—for the Community Engaged Research Vehicle

Moderator: Melvin Thompson, MBA
Executive Director, The Endeleo Institute

LC-200
Big Data Research Ready Communities in Chicago
Regina Greer Smith, MPH, LFACHE
President, Healthcare Research Associates, LLC

Data as the Passenger: The Importance of Identifying What Data Matters to the Community
Courtney Clyatt, MA, MPH
Program Officer, Patient-Centered Outcomes Research Institute (PCORI)

Little Data, Big Data: Translational Research Partners Across the Spectrum
Jonathan N. Tobin, PhD
President/CEO, Clinical Directors Network, Inc.

Friday, September 15 (continued)

9:45–11:15 am
Learning Labs, Part II (Six Concurrent Sessions)

LC-200
*Implementing a Community / Patient Scientist Academy to Engage Underrepresented Populations in Research**

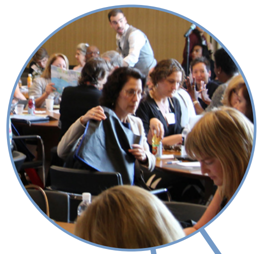

Kate Stewart (Translational Research Institute, University of Arkansas for Medical Sciences)

Anna Davis (Translational Research Institute, University of Arkansas for Medical Sciences)

* There is a maximum attendance of 25 people.

LC-220
Sharing Research Results with Those Who Need Them: Engaging with Community Partners to Plan Effective Disseminations

Rachel Hemphill (Patient-Centered Outcomes Research Institute)
Lisa Stewart (Patient-Centered Outcomes Research Institute)
Vanessa Ramirez-Zohfeld (Northwestern University)

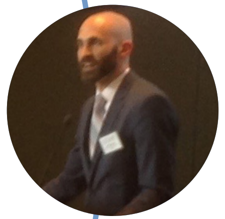

LC-230
Community Health Workers and Advocates as Stakeholders in Research: Mobilization and Engagement

Brendaly Rodriguez (University of Miami, CTSI)
Olveen Carrasquillo (University of Miami, CTSI)

LC-240
Best-Practice Strategies for Engaging Community Stakeholders and Patients as Partners in Research

Tilicia Mayo-Gamble (Georgia Southern University)
Velma McBride Murry (Vanderbilt University Medical Center, CERC)

LC-320
The Forgotten Stakeholder: Partnering with University Administrators to Create Compensation and Recognition Mechanisms that Support Efficiency, Fairness and Sustainability in Community Engagement

Lori Carter-Edwards (University of North Carolina-Chapel Hill)
Ginny Lewis (University of North Carolina-Chapel Hill)

LC-330
Development, Implementation and Evaluation of a Community Engaged Advisory Board: Best Practices for Strategies for Maximizing Success

Alicia Matthews (University of Illinois at Chicago)
Amparo Castillo (University of Illinois at Chicago)
Emily Anderson (University of Illinois at Chicago)

11:15–11:45 am
LC-200
Closing Session: Engaging Patients and Communities in Translational Science

Petra Kaufman, MD, MSc
Director, Division of Clinical Innovation,
National Center for Advancing Translational Science, NIH

Noon
Grab and Go Boxed Lunch
2nd Floor, Servery, Outside LC-200

Speaker Biographies

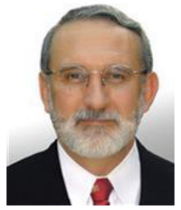

Sergio Aguilar-Gaxiola, MD, PhD
Aguilar-Gaxiola is an internationally renowned expert on mental health in ethnic populations. As on-site principal investigator of the Mexican American Prevalence and Services Survey – the largest mental health study conducted in the United States on Mexican Americans – he identified the most prevalent mental health disorders in the Mexican-origin population in California's central valley; showed that the rate of disorders increases the longer the individual resides in the United States; and demonstrated that children of immigrants have even greater rates of mental disorders.

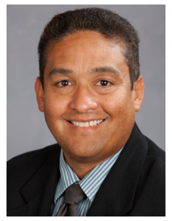

Olveen Carrasquillo, MD, MPH
Carrasquillo is Chief of the Division of General Internal Medicine and oversees a clinical, teaching and research enterprise of 40 full-time faculty, including three private clinical practices and an additional ambulatory hospital-based clinic at Jackson Memorial Hospital.

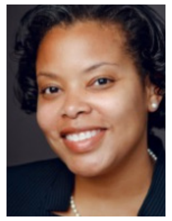

Courtney Clyatt, MA, MPH
Clyatt is a Program Officer for Engagement at the Patient-Centered Outcomes Research Institute (PCORI). She comes to PCORI with more than 10 years of experience in public health and project management.

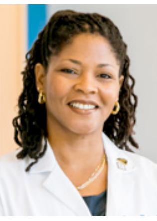

Chanita Hughes-Halbert, PhD
Hughes-Halbert research interests include sociocultural and psychological factors related to cancer prevention and control behaviors in African Americans, and also developing interventions to account for social and cultural context and building community partnerships through research.

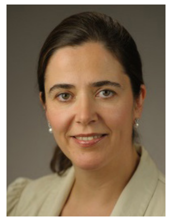

Petra Kaufmann, MD, MSc
Kaufmann is the director of both the Office of Rare Diseases Research and the Division of Clinical Innovation. Her work includes overseeing NCATS' Rare Diseases Clinical Research Network, Genetic and Rare Diseases Information Center, and Clinical and Translational Science Awards Program as well as the NIH/NCATS Global Rare Diseases Patient Registry Data Repository/GRDR® program.

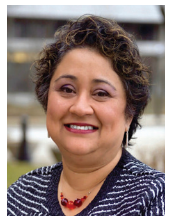

Usha Menon, PhD, RN, FAAN
Menon is Professor and Associate Dean for Research and Global Advances at the University of Arizona (UA) College of Nursing in Tuscon, AZ, and a member of the UA Cancer Center. At UA, she leads patient engagement for the recently awarded NIH Precision Medicine Initiative Cohort Enrollment Center grant.

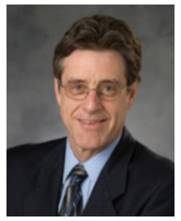

Lloyd Michener, MD
Michener is a Professor of Community and Family Medicine at Duke University School of Medicine. He founded the university's training programs in nutrition and prevention, helps coordinate the institutional chronic disease programs and is a founding member of the Masters in Clinical Leadership Program, a program created jointly by the Schools of Medicine, Nursing, Business, Law and the Institute of Public Policy.

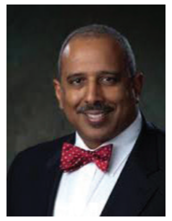

Charles P. Mouton, MD, MS
Mouton is a Professor of Family Medicine and the Vice Dean for Academic Affairs at the University of Texas Medical Branch. His research experience and interests are in health promotion and disease prevention in minority populations and the elderly, late-life domestic violence, health services research, particularly community engaged and practice-based research in underserved communities and quality end-of-life care.

Speaker Biographies

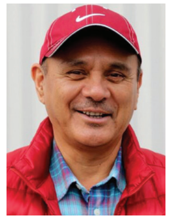

Eruera "Ed" Napia, EdD
Napia is the Program Manager for Sacred Paths Youth Services and Special Projects at the Urban Indian Center of Salt Lake. He was born into the Te Whiu and Te Popoto Hapu (sub-tribe) of the Ngapuhi Iwi (tribe) from the Tai Tokerau District of Aotearoa-New Zealand and currently lives in Salt Lake City, Utah. He was a clinical and adjunct faculty person at the University of Utah, where he taught multicultural education, bilingual education, Pacific Islander American Studies and American Indian Studies before joining the Urban Indian Center.

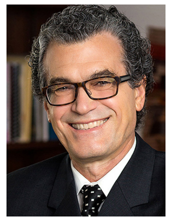

Eliseo J. Pérez-Stable, MD
Pérez-Stable is Director of the National Institutes of Health's National Institute on Minority Health and Health Disparities, which seeks to advance the science of minority health and health disparities research through research, training, research capacity development, public education and information dissemination.

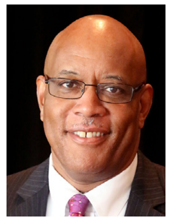

Al Richmond, MSW
Richmond has over 25 years of experience in a career that has uniquely blended social work and public health to address racial and ethnic health disparities. As a founding member and past chair of the Community Based Public Health Caucus and the National Community Based Organization Network – both affiliated with the American Public Health Association (APHA) – he helped to foster effective partnerships focused on community-identified health concerns and to increase the number of community leaders actively involved in APHA.

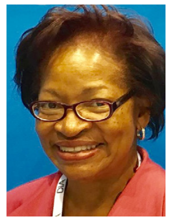

Regina Greer Smith, MPH, LFACHE
Smith is President of Healthcare Research Associates, LLC and a Founder/Board Member of Pastors4PCOR, a Steering Committee member of the CAPriCORN CDRN Chicago Area Patient-Centered Outcomes Research Network, Co-Chair of the Patient Partner Affinity Group – Asthma Evidence2Action and Patient-Governor of Arthritis Power/Creaky Joints PPRN.

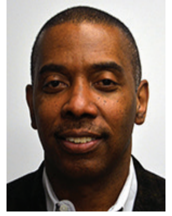

Melvin Thompson, MBA
Thompson is Executive Director of the Endeleo Institute, a public charity focused on creating a culture of health in the Washington Heights community in Chicago, IL, specifically along the West 95th Street Corridor.

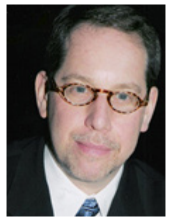

Jonathan N. Tobin, PhD
Tobin is President/CEO of Clinical Directors Network, Inc., a NYC-based practice-based research network dedicated to improving clinical outcomes for low income and medically underserved communities by creating community-academic partnerships around research, education/training and service. Tobin, a cardiovascular epidemiologist, is the Co-Director for Community-Engaged Research for the Center for Clinical and Translational Science and Adjunct Professor in the Allen and Frances Adler Laboratory of Blood and Vascular Biology at The Rockefeller University and also holds the rank of Professor in the Department of Epidemiology and Population Health at Albert Einstein College of Medicine of Yeshiva University/Montefiore Medical Center.

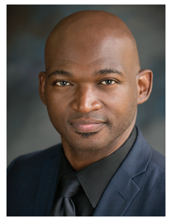

Karriem S. Watson, DHSc, MS, MPH
Watson is a Senior Research Scientist with the University of Illinois (UI) Cancer Center and the Director of Community Engaged Research for the UI Cancer Center at UI at Chicago and the Mile Square Health Center, a group of Federally Qualified Health Clinics affiliated with the UI Hospital and Health Sciences System.

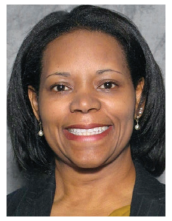

Consuelo H. Wilkins, MD, MSCI
Wilkins is the Executive Director of the Meharry-Vanderbilt Alliance, a strategic partnership between Meharry Medical College and Vanderbilt University Medical Center. She holds appointments as Associate Professor of Medicine at both institutions.

Poster Session Abstracts

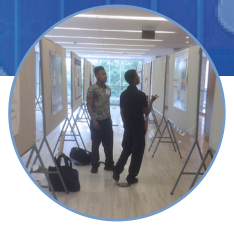

Overcoming Challenges of Community-University Research Partnerships: Exploring Opportunities for Training, Support and Program Development

Poster #1

Deborah Hendricks; Amy Shanafelt; Sheila Riggs; Kathleen Call; Milton Eder

Design-Based Community Building: Using Design to Engage People and Create Community

Poster #2

Helen Sanematsu; Sarah Wiehe

Evaluating the Impact of Stakeholder Academic Resource Panels

Poster #3

Grisel Robles-Schrader; Josefina Serrato; Michael Fagen

Health Care Providers' Perspectives on Engaging in a Clinical Data Research Network

Poster #4

Kim Unertl; Alecia Fair; Jacquelyn Favours; Rowena Dolor; Duane Smoot; Consuelo Wilkins

Building Bridges Between a Community and an Academic Medical Center via Community Tours

Poster #5

Megan Irby; Keena Moore; Mary Wigodsky; Twana Roebuck; Phillip Summers; Scott Rhodes

Engaging Youth Advocates in Community-Based Participatory Research: The Health of Youth Farmworkers in America

Poster #6

Andreina Malki; Taylor Arnold; Jackeline Leyva; Alejandra Monjarez

A Community-Academic Partnership to Understand the Correlates of Successful Aging in Place

Poster #7

Kimberly Vasquez; Dozene Guishard; William Dionne; Caroline Jiang; Cameron Coffran; Andrea Ronning; Glenis George-Alexander; Barry Coller; Jonathan Tobin; Rhonda Kost

Partnering with Teachers to Enhance Henrietta Lacks High School Symposium

Poster #8

Crystal Evans; Nancy Kass; Daniel Ford; David Lacks; Jim Potter; Barbara Bates-Hopkins; Cheryl Dennison Himmelfarb; Darcenia McDowell; Christine Weston

Community Day at the IRB: De-Mystifying the IRB and Enhancing Community Understanding of Human Subjects Projection in Research - Or, What Happens Behind the IRB Curtain?

Poster #9

Frederick Luthardt; Crystal Evans; Barbara Bates-Hopkins; Janet Johnson; Calvin Keen

Student-Led Community Engagement and Service Learning Projects in Underserved Communities

Poster #10

Ruby Thomas; Beverly Taylor; Desiree Rivers; Carla Durham-Walker

Inclusion and Integration of Community and Patient Perspectives in Review of Pilot Grant Applications

Poster #11

Patricia Piechowski; Adam Paberzs; Elizabeth LaPensee

Poster Session Abstracts

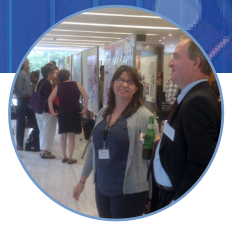

Achieving Health Equity through Health Department-Academic Partnerships & Community-Engaged Public Health Research: Healthy Chicago 2.0

Poster #12

Jen Brown; Nik Prachand; Lisa Masinter; Anne Posner; Sarah Rittner; Marc Atkins; Pankaja Desai; Doriane Miller

Health Outcomes that Matter: Engaging Patients, Community and Health System Stakeholders to Establish PCOR Priorities

Poster #13

Pamela Maxson; Nadine Barrett; Jennifer Gierisch; Ebony Boulware; Michelle Lyn

Mobile Screenings: Opening Doors to Improving Cardiovascular Health in High-Risk Communities

Poster #14

Valerie Morales Mitchell; Marlene Peters-Lawrence; Tiffany Powell-Riley

Progress in Stakeholder Engaged Research Forum: Best Practices and Key Lessons Learned

Poster #15

Han Hae-Ra; Ashley Xu; Kyra Waligora; Safiyyah Okoye; Melania Reese; Lee Bone; Cheryl Dennison-Himmelfard

Exploring African American Baby Boomers' Perceptions of Electronic Health Records: A Case Study

Poster #16

Alesha Ray

Measures of Trust and Willingness to Participate in Research

Poster #17

Victoria Villalta-Gil; Jennifer Erves; Alecia Fair; Jacquelyn Favours; Rowena Dolor; Duane Smoot; Consuelo Wilkins

Community Engagement through Coalition Building to Enhance Public Transportation and Promote Health

Poster #18

Phillip Summers; Elim Chao; Paula McCoy; James Perry; Scott Rhodes

Researcher Perspectives on Embedding Community Stakeholders in T1-T2 Research: A Potential New Model for Full-Spectrum Translational Research

Poster #19

Sheba George; Rachelle Bross; D'ann Morris; Norma Mtume; Keith Norris; Ibrahima Sankare; Teresa Seeman; Stefanie Vassar; Pluscedia Williams; Anna Lucas-Wright; Sonya Young Aadam; Arleen Brown

The Los Angeles County Health Profile

Poster #20

David Zingmond; E. Richard Brown; Gerald Krominski; Rachel Louie; Ying-Ying Meng; Melissa Pickett; Punam Parikh; Ami Shah; Peggy Toy; Sitaram Vangala; Stefanie Vassar; Steve Wallace; Arleen Brown

Multiplying Resources: Impact and Return on Investment of 10 Years of Community-Engaged Research (CEnR) and Partnership Seed Grants

Poster #21

Melvin Thompson; Rebecca Johnson; Ivonne Kang; Maryann Mason; Gina Curry; Jen Brown

Developing an Institute-Wide Community Advisory Board at the University of Michigan Institute for Clinical & Health Research

Poster #22

Karen Calhoun; Ayse Buyuktur; Zachary Rowe; Diane Carr; Maria Thomas; Ledon Chado; Sarah Bailey; Lisa Rentschler; Kevin Weatherwax; Tricia Piechowski; M. Spiroff; A. Paberz; J. Delva; T. Madiha

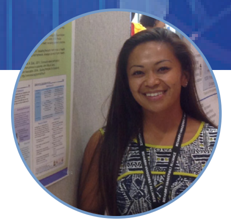

Poster Session Abstracts

Creating Strong Partnerships in the African American Community through Honest and Interactive Educational Events to SUPPORT and EMPOWER

Poster #23

Audrey Farrow; Deborah Burcombe; Garrett Davis; Timothy Hughes; Laura Baker

A Validated Scale to Measure the Person-Centeredness of Research Products

Poster #24

Mckenzie Houston; Ken Wallston; Victoria Villalta-Gil; Alan Richmond; Yolanda Vaughn; Sarah Stallings; Consuelo Wilkins

Application of Social Network Analysis for Evaluating and Improving Partnership Sustainability of Local and Statewide Community Health Coalitions in Indiana

Poster #25

Jennifer Mansfield; Lindley McDavid; Donna Vandergraff; Dennis Savaiano

Violence as a Health Disparity: Adolescents' Perceptions of Violence Depicted through Photovoice

Poster #26

Megan Irby; Lynn Rhoades; Nathan Ross Freeman; DeWanna Hamlin; Phillip Summers; Scott Rhodes; Stephanie Daniel

Development and Short-Term Impact of Community Research Training Curricula

Poster #27

Lexie Lipham; Jennifer Erves; Yvonne Joosten; Patrick Luther; Stephania Miller-Hughes

Community Scholars-in-Residence Program for Graduate Students and Postdoctoral Fellows: Hands-On Community-Engaged Research

Poster #28

Karen Glanz; Sarah Green; Jill McDonald; Alyssa Yackle

Engaging Latina Breast Cancer Survivors in Research: Building a Social Network Research Registry

Poster #29

Alejandra Hurtado de Mendoza; Adriana Serrano; Kristi Graves; Nicole Fernandez; Qi Zhu; Valeria Massarelli; Paola Rodriguez de Liebana; Claudia Campos; Florencia Gonzales; Laura Logie; Vanessa Sheppard

A Community-Engaged Approach to Measuring Trust in Biomedical Research

Poster #30

Nicollette Davis; Jacquelyn Favours; Sarah Stallings; Consuelo Wilkins

Creating a Strategic Alliance with Diverse Partners to Address Health Disparities through Innovative Precision Health Research

Poster #31

Jill Evans; Rhonda McClinton-Brown; Van Ta Park; Ysabel Duron; Jan Vasquez; Owen Garrick

Assessing Organizational Capacity Needs to Promote Partnership Readiness for Community-Engaged Research

Poster #32

Hilary Broughton; Mei-Hsi Chiang; Sarah Bobmeyer; Angela Brown

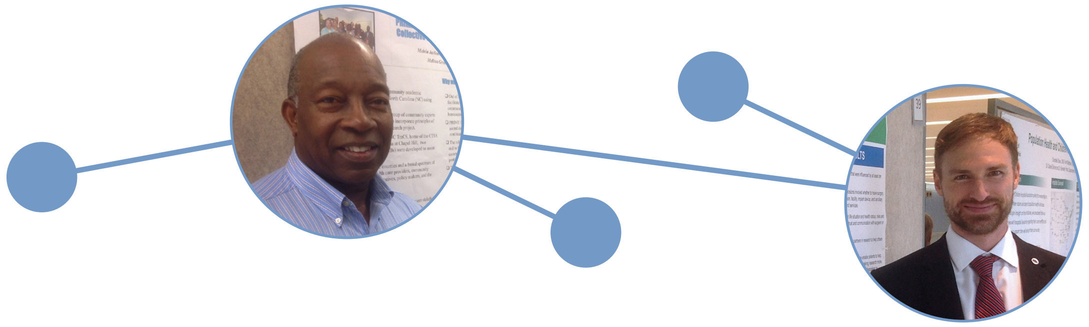

Poster Session Abstracts

PCORnet Obesity Observational Study: Short and Long-Term Effects of Antibiotics on Childhood Growth (Gathering Stakeholder Feedback to Improve Engagement Process)

Poster #33

Andrea Goodman; Tony Solomonides; Ivette Torres; Jordan Capizola; Kathleen Murphy; Juliane Reynolds; Jason Block; Sharon Terry; Doug Lunsford

Are Social Determinants of Trust Determined by Our Choice of Trust Measurements?

Poster #34

Jennifer Erves; Victoria Villalta-Gil; Alecia Fair; Jacquelyn Favours; Rowena Dolor; Duane Smoot; Consuelo Wilkins

A Tailored Educational Program to Improve Cancer Clinical Trial Participation Among African Americans and Latinos

Poster #35

Jennifer Erves; Claudia Barajas; Tilicia Mayo-Gamble; Caree McAfee; Pamela Hull; Maureen Sanderson; Juan Canedo; Katina Beard; Consuelo Wilkins

A Community Organization Assessment for Identifying Barriers to Data Dissemination for Community-Based Participatory Research (CBPR) Findings in a Faith-Based Community: The Washington, DC Cardiovascular Health and Needs Assessment

Poster #36

JaWanna Henry; Dana Sampson; Tiffany Powell-Wiley

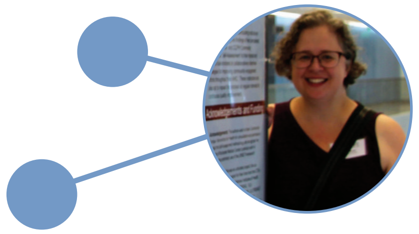

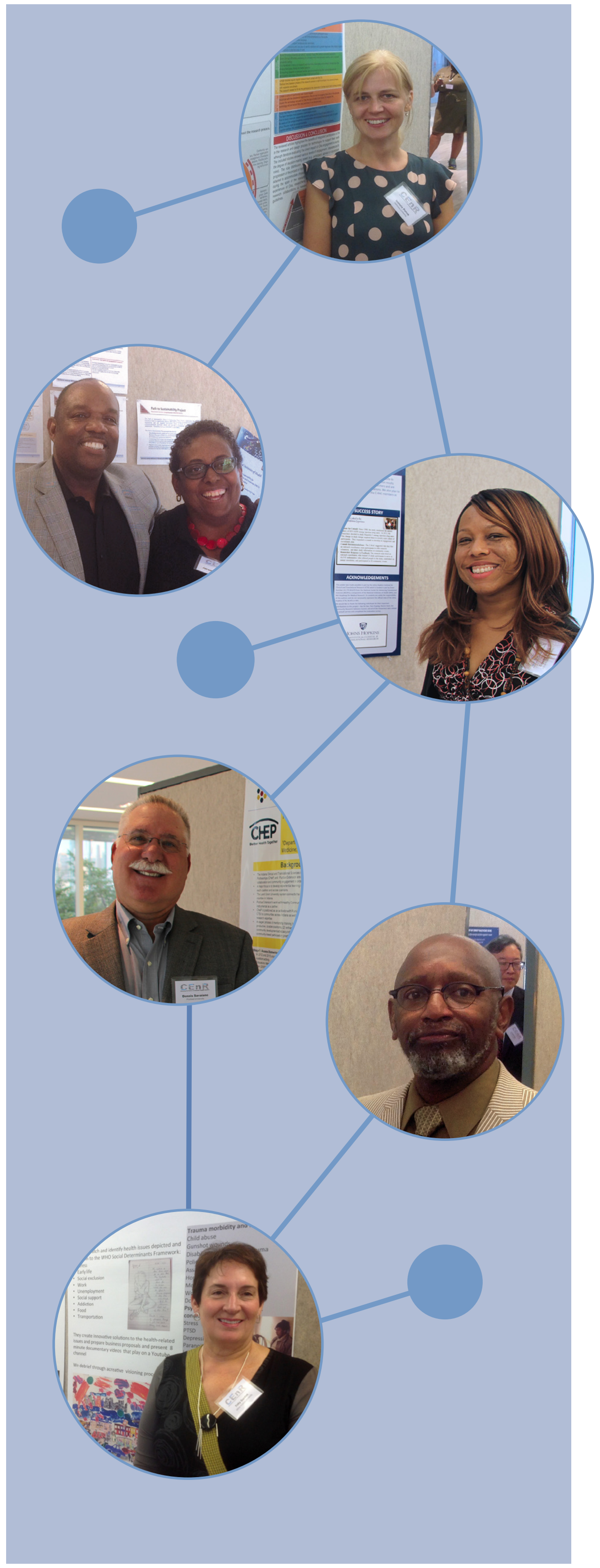

Learning Labs I

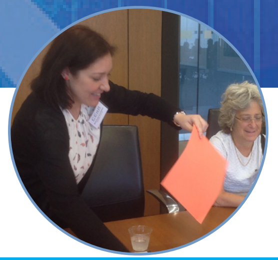

Maximizing Value of Stakeholder Engagement: Tips and Tools from Stakeholder Engagement Consulting on Nine PCORI-Funded Studies

Presenters:

Gay Thomas
University of Wisconsin-Madison, WINRS

Betty Kaiser
University of Wisconsin-Madison, WINRS

Learning Objectives:
1) Identify orientation activities that prepare stakeholders to effectively participate in the project.
2) Recognize elements of a stakeholder meeting agenda that can yield constructive feedback for the research team.
3) Describe key strategies to sustain stakeholder engagement across the project lifespan.

Mile High Community Engagement: Developing a Training Pipeline for Community Based Participatory Researchers in Colorado

Presenters:

Victoria Francies
University of Colorado Denver, Colorado CTSI

Mary Fisher
University of Colorado Denver, Colorado CTSI

Montelle Tamez
University of Colorado Denver, Colorado CTSI

Learning Objectives:
1) Describe how the pipeline within community engagement for reseachers and community members can enhance CBPR research practice, and increase community participation and capacity.
2) Learn how to incorporate the roles of Community Research Liasions and create Immersion Programs for Community Engagement.

Helping Community Members Claim Their Power: Building Capacity to Partner with Research Institutions

Presenters:

Yvonne Joosten
Vanderbilt University Medical Center, CERC

Tiffany Israel
Vanderbilt University Medical Center, CERC

Alexis Gorden
Sickle Cell Foundation

Learning Objectives:
1) Identify potential roles for patients and other community stakeholders as active partners with research institutions.
2) Develop knowledge of essential elements for increasing community capacity to take on meaningful roles with research institutions.
3) Identify strategies to address institutional barriers to meaningful community engagement.

Promotores (Community Health Workers) as Partners in Research: Lessons Learned and Recommendations

Presenters:

Katrina Kubicek
University of Southern California

Alma Garcia
Promotore

Learning Objective:
Participants will identify ways in which Promotores (lay community workers) can form part of the research team

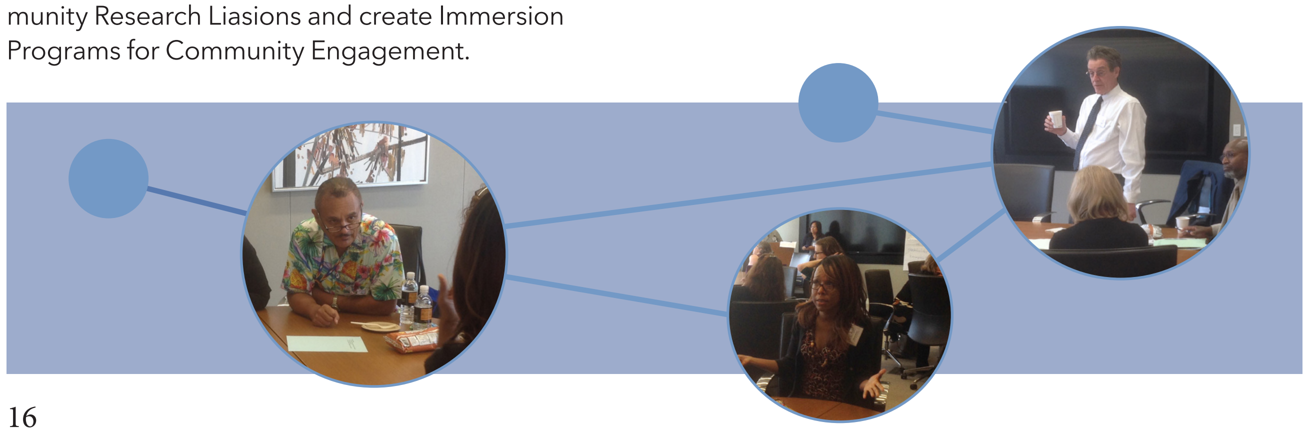

Learning Labs I (continued)

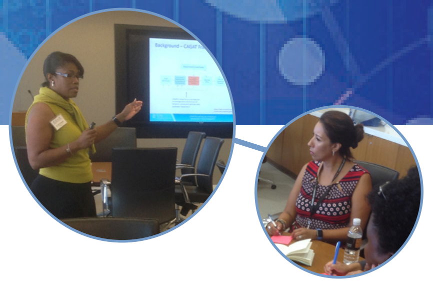

Strategies for Engaging the Community in Creating Patient-Centered Research Questions

Presenters:

Shivonne Laird
Patient-Centered Outcomes Research Institute

Courtney Clyatt
Patient-Centered Outcomes Research Institute

Learning Objectives:
1) Understand what makes a good patient-centered research question.
2) Learn what type of information can be used to inform a patient-centered research question, and how anyone (including patient and community groups) can collect this information.
3) Learn how researchers can use data to make their research questions patient-centered or community-relevant.
4) Discuss ways patients and community members can engage with researchers, and vice versa, to ensure research questions are relevant.

Engaging Diverse Communities to Understand How Precision Health Research Can Address Disparities

Presenters:

Lisa Goldman-Rosas
Stanford University

Rhonda McClinton-Brown
Stanford University

Jill Evans
Stanford University

Learning Objectives:
1) Discuss the barriers and facilitators of developing and implementing precision health research in diverse racial/ethnic communities.
2) Identify best practices for developing community-university partnerships for precision health research.
3) Understand how to develop and implement research to engage diverse communities in precision health research.
4) Identify best practices for working with researchers from diverse disciplines to incorporate community engagement in their research.
5) Become familiar with existing resources for increasing communities' capacity for engaging in precision health research.
6) Discuss diverse communities' understanding and perception of precision health research and related best practices for implementation of precision health research.

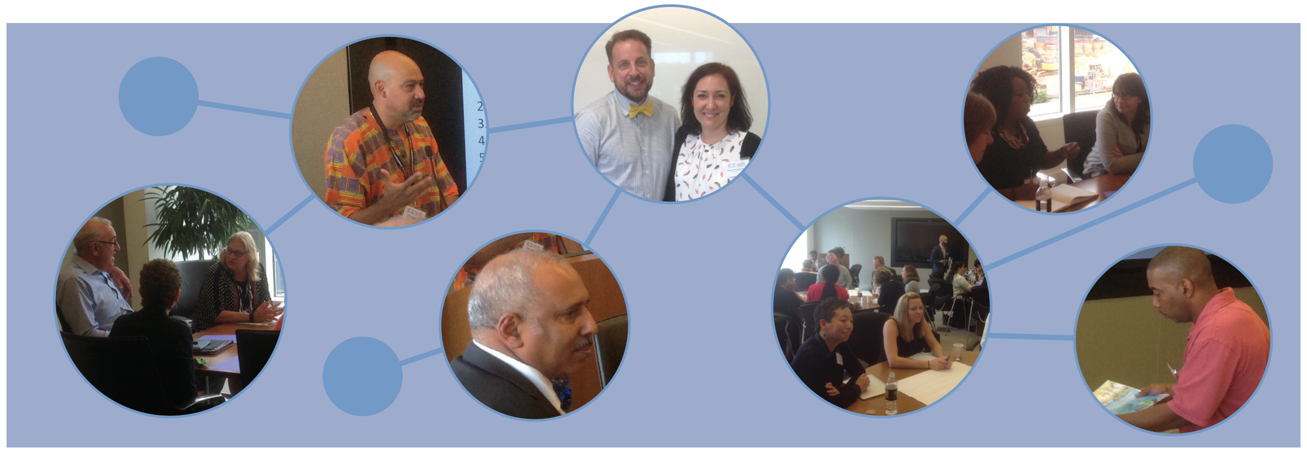

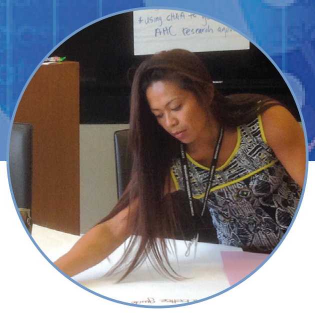

Learning Labs II

Implementing a Community / Patient Scientist Academy to Engage Underrepresented Populations in Research

Presenters:

Kate Stewart
Translational Research Institute
University of Arkansas for Medical Sciences

Anna Davis
Translational Research Institute
University of Arkansas for Medical Sciences

Learning Objectives:
1) List the two main objectives of the community/patient scientist academy.
2) Articulate at least three key concepts covered in the academy.
3) Describe at least two interactive exercises used to engage participants in the academy.

Sharing Research Results with Those Who Need Them: Engaging with Community Partners to Plan Effective Disseminations

Presenters:

Rachel Hemphill
Patient-Centered Outcomes Research Institute

Lisa Stewart
Patient-Centered Outcomes Research Institute

Vanessa Ramirez-Zohfeld
Northwestern University

Learning Objectives:
1) Learn about a variety of methods for working with community partners to plan and prepare for effective dissemination of study results to end-users.
2) Generate ideas for a dissemination plan for a research case study
3) Identify challenges for getting study results to end-users and share potential solutions and lessons learned.

Community Health Workers and Advocates as Stakeholders in Research: Mobilization and Engagement

Presenters:

Brendaly Rodriquez
University of Miami, Miller School of Medicine
Miami CTSI

Olveen Carrasquillo
University of Miami, Miller School of Medicine
Miami CTSI

Learning Objectives:
1) Articulate a process of identifying non-traditional stakeholders in research in your community, based on an example provided on engaging community health workers (CHWs) in Patient Centered Outcomes Research (PCOR) training in Florida.
2) Develop a plan for raising awareness and engaging a particular segment or underrepresented population in research.

Best Practice Strategies for Engaging Community Stakeholders and Patients as Partners in Research

Presenters:

Tilicia Mayo-Gamble
Georgia Southern University

Velma McBride Murry
Vanderbilt University Medical Center, CERC

Overall Learning Objective:
Participants will be able to identify effective strategies for engaging community stakeholders and patients as partners in research with an emphasis on expectations for challenges and strengths.

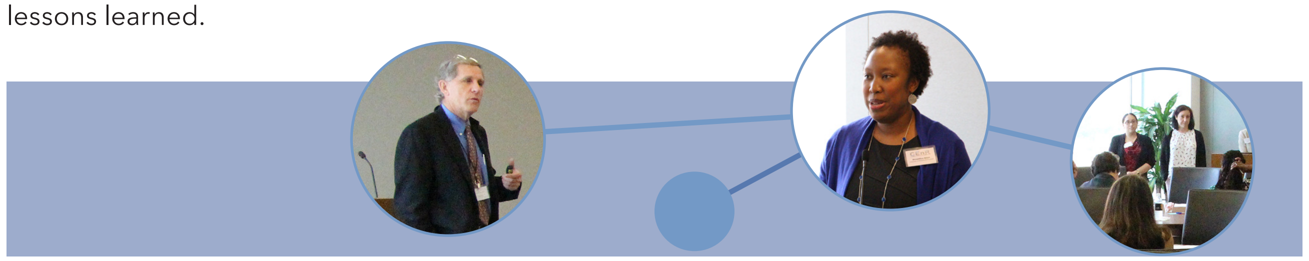

Learning Labs II (continued)

The Forgotten Stakeholder: Partnering with University Administrators to Create Compensation and Recognition Mechanisms that Support Efficiency, Fairness and Sustainability in Community Engagement

Presenters:

Lori Carter- Edwards
University of North Carolina-Chapel Hill

Ginny Lewis
University of North Carolina-Chapel Hill

Learning Objectives:
1) Define efficiency, fairness and sustainability in CEnR stakeholder engagement from the perspectives of: a) health providing/seeking communities; b) academic researchers; c) research grant administrators; and c) university administrators.
2) Discuss categories of university mechanisms for recognition and compensation of non-employee stakeholders in health research, as well as other non-financial compensation benefits for stakeholders.
3) Develop strategic plans to build and strengthen efficiency, fairness and sustainability in stakeholder engagement initiatives that utilize one or more of the university mechanisms for recognition and compensation of non-employee stakeholders in health research.

Development, Implementation and Evaluation of a Community Engaged Board: Best Practices for Strategies for Maximizing Success

Presenters:

Alicia Matthews
University of Illinois at Chicago

Amparo Castillo
University of Illinois at Chicago

Emily Anderson
University of Illinois at Chicago

Learning Objectives:
1) Describe the role of community engagement advisory boards in clinical and translational research.
2) Describe five contributions of community engaged advisory boards to improving research outcomes.
3) Discuss best practices in the formation and development of community engaged advisory boards.
4) Identify strategies for building skills and capacity among community engaged advisory board members.
5) Develop methods for evaluating the contributions of community engaged advisory boards to research teams.

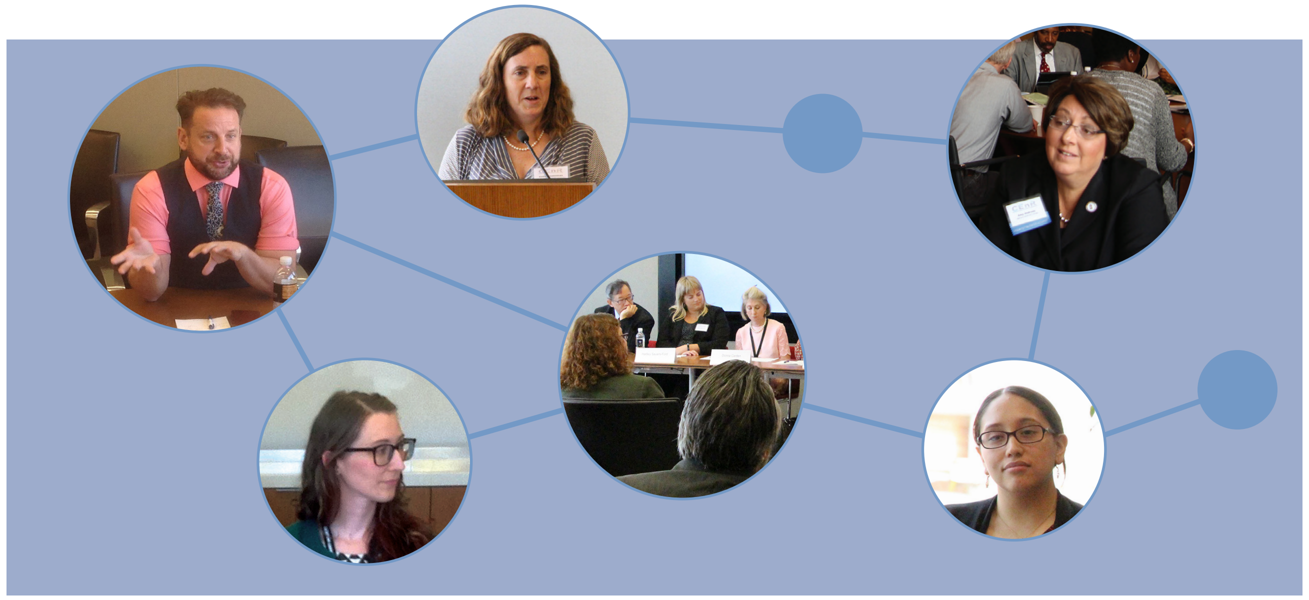

Conference Information

Co-Sponsors

Vanderbilt University Medical Center

Vanderbilt University Medical Center is home to Vanderbilt University Hospital, The Monroe Carell Jr. Children's Hospital at Vanderbilt, the Vanderbilt Psychiatric Hospital, and the Vanderbilt Stallworth Rehabilitation Hospital. These hospitals experienced more than 63,000 inpatient admissions during fiscal year 2015. Vanderbilt's adult and pediatric clinics treated more than 2.2 million patients during this same period. Vanderbilt University Hospital and the Monroe Carell Jr. Children's Hospital at Vanderbilt were recognized again this year by U.S. News & World Report's Best Hospitals as among the nation's best with 18 nationally ranked specialties.

Meharry Medical College

Meharry Medical College is one of the nation's oldest and largest historically black academic health science centers dedicated to educating physicians, dentists, researchers and health policy experts. It includes a medical school, dental school and a graduate school; and is home to the Robert Wood Johnson Center for Health Policy at Meharry. The college is also a leading producer of African Americans with PhDs in biomedical sciences. In addition to providing quality professional health care education, exemplary patient care, and compassionate community outreach, Meharry Medical College produces the *Journal of Health Care* for the Poor and Underserved, a public health journal.

Meharry-Vanderbilt Alliance

Meharry-Vanderbilt Alliance, founded in 1999, bridges the institutions of Meharry Medical College and Vanderbilt University Medical Center. Its mission is to enrich learning and advance clinical research in three primary areas–community engagement, interprofessional education and research–by developing and supporting mutually beneficial partnerships between Meharry Medical College, Vanderbilt University Medical Center and the communities they serve. Through community engagement, the Alliance serves a large community of stakeholders including surrounding universities and colleges, community organizations, faith-based outlets and community health centers. Its interprofessional education enhances students' interdisciplinary understanding and improves patient outcomes through integrated care. The research conducted provides access to experienced grant writers and materials supporting the grant application process and facilitates grant-writing workshops.

Poster sessions and reception sponsored by the Association of American Medical Colleges

AAMC
Tomorrow's Doctors, Tomorrow's Cures®

Founded in 1876 and based in Washington, D.C., the Association of American Medical Colleges is a not-for-profit association dedicated to transforming health care through innovative medical education, cutting-edge patient care, and groundbreaking medical research. Its members comprise all 147 accredited U.S. and 17 accredited Canadian medical schools; nearly 400 major teaching hospitals and health systems, including 51 Department of Veterans Affairs medical centers; and more than 80 academic societies. Through these institutions and organizations, the AAMC serves the leaders of America's medical schools and teaching hospitals and their nearly 167,000 full-time faculty members, 88,000 medical students, and 124,000 resident physicians. Additional information about the AAMC and its member medical schools and teaching hospitals is available at www.aamc.org.

CME Credits

Vanderbilt University School of Medicine designates this live activity for a maximum of 9.5 AMA PRA Category 1 Credit(s)™. Physicians should claim only the credit commensurate with the extent of their participation in the activity.

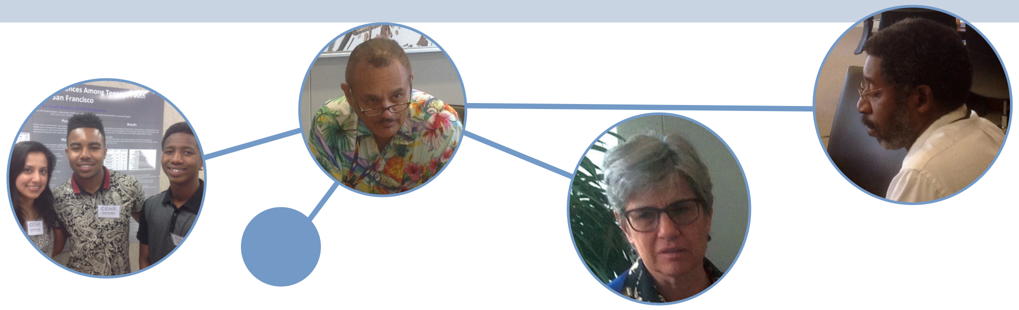

Conference Information

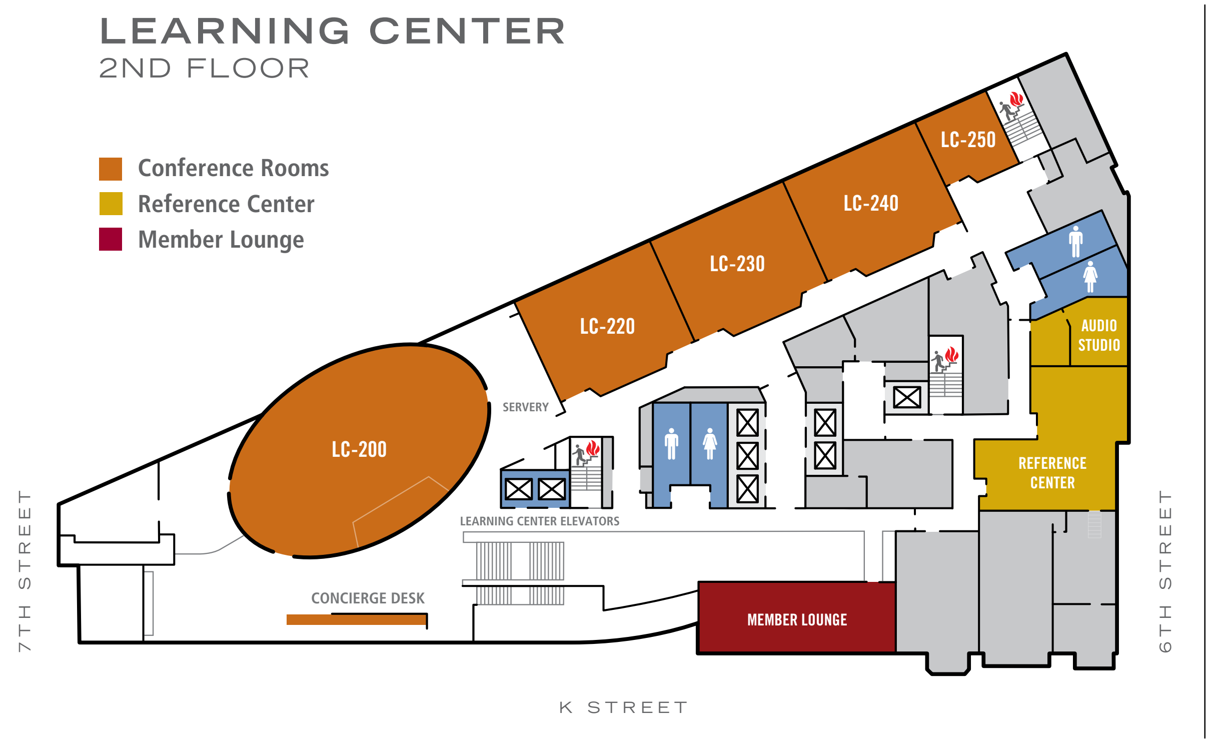

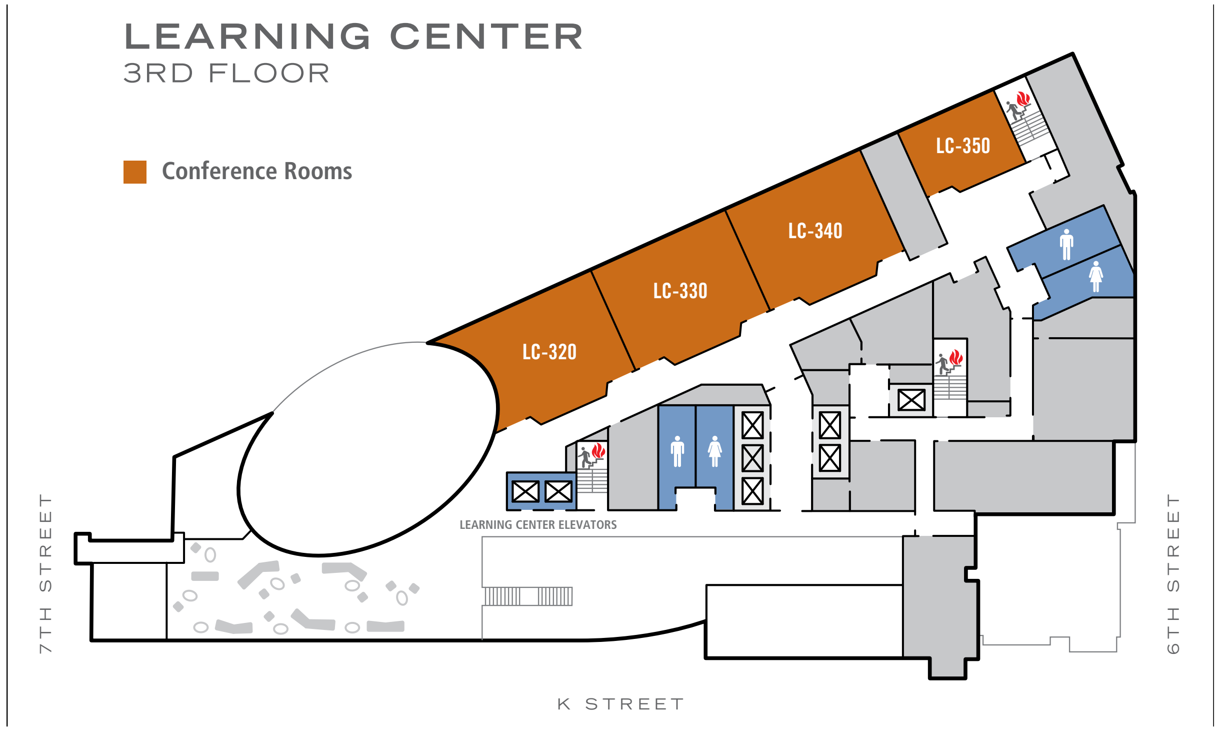

ADA Compliance

Building 655K was designed and constructed in compliance with the current Americans With Disabilities Act (ADA) requirements.

The Accessibility Codes are:
28 CFR Part 36, ADA Standards
Title 12A DCMR Construction Code
Supplement, 2008 Edition

Breastfeeding Rooms

A room for breastfeeding mothers is located on the third floor, near LC-350.

Downloading the CEnR App

Get the App

1. Go to the right store. Access the App Store on iOS devices and the Play Store on Android.

If you're using a Blackberry or Windows phone, skip these steps. You'll need to use the web version of the app found here: https://events.crowdcompass.com/center

2. Install the app. Search for *Advancing CEnR Conference*. Once you find the app, tap either **Download or Install**. After installing, a new icon will appear on the touchscreen.

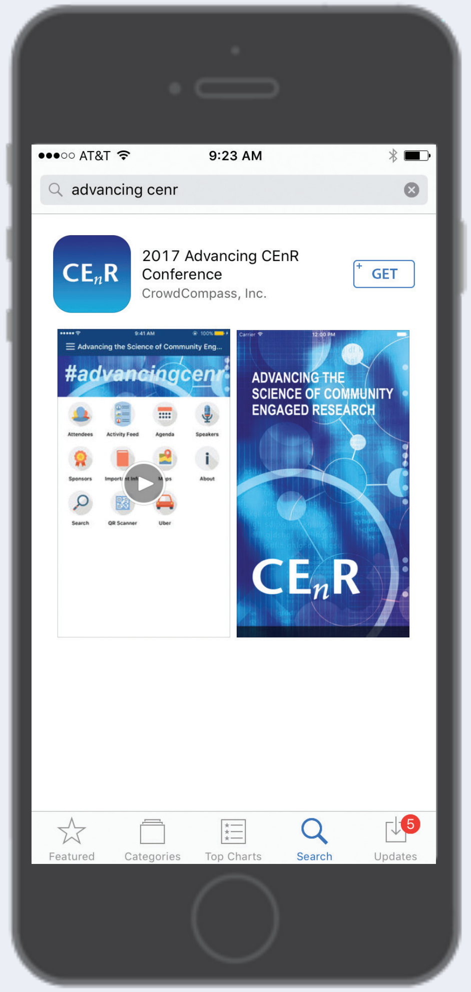

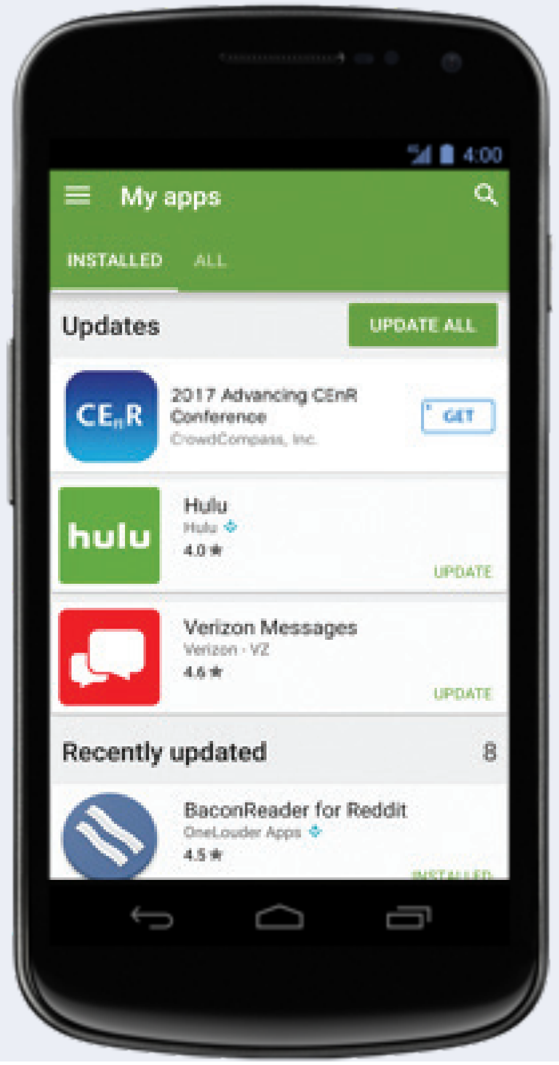

Check for updates

1. Go to the right store. Access the App Store on iOS devices and the Play Store on Android.

2. Check for updates. If you have an iOS device, tap the **Updates** tab and check the list to see if any updates are available for the *Advancing CEnR Conference* app.

For an Android device, tap the three-lined icon in the top left corner, then **My apps**. If *Advancing CEnR Conference* is listed under "Updates, tap it's name, then Update.

Notes

Acknowledgements
Special thanks to the Advancing CEnR Organizing Team, Vanderbilt University Continuing Medical Education and the Meharry-Vanderbilt Community Engaged Research Core

MEHARRY
VANDERBILT
ALLIANCE

SINCE 1999

Biomedical Sciences Building
1005 Dr. D.B. Todd Jr. Boulevard
Nashville, TN 37208

(615) 963-2820
meharry-vanderbilt.org

CONSUELO H. WILKINS, MD, MSCI
Executive Director
consuelo.h.wilkins@meharry-vanderbilt.org

@mvalliance

Facebook.com/mvalliance

@mvalliance
