# Supplementary material for: Proceedings of the 2017 Advancing the Science of Community Engaged Research (CEnR) Conference
Source: BMC Proc. 2019 Apr 19;13(Suppl 3):3. doi: 10.1186/s12919-019-0164-y (PMC6474049; doi:10.1186/s12919-019-0164-y)
Supplement: Supplementary file 2 — Transcripts of full meeting reports, annotated by slide set numbers to corresponding presenters PowerPoint Presentations. (PDF 116000 kb) [file 12919_2019_164_MOESM2_ESM.pdf]

Dismantling Structural Inequality through Partnerships

Al Richmond

ccph.executivedirector@gmail.com

Executive Director, Community-Campus Partnerships for Health

Raleigh, North Carolina

Abstract

Mr. Richmond presents an overview of Community Campus Partnerships for Health (CCPH) which celebrated its 20 Anniversary in 2017. CCPH is an organization founded on and committed to service learning, community-based participatory research, and partnerships. Mr. Richmond further shares that, through partnerships, CCPH is moving toward seeing, naming, and dismantling systems of structural inequality.

[*Al Richmond, MSW*]: Good morning. I think we are still trying to find our voice, and I am working on mine - trying to find it as well. It is such an amazing pleasure for me to be here to join you for what now has become an annual meeting. It really is an intimate space. We are very close, and it is really great to see so many of my friends from all over the country. I want you to join me, first of all, in acknowledging the hard work of Consuelo Wilkins and the Planning Committee. I feel that Consuelo is an amazing friend, colleague, confidante, just a trailblazer in the space of community engagement, and I feel so very fortunate to call her friend. I also want to acknowledge the Meharry-Vanderbilt Alliance team. I think it would be remiss for me to not recognize also that in the midst of planning this meeting that team experienced the tremendous loss of a former staff member who was killed recently, and that team has really stepped up to the plate. I also want to acknowledge Jeannine Skinner and the work and contribution that Jeannine made to the team. Jeannine had been a part of that team and had relocated to Charlotte to be a Professor at the University of North Carolina in Charlotte. The contributions she made to that team were very important and really, in many ways, she was on a trajectory forward to accomplish significant things in North Carolina and beyond.

This meeting, gathering here, is special for many reasons. In part, because we were very fortunate to be able to provide travel scholarships to many community leaders, and quite frankly, this is something that we have been working on for many years and really trying to push that forward. So, I am excited that we have at least 20 individuals here who really represent the community. I talk about the fact that I am the "C" in "Community Campus Partnerships" and the "C" in "Community Engagement," so it is a pleasure to have other colleagues join me. I think I'll have an opportunity to have lunch with you today. I would encourage you, if this is your first time being in a setting like this, to really speak up and speak out because we really do need to hear your voice. Your voice is extremely important. I would take Consuelo up on the offer that if you have a question or

concern or comment, that you step to the mic and push everyone else aside and speak your voice, right? So, do that. This is a special setting and I think it's a setting that will honor that. I want to start by talking a little bit about Community Campus Partnerships for Health. I never assume that everybody knows who we are.

I am going to start by giving you an overview of Community Campus Partnerships for Health and sometimes I will say the full name and sometimes I will just say CCPH. [SLIDE 2] Our organization is grounded in the scholarship of service learning, community-based participatory research, and the role of partnerships. Originally, these were primarily individuals in allied health professions, including physicians, nurses, dentists, and others who desired to take their academic skills and really work in communities to improve the health of those communities. These were graduate/post-graduate/postdoctoral students, who, along with our Founding Executive Director, Sarena Seifer, felt the need to create this organization out of that spirit of advancing community-engaged research, but also out of the service aspect of that work. 2017 is a special year for us. It's our 20th anniversary as a leadership organization. While we didn't invent partnerships, we have spent 20 years seeking to more fully understand and promote partnerships as a platform for social justice and health equity. In the months leading up to our 20th anniversary, we spent time as a Board and staff in a period of reflection and visioning. We had our 14th international conference last year in New Orleans, and some of you were in attendance at that amazing meeting, as I describe it, "The Best Conference Ever!" It was next to yours, Consuelo, certainly not to take anything away from your meeting, but it was an amazing meeting. It was at that time that I really, as a part of that process, really worked with the Board to catalyze and kind of describe who we are and what we do as an international organization.

[SLIDE 3] Out of that process of really visioning and thinking about ourselves as an organization, these five pillars emerged: 1) Leading; 2) Convening; 3) Partnering; 4) Training; and 5) Disseminating. In some ways, it synthesizes or catalyzes who we are as an organization. We are <u>leading</u> for social justice. We are <u>convening</u> for change. We are <u>partnering</u> for collective impact. We are <u>training</u> to enhance learning and best practices. And we are <u>disseminating</u> for impact. You will note that all of these are action words. These are foundational ways in which we carry out our mission as an organization to advance social justice and health equity. They shape our present work as well as chart a course for the future as an organization. Our core values and beliefs are listed here. [Slide 4] I won't go through all of this in terms of a continuum, but I want to highlight a couple of things. First, we view health very broadly. We believe that we should change the conditions of the environment in which people live, work, study, pray, and play. It's that second pillar there

that I'll spend some time talking about today in terms of dismantling structural inequalities. We believe in a central role that communities play in their own well-being, we believe in the power of partnerships. We are committed to social justice and strive to model equity and justice, even in our own organization. That last piece is really important and that means that we have to walk the walk and not just talk it.

All of the work of CCPH is carried out through partnerships. [SLIDE 5] As a community leader, I have had over 20 years of experience of working locally in Raleigh, North Carolina, and then in North Carolina regionally, and then became involved in the CTSA. Everything I have done has really been about partnerships. If I have accomplished any degree of success or any measure of moving the needle forward, it has been because of partnerships. I strongly believe in the power of partnerships. The work of CCPH is fully vested in partnerships. Some are long-term partnerships, with you as academic partners and community partners, and others are emerging. I recently was thinking about the fact that I should put a notice on our website that says, "Wanted: Partners." We are always interested in partnering with people and connecting with them and I see many of our partners here today. For us, it is really about living out the principles of partnership, living out who we are as an organization.

It is through this lens of partnerships that I want to share with you how I scan the world and how I see the world and this country. It's just how I see things. This is a word cloud and we took our core principles, so what you see here is all these words and really "partnership" stands out. More than anything else, we talk about the power of partnerships. [SLIDE 6] So, for me, as a leader, the images of torches and racially-charged propaganda that occurred on or near the campus of the University of Virginia on August 12th and 13th, images of Nazi, Germany, the fire-bombing that occurred at the 16th Street Baptist Church in Alabama ... these images also remind me of conversations that I had with my grandmother as a child who talked about the night rides that occurred in her community in South Carolina, as the members of the Ku Klux Klan rode through black neighborhoods with torches burning crosses. These images on the campus of UVA serve to highlight a deep divide in our nation, as we struggle with our own history of racism and antisemitism. In no way does the U.S. have a monopoly on these issues, as they are global in impact and unfortunately, they are ever-present. While we have passed laws and legislation, the structural inequalities remain. [SLIDE 7] Equally disturbing is that many individuals hold on to ideas of white supremacy and a racial hierarchy in this country and around the world. The images of August 12th and 13th were shocking, horrific, and deeply disturbing. The fact that they occurred in or close to a university campus should not be lost. I want to

highlight two reasons why I think that is important. First of all, I believe that in this country at this time there is a battle for the hearts and minds of young people around issues of white supremacy and a racial hierarchy. Secondly, I believe that we should understand that academic institutions are viewed as purveyors of thought and intellectual pursuit. The mission of the University of Virginia that was modified and adopted in 2014 reads that "The University of Virginia is a public institution of higher learning, guided by a founding vision of discovery, innovation, and development of the full potential of talented students from all walks of life [*emphasis on ALL WALKS OF LIFE*]. Today, I am joined by two of the CCPH Board members, Karriem Watson and Jen Brown, and our Board collectively stands today in outrage at the ideas of white supremacy and racial hierarchy.

What are we doing about this? It is one thing to be outraged. It is one thing to voice your concerns as an individual or collectively about these concerns. But what are we doing? I want to share with you about what CCPH is doing to challenge the status quo and challenge these ideas. I offer the work that we are doing as a gift to work in partnership with you to actually dismantle systems of structural inequality in this country. [SLIDE 8] First of all, in 2016, as Camara Jones assumed leadership of the American Public Health Association, CCPH joined with her and other public health leaders, academic leaders, community-based organizations, and faith-based organizations in launching a presidential campaign against racism. At the time, I'm sure people thought, "Wow, Camara, you are going to dismantle this system in one year?" It was an ambitious plan. It was important for her to take that on as a platform because we, in this country, have a problem, not with race, but we have a problem with racism. Not race. But racism. And the fact that Dr. Jones used her position and the platform that was the American Public Health Association to make this not just an issue for her but a public health issue ... she elevated this from being a personal issue of outrage and concern to really saying that racism impacts the health of people in this nation, and THAT is important. Therefore, when Camara came to me and said, "Al, will you support this," I said, "Absolutely, yes," because I believe it is important to do. I believe it is important because, again, it elevates the issue of racism in this nation.

On this particular slide you will see that Camara talks about racism as a system of structure and opportunity and assigning value based on the social interpretation of how one looks. What we call "race" that unfairly disadvantages some individuals and communities unfairly advantages other individuals and communities and saps the strength of the whole society through the waste of human resources. If you are not familiar with Camara and her work, I would certainly encourage you to look at [*her paper, "Levels of Racism: A Theoretical*

Framework and] a Gardener's Tale as being the primer when we look at dismantling structural inequality and understanding racism in our nation.

What else are we doing as an organization? [SLIDE 9] This summer, with the support of funding from the University of North Carolina, Robert Wood Johnson-funded Clinical Scholars Program, CCPH created the convening "Structural inequalities: An On the Ground View" in Durham, which was an amazing gathering of over 50 leaders from across the country who came together, some from academic settings, community-based organizations, and faith-based organizations, who came together for two days of learning to deepen their understanding of structural inequality in this nation. Everything about that experience was intentional. The fact that we wanted to make sure that people understood structural inequality in a way that for some of them had never experienced it before like that. First of all, it was held on the campus of a historically black college in North Carolina, and that was significant. For many people that live outside of the deep south, you have no familiarity with historically black colleges and universities. One of the things that is so interesting is that these colleges and universities were not established simply because black people wanted to go to their own school, but they were established because they had no other choice. That is important in the current dialogue in this nation. It is not about school choice. It is not about school choice. It is about, at one time in this nation, this nation had a system that did not allow and permit black people or other races of people to attend colleges and universities with white people. That's the truth. It's just that simple. Out of necessity, blacks and others established historically black colleges and universities, and that is a truth that we need to advance. It is a truth that we need to speak out on and be clear on. We held it at North Carolina Central University. We also spent much of our time on the campus of historic Stagville, which is a plantation in North Carolina. It is located in Durham. It is located in the city of medicine, kind of the county of medicine. Again, that shouldn't be lost on you as well. We intentionally went there because we wanted to expose people to what the genesis of structural inequality looks like. We took people down the road into these deep woods and to this plantation that at one time covered over 60,000 acres with thousands of enslaved people. We wanted people to understand that when left unchecked, this is what structural inequality looks like. These were real people living out their lives. These were not people who were working for pay. These were people that were enslaved. One of the things in doing my own work, and I tried to embrace this fully and understand it, but when I talk about people who worked on a plantation, I don't refer to them as slaves, because to me, that dehumanizes them. I now refer to them as enslaved people. I believe that when we use different labels and all of that, that it actually minimizes them and dehumanizes them as people. It was our intent to make sure

we understood who they were, and that is important to me as well. In launching this, we took a major step forward and did more than simply talk about inequalities as an abstract concept. The design of intensive was intentional and important.

I want to talk briefly about the definition of structural racism. [SLIDE 7] It is a system in which public policies, institutional practices, cultural representations, and other norms work in various, often reinforcing ways, to perpetrate racial group inequity. For us, this is how we examine and look at structural racism. I want to take just a minute here to also acknowledge the history of social activism in this country among college students. [SLIDE 10] These are images from North Carolina of students sitting in Woolworth's in Greensboro. I want us to think about the fact that, as we go forward and move forward in community engagement, the work that I am talking about, that we need to be involved in, is not new. This is old work. This is work that is fully grounded and working in the Civil Rights Movement. [SLIDE 11] This is an image of PHD's, pre-health dreamers, individuals who were fighting for their right to remain here in the United States. [SLIDE 12] These are images of students and communities working together at Standing Rock, standing together around social justice and health equity. I don't want us to shy away from the historic role, and the responsibility we have because of where we sit and balancing between research and communities and understanding the real issues that are impacting communities.

As I wrap this up, I want to think about and share with you our principles of partnership. [SLIDE 13] In the three years I have been with CCPH, I have really been unpacking this this idea of "Principles of Partnership," and I want to highlight one aspect of the "Principles of Partnership," and that is "Transformative Experiences." [SLIDE 14] I love this more than anything else because I believe that we have to be engaged in creating transformative experiences for the people that are engaged in our work interpersonally, institutionally, and then as a community. If our work is not transforming communities, then what are we doing? We are guilty of really supporting structures and systems of inequity and everything that people say about us then becomes true. If we don't want that to be the case, we have to be engaged in creating transformative experiences. Those of you that are community leaders will be transformed by this experience, I guarantee you. I started out in North Carolina. I could not have envisioned or imagined that one day I would be leading CCPH. I could not have imagined it. For me, this whole idea of transformative experiences is important. I want you to leave this space and this place believing that you are on a trajectory toward leadership and that you are going to transform your community. As academics and communities working

together, we can transform our communities. We can make them better. We can leave them better off than where we found them. The work of CCPH, the structural inequality training that we offered this summer, will be offered next year, and I hope that you will join us on this journey toward really better understanding how communities and campuses can work together to dismantle structural inequalities that exist, not just around race, but in particular, racism and other issues.

Finally, I believe that your work is important. [SLIDE 15] CCPH currently has a call for nominations to recognize partnerships - our 2017 RWJF Award for Health Equity. We present this award annually. The nominations are due Monday, and I have hand copies with me. I want you to apply for this award and particularly if you are doing work around dismantling structural inequalities. But for all of you, please consider applying for this, and join us in our work toward dismantling structural inequality and really living out our mission to achieve social justice and health equity in this nation. Thank you.

Declarations

- The presenter has no financial competing interests related to this presentation.
- All authors have approved the manuscript for submission
- The content of this presentation and transcript has not been published or submitted for publication elsewhere.
- This presentation was organized by the Meharry-Vanderbilt Alliance with support from NCATS and the NIMHD of the National Institutes of Health under award number R13TR001694

Advancing the Science of Community Engaged Research

DISMANTLING STRUCTURAL INEQUALITY

20 YEARS
Community-Campus Partnerships for Health
Promoting Health Equity and Social Justice

About Us

Community-Campus Partnerships for Health
Promoting Health Equity and Social Justice

The mission of Community-Campus Partnerships for Health is to promote health equity and social justice through partnerships between communities and academic institutions.

- Established in 1997
- 501c3 nonprofit membership organization
- International leader in community-campus partnerships

5 Pillars define our core work…

Community-Campus Partnerships for Health
Promoting Health Equity and Social Justice

Leading

Convening

Partnering

Training

Disseminating

CCPH upholds the following beliefs and values:

1. We view **health broadly** as physical, mental, emotional, social and spiritual well-being.

2. We emphasize partnership approaches to health that focus on **changing the conditions and environments** in which people live, work, study, pray and play.

3. We believe in the fundamental need for healthier communities and **the central role that communities play in their own well-being**.

4. We believe creating healthier communities and overcoming complex societal concerns **requires collaborative solutions that bring communities and institutions together as equal partners** and build upon their assets, strengths and capacities.

5. We believe in **the power of partnerships** to transform communities and institutions.

6. We are **committed to social justice and strive to model equity and justice** in how we approach and carry out our work.

20 YEARS

Community-Campus Partnerships for Health

Promoting Health Equity and Social Justice

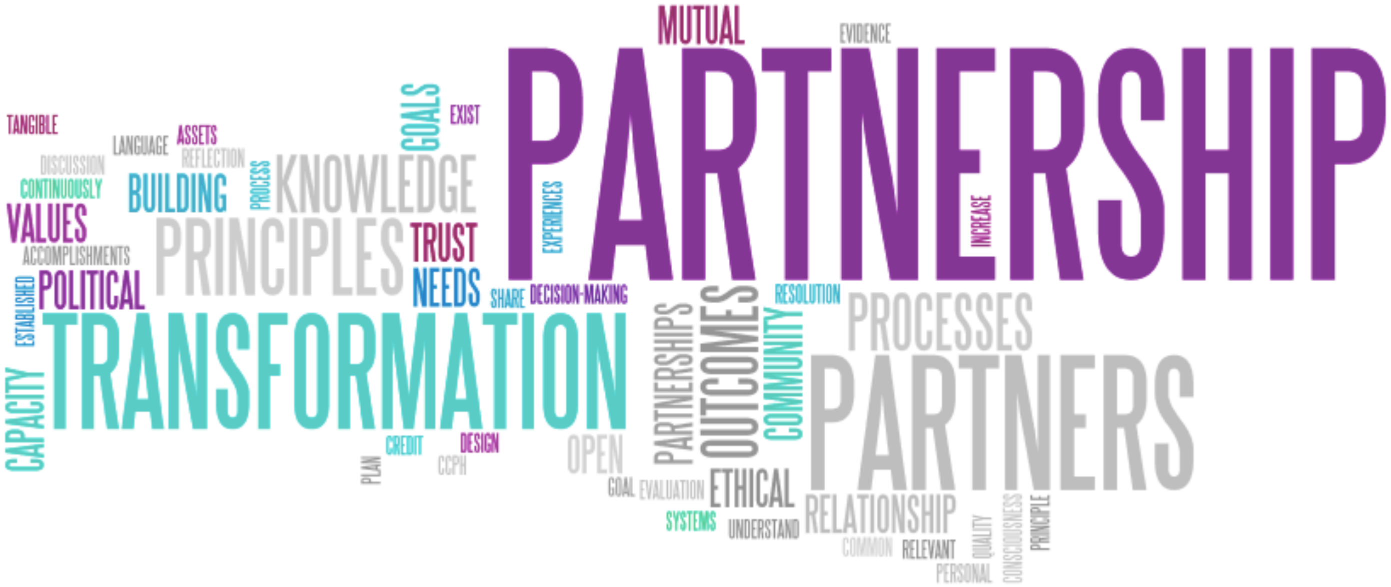

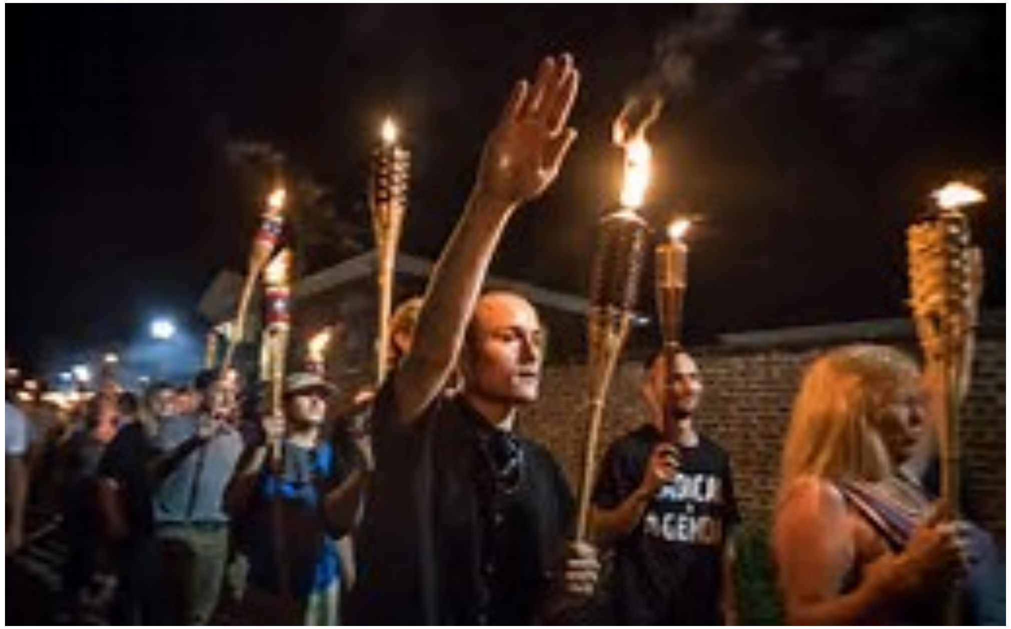

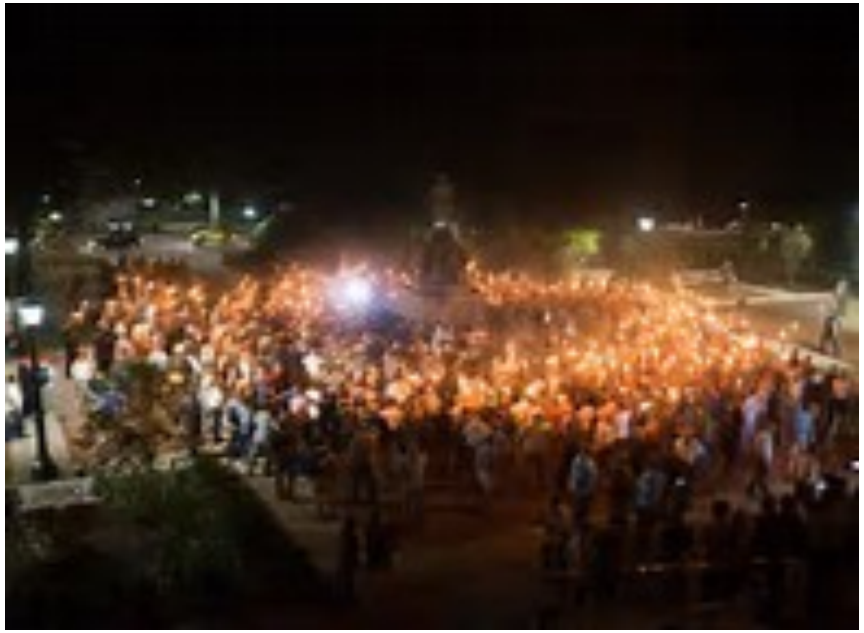

20 YEARS

Community-Campus Partnerships for Health

Promoting Health Equity and Social Justice

Defining Structural Racism

…a system in which public policies, institutional practices, cultural representations and other norms work in various, often reinforcing ways to perpetuate racial group inequity.

Community-Campus Partnerships for Health
Promoting Health Equity and Social Justice

https://assets.aspeninstitute.org/content/uploads/files/content/docs/rcc/RCC-Structural-Racism-Glossary.pdf

A National Campaign Against Racism

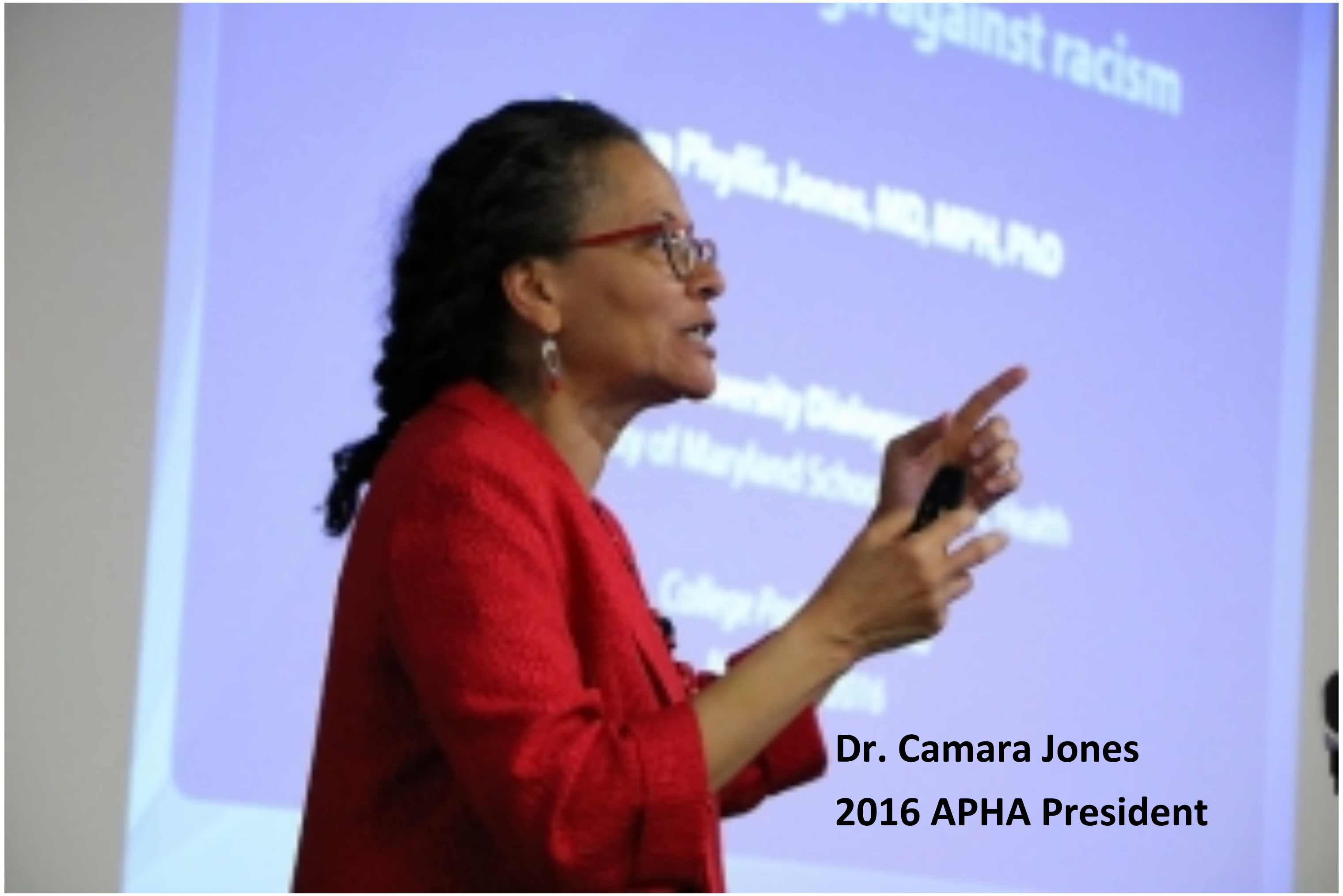

Dr. Camara Jones

2016 APHA President

Racism as a system of structuring opportunity and assigning value based on the social interpretation of how one looks — which is what we call "race" — that unfairly disadvantages some individuals and communities, unfairly advantages other individuals and communities and saps the strength of the whole society through the waste of human resources.

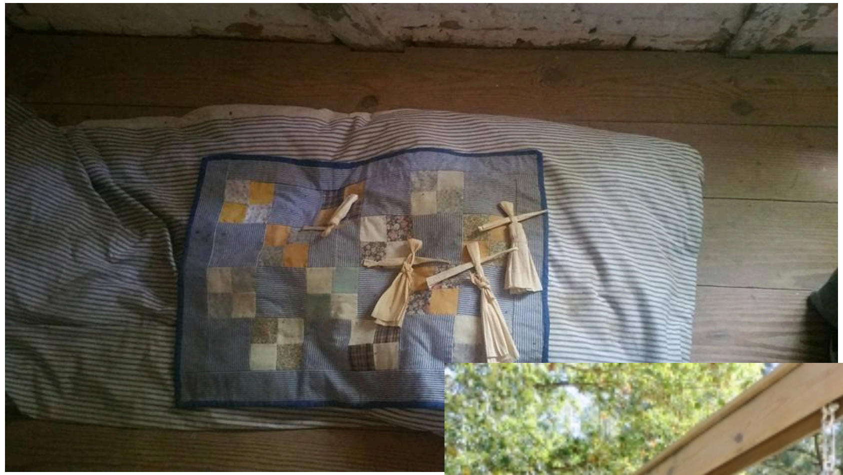

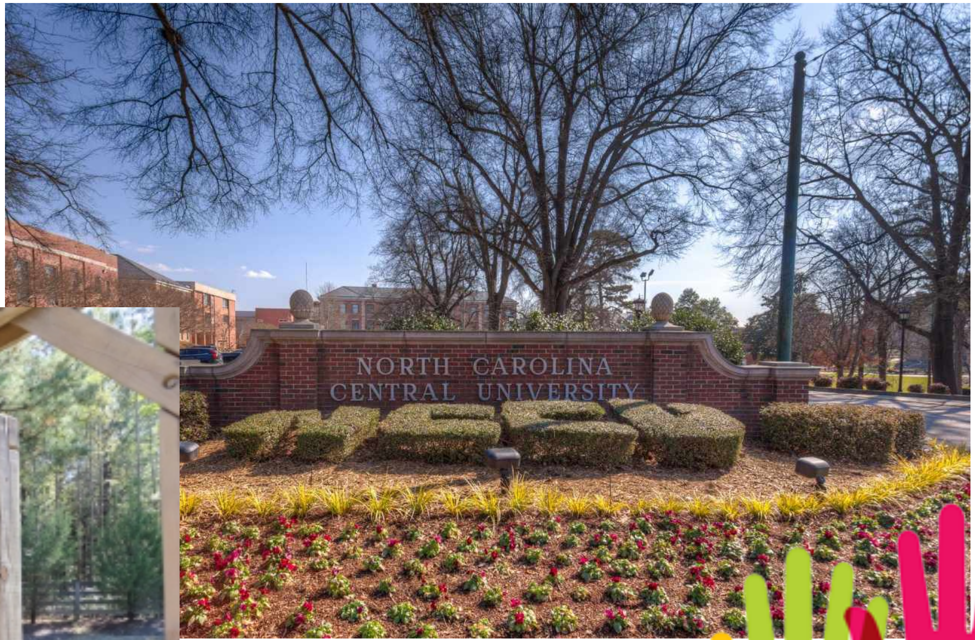

STRUCTURAL INEQAULITY: AN ON THE GROUND VIEW

Durham, North Carolina

2017 Summer Intensive

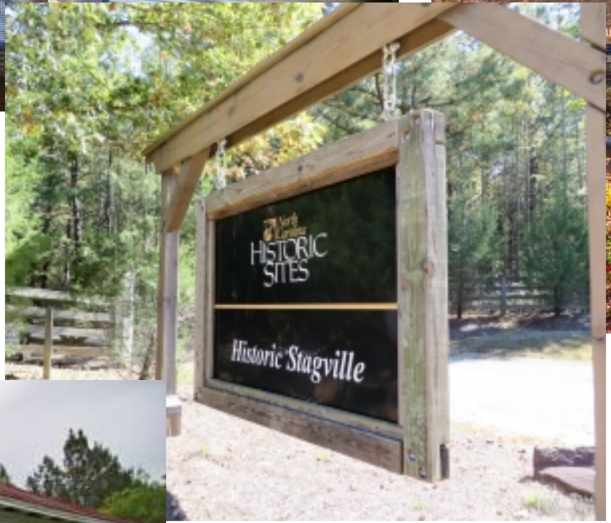

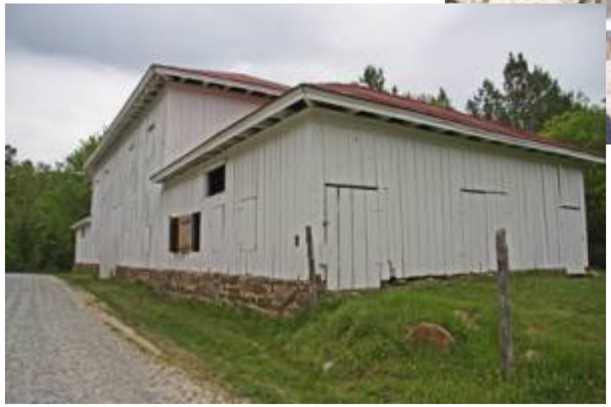

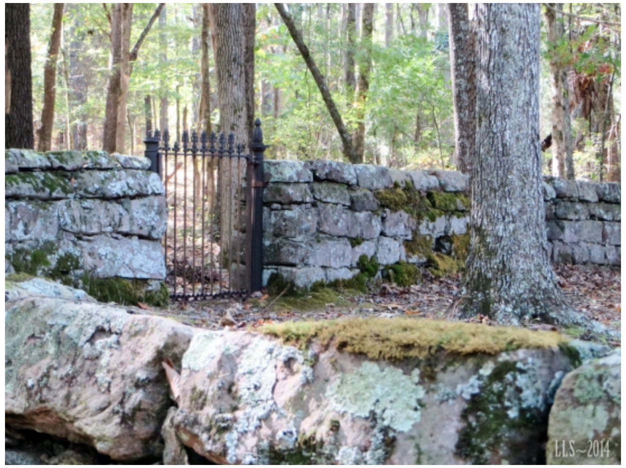

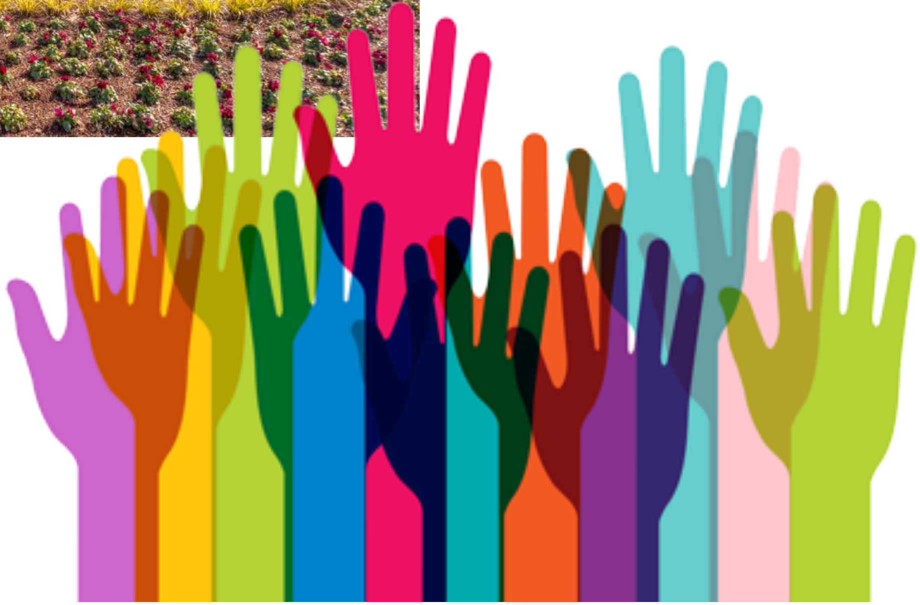

20 YEARS

Community-Campus Partnerships for Health

Promoting Health Equity and Social Justice

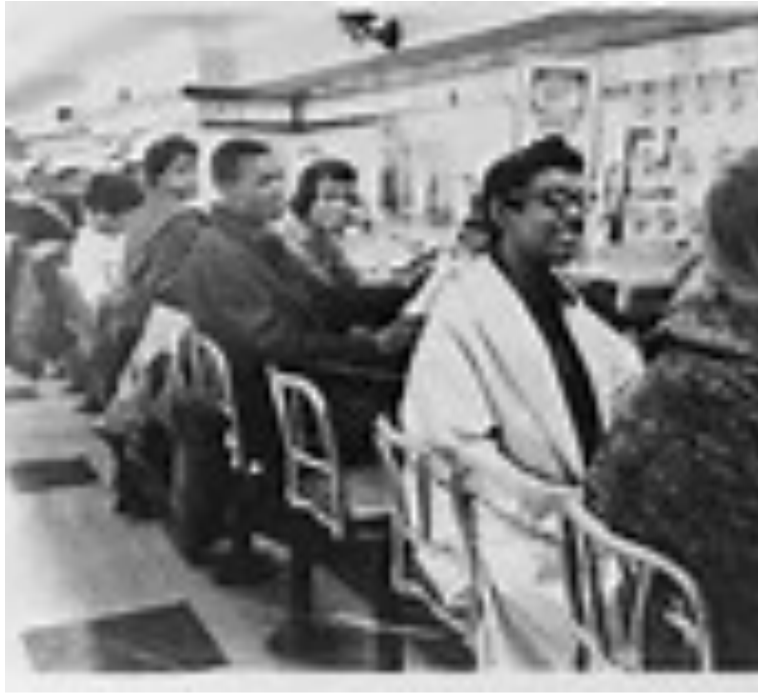

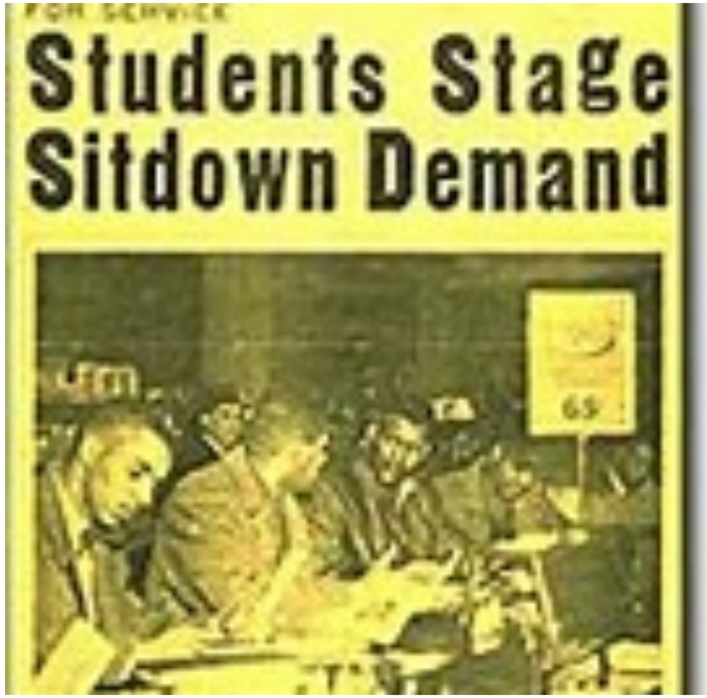
FOR SERVICE

Students Stage Sitdown Demand

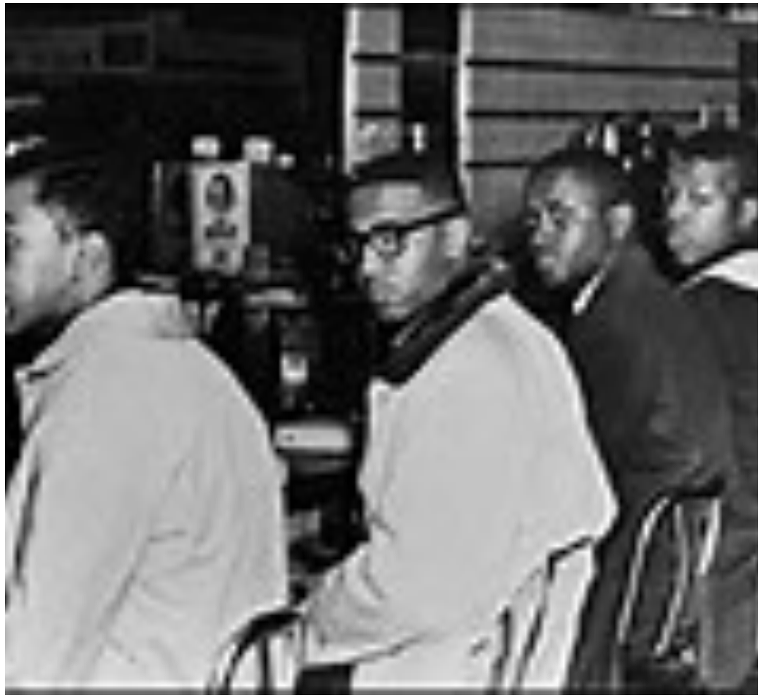

PHD

PRE-HEALTH DREAMERS

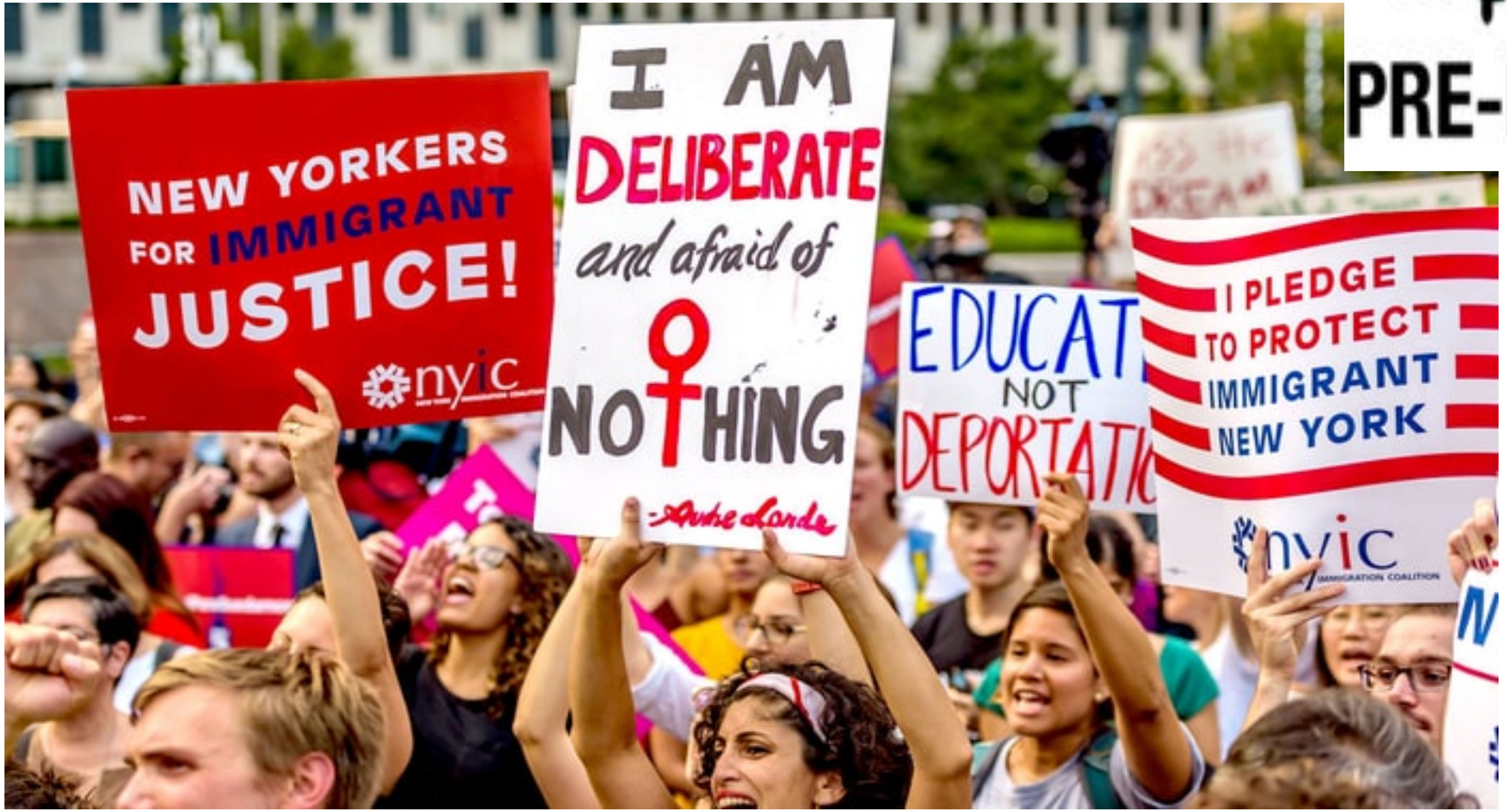

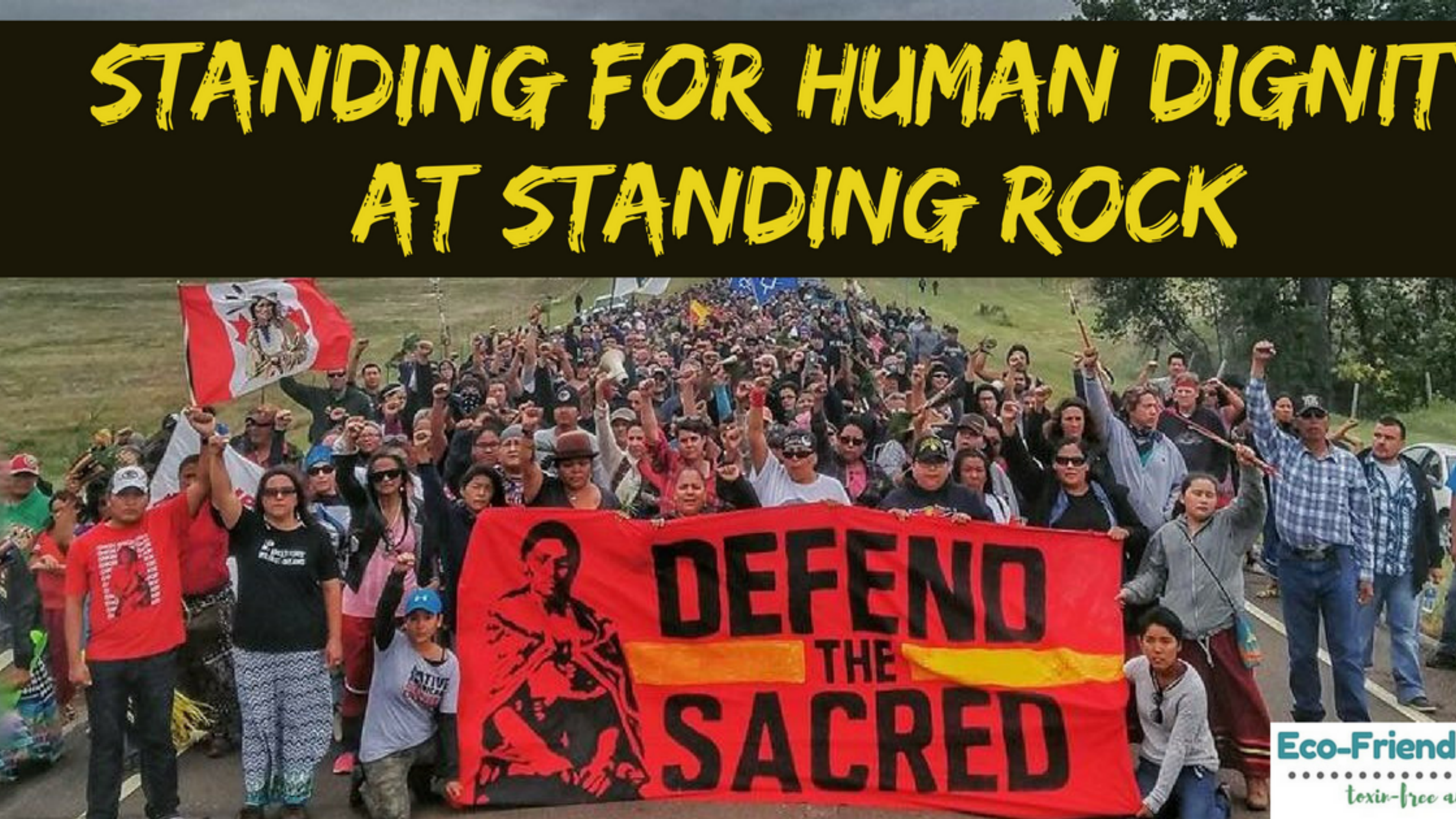
STANDING FOR HUMAN DIGNIT
AT STANDING ROCK
DEFEND
THE
SACRED
Eco-Friend
toxin-free a

CCPH Principles of Partnership

Community-Campus Partnerships for Health

Promoting Health Equity and Social Justice

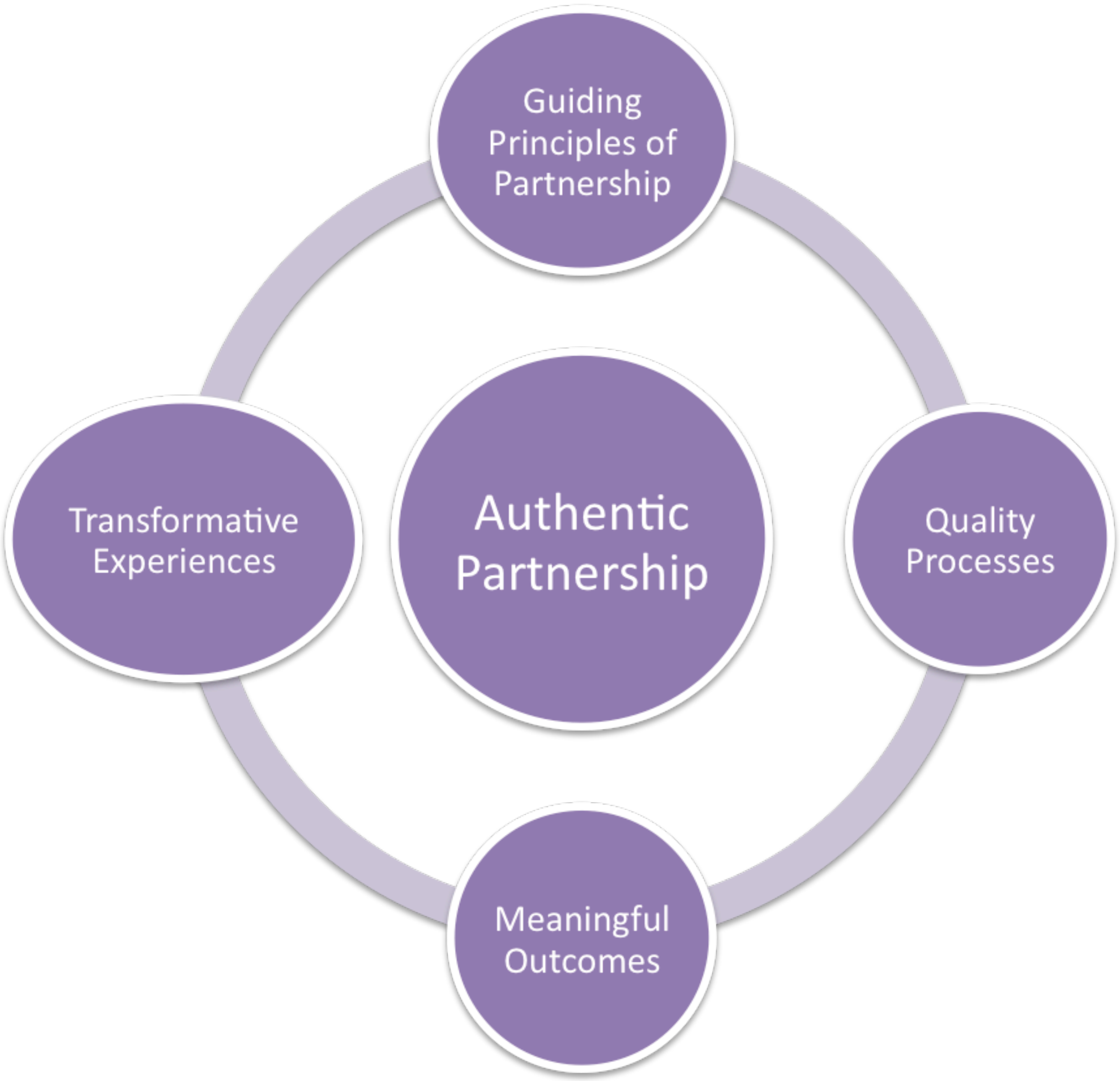

Creating Transformative Experiences

Personal → Inter-personal → Institutional → Community

Community-Campus Partnerships for Health
Promoting Health Equity and Social Justice

Call for Nominations♪

2017 RWJF Award for Health Equity♪

Presented by♪

20 YEARS

Community-Campus Partnerships for Health

Promoting Health Equity and Social Justice

Nomination Receipt Deadline♪
Monday, September 18, 2017♪

Opening Plenary Session: Innovative National and Statewide Community Engagement Initiatives to Improve Health

Community-Based Approaches to Improving Mental Health Equity in Underserved Communities

Sergio Aguilar-Gaxiola

saguilargaxiola@ucdavis.edu

Professor of Clinical Internal Medicine Director,

Center for Reducing Health Disparities University

of California-Davis School of Medicine

Sacramento, California

Increasing access to healthcare for underserved populations is critical to successful community engagement, particularly for those suffering from untreated mental illness in the Hispanic population. Multiple programs have been implemented in California, and efforts focus on programs at the local, state, and national levels. [SLIDE 1. These programs address the gap between those who are in need of services and those who are receiving services, or rather, those who are not receiving services. This is often referred to as the "treatment gap." Sobering estimates indicate that between 50-90% of people with serious mental disorders have not received appropriate mental healthcare in the past year, and this statistic is even worse in some populations. [SLIDES 2, 3] In the U.S., these are the levels of the people who are not receiving care (people who have been diagnosed with one or more mental disorders and are in need of services): for Hispanics, only three out of 10 are receiving care; for African Americans, the numbers are worse; Asian Americans even a bit worse; but even with non-Hispanic whites, six out of 10 are not receiving care. This study was conducted in the Central Valley in California with people of Mexican origin. [SLIDE 4] Of those who were in need of services (with over 1,000 people enrolled in the mid-nineties), one out of three of the U.S.-born people of Mexican origin utilized services, one out of six of the immigrants, and less than one in 10 of the migrant agricultural workers, and these numbers have not changed. Studies are also being conducted in the agricultural areas of the Salinas Valley, but to date, minimal progress has been made. In-depth interviews with subjects indicate many have suffered a decade or more with severe mental illness and have not received services, though many did seek help. Of those who do receive services, there are studies that show up to 75% of Latinos do not continue treatment. There are multiple reasons for this, including language barrier and cultural differences. [SLIDE 5] There are also significant comorbidities that need to be addressed; mental disorders are often accompanied by substance abuse or other general health conditions like diabetes. Without treatment, mental health disorders do not simply resolve over time and actually tend to intensify. Such disorders result in truncated life expectancy by 25 years, and some data obtained in the last 15 years indicates that life expectancy has been even

worse (not to mention quality of life). Estimates here in the U.S. for two decades indicate that approximately 50% of the population will suffer from one or more mental illnesses, including substance abuse. This leads to significant costs to individuals, families, and their communities, though the extent of these repercussions is difficult to measure. The cause of the treatment gap experienced by some communities is an important area of study, particularly identifying the barriers that affect access to care. [SLIDE 6] For instance, the stigma associated with mental illness is a significant obstacle, as well as a lack of sufficient and appropriate health workforce. One of the most significant issues is the lack of engagement by those in need of services such as behavioral healthcare or in some cases, even primary care. These barriers represent tremendous challenges. Disparities must be considered in the context of a growing demographic diversity. [SLIDE 7] This translates into significant burdens of diverse populations – racially, ethnically, geographically, culturally, and linguistically – resulting in ill health, premature death, and diminished productivity and social potential. This truly constitutes a major U.S. public health problem.

[SLIDE 8] California has implemented the "Mental Health Service Act," which was passed into law and went into effect 12 years ago. This program increases funding for mental health programs, especially in the area of disparities, by applying a 1% tax on those who make one million dollars or more. In 2017, the tax collected surpassed two billion dollars and constitutes roughly 17% of the operating money for the counties. Program components include *Community Services and Support* and *Prevention and Early Intervention and Work Force Education and Training*, as well as *Innovation*, which focuses on creative approaches to promote recovery and resiliency. Another program, "California Reducing Disparities Project," [SLIDES 9, 10] has two phases and millions of dollars to focus on the disparities that five populations have been experiencing: African Americans, Asian and Pacific Islanders, Latinos, LGBTQ, and Native Americans. From this program, population reports and a strategic plan were developed. The second phase started in 2017, [SLIDE 11] with 60 million dollars invested in this effort from the Mental Health Services Act. There are approximately 40 projects, including a statewide evaluation and 35 "implementation pilot projects." Instead of funding specific evidence-based practices, the state decided to invest in what is called "community-defined evidence." These are promising programs and practices that are being implemented by community-based organizations, and this money goes to identify community-defined evidence and practices and to provide resources for five years to raise the level of evidence.

With these ongoing resources from the state, the counties will get some of those community-defined evidences and continued funding. This second phase funds efforts to identify promising practices and systems change recommendations to address disparities, specifically in historically underserved populations. At the local level, [SLIDE 12] the rural county of Solano sought assistance to advance progress in the areas of community engagement and improved public health among underserved communities. This is the first county to design a multi-phase innovation training and transformation project [SLIDE 13] that combines cultural and linguistically-appropriate services with community engagement [SLIDE 14] with the goal of increasing access to mental health services and workforce diversity. There are three phases to the Solano County program. [SLIDE 15] The first phase brings together three groups of stakeholders: community leaders, community-based organizations, and the counties. [SLIDES 17, 18] The second phase involves training using a curriculum that has been refined over 10 years. [SLIDE 19] The three target groups are trained, along with representatives from various sectors, from social services, schools, churches, criminal justice, transportation, etc., to develop quality improvement projects. [SLIDE 20] The goal is to develop specific action plans to be implemented in the third phase, from culturally-competent care all the way to community engagement. [SLIDE 21] To streamline services, multiple factors are taken into account: consumer experience, provider experience, health outcomes, and cost-effectiveness as determined by a health economist and epidemiologist. [SLIDE 22]

While it is important to continue to invest in access to healthcare, there are other factors to consider. [SLIDES 23, 24] Healthcare only constitutes 10% of keeping the population healthy; other issues affecting health are personal behaviors (40%), family history and genetics (30%), as well as environmental and social factors. [SLIDE 25] Healthcare is certainly a significant part of this, but treating an illness without addressing other factors will not be effective in improving public health. [SLIDE 26] To quote Tom Frieden, "Addressing socioeconomic factors has the greatest potential to improve health. Achieving social and economic change might require fundamental societal transformation. Interventions that address social determinants of health have the greatest potential for public health benefits." [SLIDE 27] Community health programs must address health inequities, unhealthy housing, environmental hazards, limited access to food, exercise, safe neighborhoods, the impact of stress, and numerous other variables affecting public health.

There are a number of issues to address [SLIDE 28]: 1) identifying patients, not medical health needs, as part of overall car, 2) connecting patients to local services and resources to help prevent

illness or to better manage it, 3) developing strong leaders to collaborate with other sectors to improve health, 4) connecting community residents to jobs in the healthcare sector, some of which are the largest employers, and 5) linking community health workers with patients who need support.

Declarations

- The presenter has no financial competing interests related to this presentation.
- All authors have approved the manuscript for submission
- The content of this presentation and transcript has not been published or submitted for publication elsewhere.
- This presentation was organized by the Meharry-Vanderbilt Alliance with support from NCATS and the NIMHD of the National Institutes of Health under award number R13TR001694

Advancing the Science of Community Engagement Research Conference

Community-Based Approaches to Improving Mental Health Equity in Underserved Communities

Sergio Aguilar-Gaxiola, MD, PhD
Professor of Clinical Internal Medicine
Director, Center for Reducing Health Disparities
UC Davis School of Medicine

Washington, DC
September 14, 2017

UCDAVIS HEALTH
Center for Reducing Health Disparities

The "Treatment Gap"

Between 50 to 90% of people with serious mental disorders have not had received appropriate mental health care in the previous year.

Treatment Gap in the U.S.

- Levels of **unmet need** (not receiving specialist or generalist care in past 12 months, with identified diagnosis in the same period)
  - Hispanics – 70%
  - African Americans – 72%
  - Asian Americans – 78%
  - Non-Hispanic Whites – 61%

Source: Alegria et al., 2006

Mexican American Prevalence and Service Survey (MAPSS)

Who Utilized Services?

- 38% of U.S. born received care
- 15% of immigrants received care
- 9% of migrant agricultural workers received care

Source: Aguilar-Gaxiola, Vega, et al., 2000

Untreated Mental Illness

- Intensify over time…can reduce life expectancy
- Causes intense and prolonged suffering to individuals and their families
- Limits individuals' ability to reach social and educational normative goals
- Leads to significant costs to individuals, families and communities

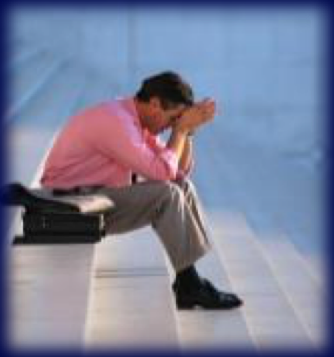

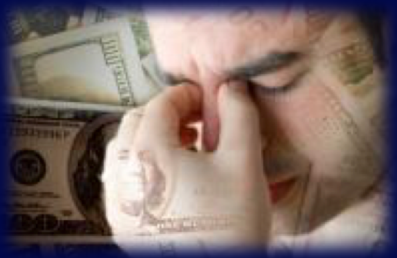

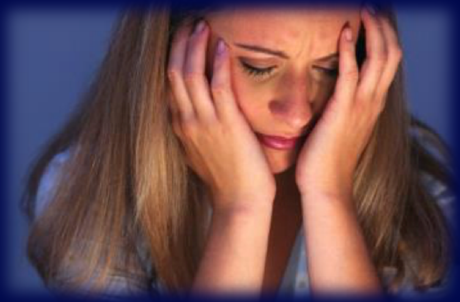

Why the Treatment Gap?

- Multiple barriers
  1. Individual level (e.g., stigma)
  2. Community level (e.g., lack of culturally and linguistically appropriate services)
  3. Systemic level (e.g., lack of a sufficient and appropriate health workforce)
- Lack of engagement in behavioral healthcare

Significance of Disparities

- In the context of growing demographic diversity in U.S.
- Significant burden of unmet mental health needs among diverse racially, ethnically, geographically, culturally and linguistically diverse populations
- Translates into ill health, premature death, diminished productivity and social potential, wasted resources
- A major U.S. public health problem

Source: Primm, 2009

State Level

The Mental Health Services Act (MHSA)

- The Mental Health Services Act (MHSA) was passed by California voters on November 2004 and went into effect in January 2005.
- The MHSA provides increased funding for mental health programs across California.
- The MHSA is funded by a 1% tax surcharge on personal income over $1 million per year.

California Reducing Disparities Project (CRDP)

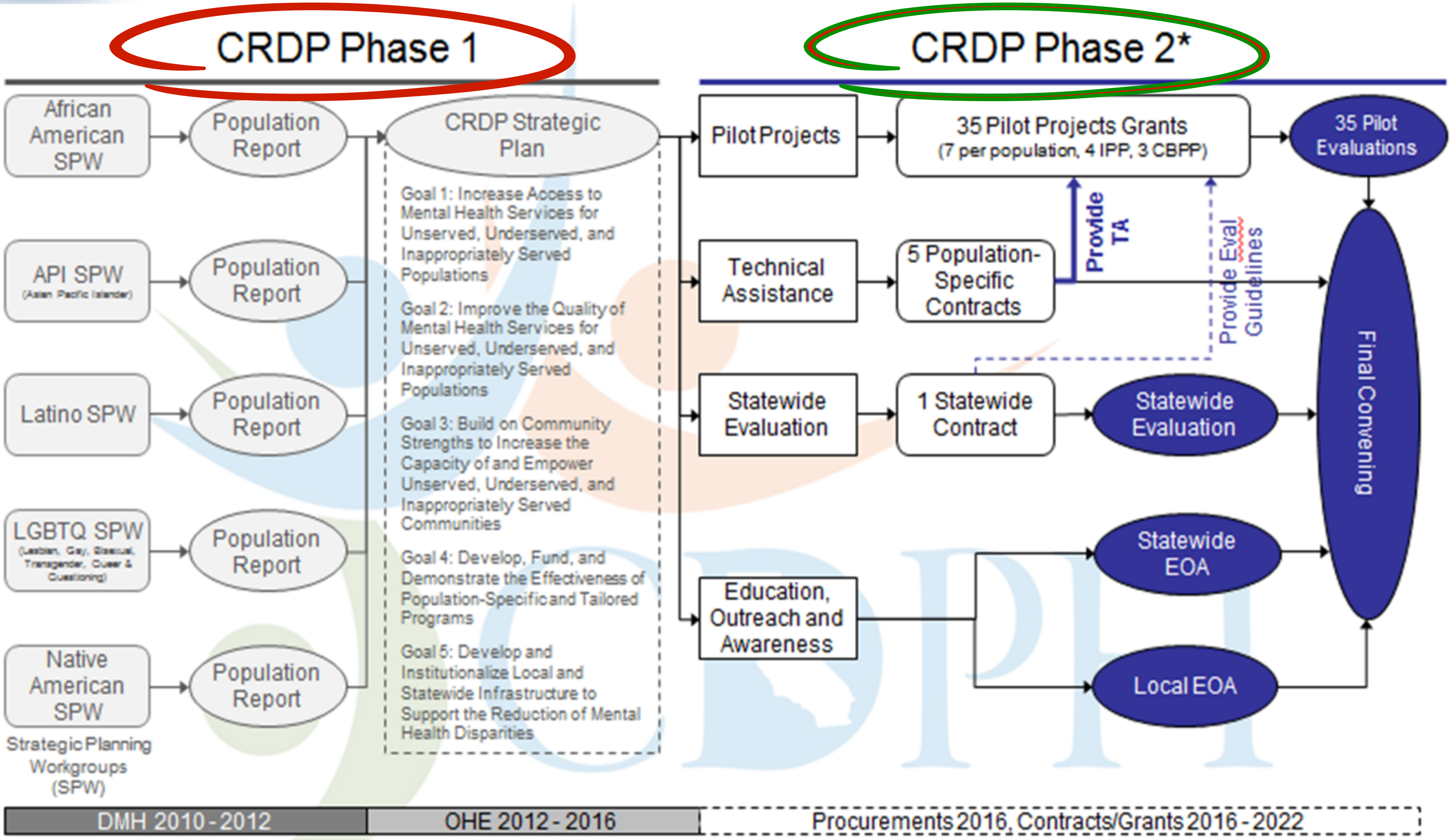

* In process, specific details subject to change

CRDP Phase I Population Reports

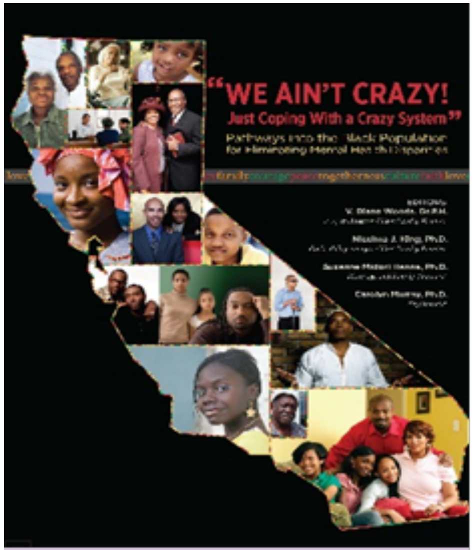

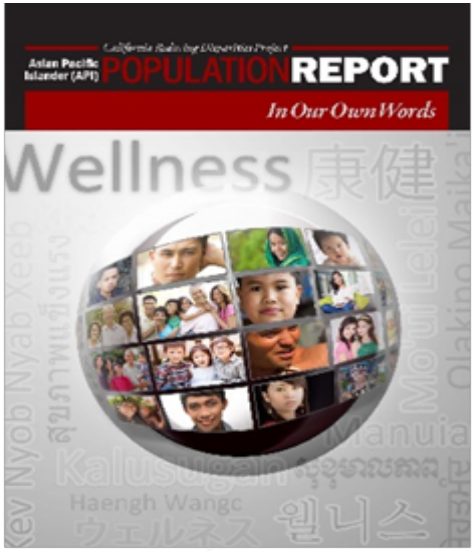

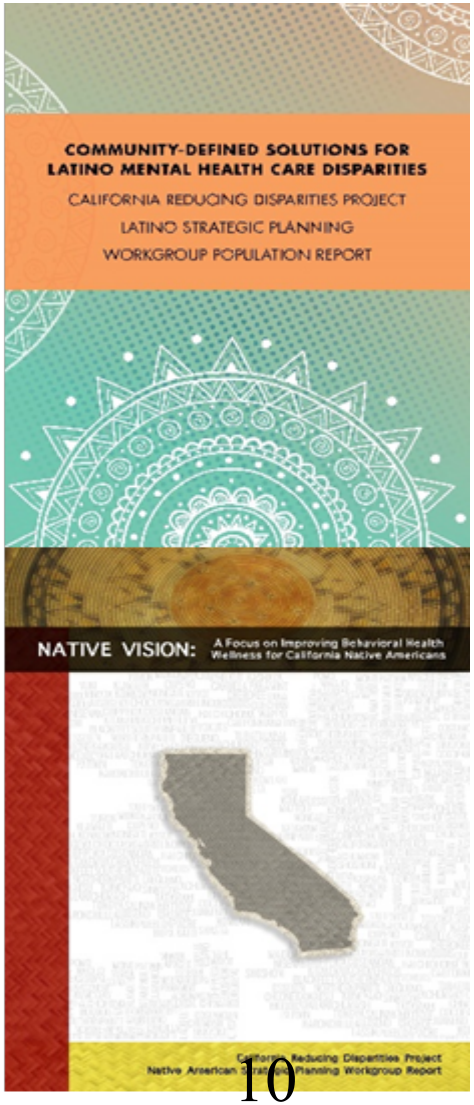

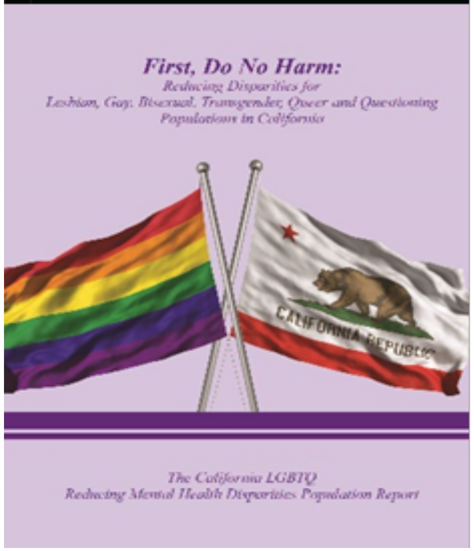

CDPH California Department Of PublicHealth

California Reducing Disparities Project (CRDP)

Population Reports

CRDP Phase II

- MHSA funded $60m initiative to identify promising practices and systems change recommendations to address persistent disparities in historically underserved populations.

- Priority Populations:

  African American; Asian and Pacific Islander; Latino; LGBTQ; and Native American communities

- In total, over 40 contractors and grantees are funded over six years to implement Phase II of the CRDP.

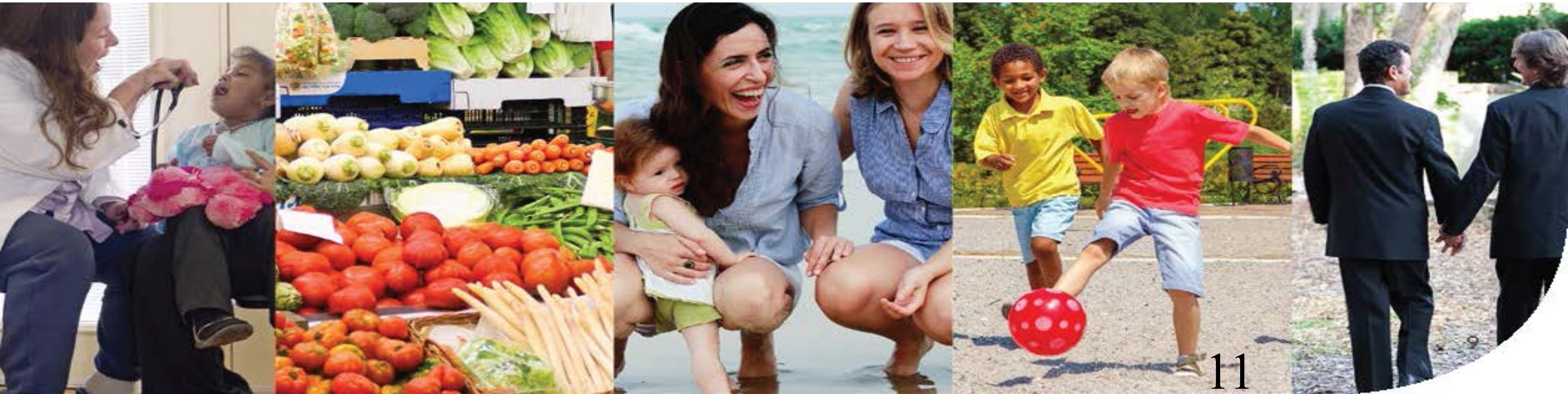

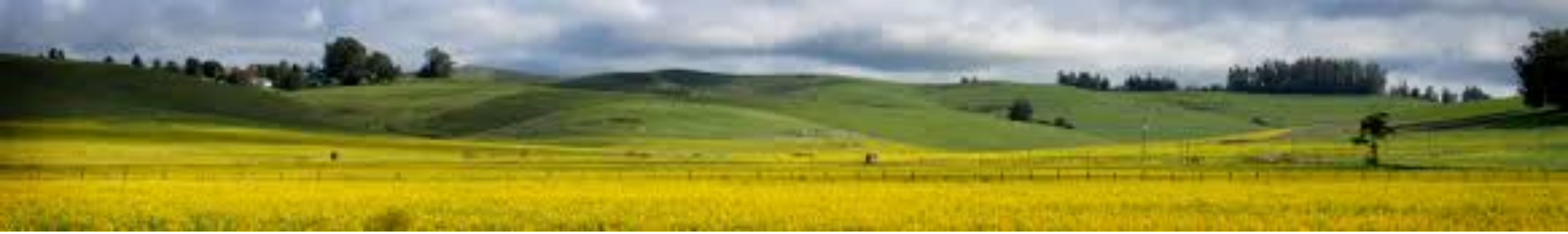

Solano County Mental Health Interdisciplinary Collaboration and Cultural Transformation Model

First county to design a multi-phase innovation training and transformation project that combines CLAS with community engagement

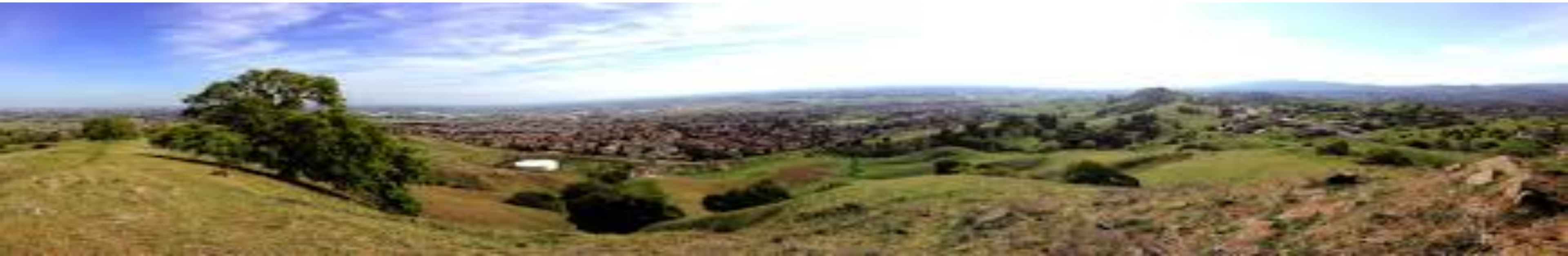

MHSA Innovation – Nuts & Bolts

- Innovation programs and services must be:
  - Unique and creative: new, not just “new to me”
  - Promote recovery and resiliency
  - Developed with communities, including underserved populations within communities
  - Lead to system reformation and new approaches

Project Goals

- **Improve access to and utilization of mental health services** for Latino, Filipino American and LGBTQ communities
- **Enhance collaborative partnerships** between County, Community and CBOs
- **Increase workforce diversity**
- **Develop organizational policies, programs and support systems** to ensure and sustain cultural and linguistic competency in service delivery

MENTAL HEALTH SERVICES ACT
PROPOSITION 63
CALIFORNIA

WELLNESS • RECOVERY • RESILIENCE

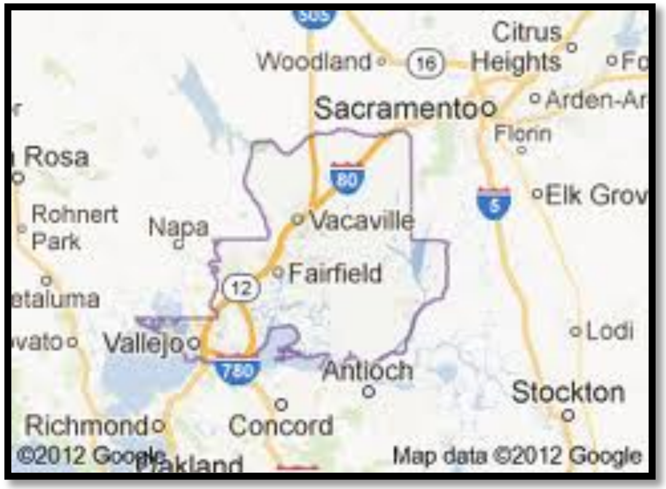

Penetration Rates FY 2013-2014

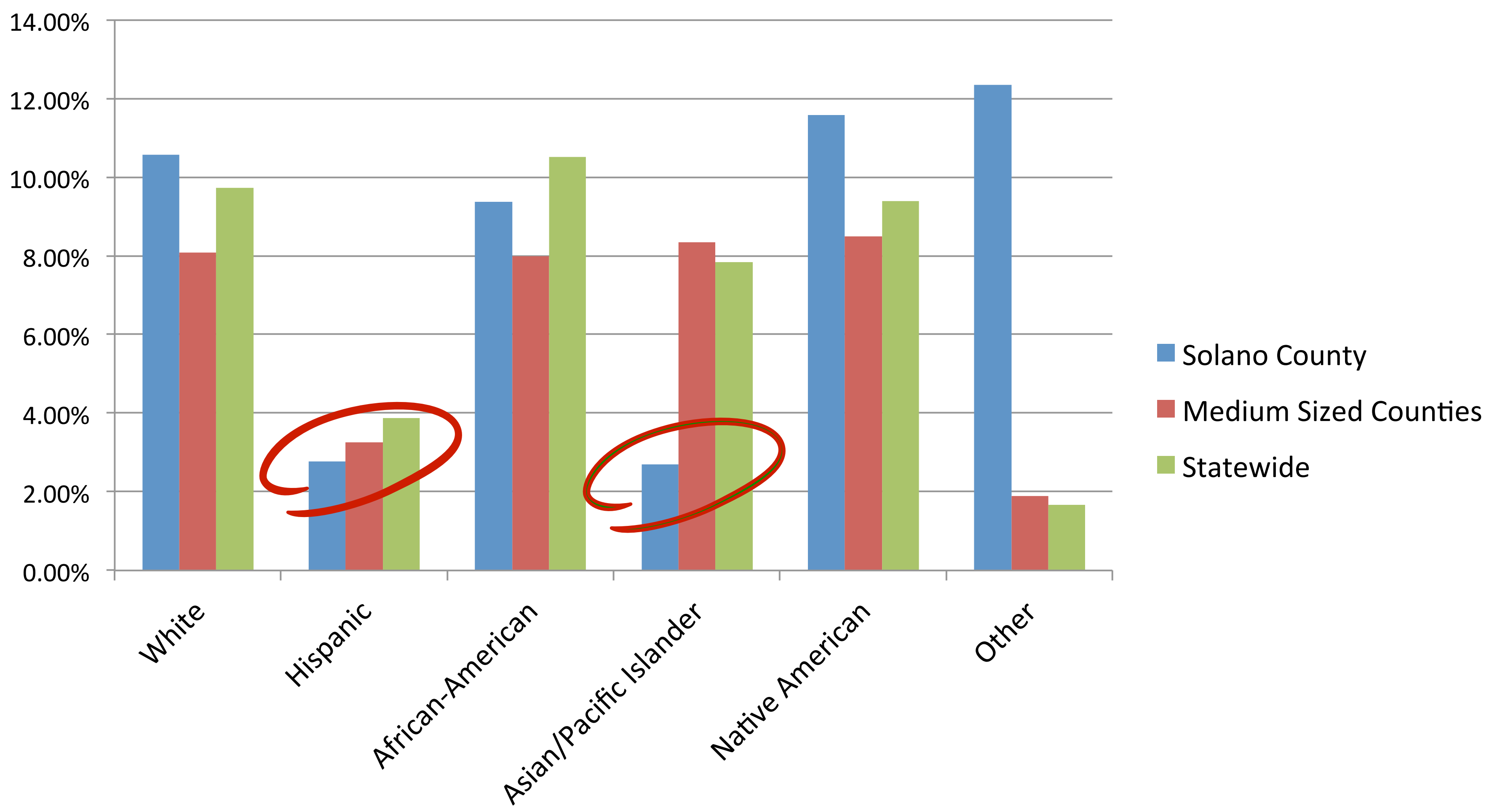

The Project at a Glance

**Phase 1** Years 1-2	Collect stories, histories and strengths of the Latino, Filipino American and LGBTQ communities, Solano County and community-based organizations (CBOs), and build on them	Comprehensive cultural health assessment
**Phase 2** Years 2-3	Develop a CLAS leadership and program development training specific to the health and mental health needs of Solano County and use this transformational knowledge as foundation for quality improvement (QI) plans	CLAS leadership development & training program
**Phase 3** Years 4-5	Coordinate and implement QI plans with coaching from a team from the Center for Reducing Health Disparities and feedback from the community to accomplish positive outcomes	QI planning & implementati on
**Evaluation** Years 1-5	Evaluate joint QI plans using a "Quadruple Aim" mixed-methods (qualitative and quantitative) approach that will examine: consumer experience, provider experience, health outcomes and cost effectiveness	Quadruple aim & sustainable community engagement

Discovering the Stories, Strengths and Histories

Phase I: Organization Cultural Assessment

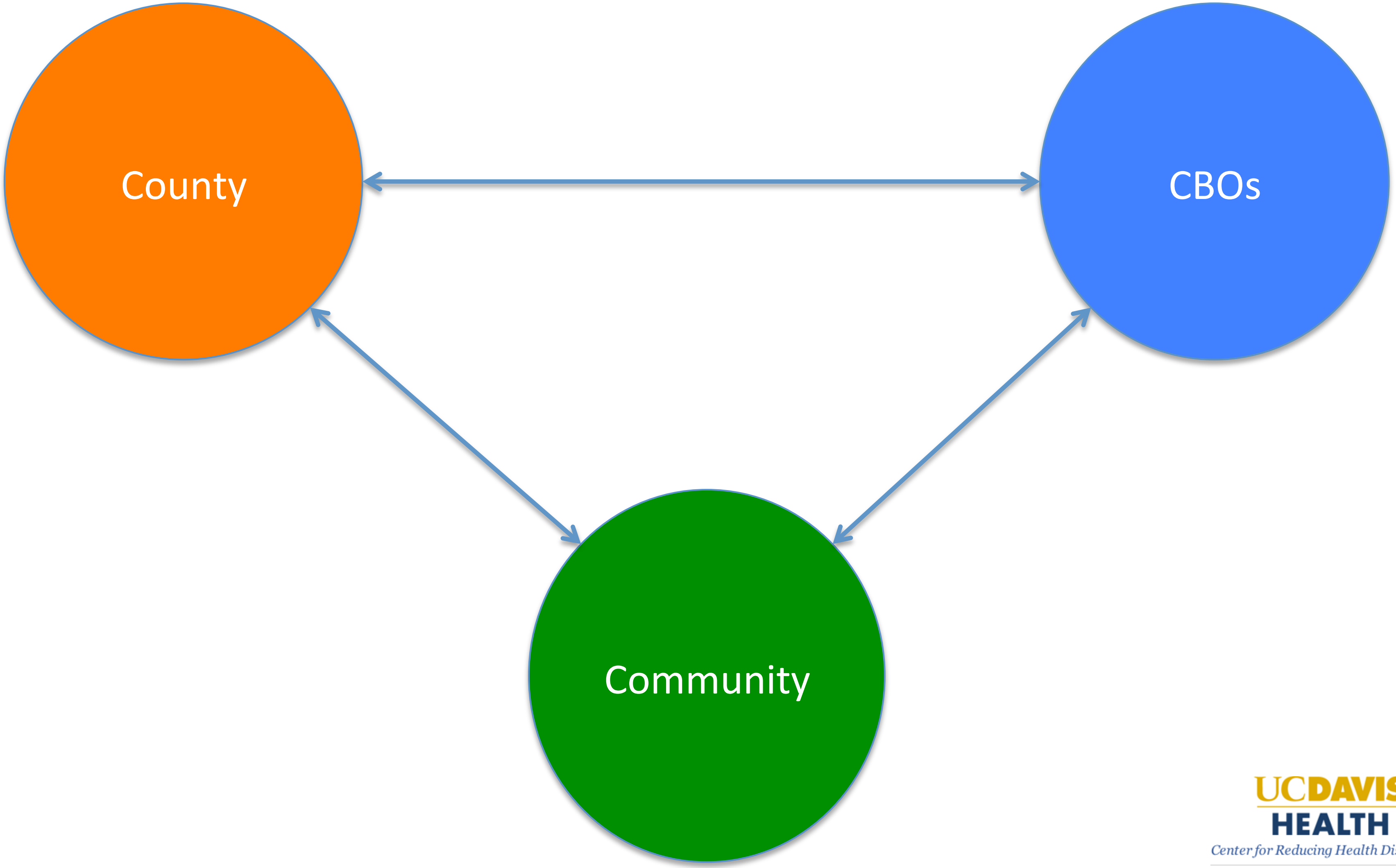

Who we have talked to:

Type	Number of Participants	Detail
Key Informant Interviews		Interviews conducted with community leaders and advocates, including faith, CBO and school leaders
Latino	14	
LGBTQ	14	
Filipino	12	
Consumers	6	Aggregated
Focus Groups	37	Aggregated for 8 focus groups
County staff	12	Administrative and Programmatic staff and leaders
Providers	13	Primarily County Behavioral Health Providers
CBOs (Online Survey)	20	CLAS Self-Assessment Survey
Other County Leaders	10	Transportation; Housing; Insurance; Other Health Systems
Community Forums	61	1 CF per community
Total Number	199	

A Shared Culture of CLAS

Phase II: CLAS Transformational Curriculum Training

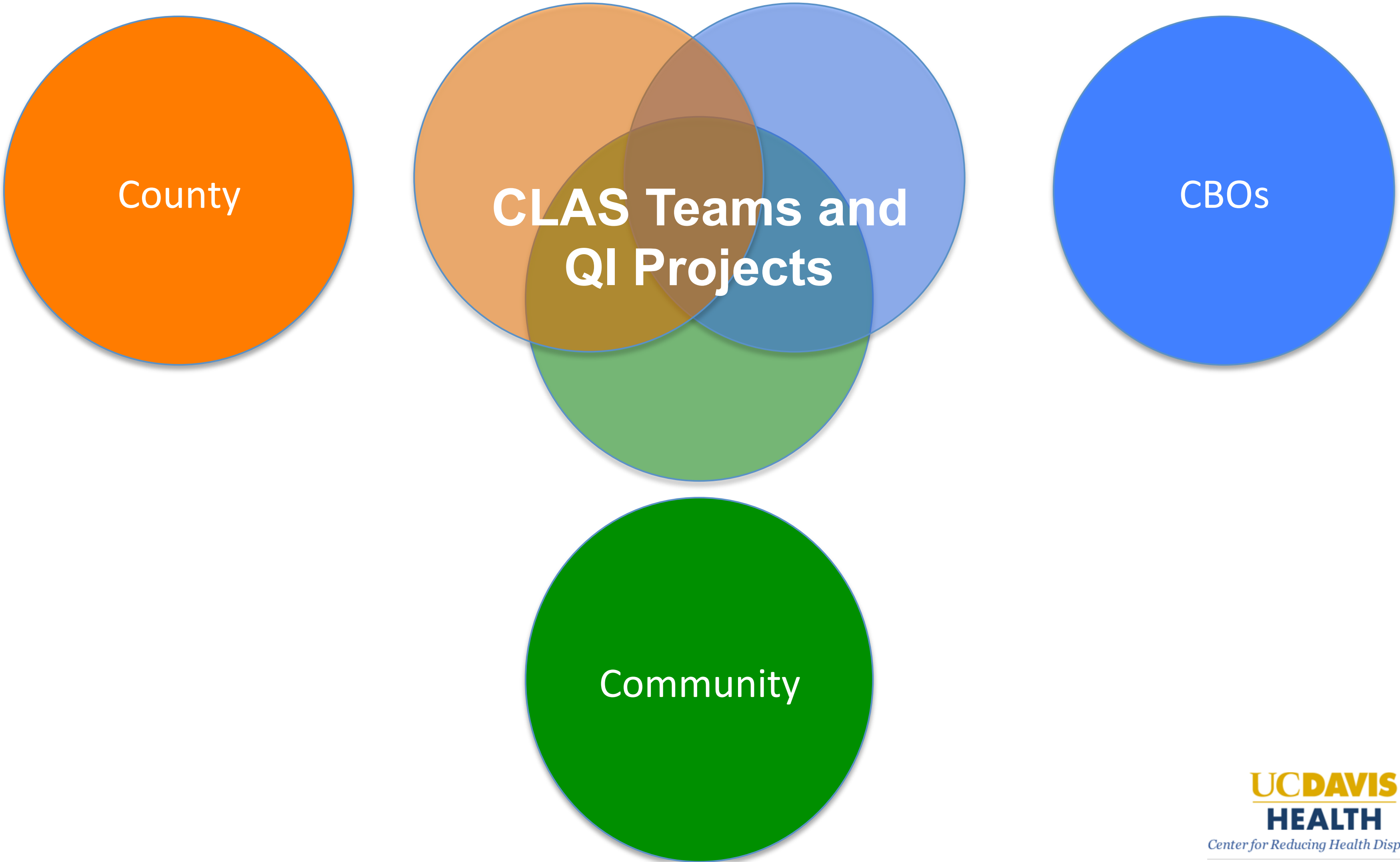

Session 1: Overview

- Current State of Mental Health Experienced by 3 communities
- What Are the CLAS Standards and How Can They Drive Solutions
- Introduction to System Change

Session 2: From Person Centered Care to a Vision of Wellness

- Mental Health Narratives: Discussion Panels
- Appreciative Inquiry: A Strength-based Approach to System Design

Session 3: Getting to Know the CLAS Standards

- Team Presentations on CLAS Standards: What they are and potential implementation strategies.
- Model programs and best practices

Session 4: Quality Improvement Projects

- Leadership Talk
- Discuss QI Plan Feedback
- Coordinate QI Plans for maximal impact and support

Implementation Coaching Sessions (6 monthly meetings)

Walking the Talk

Phase III: Implementation

Coordinated QI Programs

- Culturally Competent Care
- Language Supports
- Diverse Workforce and Leadership Development
- CLAS Policies and Procedures
- Community Engagement

"Quadruple Aim" Outcomes

- Consumer Satisfaction Surveys
- Focus Groups
- Utilization Rates

- Provider Satisfaction/ Burnout* Indicators
- Social Network Inventories

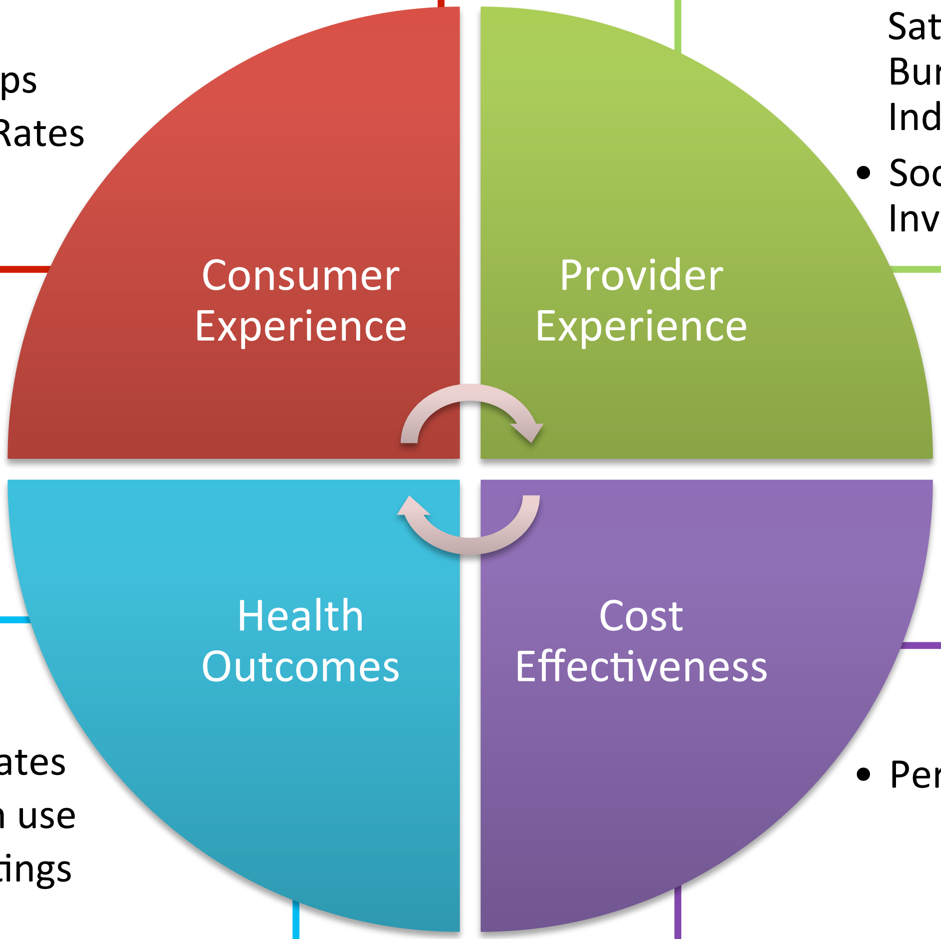

- Hospitalization rates
- Emergency room use
- Psychometric ratings (i.e., BDI, PHQ, Hopkins)

- Per capita costs

Source: Bodenheimer & Sinsky, From Triple to Quadruple Aim, 2014

Is it possible to improve community mental health by focusing primarily in access to care?

It is more than access to care…

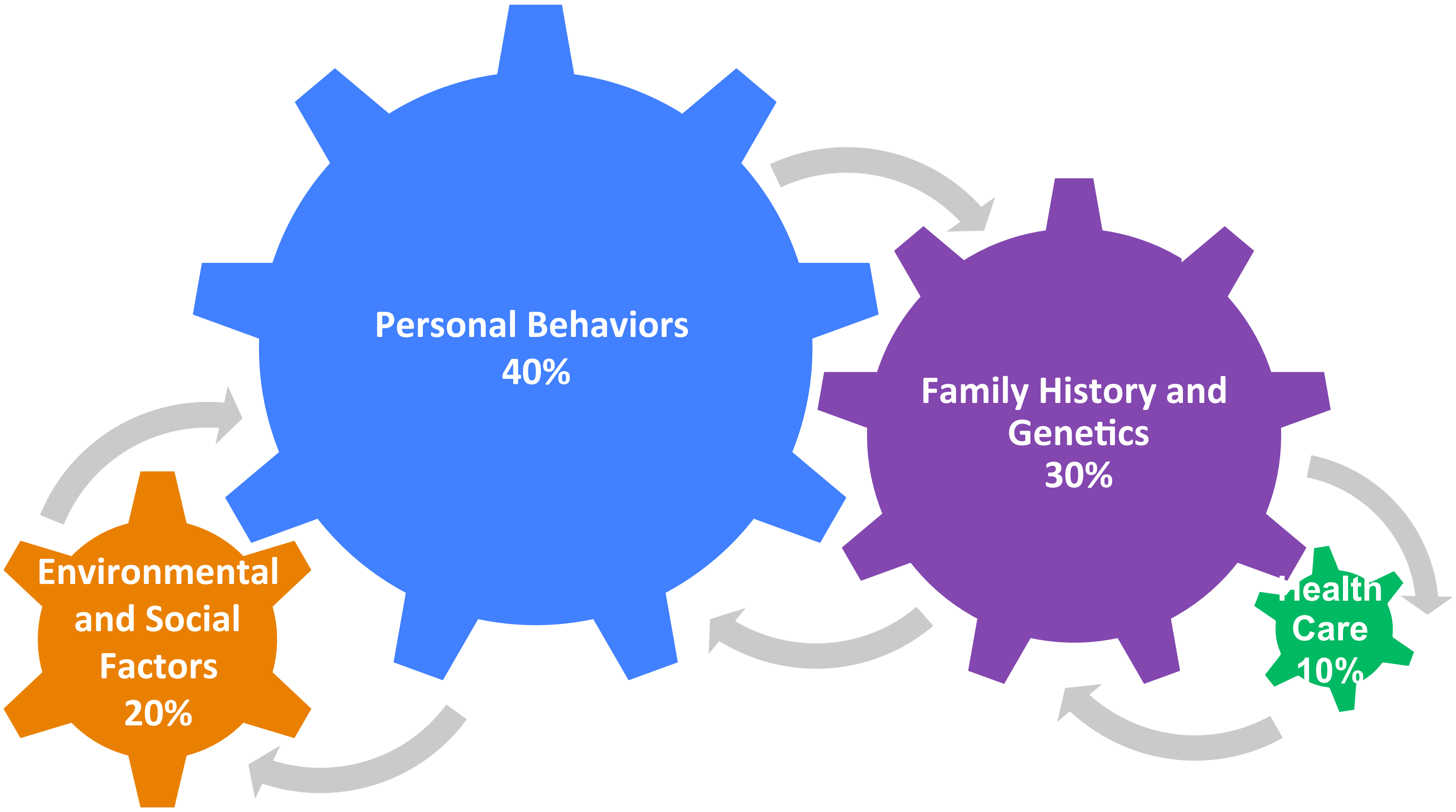

Health is driven by multiple factors that are intrically linked-of which **access to health care is one component**

We need Care that Addresses the Social Context

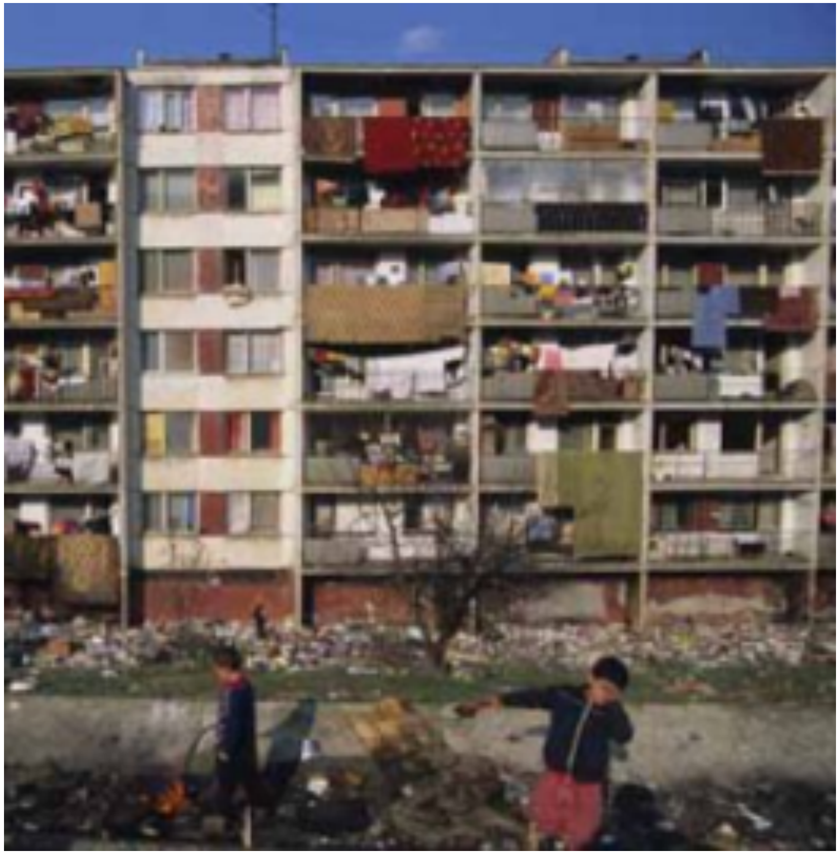

Why treat illness and send people back to live in the same conditions that made them sick in the first place?

Source: Williams, 2016

UC DAVIS HEALTH
Center for Reducing Health Disparities

Determinants of Health: Focus on policy, systems, and structural change

"[A]ddressing socioeconomic factors has the greatest potential to improve health.... Achieving social and economic change might require fundamental societal transformation.... Interventions that address social determinants of health have the greatest potential for public health benefit."

CDC Director Dr. Thomas Frieden

Source: Frieden, A Framework for Public Health Action: The Health Impact Pyramid," *American Journal of Public Health*, 2010

Coming to Terms with Health Inequities

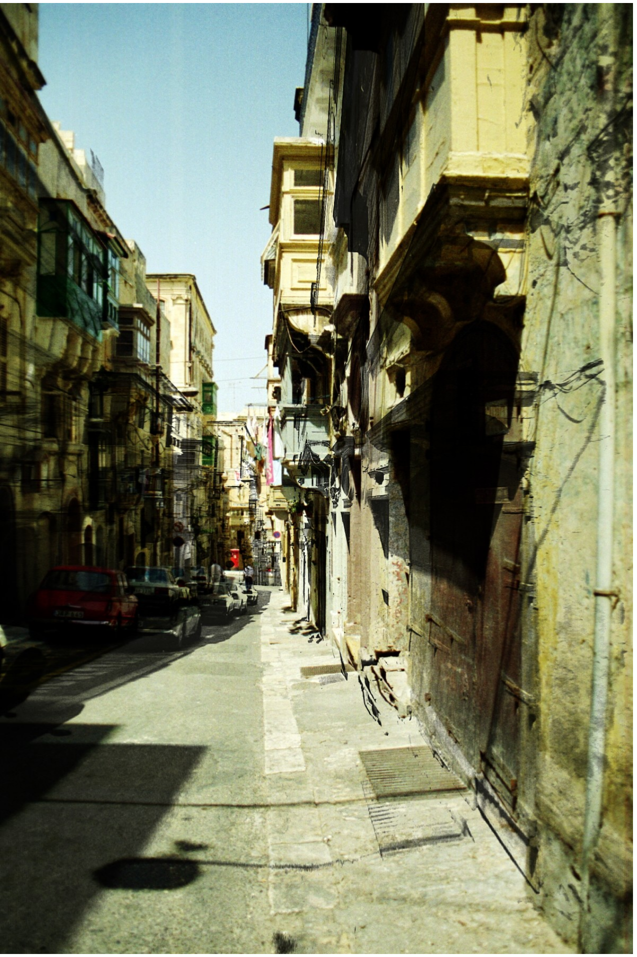

- Unhealthy housing
- Exposure to array of environmental hazards
- Limited access to healthy food & basic services
- Unsafe neighborhoods
- Lack of public space, sites for exercise
- Limited public transportation options
- Inflexible and/or poor working conditions
- Impact of chronic stress

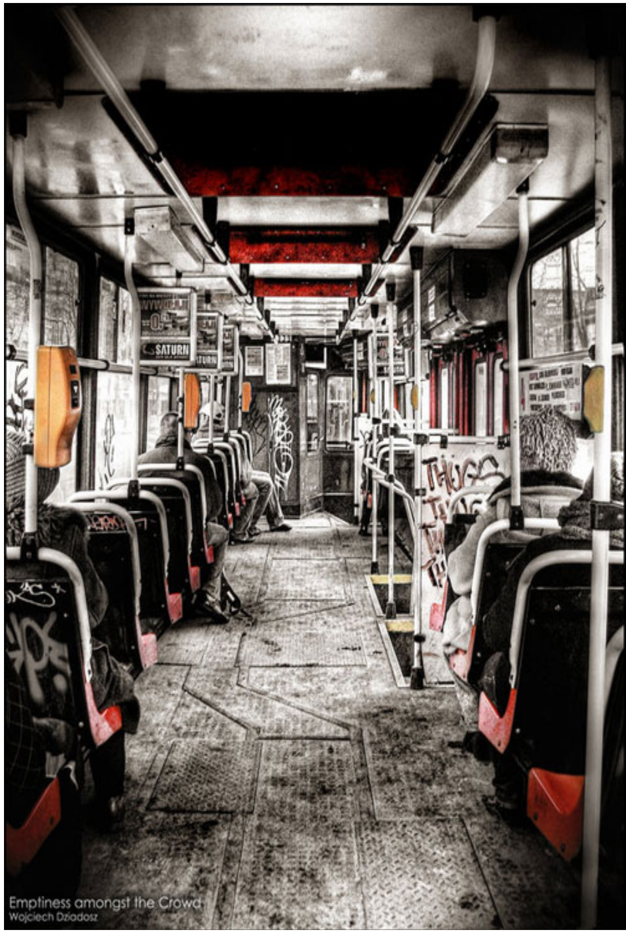

Source: Barnett, 2017

UCDAVIS HEALTH
Center for Reducing Health Disparities

New Directions

- How can we identify patients' non-medical health needs as part of their overall care?
- How can we connect patients to local services/resources that help people avoid getting sick in the first place or better manage illness, including mental health needs?
- How can we be a strong leader and champion to collaborate with other sectors to improve health where patients live, learn, work and play?
- How can we connect community residents to jobs in the health care sector – one of the largest employers?
- How can we use community health workers to provide services or link patients to needed supports?

Source: Williams, 2016

UC DAVIS HEALTH
Center for Reducing Health Disparities

Special Session

Community Engagement in Minority Health Research to Reduce Disparities

Eliseo J. Pérez-Stable

eliseo.perez-stable@nih.gov

Director, National Institute for Minority Health and Health Disparities

Bethesda, Maryland

Abstract

In this presentation, Dr. Eliseo Pérez-Stable describes the history of the National Institute for Minority Health and Health Disparities (NIMHD), defines health disparity populations as perceived by this NIH center, and discusses research and policy priorities that are important for reducing health disparities in the United States. He is introduced by Dr. Meryl Sufian, PhD, a Program Officer with the NIMHD in Integrative Biological and Behavioral Sciences

[*Meryl Sufian, introducing the speaker:*] Eliseo Pérez-Stable has been the Director of the National Institute of Minority Health and Health Disparities for just two years and, just briefly, because he is going to be talking more about this, the NIMHD seeks to advance minority health and health disparities through research, training, research capacity development, public education, and information dissemination. Dr. Perez-Stable has been leading research in smoking cessation in Latinos for over 30 years, both in the United States and Latin America. Prior to coming to NIMHD, he was Professor of Medicine at the University of California, San Francisco. He has received many awards throughout this career. He has authored many scientific papers, reviewed articles for professional publications, delivered keynote lectures, like he is today, and presentations, both at domestic and international conferences. He earned his Bachelor's Degree in Chemistry from the University of Miami, his M.D. also from the University of Miami, and he completed his primary care internal medicine residency and research fellowship at UCSF. [SLIDE 1]

[*Eliseo Pérez-Stable*] Good afternoon, everyone. It is so great to see all these wonderful faces here for this important meeting on this critical topic, so I am really honored to be able to address you today. I am going to introduce the NIMHD and address the issue at hand regarding community engagement. [SLIDE 2] NIMHD started as a consequence of Dr. Sullivan [*then Secretary of the U.S. Department of Health and Human Services, Dr. Louis Sullivan*] starting an office in the office of the NIH Director in 1990. Dr. John Ruffin was named the Director of that office in 1990 and he remained throughout until he retired in 2014. The critical step occurred in 2000, led by representative Stokes [*Representative Louis Stokes, D, OH*] in Congress ... the office was

elevated to a center, and for those who know the NIH structure, as a "center," you get a seat at the table. You are now able to generate grant programs. It started a whole process of what minority health and health disparities research would be about. Then, as part of the Affordable Care Act (ACA) in 2010, there was a requirement that all the agencies in Health and Human Services have an Office of Minority Health. The Office of Minority Health already exists in the Secretary's office. NIH had the center and as part of the ACA, we were elevated to an institute. In March 2014, Dr. Ruffin retired. Dr. Yvonne Maddox became acting Director, and then I started on September 1, 2015. Our budget this year was $289 million. Our mission is dual. [SLIDE 3] We are interested in minority health and health disparities and to distinguish those two areas: Minority health is everything related to racial-ethnic minority groups in the U.S., whether the outcomes are good or not good. This is in terms of improving health, keeping it well, or making it even better, as well as understanding mechanisms and advancing knowledge. We are also mandated to look at health disparities. These are outcomes that are worse in disadvantaged groups and using that knowledge to develop interventions to reduce the disparities. We have a very small training portfolio. We hope to expand that or evolve that in some ways, but we are totally committed to diversity of the biomedical research workforce and work closely with other institute directors as well as Hannah Valantine (MD MRCP), who is the Chief Diversity Officer at NIH.

Minority health definition ... [SLIDE 4] anything that relates to minority health, and we really endorse the idea that a common bond is being subject to discrimination historically. As far as racial-ethnic groups, this is what the Office of Management on Budget lists. [SLIDE 5] 2020 may see some slight changes. These are the official names used by government. As Director of NIMHD, I am empowered to decide who is a health disparity population. [SLIDE 6] I am mandated to consult with the Director of AHRQ and we are supposed to agree on that. Racial-ethnic minorities, less privileged socioeconomic status of any race, so poor people, and "underserved rural residents" to emphasize that we are still looking at poor as well as minorities in rural areas that have unique characteristics. [Slide 7] Last year, in October, we declared sexual gender minorities a disparity population for NIH research purposes and that got through the department, I think in large part because of the persuasion that they are subject to discrimination in society as other minority groups and poor people have been. There is precious little data on long-term outcomes for sexual gender minorities and this is one of the priorities, I think, the research community needs to address. We are interested in poor health outcomes attributed, in part, to social disadvantage, but not exclusively, in these populations. For example, the fact that White men still have higher rates of heart attack is not of central interest to us. It is not a health

disparity — it is a difference. I think if you have any questions about that, I am happy to address them. Now, I mentioned "poor," and one of the challenges of the research is - how do you measure this? This is a list of how some different groups have done it. My own personal preference is to use education as a simple one-question item. It is imperfect, but it is fairly robust across the lifespan in adults, as opposed to income, which varies quite a bit. It usually comes down to wealth, which is very hard to measure, and few data sets actually have that. For example, two physicians, and one was first in his family to go to college and makes the same money as another physician, same education, who is a third-generation home owner in San Francisco where property prices have doubled every 10 years. So, you can see the difference there in wealth, even though with any survey, you would say they are exactly the same SES. I think we have done a better job of measuring race-ethnicity, and we do not do a very good job at addressing social class in any of our human research. If anyone raises the question of why bother, this slide [SLIDE 8] should convince anyone who is a scientist, or data-driven, or is a regular person who can look at bars ... so, if you are poor, you can see this "under $25,000," for household income which is a little bit above poverty ... but your mortality is three times more than if the household income is over $115,000, and you all know that $115,000 is not "wealthy". The median household income was reported at the highest ever at around $59,000. So, you can see even that gradient is double the mortality. SES is a powerful predictor of health. We have known this for a long, long time and I think this is something we need to measure in all our research. There are many other social determinants. [SLIDE 9] This is just a list of some determinants such as, immigrant generation, national origin, family background, geographic location, sexual orientation and gender identity.

At NIMHD, we think about mechanisms leading to health disparities and we have created these four topic areas for thinking about it. [SLIDE 10] We have a framework on our website. [SLIDE 11] A lot of the research has been here - individual behavior, so, lifestyle, beliefs, what happens ... focus on the individual differences. That is all good, but there is a lot of other stuff going on around racism and stress, adverse conditions, the adverse effects of childhood adversity (effects on adult health). Biological process, in the last 20 years, I would say has exploded with information to a degree that no one really can keep up with it. Yet, the social/behavioral population clinical scientists have to stay connected to this. Epigenetics and genetic differences that have gotten the most play, but also what happens with stress indicators and inflammatory markers, and telomere lengths. The basic scientists need clinical and population scientists to be there to ask the question - well, what about differences by race-ethnicity? What about differences by gender? What are the differences by socioeconomic status? The moment is now to get on that train. Similar to biology, the empirical evidence on the importance of the "built environment" or the "physical environment" has exploded

in the last 20 years. Now we see the importance of where you live - are the windows broken, is there trash on the street, is it safe to walk, do you have sidewalks - all of these are factors, that scientists have begun to incorporate into public health science and behavioral research. Mobile technology has made that even more interesting engaging the cultural environment. The question is - how do you blend all these together?

I also think NIMHD has an opportunity to study the clinical setting. At NIH, clinical research has gotten a lot of play, a lot of importance. We treat people with cancer with immunotherapy and save or prolong their lives in ways that were unimaginable 20 years ago. It is really compelling. There are big clinical cohort studies with a disease focus. But what goes on every day in an office or a practice or an emergency room or in a hospital has not been of great interest to any particular NIH institute. We want NIMHD research to be in that space ... focused around minority health and health disparities. I do think a lot of disparities can be ameliorated or generated in the clinical setting in the laboratory of doctor/clinician/patient communication. We put this framework on our website to express our interest and reflect the complexity, of the kind of research we are focused on. It is a guide or framework, not an explanatory or causal model.

Switching to the diversity in science, I think this is a central topic, hopefully very much at home in this conference. [SLIDE 12] We do need diverse clinicians. In 2015, somewhere around 12% of medical school graduates were either Latino or African American and if you add the American Indian and the Pacific Islanders, it just barely bumps it up a half percent. In the biomedical scientific workforce, it is worse. About 7% of principal investigators at NIH are either Black/African American or Latino/a/Hispanic, and Dr. Hannah Valantine, Chief Officer for Scientific Workforce Diversity has taken this on as a challenge. We absolutely need to have our populations engage in scientific research careers. This is data that is dated from fiscal 2015 on principal investigators by NIMHD by race-ethnicity. [SLIDE 13] These data are de-linked from the review; it is not something we even have when making funding decisions. This is all of NIH compared to NIMHD. We think NIMHD funding is one method for diversifying the workforce. I am really proud when we have principal investigators, like Consuelo Wilkins, who come from underrepresented minorities and lead in a state-of-the-art science.

On inclusion of diverse participants [SLIDE 14] - for a long time, people said, "We will be nice and have some people of color in our study." It really is not about being nice or being "politically correct," as some would argue. It is really about science. You don't know what you don't know, as they say in English. If you want to advance knowledge, you have to have diverse samples. There are many examples of where this has come

forth. If you have another drug approved for a condition that disproportionately affects minority groups and all the studies the FDA reviewed had a total of 5% minority, that should not be acceptable anymore. So, inclusion does require community engagement. The scientists have to go out and engage the community. The community has shown that they are willing to engage with the scientists and I think we need to meet each other in a common ground.

Inclusion of minorities in clinical studies is not just about social justice and common sense because 40% of the population, by 2020, will check one of those boxes that is not White. It is good science. I think anybody who has done research in communities of color knows that it is harder. [SLIDE 15] The people who end up doing it usually are committed or have a particular focus. It takes more resources. The one thing I think that is consistent in talking to all the investigators who have worked successfully in minority communities is you really need to have more face time with the community. That means going to the community, getting to know them, having them see you as a real person, and then engaging in conversation. You have to be willing to invest the time and take the risk. Now, we think minority scientists are not effective, but there is no data to support that. I would also like NIH studies to be more accountable, so that it is not just "check the box" when you submit a grant. In the four years I sat on an advisory council at NIA, I never saw much of any rejection of a grant or saying, "We have a worry about this grant because they didn't really say they were going to recruit any minorities." As long as they put a reason, it was appropriate. We are working on a system where we can update that and monitor inclusions, so if a scientist says, "I am going to recruit 35% minorities" in their proposal, and they get funded after one year, after two years we are able to monitor what they are actually doing, and that will be a step in the right direction. Ideally, we should have a reward for a scientist who does an extremely good job of having diverse samples. One thing that we need to combat is the myth that these barriers are insurmountable. Some people give up because it is harder, and they say, "Well, I've got to get my sample so I can't expend the extra effort to do this." This is minority enrollment at NIH in clinical research. [SLIDE 16] These are reports that have been done by our office and the Director's office. You can see it is not all that bad. Yet, these are cross-sectional samples. So, you can have one study that has 16,000 Latinos, like the Hispanic Community Health Study/Study on Latinos, and that will boost up the numbers, or the Jackson Heart Study or the Strong Heart Study, and it doesn't monitor recruitment by different conditions. We do need to continue to monitor this and make progress.

Important message for the scientists - not every study needs to be powered. [SLIDE 17] Everybody thinks, "I can't have enough African Americans in my study to be able to do a test of difference stratified by race-ethnicity." I say, "You don't need to do that - just make sure you have some." The reason for that is, unless epidemiology drives you to say, "We need a stratified sample," in which case you power the study to do this (like, let's say the Diabetes Prevention Study), but you won't ever find out if there are going to be big differences unless you have some people in the sample, and I think this is the message that investigators need to hear. Not every study needs to include all groups. Participants need to be somewhat more representative, and for us to see that there is a hint that there might be something going on in this particular condition, we should then do another study where it is powered by race-ethnicity.

These [SLIDE 18] are just a small list of some examples where we have seen big effects of race-ethnicity that have been observed and supported by empirical data. If you smoke cigarettes, the nicotine gets metabolized to cotinine, and if you are Mexican American, White or African American it has clearly shown that there are different cut-points of what defines a biochemical smoker, and this is actually a research tool. Similarly, a BMI screen of 23 works for Asian/Pacific Islanders for diabetes, because at a lower BMI they have higher metabolic risk. What has not been talked about very much is that actually for both Latinos and African Americans, the optimal BMI cut-point is probably 28 based on mortality data, but nobody is saying to screen at 28. Smoking intensity on lung cancer: the differential effects of if you smoke 20 cigarettes a day and you are African American, you are at a higher risk of developing lung cancer than if you are White or Latino or Japanese American and no one understands why that is. These are observations that need to be addressed. Alzheimer's disease mortality is different by race-ethnicity. A group at UCSF found a gene that protects Latinas from breast cancer and this gene is linked to American indigenous background, so women who had American indigenous mixture had a 40% lower chance of developing breast cancer if they had this gene and this gene was present in 15% of the women. Isn't that interesting? And we ask that question because the epidemiology for years has shown lower rates of breast cancer. Patients with diabetes in the Kaiser system have different rates of heart attack and end stage renal disease. Even though they all have diabetes and are all in the same system, so similar care, all of the minorities tended to have lower rates of heart attacks by about 20-30%, and all of the minorities have higher rates of chronic kidney disease by about double. We don't understand why that is. There are a number of pharmacogenomic studies, clopidogrel, which is Plavix, a first-generation antiplatelet drug for aspirin, doesn't work in Pacific Islanders very well. Over 50% did not respond to clopidogrel. We did not discover that until after the drug was marketed and marketed

heavily in Hawaii where a lot of the people are Pacific Islanders. Researchers at UCSF years ago showed that if you are Puerto Rican Latino, your response to inhaled beta agonist, or albuterol, was not as good. So, that may be a partial explanation as to why there is worse asthma in Puerto Rican Latinos as compared with Mexican American Latinos.

NIMHD has three functional branches: biological behavior sciences related to mechanisms and etiology (not basic science, community health and population science, and clinical and health services research [SLIDE 19]. Community health relates to the long history of funding we have had in community-based studies, CBPR included. And we are beginning to address the issues of - what do we do about population health? How can we leverage big data? I think that is going to be increasingly relevant. I mentioned before about the clinical and health services and the importance of that setting. Here are a couple of examples of studies we funded as examples of community-engaged research. [SLIDE 20] This is a center that we funded at Flint under the Health Disparities Research on Chronic Disease Prevention with Dr. Debra Furr-Holden as principal investigator. There was a tremendous amount of groundwork on collaboration, academic, community organizations, as well as political partners using CBPR approaches. The main research project was an evidence-based church challenge to promote physical activity derived from prior work, "The Heart and Soul Study," out of University of Michigan by Ken Resnicow, that is now expanding and deploying it in a slightly different format in Flint. There is a study in Miami to improve the health of South Florida's vulnerable populations through collaborative participatory research. [SLIDE 21] They are interested in community health workers, and I think these different levels of facilitators is where community engagement really has made a huge contribution to our understanding of how we can apply this. They want to screen for different health conditions, HIV, hepatitis C, colorectal cancer, and cervical cancer, in a nonclinical setting. They already did the study showing that women can do their self PAP test and it can be acceptable and effective in terms of valid results. This has also been done in some countries in Africa, Hepatitis C, and HIV, with on-site testing, and then colorectal cancer, of course, with fecal occult blood test.

Let me switch to talk about racism, a topic that no one likes to hear about. I think of racism and discrimination as a model of chronic stress. These are three dimensions that I put on the slide just sort of to illustrate the point [SLIDE 22]. So, most people talk about racism or discrimination in terms of the first one - interpersonal. You are followed in a store. Someone calls you a name. There are a variety of different circumstances where you feel that you walk in somewhere and everybody is looking at you like, "What are you

doing here - you don't belong here." That has been the most studied. There are good measures for that. Nancy Krieger, David Williams, and other have developed y reliable measures. Structural racism, on the other hand, is something that, until very recently, I kept asking myself, "Is this really a research construct or a system construct?" We held a scientific workshop last May to try and address this. I think we answered the question: It is both. We are not yet sure how we should measure it. This is an area where look to stimulate the scientific community to do more work in. There are historical cultural and institutional practices that have existed for many, many years that are important for us to deal with ... not denying them, not saying we have a solution, but acknowledging in our research that racism is internalized it in a way that it affects health through biological responses, some that we may or may not be able to measure yet. It may lead to epigenetic changes in their interactions with the environment. This is again another area that needs attention. A snapshot from the Kaiser Family Foundation asking a large sample of Americans, by race-ethnicity, as you can see there [SLIDE 23], "In the last 30 days, were you treated unfairly?" The first column percent is anywhere. So, 53% of African Americans say that something like this happened to them in the last month. So, this is not a rare event. Over 1/3 of Latinos experienced it. It may have different flavors in Latinos, but it is there. Then, 15%, you can say is the reference point for whites. The right column is in healthcare. So, even in our healthcare setting, our minority patients are more likely to perceive discrimination compared to their white counterparts.

This is, again, a list of some of the biological measures that minority health and health disparities research people are trying to address [SLIDE 24], but there is a long history of studies of cardiovascular reactivity related to anger and adverse events related to blood pressure changes and now people are going back to this and looking at this. This was done back in the 1980s by psychologists. Blood pressure, glucose homeostasis, hemoglobin A1c, lipids, the inflammatory axis, including cortisol, which has not been around since I was in medical school, and then grouped together as allostatic load. I mentioned telomere length and sleep as othet important outcome and predictor. Some of what we see in adverse health events are the effects of poor sleep. Sleep is an important biological function of our human body. As one of my colleagues at NIH says, it is when you wash your brain.

As I wind down, I just want to show you some data of things that I think are encouraging in some ways. There is a lot of play about decreased life expectancy in the U.S. a couple of years ago. [SLIDE 25] All of that decrease was from poor Whites. Blacks have actually improved at an unexpectedly high rate. So, in the years

2000 to 2014, African American life expectancy increased by 4% annually when the projects were 2%. That is what you expected if society continued to progress in the right direction. Latinos continued to defy any rational explanation. They have the longest life expectancy despite adverse socioeconomic status, and this is now very consistent. No one argues about the data not being accurate. American Indian, Alaskan Native, and Native Hawaiian data is not published by CDC yet. This was presented by one of the Census scientists, but it is really startling how low it is.

This is bearing down on African American mortality. [SLIDE 26] You can see that for older people, over 65, there is actually a crossover, and actually African Americans do a little bit better in mortality than their white counterparts after 65. It is in this midlife (50-64) that we see a persistent gap that hasn't narrowed as much, and it is an area of opportunity. Then, most recently, stroke deaths were reported. [SLIDE 27] Well, we know what to do with stroke. We know that you have to control blood pressure. We know you have to control vascular risk and not have excess alcohol. We have also gotten better at treating. We have gotten better at identifying early signs of cerebrovascular insufficiency so that we put people on appropriate therapy early rather than waiting for people to actually have a stroke and have a deficit. You can see that progress is in all groups, despite the fact that disparities persist. The last bit of data shows what we have known, that income inequality leads to higher deaths. [SLIDE 28] We see this gap of life expectancy for women and men based on the poorest 1% compared to the richest 1%. It is an extreme example, but we have known this, and that gap is getting worse is their analysis. What was not highlighted by the media as much is the fact that if you took the bottom quintile in some areas compared to the bottom quintile in other areas, people in some areas actually live three to four years longer than in others. What are those communities doing right to allow their most vulnerable to actually do better? I think that is a question for us as scientists and you as the community leaders who are here.

Then, some opinions: I think if we look at policies to reduce health disparities in the healthcare setting [SLIDE 29]: Expanding access. We have seen what has happened in the last three to four years with the expansion of insurance. We have cut the proportion of uninsured in half. We will see what happens with health if we allow this experiment to go forward. The areas that we have public health consensus. We have achieved childhood immunization rates well over 90% in all race-ethnic groups in the U.S. In fact, the group that is lagging behind now appears to be the white middle class for reasons that are unrelated to access. The coordination of care, I think, is a central component of healthcare settings. People still want "doctors" to see and consult with, but

clinicians can't function alone. They need a system. They need support. They need medical assistants, nurses, nutritionists, psychologists, mental health clinicians. I believe the patient-centered medical home is an effective way of creating this with information systems. It is good primary care with good information systems. Then, it facilitates addressing issues around communication and cultural competence as well. Performance measurements are controversial. This is where you say, "We will give you more money if you do above this metric in these 5 or 6 or 10 different things." The threat there is the clinicians who are most committed to taking care of the most vulnerable populations could do much worse, and it could be a mechanism of redistributing resources to the more elite institutions.

So, what do we need to do? I start by saying that we need to shift to population health. I don't care if the system is Kaiser or a University or a private model. If they say, "Well, this is your population - figure out how to take care of them," and you have to be responsible for a spectrum, instead of just taking care of the people who are well and wealthy, I think that is an approach that can work. This is what the Accountable Care Organization ideal was when the ACA was implemented. I think this is a model that can work to decrease disparities as well. Then, engage community resources in promoting health. Usually we talk about nutrition, physical space, and tobacco, and I already talked about discrimination.

Last, in late June, I was part of a National Heart, Lung, and Blood Institute-funded workshop on engaging community research to reduce cardiovascular disparities. Dr. George Mensah, who runs the Implementation Science Program, organized it and brought scientists together. He produced a manuscript that is being reviewed and I borrowed some of the lessons from the tables he had there on what the group concluded. [SLIDE 30] You learn to use population data to identify hot spots and people have begun to do this. The other approach is to target the most vulnerable. [SLIDE 31] So, you are in a health system. You can have standard procedures for everybody, but you then want to target the most vulnerable people. Sometimes they are the elderly, the minorities, or the poor, whatever way you are using to measure that. You've got to address trust/mistrust of individuals and most professionals a lot of times get very defensive around us and their defensiveness is that they are taking it personally, like you don't trust me as the clinician. Recognize the good things, the resilience, that exists in communities. I think this is really important. It is not all about how bad we are. There are many good things that happen in communities that actually have some cohesion. Researchers really need to be aware of, know, and learn from communities, and the only way to do that is to actually go to the community and employ the members, engage the leaders, and create

partnerships. [SLIDE 32] I keep coming back to building this trust. I think we have to be out there. We have to be in the community and not just thinking about the research or the progress that we are trying to make. There are many ways of doing it - providing resources and training, funding opportunities (this conference is a good example), engaging ... the first time I saw this at UCLA was doing pilot studies for research in minority aging and they had community members actually review the grant application and work with the investigator to actually figure out how to make the grant relevant to the community. You could say that limits some of the research questions they ask. Perhaps. But it does actually work both ways. People can then find that if you do a secondary data analysis, what does this mean for the community you are working on, and I think it would avoid problems. All of our centers have Advisory Boards that facilitate them two-way exchanges, not just, "Oh, we have got to have a meeting." This is a vehicle by which you can bring people together. I am a real supporter of Advisory Boards that have both community and academic members in it. Then, using evidence-based interventions and figuring out always how things can be sustainable. I am going to just put this up and not dwell on it - training the next generation. [SLIDE 33] We know there is a tremendous amount of effort there. I want to end with a thank you and I think we have a little bit of time for questions. [SLIDE 34, 35]

Question: Research faculty is one role that needs diversity, but there are also research coordinators, research project managers, other staff positions within research teams, and I wondered if your institute or other NIH institutes have programs that are addressing workforce pipeline for full research team, not just faculty.

Answer: Great question. I don't think our institute is doing that at all. I think when I was at UCSF, we saw that as the responsibility of the institution and the academic units. So, the cancer center would do that and the CTSI would do that within the institution. But it is really an important point. I don't even know the data on the diversity of that workforce, research coordinators. I know that there is very little diversity in clinical trial coordinators, but in community outreach workers, people do go to the communities and hire people from those communities to do that work. But I think that is a great point.

Question: You made mention of "class," and I struggle sometimes with how to categorize class, how people are thinking about that. I have recently heard the term precariat, like people are in a "precarious" place. Would you have any terms for how you would start thinking about class?

Answer: It is a sociology term and I am not a sociologist, so I would be pretentious to say that I could give you all the definitions, but it clearly comes out of the European way of thinking and from sociology. The United States, for decades, if not still today, was the land of opportunity where you could always move up if you had the opportunity, unlike let's say, England, where you were born into a specific social strata, and that is where you were. You were working class, or you were royalty, two extremes. So, I use it interchangeably with socioeconomic status, if that is a more palatable term here. As I said, it is defined by income and education, most globally, although wealth is really the bottom line. In mobile populations, you will see variation across time ... just like the example I gave you about the two physicians, also, immigrants are mobile. They often come here with a higher level of education and frequently have a downward mobility in their socioeconomic status in this country. Sometimes they stay the same or go up, but that is not uncommon. So, this whole issue of how social movement, especially among immigrants ... they use the term segmented assimilation of how individuals move across that. I think that is a better explanatory model than just talking about acculturation, for example. But I use that term. It may be, in and of itself, a statement, and it is sort of like trying to make sure people don't think, oh, we live in a classless society and everyone can make it if they get the right opportunity, and try not to assume that the audience believes that, because it isn't true. The data area really quite the opposite despite all of the progress that we've made. I don't know if that helps.

Declarations

- The presenters have no financial competing interests related to this presentation. Both Dr. Pérez-Stable and Dr. Sufian are employees of the NIMHD, whose award helped support this conference.
- All authors have approved the manuscript for submission
- The content of this presentation and transcript has not been published or submitted for publication elsewhere.
- This presentation was organized by the Meharry-Vanderbilt Alliance with support from NCATS and the NIMHD of the National Institutes of Health under award number R13TR001694

Community Engagement in Minority Health Research to Reduce Disparities

Eliseo J. Pérez-Stable, M.D.,
Director, National Institute on Minority Health and Health Disparities

eliseo.perez-stable@nih.gov

Advancing the Science of Community Engaged Research Conference

September 14, 2017

DEPARTMENT OF HEALTH & HUMAN SERVICES-USA
NIH National Institute on Minority Health and Health Disparities

NIMHD History

1990	2000	2010	2014	2015	2017
Established as an Office under the NIH Director through DHHS Secretary Louis W. Sullivan, M.D. in 1990	Transitioned to a Center through legislation championed by Representative Louis Stokes (D-OH) in 2000	Patient Protection and Affordable Care Act contained language championed by Senator Ben Cardin (D-MD) to transition to an Institute in 2010	John Ruffin, Ph.D. led all the entities until his retirement in March 2014; Yvonne T. Maddox, Ph.D. became Acting Director	Eliseo J. Pérez-Stable, M.D., started September 1, 2015	FY 2017 budget is $289 million

NIMHD Mission

- Support research that advances understanding and improvement of health and disease in minority racial/ethnic groups
- Support research to understand the causes of and define mechanisms leading to interventions that reduce health disparities in specific populations
- Supports the training and development of a diverse scientific workforce as part of broad NIH mandate

Minority Health Definition

- Minority Health Research focuses on health determinants that lead to specific outcomes within a minority group and in comparison to others
- Race and ethnic minorities share a social disadvantage and/or are subject to discrimination as a common theme

OMB Race/Ethnicity Classification

- **African American or Black**
- **Asian (>20 countries)**
- **American Indian and Alaska Native**
- **Native Hawaiian and other Pacific Islander**
- **Latino or Hispanic (20 countries)**
- **White (Middle Eastern/North African)**

Health Disparity Populations

– Health disparity populations include:
- racial/ethnic minorities
- less privileged socio-economic status
- underserved rural residents, and/or
- sexual gender minorities

– Populations have poorer health outcomes often attributed in part to social disadvantage, being subject to discrimination, and underserved in the full spectrum of health care.

Assessment of Socioeconomic Status or Social Class in Clinical Medicine

- Education – in years or categories
- Income – annual household/dependents
- Occupation categories – Whitehall
- Life course SES — effects understudied
- Parental education (children)
- Type of insurance
- Wealth or total assets

Relative risk of All-Cause Mortality by US Annual Household Income Level

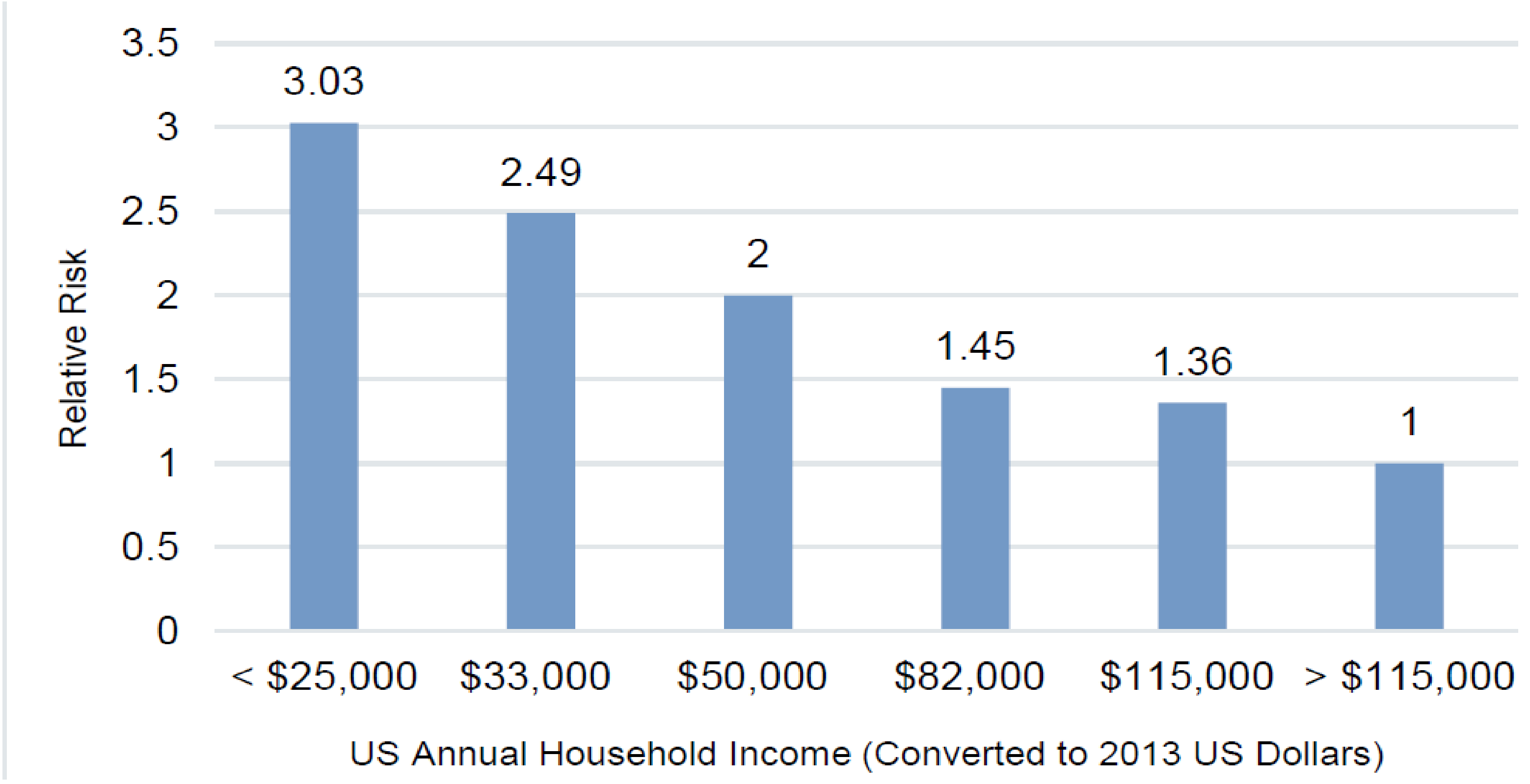

Other Social Determinants

- National origin/background
- Urban/rural or geographic region
- Cultural identity, Religion
- Immigrant generation, documentation
- Language proficiency, acculturation
- Literacy, numeracy, food insecurity
- Sexual orientation, gender identity
- Disability status

Mechanisms Leading to Health Disparities

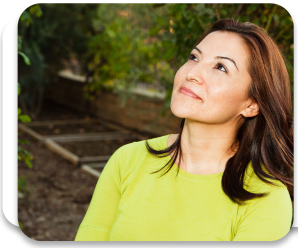

Individual Behaviors, Lifestyle, Beliefs and Response to chronic Stress: racism, adverse conditions, food insecurity, witness to violence, immigrant, limited English proficiency

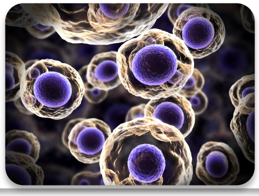

Biological Processes and Genetics: earlier age of onset, gene variant, metabolic differences, susceptibility, faster progression or greater severity

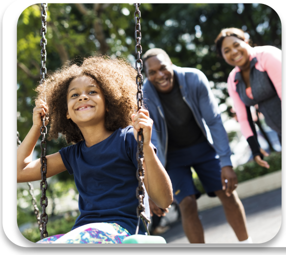

Physical and Cultural Environment: place, social system, neighborhood, infrastructure, family, social interactions, network, community cohesion

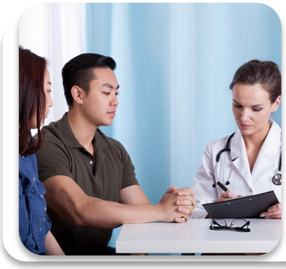

Clinical Events and Health Care: differential treatments, poor communication, adverse events to medications, falls, progression of disease, access, use/abuse of appropriate services, end of life care

Health Disparity Populations: Race/Ethnicity, Low SES, Rural, Sexual/Gender Minority
Other Fundamental Characteristics: Sex/Gender, Disability, Geographic Region

Domains of Influence	Levels of Influence			
	**Individual**	**Interpersonal**	**Community**	**Societal**
**Biological**	Biological Vulnerability and Mechanisms	Caregiver-Child Interaction<br>Family Microbiome	Community Illness Exposure<br>Herd Immunity	Sanitation<br>Immunization<br>Pathogen exposure
**Behavioral**	Health Behaviors<br>Coping Strategies	Family Functioning<br>School/Work Functioning	Community Functioning	Policies and Laws
**Physical/ Built Environment**	Personal Environment	Household Environment<br>School/ Work Environment	Community Environment<br>Community Resources	Societal Structure
**Sociocultural Environment**	Sociodemographic<br>Limited English<br>Cultural Identity<br>Response to Discrimination	Social Networks<br>Family/Peer Norms<br>Interpersonal Discrimination	Community Norms<br>Local Structural Discrimination	Societal Norms<br>Societal Structural Discrimination
**Healthcare System**	Insurance Coverage<br>Health Literacy<br>Treatment Preferences	Patient-Clinician Relationship<br>Medical Decision-Making	Availability of Health Services<br>Safety Net Services	Quality of Care<br>Healthcare Policies
**Health Outcomes**	**Individual Health**	**Family/ Organizational Health**	**Community Health**	**Population Health**

Lifecourse

Diversity in Science and Medicine is a Demographic Mandate

- **Develop a diverse clinical workforce that will care for our patients**
- **Develop a diverse biomedical scientific workforce that will conduct biomedical research in all areas of science**
- **Engage under-represented populations to participate in clinical research and Precision Medicine Initiative**

Comparison of Racial and Ethnic Categories of Principal Investigators, NIMHD and NIH, FY 2015

	NIMHD	All NIH
African American or Black	19.6%	2.2%
AI, AN, NH or OPI	2.8%	0.3%
Asian	9.5%	17.7%
White	50.8%	68.6%
Multi-Race	0.0%	1.3%
Unknown	9.3%	5.1%
Withheld	6.4%	4.9%
Latino or Hispanic	12.9%	4.7%

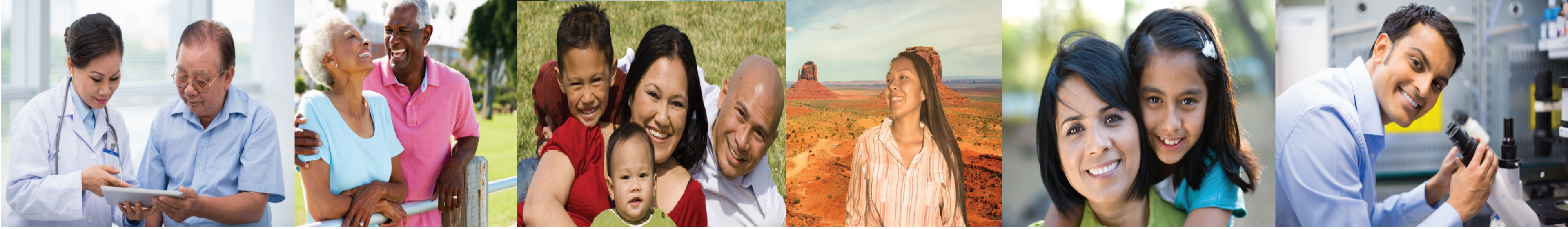

Inclusion of Diverse Participants

- **Disparity populations are underrepresented in biomedical research**
- **Inclusion of minorities in clinical studies is an important for advancing knowledge**
- **Inclusion will require community engagement**
- **Social justice, good science, and common sense mandate inclusion (40% US population)**

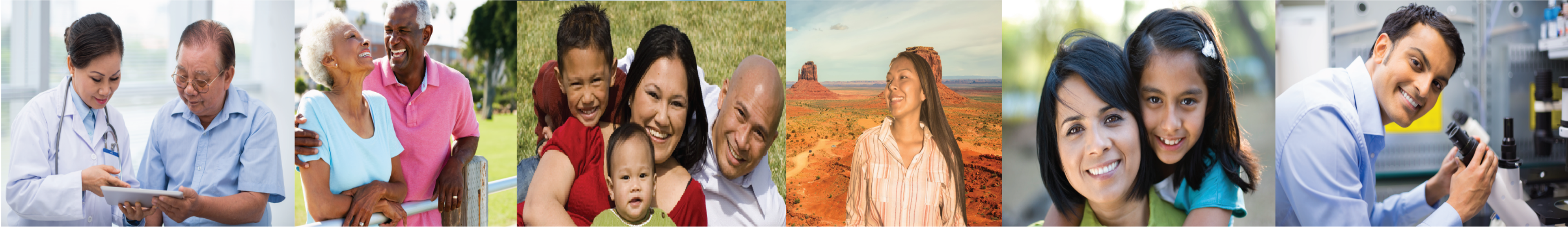

We Have to be at The Table

- Yes, it is harder to recruit minorities and it usually takes more resources and other skills
- More face time and personal messages needed
- Minority scientists are probably more effective
- There is a need to implement accountability on minority recruitment — not just “check the box”
- End myths that barriers are insurmountable

NIH National Institute on Minority Health and Health Disparities

Minority Enrollment in NIH Clinical Research

Race/Ethnicity	2010	2014
All Minorities	28.1%	26.2%
White	70.1%	68.3%
Black	11.9%	11.1%
AI/AN	0.8%	0.7%
Asian	6.6%	5.1%
Latino	7.8%	8.3%
NH/PI	0.7%	0.4%
Multi-race	1.6%	1.3%
Unknown	8.3%	13.1%
Sample size	21,523,076	25,209,874

Importance of Inclusion in Trials

- Not every study needs to be powered to examine racial/ethnic differences
- No every study needs to include all groups
- Participants should be more representative of the population to provide meaningful opportunities to examine the complex relationships
- Understanding these interactions can extend our understanding of disease pathology and expand therapeutic options for all

Clinical Examples Where Race/Ethnicity Matters in Understanding Health and Disease

- Optimal Cotinine cut points to define smoking
- Optimal BMI cut points to define risk
- Effect of smoking intensity on lung cancer
- Alzheimer's Disease mortality
- Gene protects Latinas from breast cancer
- Diabetes results in different rates of heart attacks and ESRD
- Clopidogrel, APOL 1, response to albuterol

NIMHD Areas of Research

- **Integrative Biological and Behavioral Sciences**: Focus on mechanisms and etiology (not basic science)
- **Community Health and Population Science**: Leverage track record in Community-Engaged research and expand to Population Health
- **Clinical and Health Services Research**: What happens in the clinical setting unrelated to a specific disease

Flint Center for Health Equity Solutions

- **Health Disparities Research on Chronic Disease Prevention**
- **Active collaborations with academic, community organization, national, and political partners using CBPR**
- **Church Challenge to promote physical activity using evidence-based model from Heart and Soul**

Debra Furr-Holden, PhD, PI, U Michigan, U54MD011227

CHW-Led Multi-Modality Program: The South Florida Center for Reducing Health Disparities

- **To improve the health of South Florida's most vulnerable populations through collaborative, participatory research**
- **Test the effectiveness of a Community Health Worker-led intervention**
- **Screening for 4 health conditions: HIV, Hepatitis C, colorectal cancer, and cervical cancer in a non-clinical setting**
- **Pragmatic Trial**

Olveen Carrasquillo, MD, PI, U Miami, U01MD0101614

Racism and Discrimination

- *Interpersonal*: Most work done, good measures developed, associations established, most common
- *Structural*: History, culture, institutions, and codified practices that perpetuate inequity; research or systems construct?
- *Internalized*: How discrimination (as above) effects individuals who are not aware or sublimate; accept cultural or biological inferiority

Perception of Unfair Treatment: 2015

In past 30 days were you treated unfairly because of racial or ethnic background in store, work, entertainment place, dealing with police, or getting healthcare?	Percent agree
Latinos	36% / 14%
African Americans	53% / 12%
Whites	15% / 5%

Kaiser Family Foundation Survey of Americans on Race, November 2015.

Biological Measures in MH/HD Research

- Physiological measures that focus on metabolic pathways: SBP, A1C, lipids
- Cortisol and HPA Axis: saliva, hair
- Inflammation: CRP, IL-6
- Allostatic load: groups 8-14 measures
- Telomere length in white cells
- Sleep quantity and quality
- Brain imaging?

Life Expectancy in the U.S., 2014

	Men	Women
Whites	76.5	81.1
Blacks	72.0	78.1
Latinos	79.2	84.0
AI/AN and NH (2007-09)	68.0	74.3

Arias E, NCHS, CDC, 2016

All-Cause Mortality: Whites and Blacks

FIGURE 1. Death rates among blacks and whites, by age group (years) — United States, 1999–2015

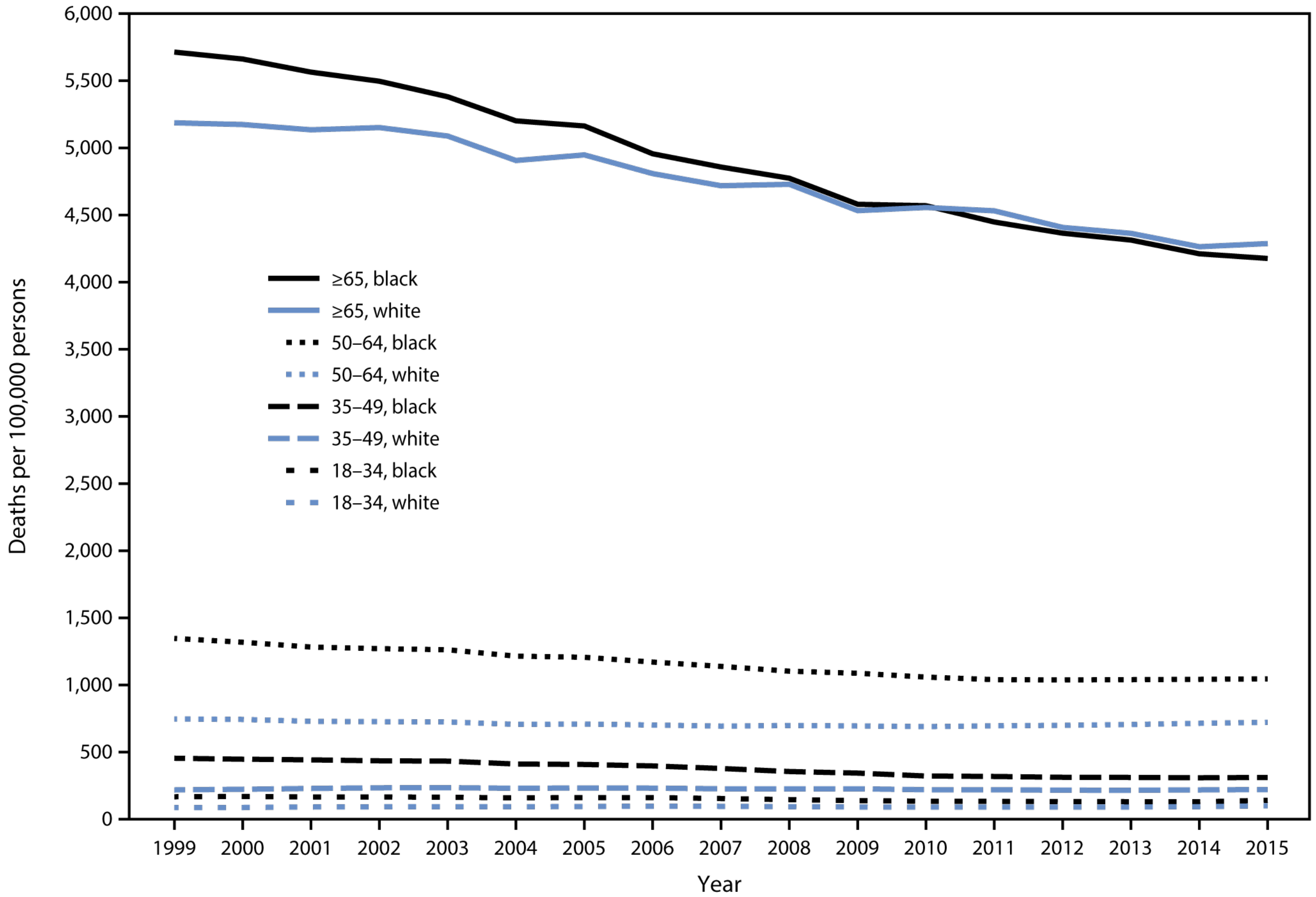

Cunningham TJ, et al MMWR 2017; 66:444-456

Trends in Stroke Death Rates

Age-standardized Rates, 2000-2015, age ≥ 35 y

MMWR, September 6, 2017, 66: 1-7

	2000	2015
Whites	115.2	71.3
Blacks	161.1	102.0
Asians/PI	103.3	58.5
AI/AN	97.2	62.1
Latinos	89.7	62.5

Place, Income and Life Expectancy

- Tax records for pretax household income and death registry in SSA
- Richest vs. poorest 1% live on average: 10.1 y (women) and 14.6 y (men) longer
- Inequality in 5% increased by 2 y for men and 3 y women over time
- Bottom quintile in some local areas live an average 4.5 years longer than others

Chetty R, et al, JAMA. Online April 10, 2016

Policy Strategies to Reduce Health Care Disparities

- Expand Access: Health insurance, place and clinician as fundamental
- Public Health Consensus
- Coordination of Care: Systems, navigators, and target conditions
- Patient-Centered: PCMH, effective communication, cultural competence
- Performance measurement: Risk

Community Engaged Research to Reduce Health Disparities: What is Needed?

- Shift models of care to population health with accurate demographic and social determinants of health
- Enhance access to health care services: portal for patients, e-referrals, tele-medicine
- Address access to real food and safe places
- Engage community resources in promoting health: nutrition, physical space, tobacco
- Recognize and manage discrimination

High Burden Communities

- Use population data to identify hot spots
- Target most vulnerable by demographics
- Recognize and Re-enforce resilience
- Address trust/mistrust, hopelessness
- Language and cultural variation
- Researchers need to be aware of, know and learn from communities
- Employ members, engage leaders, partnerships

Engage Community Stakeholders

- Build trust
- Cultural humility
- Provide resources, training, funding opportunities
- Engage community members in pilot project grant reviews
- Advisory boards
- Evidence-based interventions
- Sustainability

Training the Next Generation of Researchers for Health Equity: What is Needed?

- Provide opportunities and create long-term mentoring plan
- Rigorous scientific base and training
- Network with established scientists
- Multi-disciplinary teams essential
- Nurture and support emerging scientists through all available mechanisms
- Say No, but learn to say Yes
- Manage bias, minority tax, and rejections

Thanks!

Questions

Comments

Connect With Us

Visit us online
www.nimhd.nih.gov

Connect with us on Facebook
www.facebook.com/NIMHD

Follow us on Twitter
@NIMHD

From the Frontline: What Does Precision Medicine Mean for Me?

Usha Menon

umenon@health.usf.edu

Associate Dean for Research & Global Advances, University of Arizona College of Nursing

Tucson, Arizona

Abstract

Dr. Menon discusses the importance of engagement to the NIH's All of Us project, a multi-year study that intends to recruit 1 million participants that, as a group, represent the diversity in the United States. She discusses how engagement cannot be effective if it stops with outreach, because that is unidirectional. Finally, she explains the difference between equality and equity, and discusses how a focus on equity can keep this large project from creating more health disparity than already exists.

[*Dr. Menon*] I am going to start with this old Vulcan greeting. If you are a Star Trek fan, you might recognize this. [Slide 1] Greetings. I am pleased to see we are different. May we become greater than the sum of the both of us [Slide 2]. I think that is the important thing about meetings like this. They bring us together and it is not just race/ethnicity. It is not just the color of your skin. It is not just the accent that I speak with. It is about the diversity of thought and scholarship. From the frontlines, what does precision medicine mean for me? For the past year and a half, myself and my family have been helping to build a plane as we flew it. That is called the All of Us Research Program funded by the National Institute of Health. The basic premise of where we start from is that of all forms of inequality, injustice in healthcare is the most shocking and inhumane [Slide 3]. It is critical as you build your team to think about people who feel and think like you and have the same philosophy [Slide 4]. Often, they have you sort of think about, "Hey, is everybody in the boat rowing in the same direction?" How do you get teamwork going? Maybe you need to ask - is the right person in the boat with you? Never mind about rowing in the right direction, but is the right person in the boat with you? This is a snapshot of what is going to be one program of a million+ people, so many, many, many faces and so many different ways to engage the community. What is engagement for us? it is education, consultation, communication, extension, partnership, participation. All of it is engagement at some level [Slide 5]. What we are discovering as a team is that it is not just about the community external to us, but we have a 90+ person team between the University and Banner Health and that is our community. We have to engage them and make sure we are all going the same direction [Slide 6]. There is a difference between equality and equity. Engagement for us is not just outreach, so we don't use that word because it seems very

unidirectional. It seems like, "Oh, I am reaching out to you," whereas what we really want to do is make it a bidirectional conversation. We are all about fostering the norm of inclusion [Slide 7].

What does precision medicine mean for me? This is what we have heard from our community constituents, from our patients, but also from the 67-something groups that Dr. Wilkins, herself, lead during the initial inception of this program. People said they wanted to be included, that they wanted their voices included at levels beyond just putting them on an advisory board and asking their opinion every three to four months. It is a planned effort. We are reorienting toward maximizing diversity through targeted inclusion, reaching hard-to-reach populations, and using very thoughtful inclusion and exclusion criteria [Slide 8]. At the University of Arizona, we have something called the Arizona Board of Regents Tribal Consultation Policy. It has governed every piece of engagement my team and I do with the American Indian community. When you think about fostering inclusion but working within the constraints of what your Board of Regents put forth for you, it does make it very difficult. A consultation before ... I can communicate with the tribes about any kind of research, bringing all 22 tribes to the table to have a conversation. It is very systematic for us. It reflects the needs and preferences and priorities. And it is inclusive, broadly, of diversity. It provides the opportunity to interact with our potential participants. We do not call it recruitment because recruitment [Slide 9]. is a separate step. Recruitment is when you have actually brought that person in and you have gotten informed consent. We are talking about raising awareness and engaging individuals to tell us how we should conduct ourselves in the community. Do we have setbacks? Yes. Lewis Grizzard is a great southern author [Slide 10]. He has the best book titles - Elvis is Dead, and I Don't Feel So Good Myself. It happens. There are setbacks. Have I messed up? Absolutely. It is that humility that you walk into the community with. Has my team messed up? Absolutely. But we learn from those mistakes and go back to the community and we apologize. Is there a formula? I don't think so. Jack Handy had "Deep Thoughts" ... "I wish I had a Kryptonite cross because then you could keep both Dracula and Superman away [Slide 11]". These are the formulas we think about. I understand cultural competency and there is a checklist and I am going to make my team go through a checklist, and low and behold, I am going to unleash them on the world and mess it up with the rest of everybody coming behind because we didn't stop to think about the fact that there isn't a formula. As a previous speaker mentioned, just look at Asians. I am South Asian and often I get lumped in with another group. My culture is very different from other parts of Asia. How and where does culture fit into engagement? [Slide 12] That is the key piece I want to talk to you about in terms of our precision medicine with all of our research program strategies. So, the first is using visual strategies - colors, images,

graphics. This is important. People want to see people who look like themselves. They want to hear the same kind of languaging. Here is a program brochure for the All of Us program that we are adopting for Alaska [Slide 13]. When I got to the Alaska Native Health Research Conference and the Alaska Federation of Nations ... now, if that isn't the cutest little picture, we went through a lot of vetting with some cultural brokers in order for me to even show you this. These are the images people would like to see, celebrating them and celebrating their culture. Evidential strategy [Slide 14]. Enhance the perceived relevance of the health issue. For us, it is not one health issue because we are talking about precision health. This million+ cohort is going to generate hundreds of studies on hundreds of issues and many diseases and health conditions. We can make it relevant when talking to individuals [Slide 15]. If I were speaking to Asian Americans, I might say, "Listen, suicide is the eighth leading cause of death for Asian Americans whereas it is the 11th leading cause for all other racial/ethnic groups." You make the issue relevant to that community. Here is something I used at the National Association of Hispanic Nurses Conference because I wanted to really talk about [Slide 16]... I did not need to preach to them about the disparities that still exist with Latinos, right? What I needed to say was, "Here is the problem - less than 1% are actually enrolled in clinical trials." Linguistic strategies [Slide 17]. Language ... of course, translating into the language of choice for that individual, but there is also the vernacular. There are also idioms that people use within their culture and it may or may not be okay for me to say some of them. It may or may not be okay for some of them to say them to me. Let's look at this [Slide 18]. Does anybody know where this came from - "The Vodka was good, but the steak was bad." This came from an English phrase that was translated into Latin and simply back-translated. "The Spirit is willing, but the flesh is weak." Do you see how you lose the intent if you follow a formula? It was actually our methodologist that taught me this. I have never used that translation in any of my studies because we use a committee-based translation so that we get the perspectives of multiple people. We are going to content equivalent, not linguistic equivalent. Humor [Slide 19]. This may or may not be funny to some of you, yes? You will be happy to know that race played no part in this decision. It may not be appropriate for me to use with all audiences. Involving those constituents, health workers and community members [Slide 20] ... I want to show you an example of something we are doing [Slide 21]. I don't know if you can see it very well, but it is the picture of the gentleman, the poster on the wall ... this is the Banner Health Hospital cafeteria in Tucson. We are still in beta phase, so we are not allowed to do a lot of external communications. We are still doing this within our group. What we did is we put out a call and asked what we call our "friendlies," the initial first 800 or so who are in the program, whether they would like to give testimonial. They agreed with their pictures. So, if you go into the cafeteria, you see someone who might be working in your department. You

see a poster. You see them say, "Precision medicine is really important." Then, you think, "Maybe it is important for me to do this." So, they are all over most of the cafeterias now. Remember I talked about taking into account the needs and preferences of the community? We have a retention newsletter [Slide 22] and this goes out ... if you are one of the million enrolled, you get this newsletter. The first element that you see came from community needs assessments already done by the local library. We didn't recreate the wheel. People are tired of being surveyed, so, we used existing needs assessments rather than gathering data with new surveys. We had a couple of grad students go through and figure out what the top utterances were and then focused our retention newsletter on the needs the community told us they had. We are trying to be respectful and give back. Sociocultural strategy [Slide 23]. This is recognizing a group's cultural values, beliefs, and behaviors, and understanding that they should be recognized, reinforced, and leveraged to give context rather than to assume that there is a formula.

Let's look at some of these. This is a very classic picture of missed expectations [Slide 24], which can lead to discord. I am a little over half a century now, but I have also lived over half my life in the United States. When I go back and engage with my family, there is a distinct difference because they truly feel I am not just acculturated - I have been assimilated. This is about taking on [Slide 25] the culture of the place you are. We are into Wilbur the Wildcat County and that is the mascot for the University of Arizona, we have these paw prints for the All of Us program. That is actually a picture of my truck that has this on it and I drive around town with it. Not only does it say, "Hey, look, I am recognizing something that is really important in Wildcat Country," but I am also saying, "I believe in it so much that I am willing to put this on my truck and drive around with it." It is really all about perspective [Slide 26]. You can be as culturally salient, sensitive, and appropriate as you can be, but if the person receiving the message doesn't perceive that, then it is not worth anything. Depending on where you are, the Australia or the U.S. [Slide 27], you may be down under or top up - it just depends on where you are looking at things from. It is hard work. What I say to my team is, "The impossible could not have happened, and therefore, the impossible must be possible in spite of appearances [Slide 28]." It is not about making the same kind of brochure to go out to everybody. It is about making sure people get what they need in order to be successful [Slide 29]. It comes to equality versus equity [Slide 30]. Equality means you give the same size box to everybody and think you've created fairness, but you haven't. If you look at the next picture, that is true equity, because you gave these individuals the size of box they needed in order to watch the baseball game. That was success for them. Who is accountable? All of us. Every single one of us in this room is accountable and I hope you will take this message back with

you. Jack Handy says, [Slide 31] "If you ever crawl inside an old hollow log and go to sleep, and while you are in there some guys come and seal up both ends and put it in a truck and take it to another city, boy, I don't know what to tell you." It is your responsibility to make sure that people don't try to put a formula into engagement, that you follow the appropriate strategies. When is the time to act? [Slide 32] Here is Lewis Grizzard - "When My Love Comes Back from the Bathroom, Will I Be Too Old to Care?" We do not have time to waste. Every single day I do not do the right thing in this community is two steps back for this national program and my university. If we don't have the answer, we say we don't have it. Who is watching? [Slide 33] Every single person. "Don't Bend Over in the Garden, Granny, You Know Them Taters Got Eyes." These are the titles I was telling you about. But every single time we go out and talk about research, and we don't do what is appropriate for that group who is giving us their time and their trust, somebody is watching. You are setting a precedent, not just for yourself, right, but you are setting a precedent for those people coming behind you as well. I believe if you can dream it, you can make it happen [Slide 34]. My team is all about making this happen in the absolutely appropriate way and we have pushed back against leadership and said, "We will not do that," or "We will not go to that meeting because we do not have the appropriate materials," or, "We will not go engage x, y, z community because we don't have anything to show them that is appropriate and sensitive to them." Coming back, I think the key message I wanted to talk with you about in terms of engagement is not to think about equality but to really think about equity, [Slide 35] that through equity you don't create more disparity. Precision health is an amazing opportunity. It is going to change the future of healthcare. It is going to change the future of medicine. Whether we like it or not, it is coming. It is already here. How can we not create more disparity through this opportunity, but actually create equity?

<u>Declarations</u>

- ☐ The presenter has no financial competing interests related to this presentation.
- ☐ All authors have approved the manuscript for submission
- ☐ The content of this presentation and transcript has not been published or submitted for publication elsewhere.
- ☐ This presentation was organized by the Meharry-Vanderbilt Alliance with support from NCATS and the NIMHD of the National Institutes of Health under award number R13TR001694

Greetings! I am pleased to see that we are different. May we become greater than the sum of both of us.

Vulcan Greeting

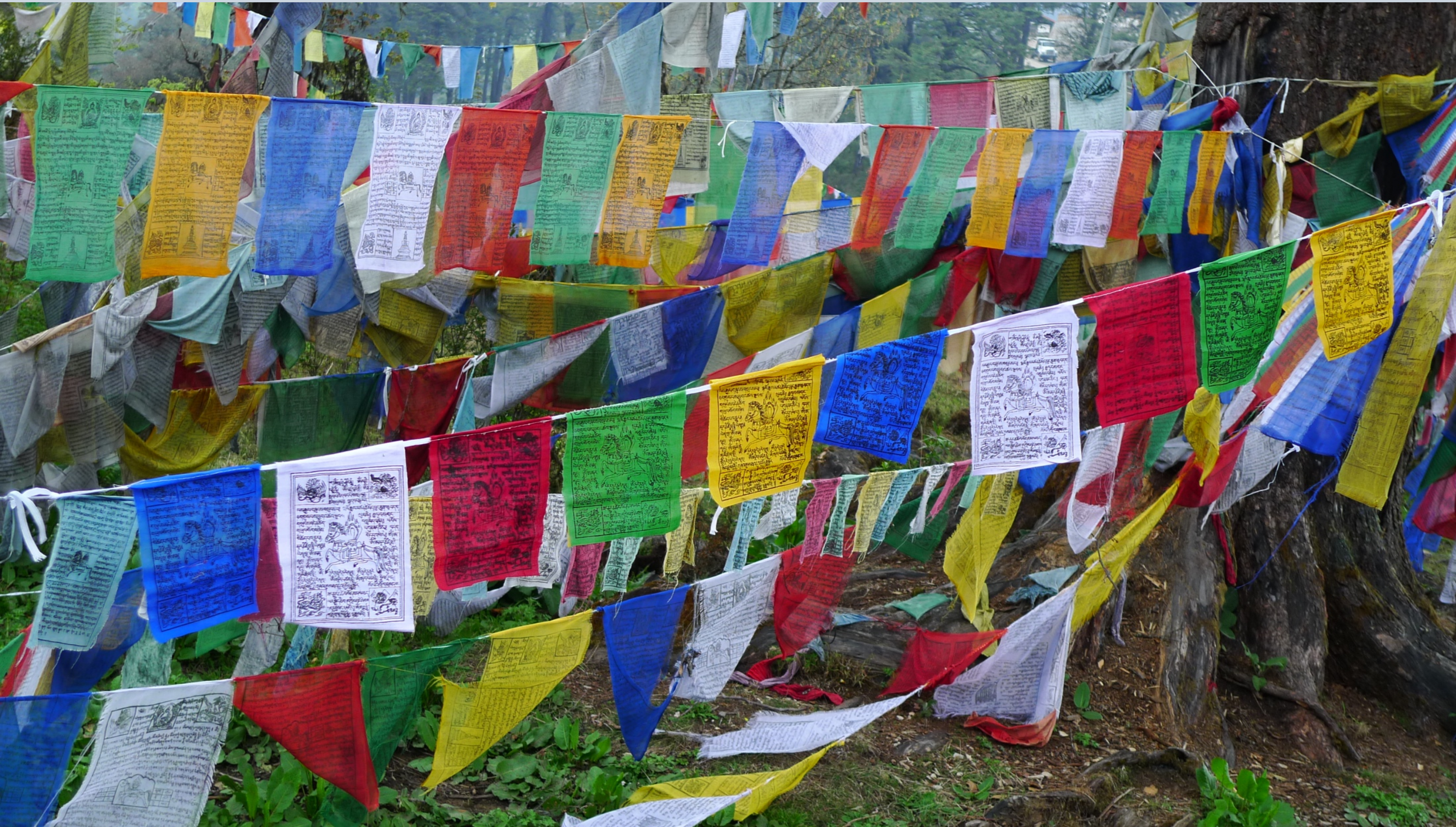

From the Frontlines: What Does Precision Medicine Mean for Me?

Usha Menon, PhD, RN, FAAN
Professor;
Associate Dean, Research and Global Advances
Co-Principal Investigator: *All of Us* Research Program at UA-Banner Health

- Of all the forms of inequality, injustice in health care is the most shocking and inhumane.

~Martin Luther King, Jr.

All of Us RESEARCH PROGRAM | The Future of Health Begins With You

Banner Health®

The University of Arizona Health Sciences

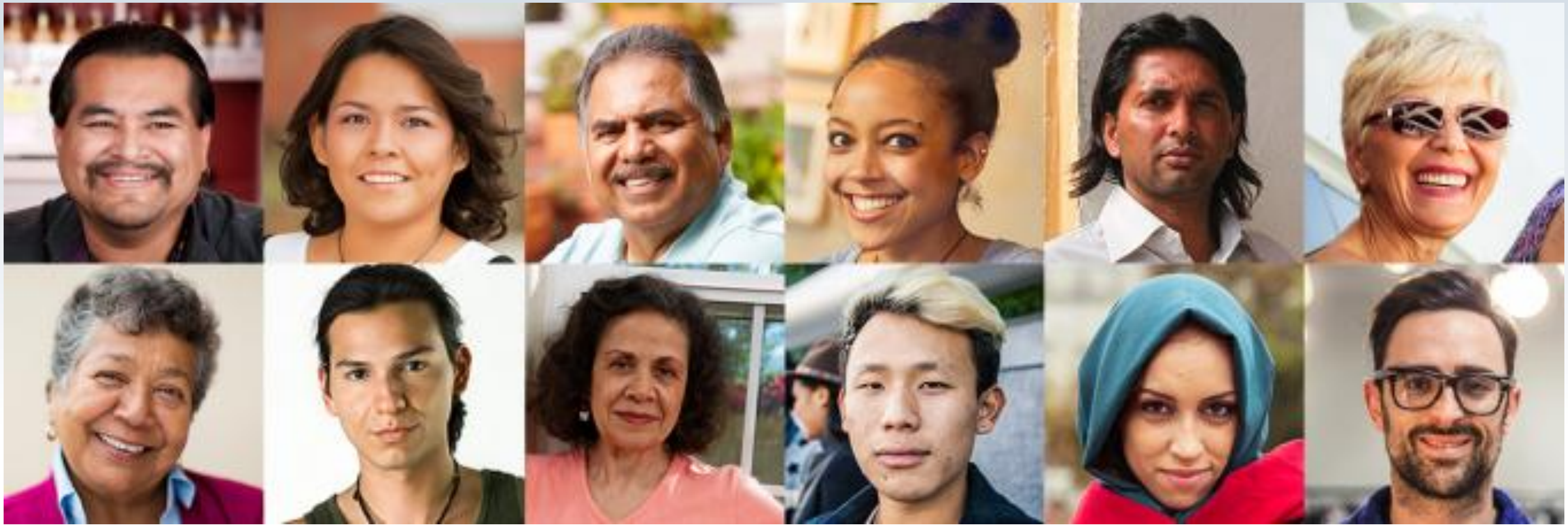

The future of health begins with you.

ARIZONA | COLLEGE OF NURSING

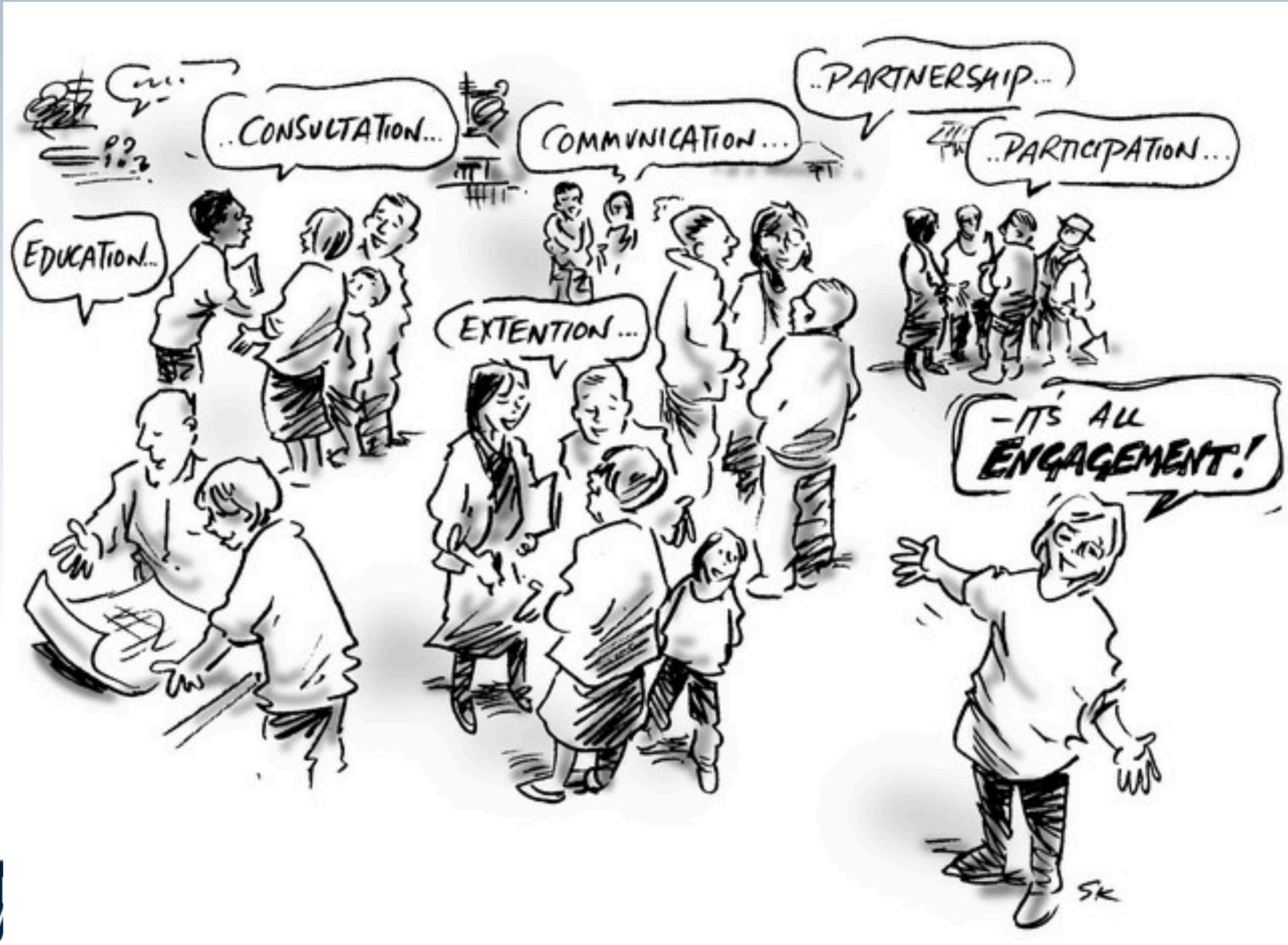
EDUCATION...
...CONSULTATION...
COMMUNICATION...
..PARTNERSHIP...
..PARTICIPATION...
EXTENTION...
-IT'S ALL ENGAGEMENT!
SK

Community Engagement in Precision Medicine

- Equality vs. Equity
- Engagement
  - Outreach (uni-directional)
  - Include bi-directional strategies

Fostering the Norm of Inclusion

- Defined as "the planned efforts to ***consistently engage and examine diverse groups***" in research and health care.
  - Historical paradigms: overreliance on convenience – readily accessible groups
  - Reorientation towards maximizing diversity, **planned and targeted inclusion**, and meaningful representation to promote equity
    - Reaching "hard to reach" underserved/marginalized groups
    - Thoughtful inclusion/exclusion criteria
- The goal is to increase access and acceptability of interventions, and foster early dissemination to all.
- Alcaraz, et al, 2016

Engagement at UA-Banner Health

- Systematic
- Intentional process
- Reflects needs, preferences and priorities
- Inclusive of the range of age, social, racial, ethnic, cultural, geographical and health statuses of individuals

Engagement vs. Recruitment

- Engagement provides the opportunity to interact with partners where the outcome may be a bi-directional increase of knowledge, information and increased partner input.
- Outcome of recruitment and enrollment is participation.
- As such, engagement strategies are threaded through awareness, recruitment, enrollment and retention activities.

Setbacks

- *Elvis is dead and I don't feel so good myself!*

- *Lewis Grizzard*

Is there a formula?

- *I wish I had a Kryptonite cross, because then you could keep both Dracula AND Superman away.*

- *Jack Handy*

How and where does culture fit in?

- *Strategies to target health education programs:*
- **Peripheral strategies** – colors, images, graphics

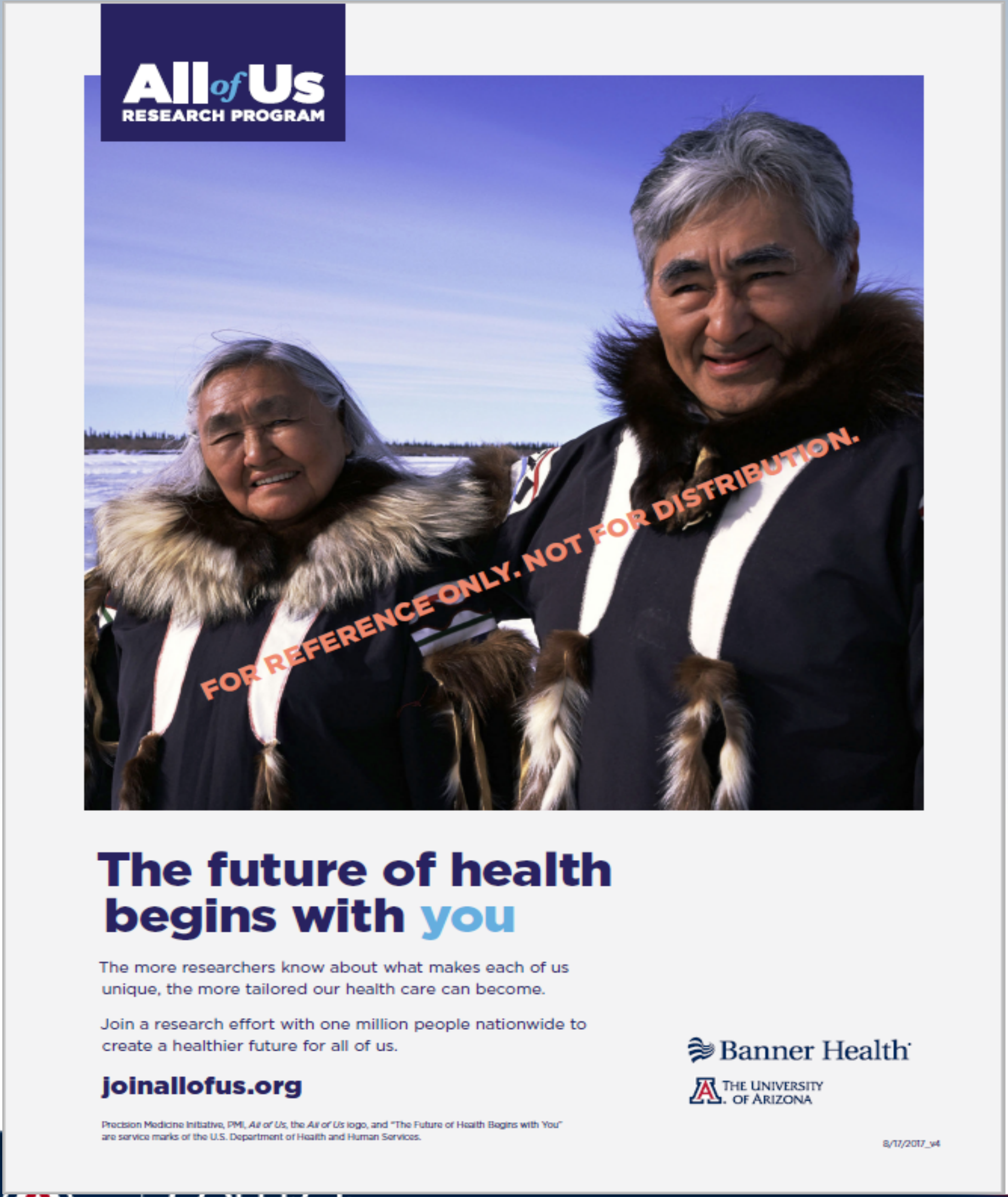

All of Us
RESEARCH PROGRAM
FOR REFERENCE ONLY. NOT FOR DISTRIBUTION.
The future of health begins with you
The more researchers know about what makes each of us unique, the more tailored our health care can become.
Join a research effort with one million people nationwide to create a healthier future for all of us.
joinallofus.org
Banner Health
The University of Arizona
Precision Medicine Initiative, PMI, All of Us, the All of Us logo, and "The Future of Health Begins with You" are service marks of the U.S. Department of Health and Human Services.
8/17/2017_v4

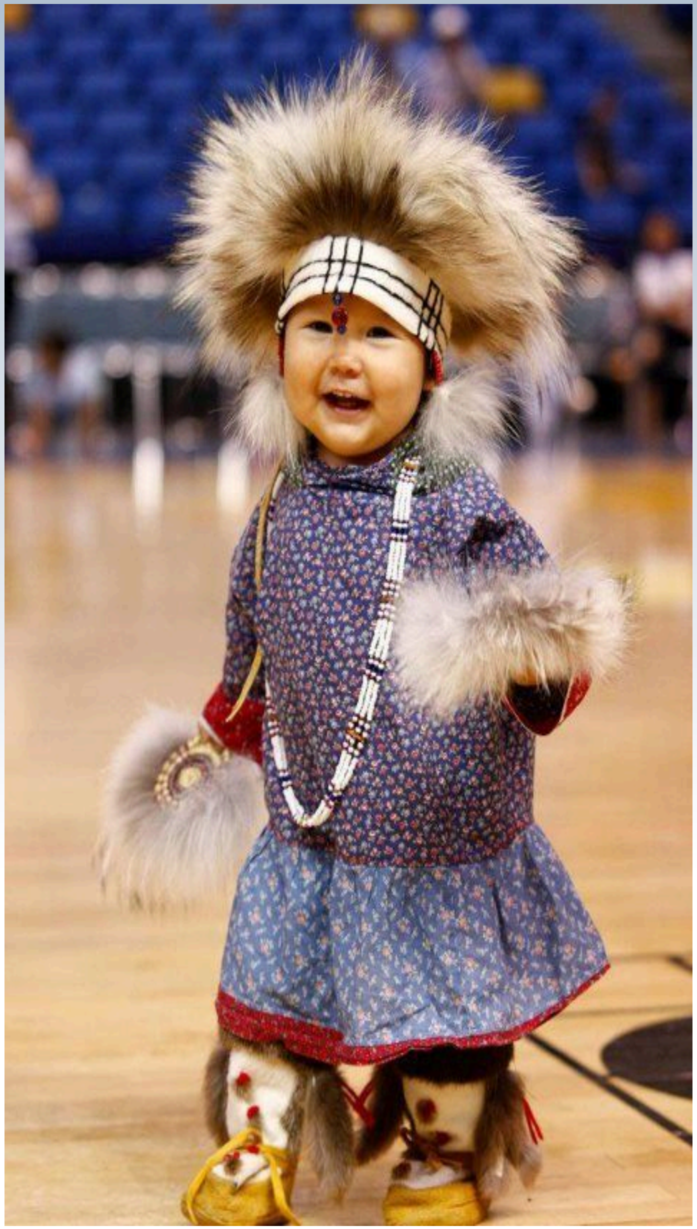

- **Evidential strategies** - enhance the perceived relevance of a health issue for a given group by presenting evidence of its impact on that group

Example

- Suicide was the 8th leading cause of death for Asian-Americans, whereas it was the 11th leading cause of death for all racial groups combined.

Example

- Hispanic/Latinos are the largest ethnic minority group in the U.S. comprising 17.6 % of the total U.S. population (56.6 million).
- Projected that the Hispanic/Latino population will constitute 28.6 % of the total U.S. population by 2060.
- However, Hispanic/Latinos represent only 1% of participants nationwide in clinical trials.

- **Linguistic strategies** – language, vernacular, idioms

- The vodka is good but the steak was bad.

- The spirit is willing but the flesh is weak.

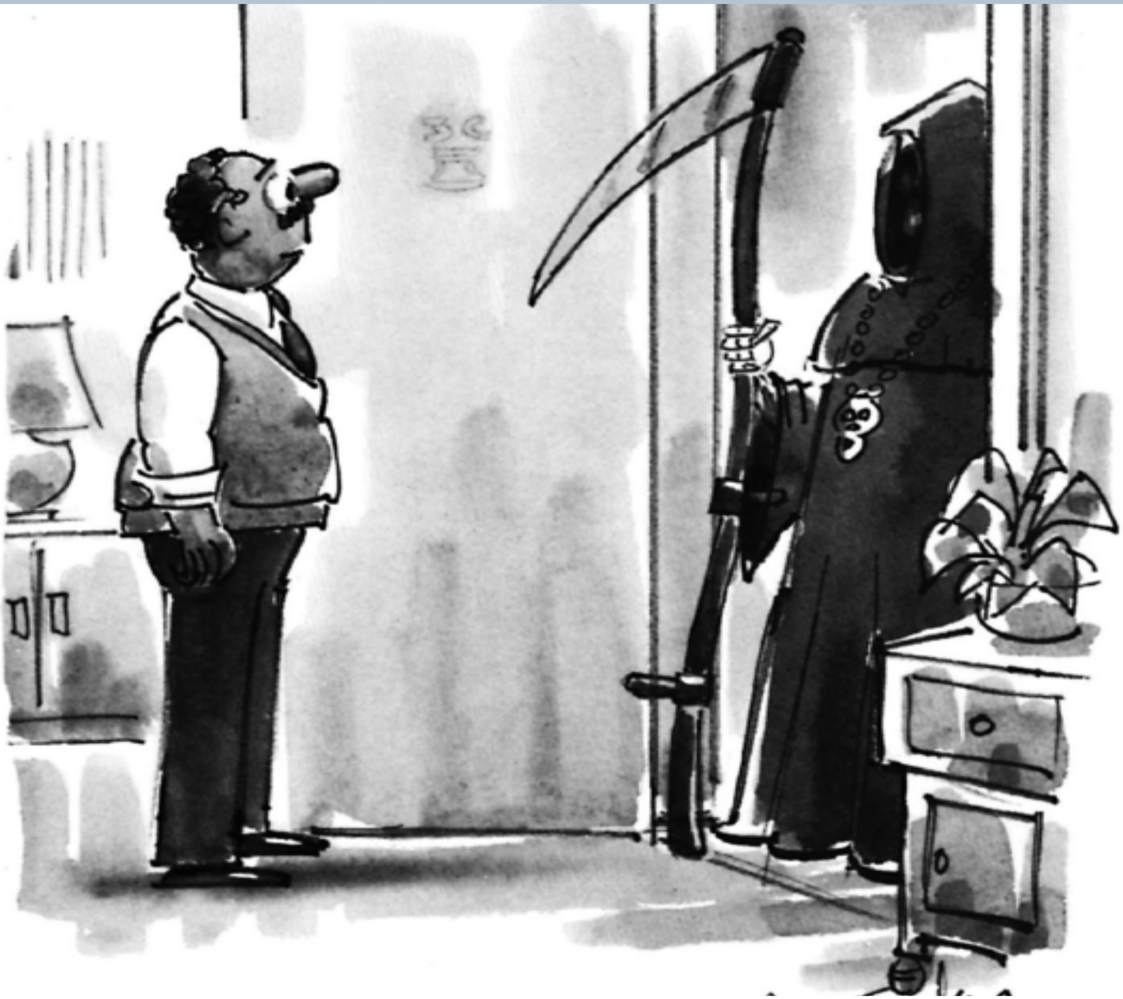

You'll be happy to know that race played no part in this decision!

- **Constituent-involving strategies** – lay health workers, community members as staff

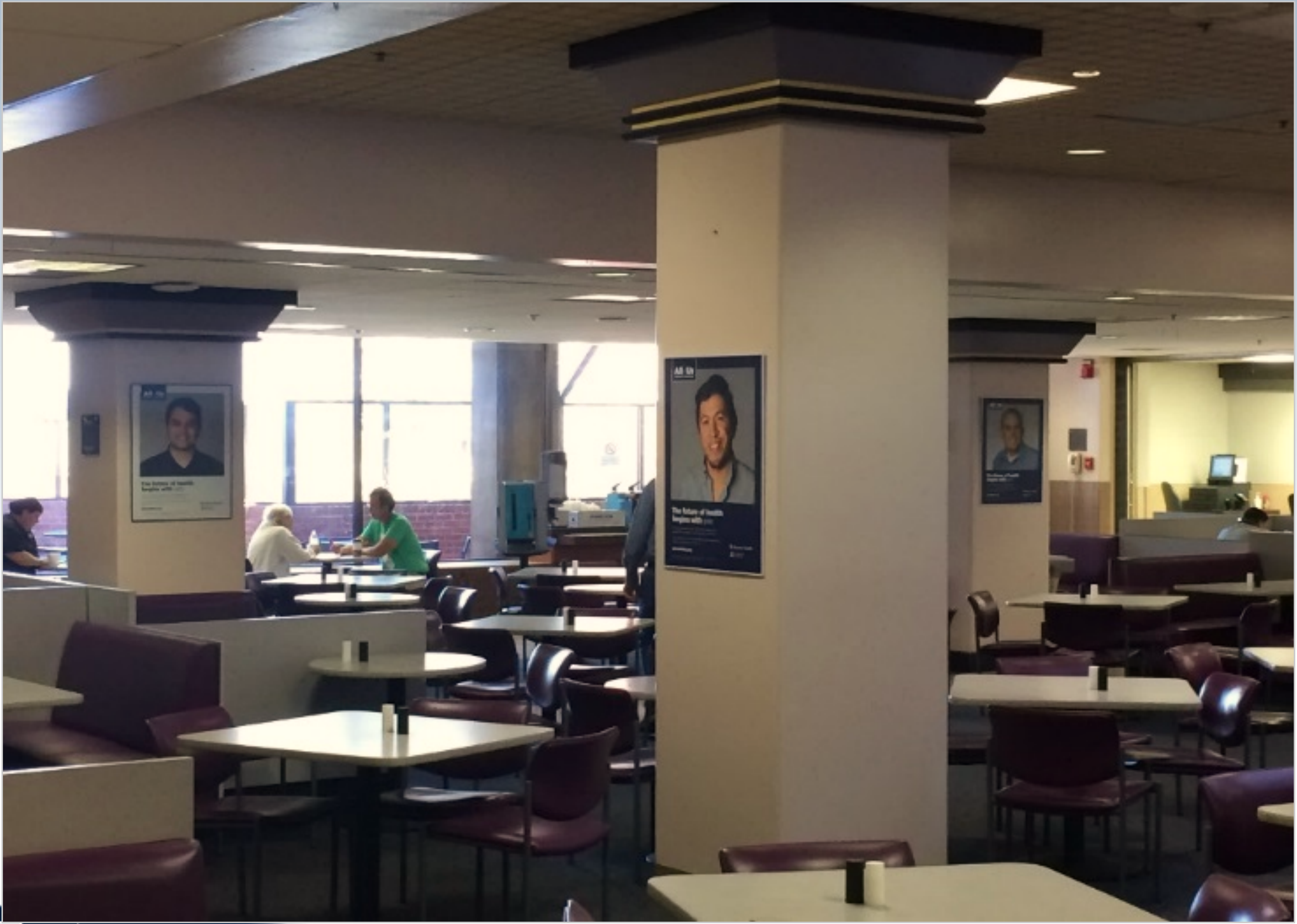

All of Us RESEARCH PROGRAM | Banner Health | ARIZONA

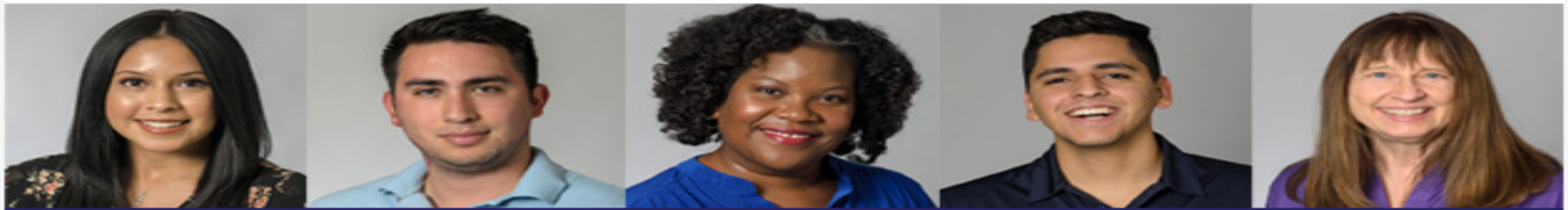

THE FUTURE OF HEALTH BEGINS WITH YOU

Thank you for being one in a million! We are sending this newsletter to keep you informed about the *All of Us* Research Program at UA-Banner as well as to provide a few useful tips for the month.

AllofUsAZ.org | 877-268-2684

YOU ARE NOW ONE OF 506 PARTICIPANTS FROM ARIZONA!

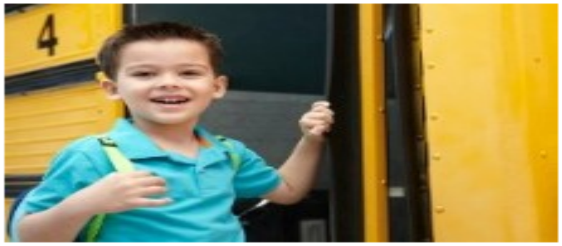

Back to School Safety

As summer draws to a close and children start heading back to school, family life can get pretty hectic. It's important to remember – and share with your children – some key tips that will help keep them safe and healthy throughout the school year. Click the photo for more.

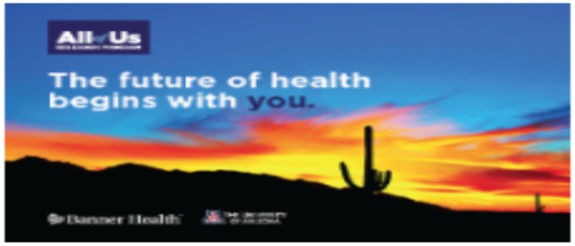

All of Us Anthem Video

Click on the photo to follow us on Facebook and see the recently-released *All of Us* anthem video. *All of Us* is more than just a medical research program. It's the promise of positive change, for generations to come. #JoinAllofUs

- **Sociocultural strategies** - a group's cultural values, beliefs, and behaviors are recognized, reinforced, and built upon to provide context and meaning to information and messages about a given health problem or behavior

- Kreuter et al. (2003). Achieving cultural appropriateness in health promotion programs: targeted and tailored approaches. Health Education and Behavior, Vol. 30 (2): 133-146

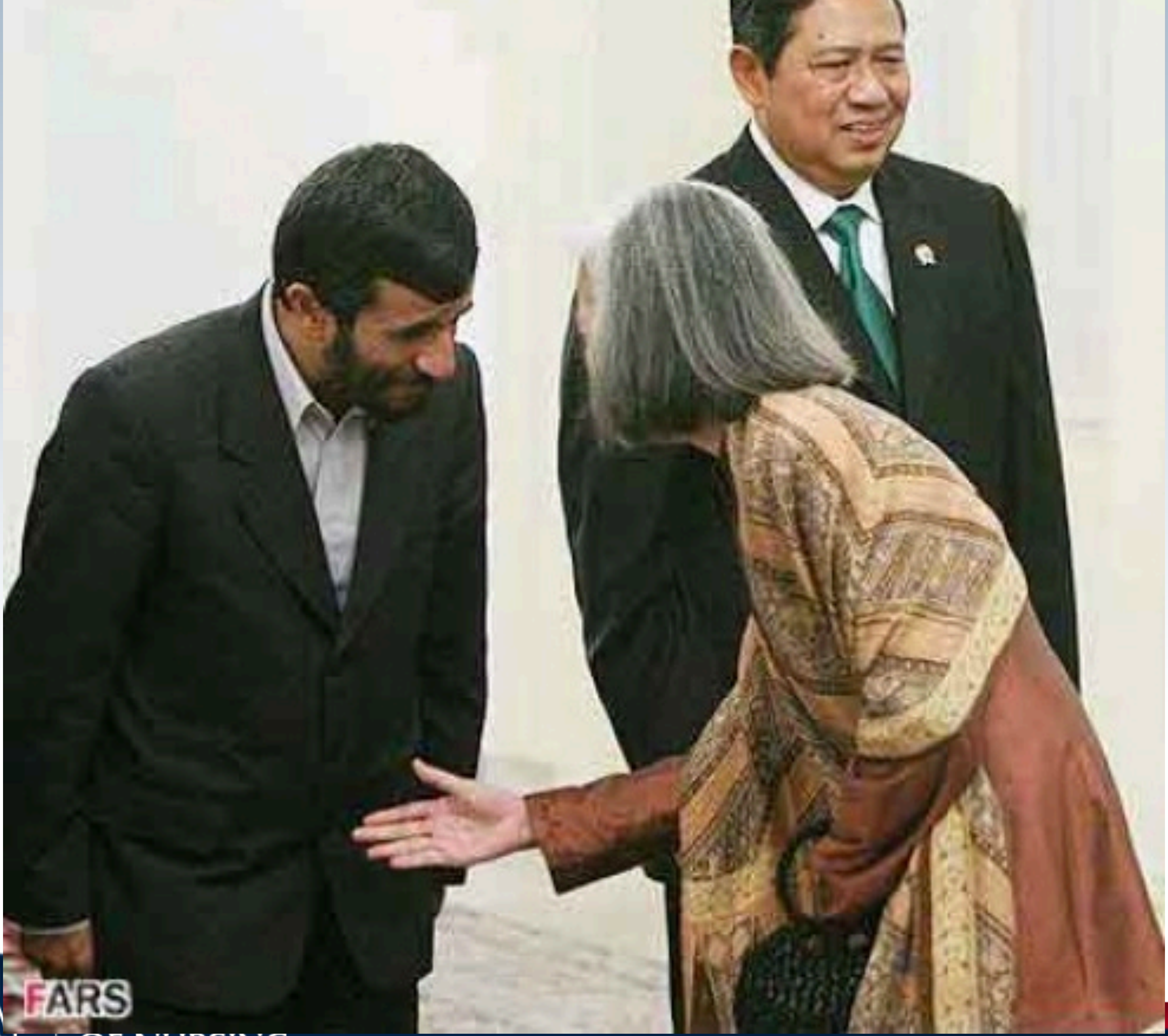
FARS

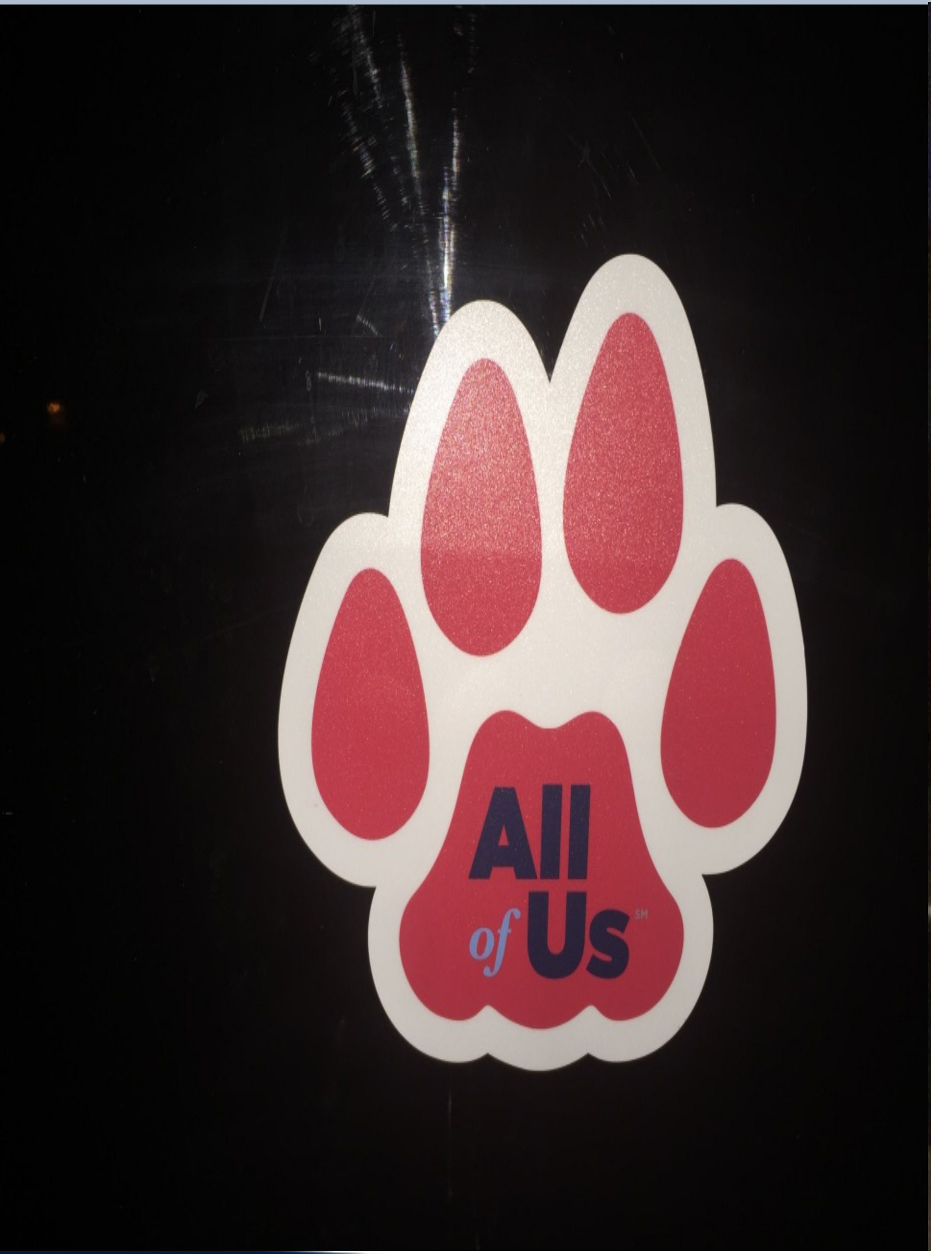
All
of Us
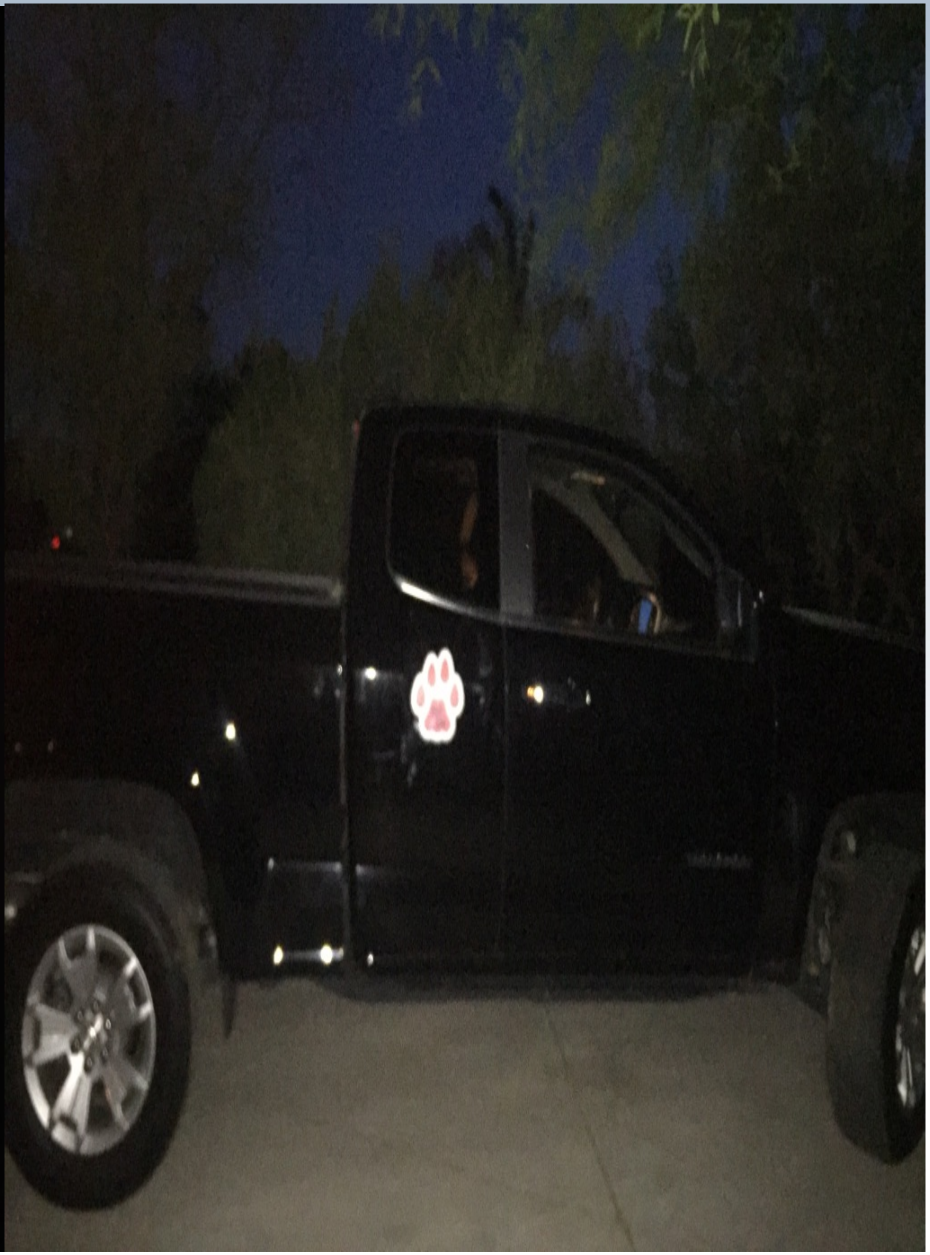

- **PERSPECTIVE**

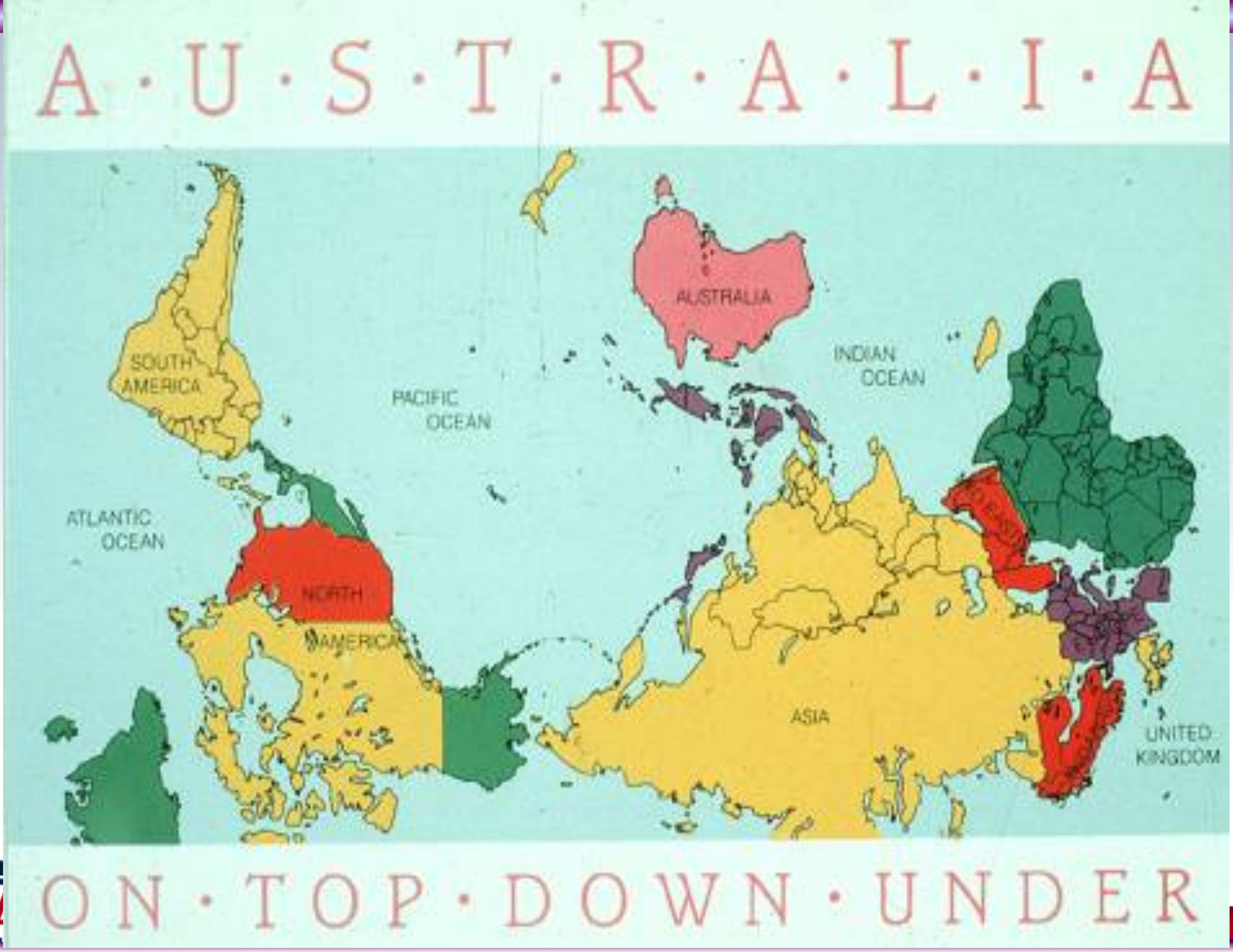
A·U·S·T·R·A·L·I·A
SOUTH AMERICA
PACIFIC OCEAN
AUSTRALIA
INDIAN OCEAN
ATLANTIC OCEAN
NORTH AMERICA
ASIA
UNITED KINGDOM
ON·TOP·DOWN·UNDER

- “The impossible could not have happened, therefore the impossible must be possible in spite of appearances.”

  — Agatha Christie, *Murder on the Orient Express*

FAIR ISN'T

everybody getting the same thing.......

FAIR IS

everybody getting what they need in order to be

SUCCESSFUL.

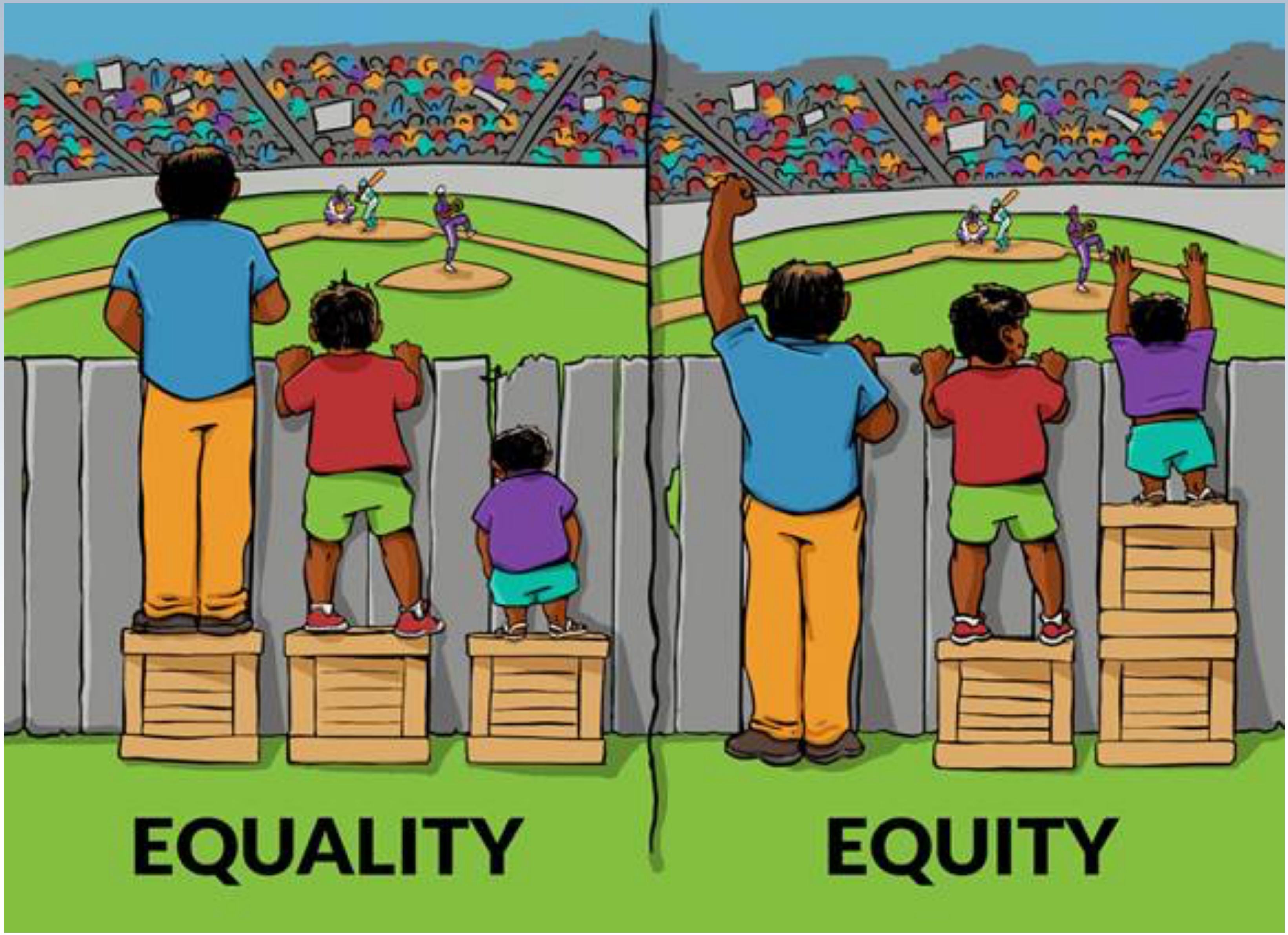
EQUALITY
EQUITY

Who is Accountable?

- *If you ever crawl inside an old hollow log and go to sleep, and while you're in there some guys come and seal up both ends and then put it on a truck and take it to another city, boy, I don't know what to tell you.*

- *Jack Handy*

The time to act is now

- *When my love comes back from the restroom will I be too old to care?*

- *Lewis Grizzard*

Who's watching us?

Don't bend over in the garden, Granny,
you know them taters got eyes!

Lewis Grizzard

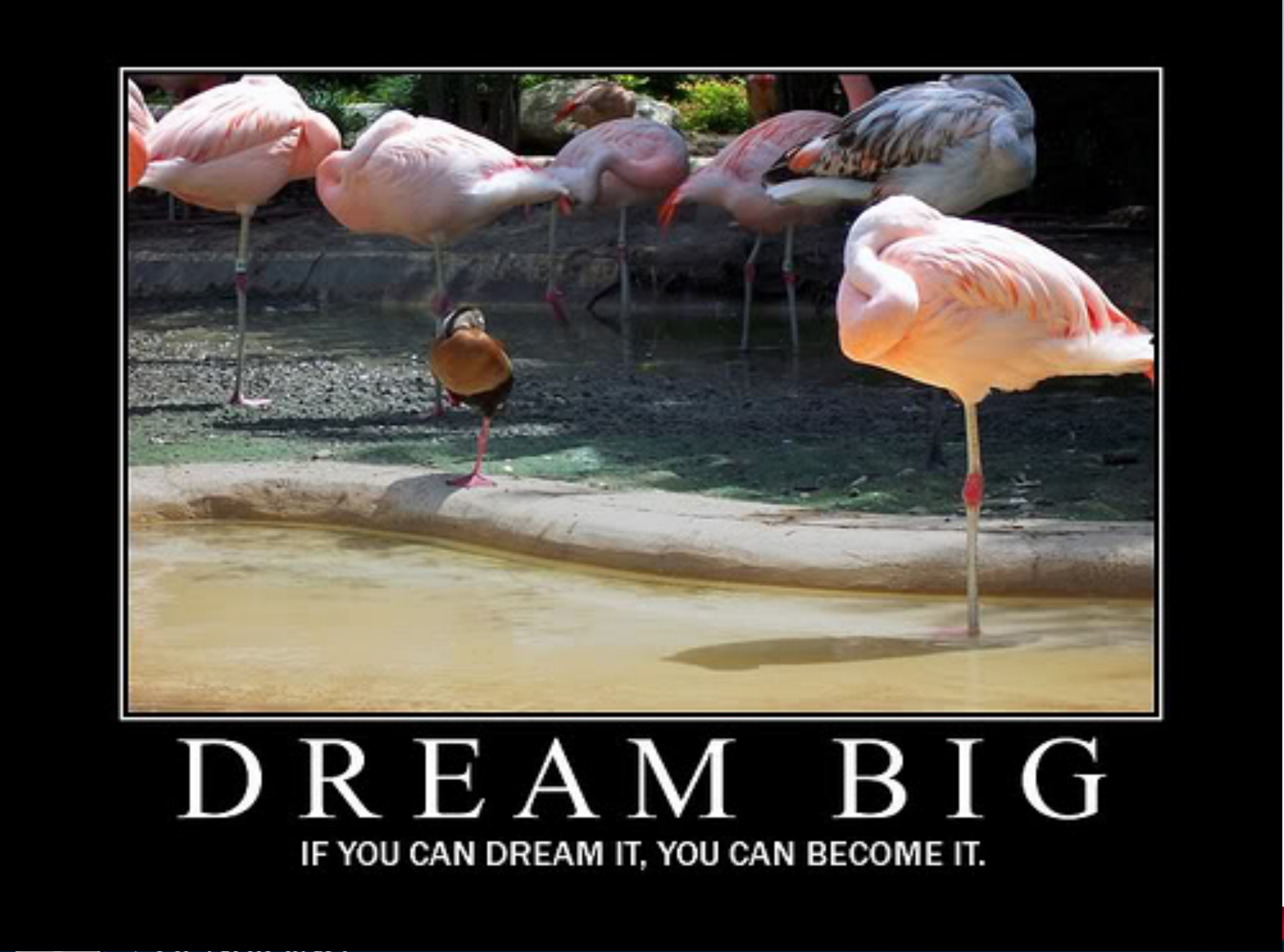
DREAM BIG
IF YOU CAN DREAM IT, YOU CAN BECOME IT.

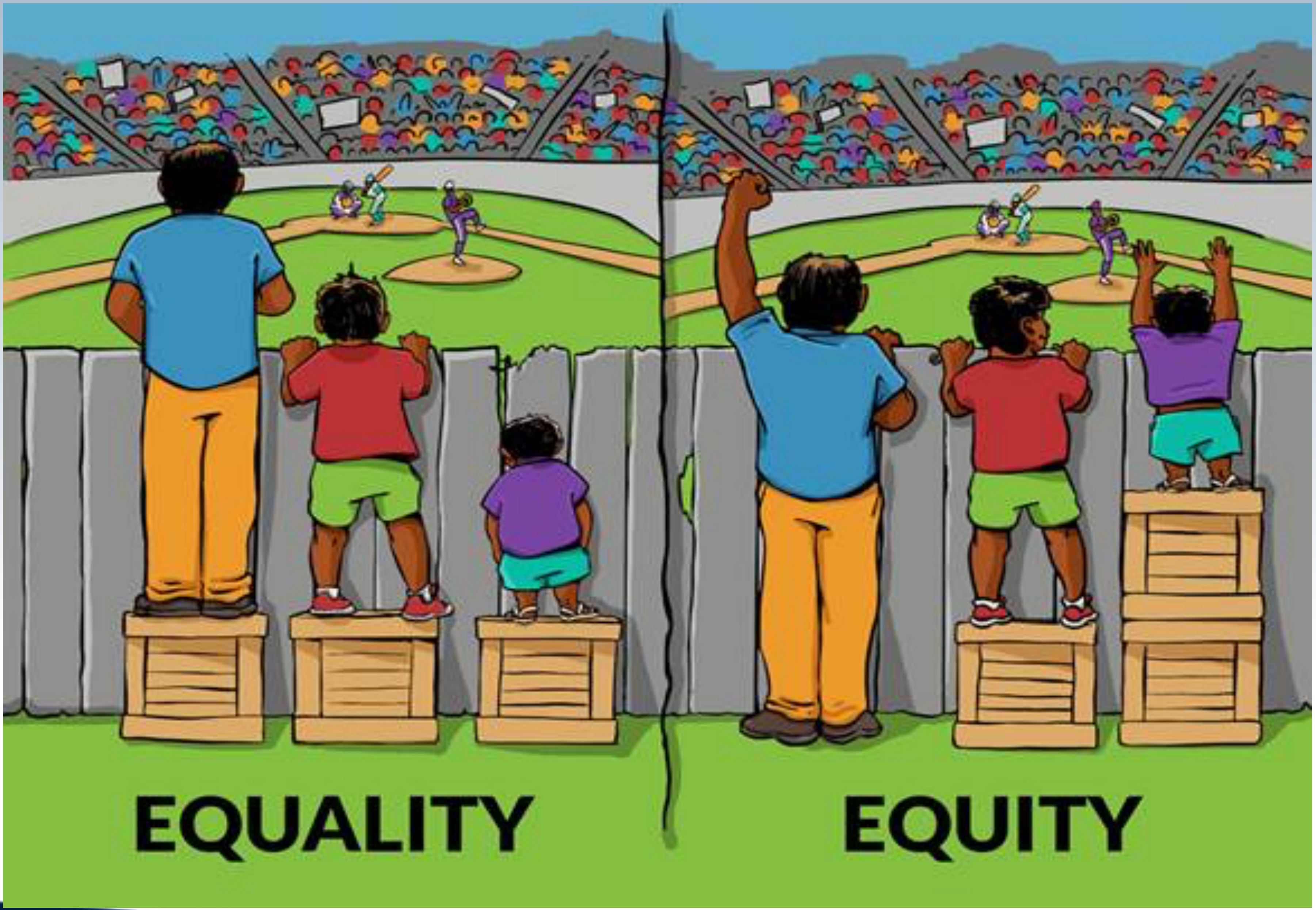
EQUALITY
EQUITY

Academic-Community Partnerships for Precision Medicine

Chanita Hughes-Halbert

hughesha@musc.edu

Associate Dean for Assessment, Evaluation and Quality Improvement, Medical University of South Carolina

Charleston, South Carolina

Abstract

Dr. Hughes-Halbert discusses the landscape around precision medicine and academic community partnerships within the context of the MUSC [*Medical University of South Carolina*] Transdisciplinary Collaborative Center in Precision Medicine and Minority Men's Health. Minority men remain underrepresented, even within health disparities research, and this NIMHD-funded center focuses on transdisciplinary research in the health areas that most affect these men in the United States - namely heart disease, stroke, diabetes, and HIV. The Center includes investigators from many disciplines, including medical oncologists, geneticists, bioinformaticians, nursing science, and individuals with expertise in dissemination and implementation science.

[*Dr. Hughes-Halbert*] Good afternoon, everyone. I am really excited to be here and would like to thank the conference organizers for inviting me to talk about academic community partnerships for precision medicine. Because one of the roles I play at MUSC [*Medical University of South Carolina*] is Director for the Special Populations Core, and along with my colleague, Dana Burshell, we work really closely with our Community Engaged Scholars program to think about issues related to inclusion and diversity.

What I wanted to talk about today is the work that we have been doing within the context of precision medicine and academic community partnerships and frame this within the context of our Transdisciplinary Collaborative Center in Precision Medicine and Minority Men's Health. I think about it as a very large academic community partnership, so we actually have some of the same slides. [SLIDE 2] Great minds think alike. I would echo the comments that were just made because we are at a very critical point in healthcare and healthcare delivery with the opportunity for precision medicine to really promote equity by providing individualized strategies for early detection, prevention, and treatment. The All of Us Research Program (again, great minds thinking alike) [SLIDE 3] builds on the forethought that NIMHD had in terms of active community engagement, meaningful community engagement, and the research program. When I was at the University of Pennsylvania, we were really fortunate to be one of the centers funded through this community-based participatory research program at NIMHD. [SLIDE 4] We really thought about that as we developed our Transdisciplinary Collaborative Center (TCC) in Precision Medicine and Minority Men's Health, and one of the first things we want to start with issues and topics that are relevant for community organizations. As we

thought about building our TCC, [SLIDE 5] we wanted to build a regional consortium that supported active engagement of academic community stakeholders and public health stakeholders in precision medicine and health disparities research, and as part of that, really engage collaboratively across a multiregional consortium. In our translational research efforts, we really wanted to think about and develop and implement strategies for dissemination and knowledge translation to facilitate implementation of best practices of precision medicine approaches.

I wanted to tell you why we decided to focus on minority men's health within the context of health disparities. [SLIDE 6] I mean, we have heard about that over today about disparities from our keynote speaker. We heard about the disparities that exist. We focused on minority men because as we did a landscape review of the research in this area, minority men were underrepresented with respect to the larger health disparities research agenda, and we felt it was time to take a stand and really engage and develop and conduct transdisciplinary research that addressed some of the pressing issues that face minority men - heart disease, stroke, diabetes, and HIV, as examples of some of the disparities where we see particularly striking disparities. One of the things that I learned working as part of the West Philadelphia Consortium to Address Disparities is that it really does take a collaborative effort, and the TCC approach really pushed us to move beyond sort of a citywide perspective and think more regionally in how we address minority health and health disparities. As you can see here, [SLIDE 7] we brought together several different types of academic institutions of the University of Pennsylvania, Hampton University, which was leading a Transdisciplinary Collaborative Center in Minority Men's Health and had a focus on chronic disease prevention. The University of Texas Health Sciences Center in San Antonio has a focus on minority health from the perspective of Hispanic populations. We wanted to be inclusive from an academic side, but also engage diverse types of community-based organizations. [SLIDE 8] We brought together the Low Country Area Health Education Consortium, which is our AHEC in South Carolina but has a regional/national outreach approach. We also continue to partner with the National Black Leadership Initiative on Cancer and the Hope Institute based out of Baltimore, Maryland, which focuses on ethical, legal, and social issues with respect to inclusion and diversity in clinical trials. We are partnering with the Southeastern Health Equity Council to think about, again from a regional perspective, diversity and disparities issues.

We built upon the base we had established with our academic community partnership through the work in Philadelphia and expanded that in the model that we implemented in South Carolina through our TCC in

Precision Medicine and Minority Men's Health. We needed to have multidisciplinary investigators in a leadership capacity across our center. [SLIDE 9] We have a very diverse group of investigators, who are actively engaged in our center leadership. The group includes medical oncologists, geneticists, bioinformaticians, nursing science, and individuals with expertise in dissemination and implementation science. Our overarching framework is shown here [SLIDE 10]. I was really excited to hear stress and stress reactivity mentioned in the keynote address, because as we were thinking about our application, we recognized that there are some unique stressors that minority men face, both acute stressors and chronic stressors. We also know from the literature that stress and stress reactivity impacts the initiation and progression of disease, and we were interested in thinking about how could we use that? How can we establish markers to understand biological functioning? That is really the overarching framework of our center, to understand the allostatic process and how it influences disease initiation and progression. [SLIDE 11] When we thought about diseases, we know there are many conditions where disparities exist. Some of the things that we thought about in focusing our initial center work was that we wanted to focus on a condition that had a significant clinical and public health impact and also a condition that was relevant for community stakeholders and individuals in the community. We also wanted to understand diseases that had biological, clinical, and public health relevance to other chronic conditions. [SLIDE 12] As we thought about this, and again, building upon the work that we and others have done, it led us to focus on prostate cancer as part of our initial center focus. So, as you can see here, we have looked at utilization of prostate cancer screening among African American men and found that, although there is a recommendation that men engage in an informed decision-making process, that really doesn't play out in terms of how men are making decisions about prostate cancer screening and our work, as cited here. And we also know, if you think about the continuum from screening to diagnosis to treatment, that there are differences in quality of life following prostate cancer diagnosis [SLIDE 13]and those differences are influenced by men's sociocultural factors, so their beliefs about religiosity and temporal orientation and are important as far as how they react to being diagnosed with prostate cancer. Then, we think about the context of minority men being included in any type of study, but particularly when we think about participation in genetics research, that there is a wide range of level of intentions and interest. Some of my early work focused on genetic counseling and testing for inherited breast cancer risk and so we were thinking, and one of the challenges we experienced was, making our information available to women across diverse settings. [SLIDE 14] So, we wanted to understand intentions to donate to Biobanks and intentions to participate in precision medicine research, and you see that from the two examples here shown from our own work that there is a lot of variation. If you ask individuals if

they would be willing to participate or donate to a Biobank, about 23% of those are "very likely," but if you ask them or give them more information about the attributes of the study, what it would actually involve in terms of their participation, it is much less likely with only 31% saying they would participate in a study that was sponsored by the government where they had to give a biospecimen or complete a survey about their personal and health history and that they would not get results back. So, again, if you provide more information about the research, we see less of a willingness to participate. So, I am really excited to see some of the work that you are doing to engage populations in the All of Us research program.

This just shows you an overview of our center projects,[SLIDE 15] again, focusing on stress and stress reactivity and how it affects some of the critical junctures within the trajectory of being diagnosed and treated for prostate cancer. What I will now share is how we are organized, because I think as I have been working within the context of academic community partnerships and some of the last work that we have been doing in Philadelphia, one of the things that I have been realizing is that it really does take a lot of forethought and effort to organize the administrative components to ensure that there is a broad strategic oversight and management of the day-to-day activities of the partnership to ensure that things are happening as they should happen and that you stay on target. One of my partners when I was an Assistant Professor was like, "You are such a task manager - how can you be so uptight at such a young age?" I was like, "We've got to keep the ball rolling here!" So, that has some advantages and some strengths, depending on the context.

So, what I want to share with you now is sort of the organization of our center and really how some of the roles and challenges that we have experienced ... not challenges, but the decisions that we have made along the way. So, our Consortium Core [SLIDE 16] is responsible for the overarching infrastructure that provides the direction and sets the priorities and governance of our center. Some of the things that the Consortium Core is charged with is working across all of our other cores to implement strategies and develop a research program that addresses the priorities that have been identified with respect to precision medicine. We also have an implementation core[SLIDE 17] that works very closely with the Consortium Core to develop materials and methods for determining readiness and capacity of different stakeholders across regions to implement and adopt precision medicine approaches and to clinical care and public health practice. Our Data Integration Core[SLIDE 18] is working to integrate sort of on a much more detailed level (the data that is being generated through all of our projects) so that we can use that information to translate it into effective strategies for precision medicine. So, as we have taken a step back, we are now in our second year of our center. The first

year was really spent building the infrastructure, building the administrative components, and making sure that all of our cores and projects had timelines and milestones that were relevant for the work that we were charged with doing. As I prepared this talk, it really made me think - how do we go about integrating all of these activities to make sure we are engaging our academic and community partners effectively and meaningfully? [SLIDE 19]So, one of the things we did was first to take a step back and think about this within an overarching framework. [SLIDE 20]Ultimately, we want to develop strategies that can be implemented into care - public health practice, clinical care. So, as we think about this from a translational perspective, we are dealing with work that will understand the ways in which biological, clinical, and social factors influence the progression and initiation of disease, but ultimately, we want to translate that down into evidence-based recommendations or policies that are implemented in healthcare systems and prevention programs. The first thing we had to do to do that was for us to sort of come up with a definition of precision medicine[SLIDE 21] that made sense to us and guided the overarching work that we did. You can see here that our definition of precision medicine that we adopted is consistent with the one with NIH, but I have highlighted the focus for us and that is really to ensure that people receive the right medical care in the right dose to the right patient at the right time, and that really resonated with the academic and community members of our consortium core and has resonated with all of our constituents in each of our four activities and center projects.

So, we have also worked to establish goals for community engagement. [SLIDE 22] This was something that both our academic and community partners and our consortium core really felt like we needed to do so that we had an overarching framework that guided the activities that we did with respect to community engagement. You can see here that our goals for community engagement [SLIDE 23] ... we don't just want to send information out to be sending information out. We want it to be information that people can use to increase their knowledge and awareness and their level of being informed about precision medicine and they can use that information and develop their capacity to apply it to the decisions they are making about their healthcare or clinical practice. We also want to develop and engage different stakeholders to ensure that they are able to make informed decisions about their participation in precision medicine research and/or referring members or patients for participation in studies. I think the point that you made about if people decide at the end of the day that they don't want to participate in a study, that they don't want to participate in any activity, that is okay. The ultimate goal for participation is that people have made an informed choice that is consistent with their knowledge, beliefs, and values. I think most importantly, what we want to be sure that we do as an ultimate goal is to ensure that the use of precision medicine doesn't result in exacerbating racial and ethnic

disparities in healthcare and outcomes. I think that precision medicine is at a really critical juncture where we have to start thinking about and sharing that information and knowledge and disseminate it across diverse settings. One of the things that we know is there are some very complex genomic strategies, and every group will not be able to implement those very complex strategies, but they need to know how to refer people and refer patients so that they are able to access those things so that it does not result in the haves and the have nots. Some of the things that we have started to do is we will ... I guess as a behavioral scientist, I cannot resist the temptation to do a survey, but I think it is important within this context, because one of the things ... just as we have been thinking about precision medicine, we have had a series of conversations about - well, do people really understand what precision medicine is? If you say "precision medicine," when I think about it, it means one thing, and when you think about it, it means something different. So, I do think there is an opportunity and it is important to understand - well, what do people think and know about precision medicine, what are they concerned about, and what are their preferences for where we would focus our efforts initially and over the longer term? I think it is really important to understand organizational capacity for precision medicine and we have been thinking about this from the perspective of - if you are a primary care provider, your roll in precision may be a little different than if you are at an NCI-designated comprehensive cancer center. So, we want to understand the roles and responsibilities and capacities that different types of organizational healthcare settings have for precision medicine so that we can understand what resources are needed and how ready they are and what types of activities need to happen to increase their readiness.

Our last engagement strategy is something we are really excited about. It is an Evidence Academy. [SLIDE 24] An Evidence Academy is an approach that one of my colleagues, Cathy Melvin, at MUSC, has developed with colleagues at UMC Chapel Hill that focuses on knowledge translation for the purpose of developing tailored implementation strategies. So, I think there are lots of examples where there have been policy recommendations passed down from (I called it "on-high") policymakers like CMS and others where it's like there are really great policies, but when you think about how you implement that in a busy clinical practice, there is sort of like a disconnect between where the rubber will actually meet the road. So, the Evidence Academy is really structured around sort of a co-learning process where individuals sort of present their understanding, bring everyone to the same baseline understanding about a particular issue, and then work to develop best practices that are realistic for their particular type of setting. So, we are doing that through regional implementation teams. We have developed them to be composed of different types of stakeholders

who are critical for adopting precision medicine. So, it includes healthcare providers, community public health officials, as well as individual patients. So, these are the things that we will be asking them to do. I am over the future years of our center, so I am really excited about that, and the first thing is we will bring them to our Evidence Academy, which will be held November 16th to sort of start our work with respect to dissemination and implementation.

So, I thought I would conclude by sharing some thoughts. One is - as we developed our regional implementation teams, we thought very carefully about who are the right stakeholders[SLIDE 25] who need to be engaged in this conversation at this point? I think one of the challenges with precision medicine is that it is emerging. It is a work in progress, so the actual clinical implication is being defined. I like how you described it as a plane that is being built and we are flying it now. So, determining who the right stakeholders within that context has been a little challenging, I must admit. The other thing that I have learned over the years is - when and how do we expand our partnership and when do we limit participation? We have a very effective working partnership at the moment, but as we have been thinking about this, one of the decisions we will have to make is - when will it be time to expand our partnership and what will we need to sort of bring it back in to keep it more precise (I guess some pun intended)? [SLIDE 26] The other things that I think about are - as we talk to community stakeholders and patients in different healthcare settings, I always come back to this issue of precision medicine versus basic healthcare services, and one example that comes to mind (and I am hearing the latest in exome sequencing and genome sequencing and those are really very specialized services), I think we want to be able to develop and implement strategies for precision medicine that really enhance basic healthcare services and really facilitate access to and addresses basic healthcare needs. The last point that I will make is - in thinking about the structure for our consortium, it has been really critical to have a very strong administrative team that works to coordinate and integrate all of the activities across our different cores and research projects. I will end there. Maybe that will be a point for discussion.

Declarations

- The presenter has no financial competing interests related to this presentation.
- All authors have approved the manuscript for submission
- The content of this presentation and transcript has not been published or submitted for publication elsewhere.
- This presentation was organized by the Meharry-Vanderbilt Alliance with support from NCATS and the NIMHD of the National Institutes of Health under award number R13TR001694

Academic-Community Partnerships for Precision Medicine

CHANITA HUGHES HALBERT, PHD
Department of Psychiatry and behavioral Sciences
Hollings cancer center
South Carolina Center for Translational Research
Medical University of South Carolina

VISION FOR PRECISION MEDICINE

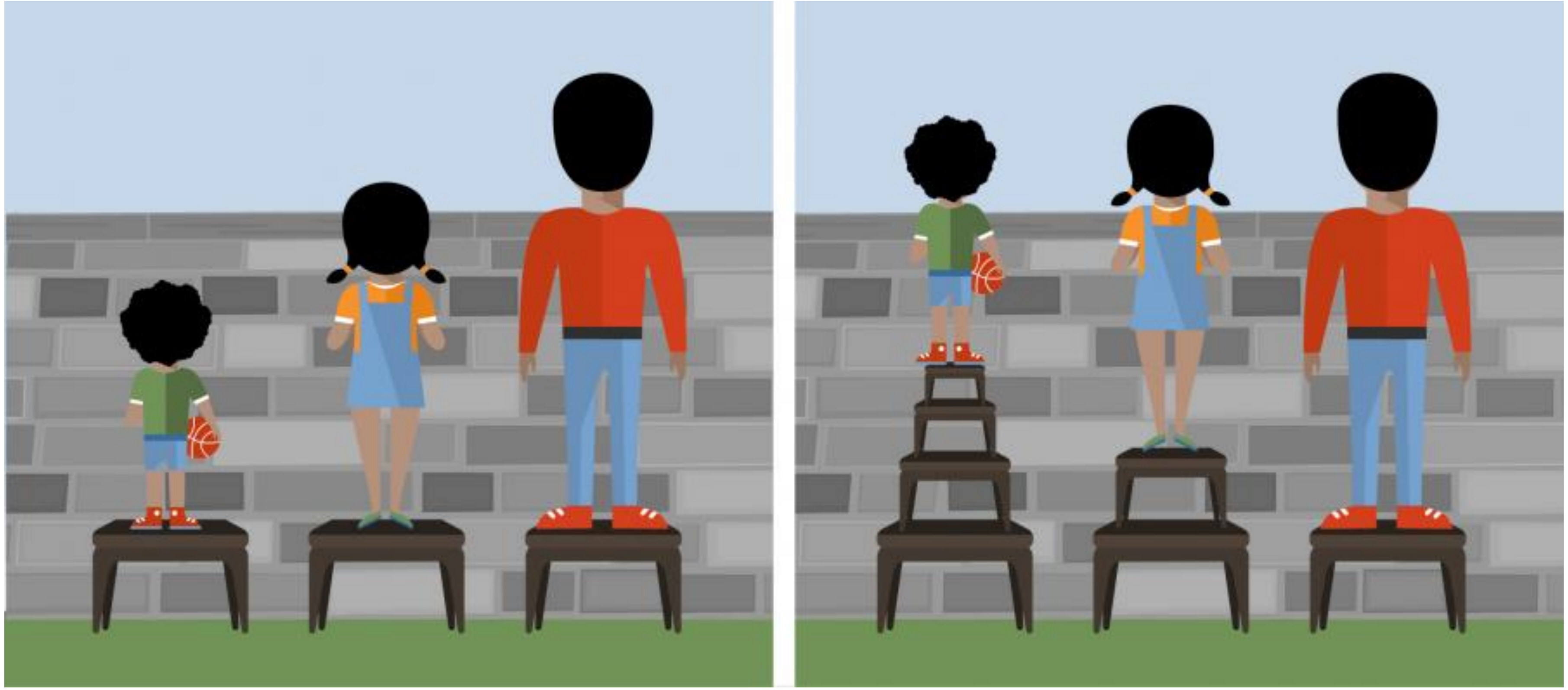

Promote health equity through individualized approaches for early detection, prevention, and treatment

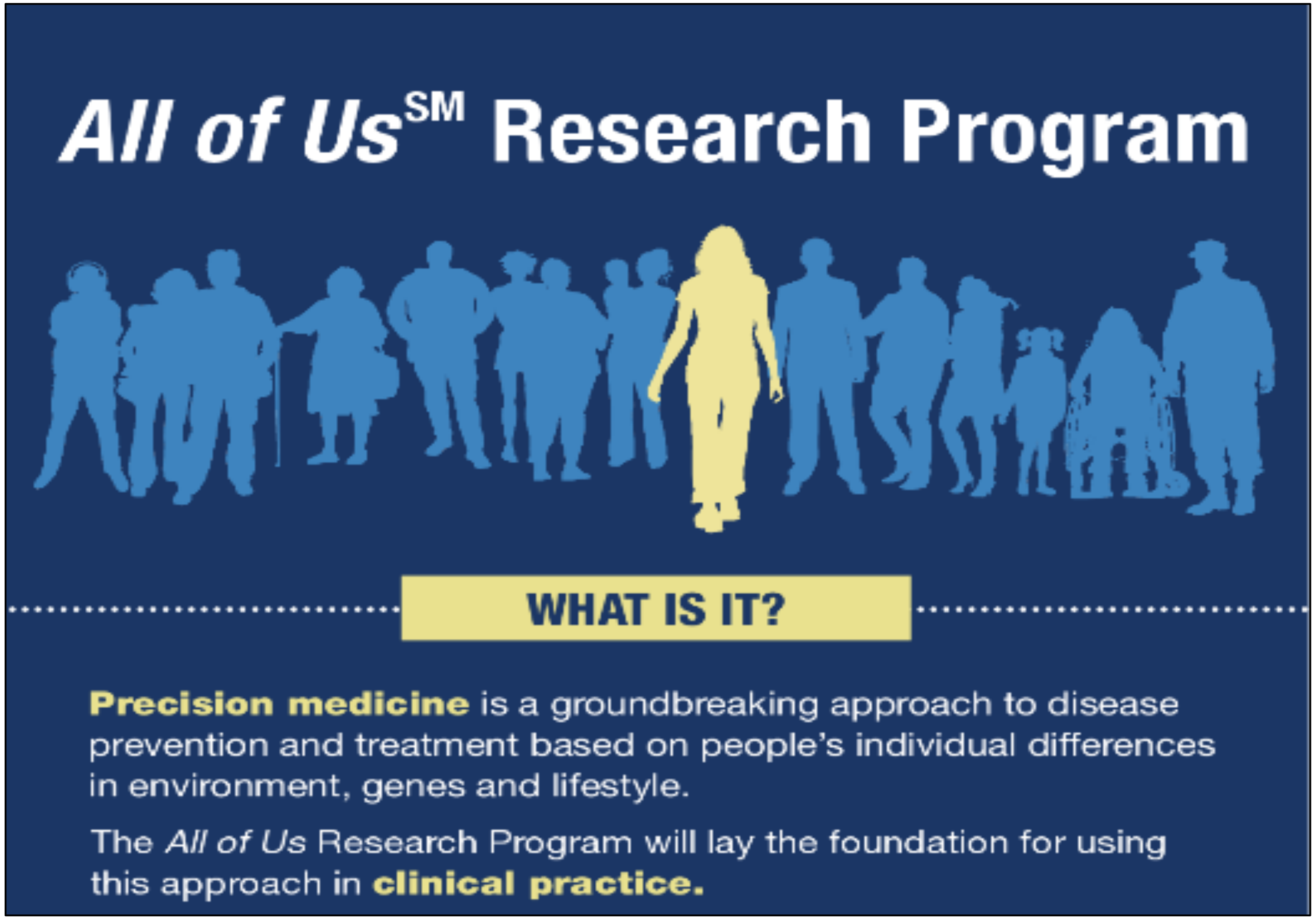
All of Us SM Research Program
WHAT IS IT?
Precision medicine is a groundbreaking approach to disease prevention and treatment based on people's individual differences in environment, genes and lifestyle.
The All of Us Research Program will lay the foundation for using this approach in clinical practice.

PRINCIPLES OF ACADEMIC-COMMUNITY PARTNERSHIPS

- Community-based participatory research (in health) is a collaborative approach to research that equitably involves all partners in the research process and recognizes the unique strengths that each brings

- CBPR begins with a research topic of importance to the community with the aim of combining knowledge and action for social change to improve community (health)

Mission

- Establish a Consortium that supports the active engagement of diverse stakeholders in precision medicine and health disparities research
- Conduct translational research to understand the interaction between biological, social, psychological, behavioral, and clinical factors and health care and disease outcomes among minority men
- Support the dissemination of evidence related to precision medicine and health disparities
- Integrate data on biological, social, psychological, and clinical factors to validate findings from prospective TCC research

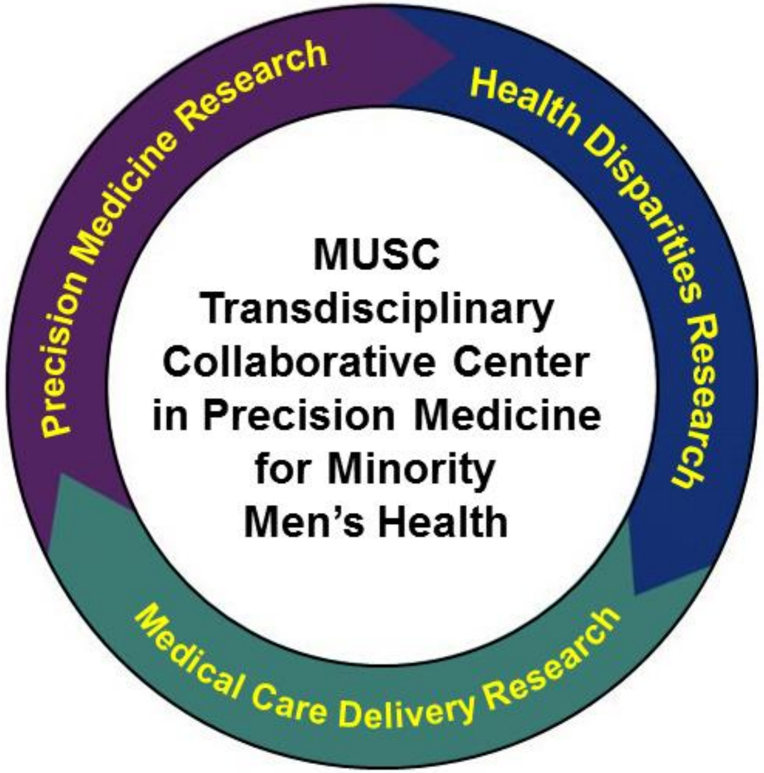

NIMHD

NATIONAL CANCER INSTITUTE®

Rationale

- Minority men have poor health outcomes and lower life expectancy compared to non-minority men
  - African American men 30% more likely to die from heart disease than white men
  - African American men are 60% more likely to die from stroke than white men
  - Hispanic men are twice as likely than white men to die from diabetes
  - Hispanic men have a higher death rate from HIV compared to white men
- Limited attention is given to minority men as part of health disparities research

MUSC TRANSDISCIPLINARY COLLABORATIVE CENTER IN PRECISION MEDICINE AND MINORITY MEN'S HEALTH

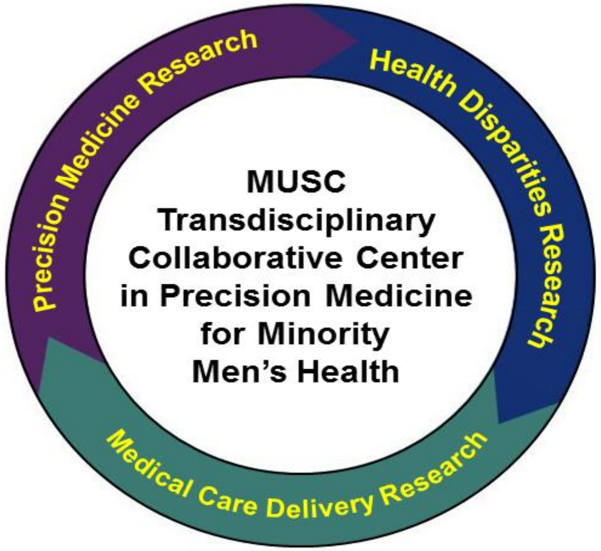

MUSC TRANSDISCIPLINARY COLLABORATIVE CENTER IN PRECISION MEDICINE AND MINORITY MEN'S HEALTH

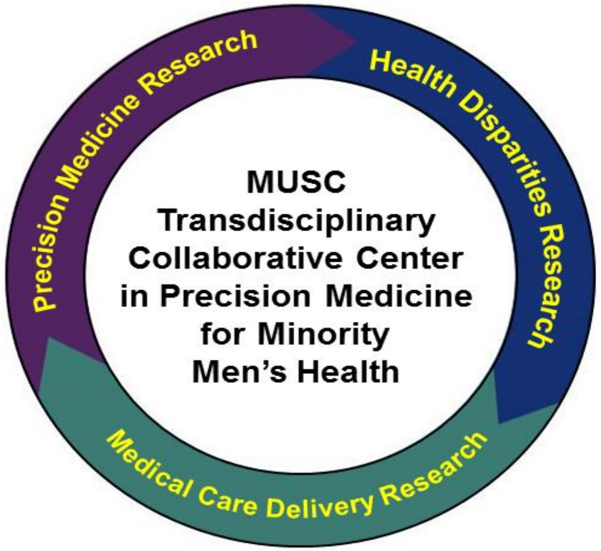

- **Low Country AHEC**
- **National Black Leadership Initiative on Cancer**
- **Hope Institute, LLC**
- **Southeast Health Equity Council**

Transdisciplinary Collaborative Center in Precision Medicine and Minority Men's Health

Multi-PIs/Admin
Hughes-Halbert, Lilly, & Ethier

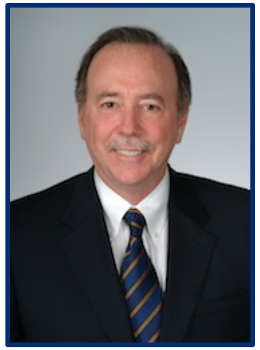

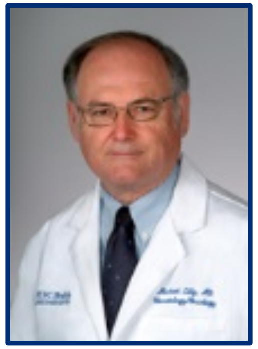

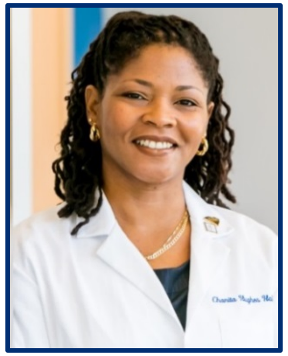

Data Integration
Lenert, Obeid

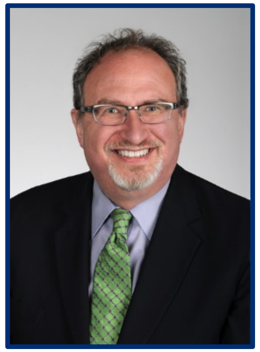

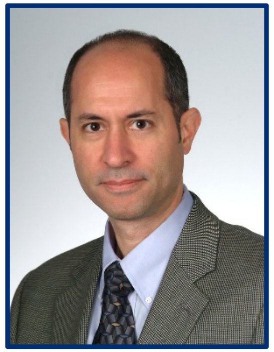

Dissemination & Implementation
Melvin, Magwood

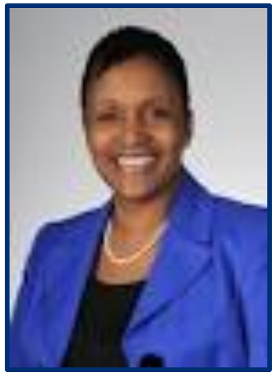

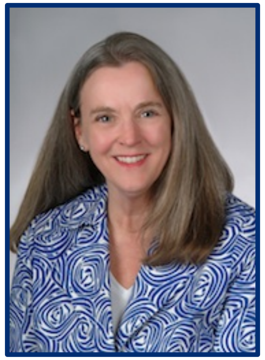

MUSC TRANSDISCIPLINARY COLLABORATIVE CENTER IN PRECISION MEDICINE AND MINORITY MEN'S HEALTH

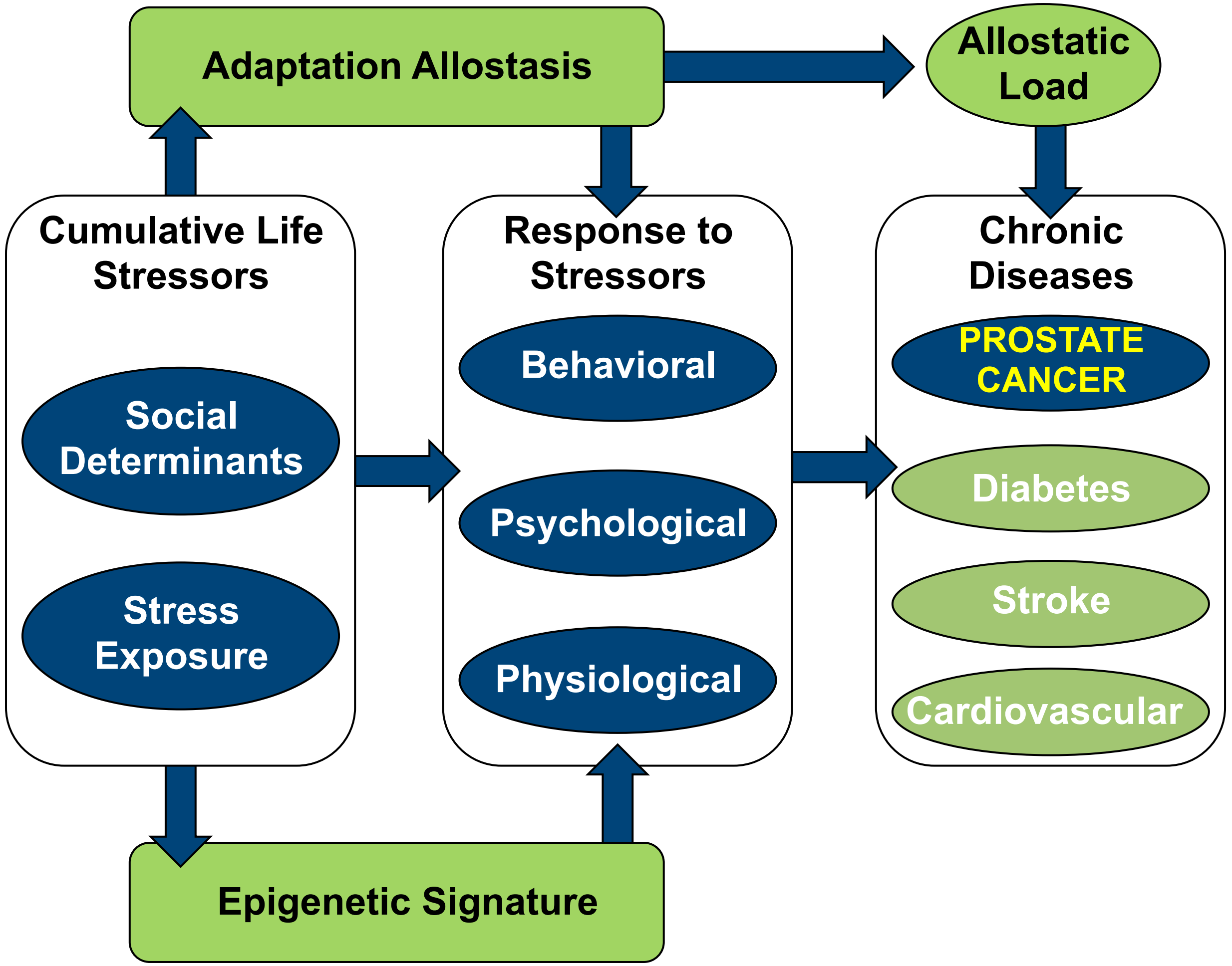

- Minority men experience unique acute and chronic stressors
- Social and psychological stressors impact biological processes involved in the initiation and progression of disease
- Allostatic load is a marker of how much social and psychological stressors impact biological functioning
- Racial disparities in allostatic load exist
- Need to understand the effects of allostatic load on disease processes and outcomes

PRECISION MEDICINE AND DISPARITIES

- Disease has a significant clinical and public health impact
- Priority condition for community residents and other stakeholders
- Biological, clinical, and public health relevance to other chronic conditions

Article

Ever and Annual Use of Prostate Cancer Screening in African American Men

American Journal of Men's Health
1–9
© The Author(s) 2015
Reprints and permissions:
sagepub.com/journalsPermissions.nav
DOI: 10.1177/1557988315596225
ajmh.sagepub.com
SAGE

Chanita Hughes Halbert, PhD[1,2], Sebastiano Gattoni-Celli, MD[1,2], Stephen Savage, MD[1,2], Sandip M. Prasad, MD[1,2], Rick Kittles, PhD[3], Vanessa Briggs, MBA[4], Ernestine Delmoor, MPH[5], LaShanta J. Rice, PhD[1], Melanie Jefferson, MPH[1], and Jerry C. Johnson, MD[6]

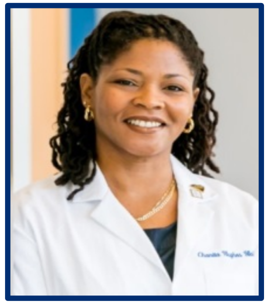
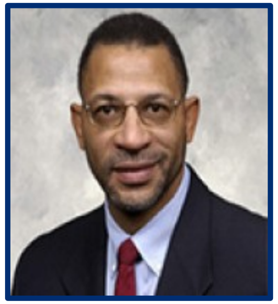
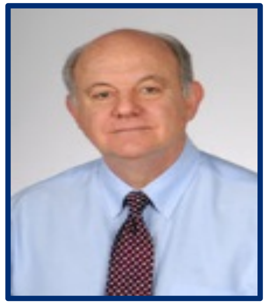
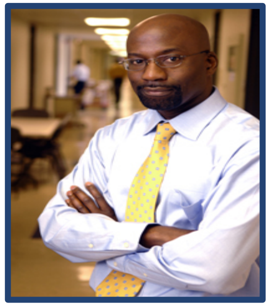
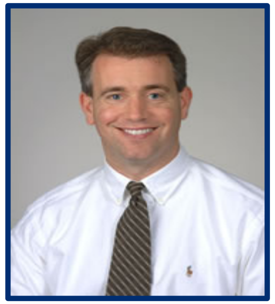

NATIONAL® CANCER INSTITUTE

Triumphant Living Collaborative
West Philadelphia Consortium to Address Disparities

Health Outcomes Research

Racial Differences in Quality of Life Following Prostate Cancer Diagnosis

Chanita H. Halbert, James Coyne, Benita Weathers, Brandon Mahler, Ernestine Delmoor, David Vaughn, S. Bruce Malkowicz, David Lee, and Andrea Troxel

[illegible]

Psycho-Oncology
Psycho-Oncology (2009)
Published online in Wiley InterScience (www.interscience.wiley.com). **DOI**: 10.1002/pon.1574

Sociocultural determinants of men's reactions to prostate cancer diagnosis

Chanita Hughes Halbert[1]*, Glenda Wrenn[2], Benita Weathers[3], Ernestine Delmoor[4], Thomas Ten Have[5] and James C. Coyne[1]
[1]*Department of Psychiatry and Abramson Cancer Center, University of Pennsylvania, Philadelphia, PA, USA*
[2]*Department of Psychiatry and Robert Wood Johnson Clinical Scholars Program, University of Pennsylvania, Philadelphia, PA, USA*
[3]*Department of Psychiatry, University of Pennsylvania, Philadelphia, PA, USA*
[4]*Philadelphia Chapter, National Black Leadership Initiative on Cancer, Philadelphia, PA, USA*
[5]*Department of Biostatistics and Epidemiology, University of Pennsylvania, Philadelphia, PA, USA*

CENTERS FOR POPULATION HEALTH
CPHHD
AND HEALTH DISPARITIES

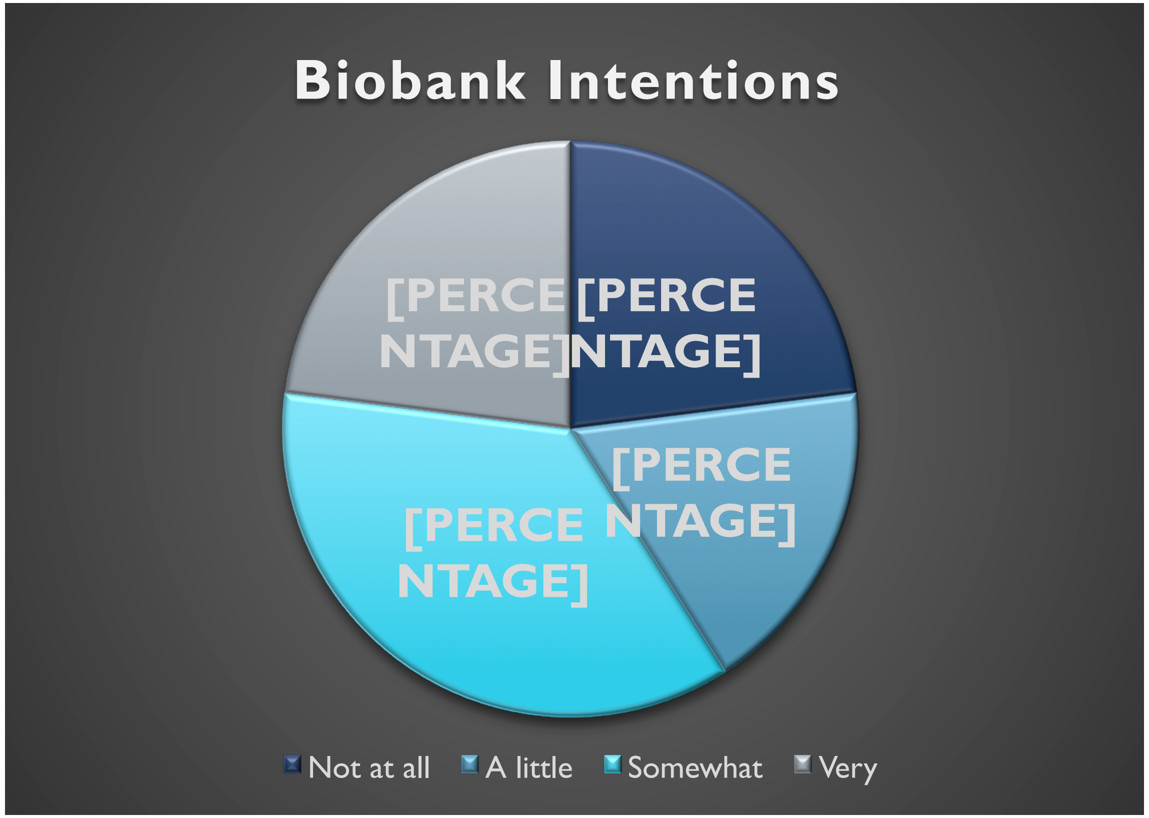

McDonald et al., Public Health Genomics, 2014

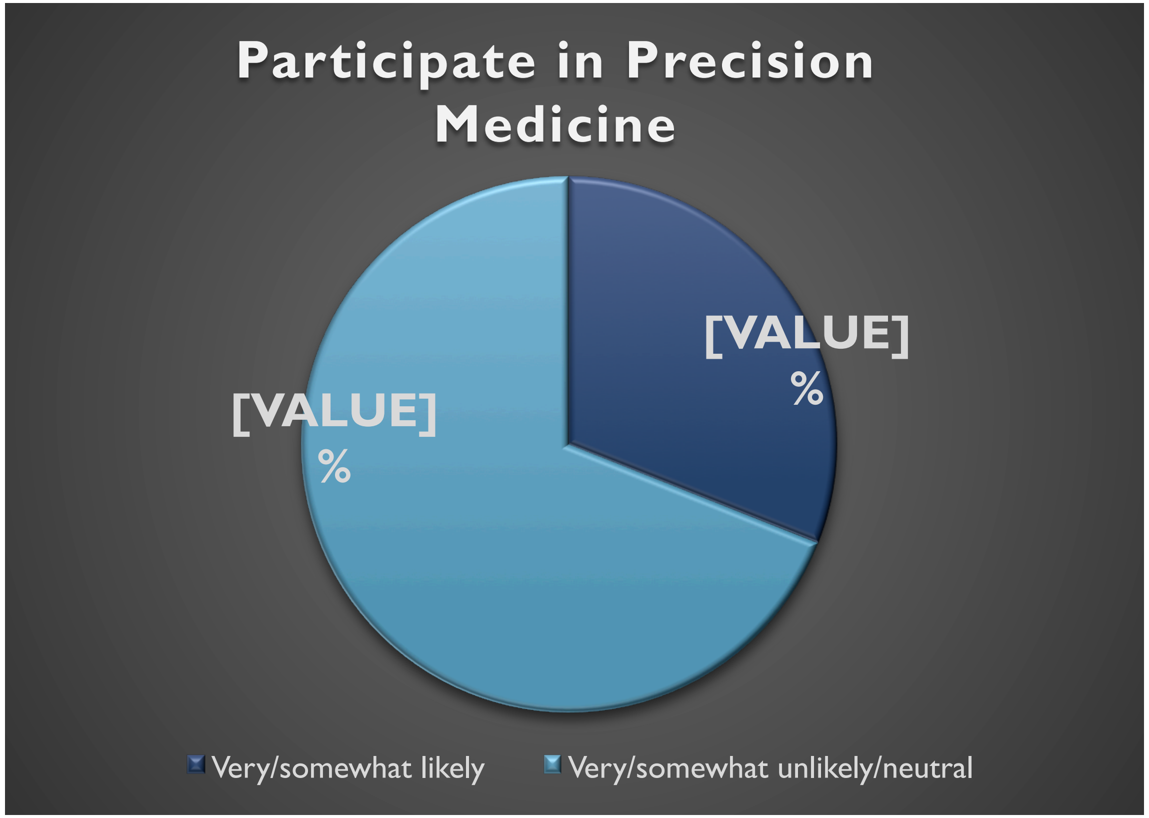

Halbert et al. PLOS One, 2016,

OVERVIEW OF CENTER PROJECTS

- **Project 1:** Sociobiological Responses to Stress in Prostate Cancer Survivors *(M. Lilly, C. Hughes-Halbert, Co-Leads).*
  - *Examine the effects of stress reactions and allostatic load on immune responses to a prostate cancer vaccine among survivors at high risk for recurrence*
- ***Project 2:*** Defining an Integrated Allostatic Load Index with Immune and Tumor Microenvironment Factors *(R. Drake and J. Wu, Co-Leads)*
  - *Identify novel biomarkers for prostate cancer based on metabolites, glycans, and immune modulators in clinical prostate cancer biopsy samples, prostatectomy tissues, tissue microarrays.*
  - *Characterize the distribution of these biomarkers based on allostatic load, racial background, social factors, and psychological characteristics*
- ***Project 3:*** Integrating Genomic and Sociobiological Data to Inform the Development of Prostate Cancer Treatment *(S. Gattoni-Celli, Lead)*
  - *Evaluate the effects of Vitamin D supplementation on molecular changes in prostate cancer tissue*
  - *Examine the effects of Vitamin D on HPA axis functioning and allostatic load biomarkers to determine individual response to supplementation*

CONSORTIUM CORE

- Form a multi-regional infrastructure to provide the direction, priorities and governance of the TCC to promote health equity among minority men through precision medicine

- Identify priorities for research in precision medicine and minority men among diverse academic, community, clinical, and public health stakeholders

- Implement and monitor pilot project research to address minority men's health in precision medicine

- Translate findings from TCC research into clinic and community-based practices to address racial disparities among minority men

IMPLEMENTATION CORE

- Develop materials and methods to determine the needs, readiness, and capacity of stakeholders across multiple regions to implement and adopt precision medicine approaches into clinical care and public health practice.
- Identify ethical, legal, and social issues related to linking and integrating data on biological, social, psychological, behavioral, and clinical factors and develop recommendations to address these issues.
- Deliver Evidence Academies across the regions in the MUSC TCC Consortium to actively engage diverse stakeholders in the development of best practices for implementing precision medicine interventions into practice.

DATA INTEGRATION CORE

- Create a standards-based resource using nationally-recognized tools including REDCap and Informatics for Integrating Bench to Bedside(i2b2), for integration of data on the tumor microenvironments derived from proteoglycan analyses of prostate tissue and clinical studies of impacts of glucocorticoids on pathways for vitamin D effects.

- Develop natural language processing-based tools to extract discrete details on social stressors from clinicians' notes and merge these data with clinical data within the i2b2 environment.

- Integrate clinical and experimental data into longitudinal patient records to expand data sets to represent the chronological order of significant clinical and social events surrounding the timing of a critical cancer diagnosis.

- Use data mining strategies to determine the temporal links between allostatic load and disease risk and outcomes.

How do you integrate these activities to facilitate academic and community engagement?

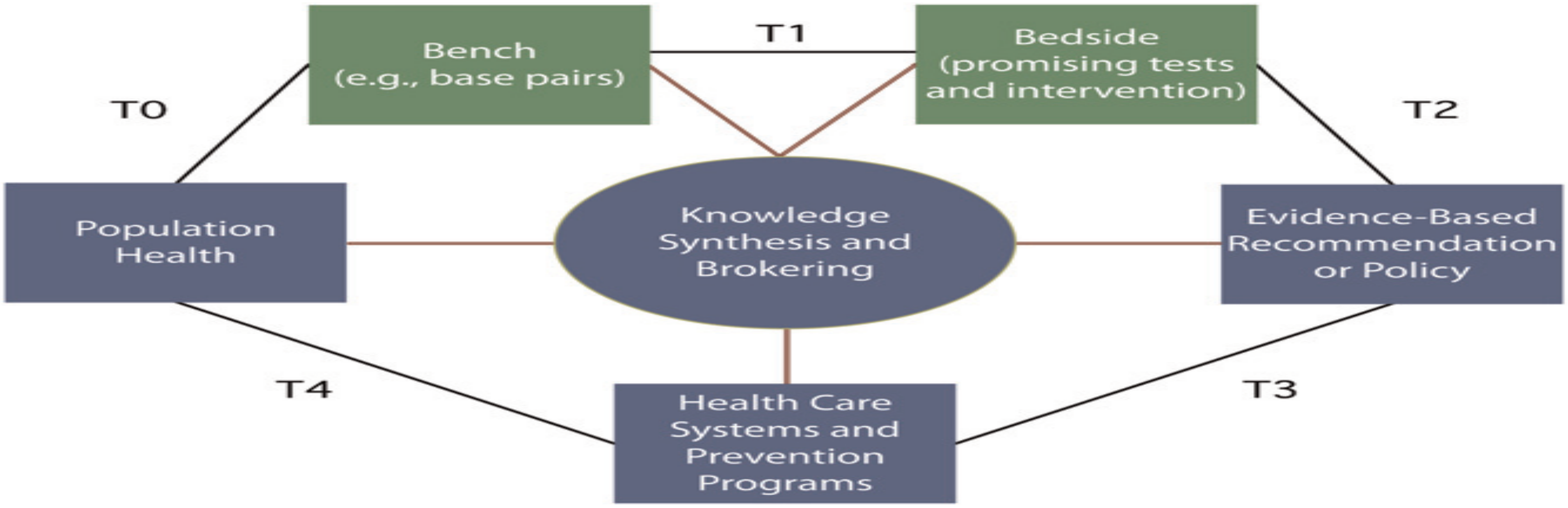

Source. Adapted from Khoury et al.[2]

FIGURE 1—A framework for multidisciplinary research in genomics and health beyond bench to bedside, with green representing the first phase of translation (T1) and blue representing the second phase of translation (T2–T4), with a feedback loop to basic science discoveries (T0).

Khoury, Gwinn, Bowen, Dotson. Am J Public Health, 2012

ADOPT DEFINITION OF PRECISION MEDICINE

Precision medicine is an emerging approach for preventing and treating diseases that uses biological, environmental, and lifestyle information to help develop personalized treatments and procedures. By combining this information, the delivery of medical care will be more personalized as doctors and patients will be able to co-develop targeted plans for prevention, detection and treatment. The goal for precision medicine is to provide the right medical care in the right dose to the right patient at the right time.

ESTABLISH GOALS FOR COMMUNITY ENGAGEMENT

The MUSC TCC in Precision Medicine and Minority Men's Health will engage diverse stakeholders in activities to ensure that groups, organizations, and individuals:

- Are knowledgeable, aware, and informed about precision medicine
- Have the capacity and resources to apply and/or use precision medicine as part of their health care or clinical practice
- Have sufficient information to make informed decisions about participating in precision medicine research and/or referring patients/members for participation in studies
- Understand how to assure that the use of precision medicine does not exacerbate racial and ethnic disparities in health care and outcomes

COMMUNITY ENGAGEMENT STRATEGIES

Precision Medicine Survey: Identify priorities, concerns, and preferences of patients about precision medicine

Organizational Capacity for Precision Medicine: Identify resources and readiness for precision medicine

Evidence Academy: Dissemination strategy that focuses on enhancing knowledge about a health-related topic and developing tailored implementation strategies

REGIONAL IMPLEMENTATION TEAMS

Composed of stakeholders in each region who are critical to promoting, implementing, and adopting precision medicine. Team members will represent individual patient, health care providers/teams, organizational, community, and policy groups.

- Stakeholder interviews and review of draft documents and processes in Year 2
- In-person regional meetings (Evidence Academies in Years 2 and 4)
- Development and implementation of regional plans during years 2 & 3 using knowledge gained through TCC research to inform decisions and actions to change policies, programs and/or practices affecting the uptake and use of these interventions in their region and particularly among minority men

ISSUES FOR ACADEMIC-COMMUNITY PARTNERSHIPS

- Determining who are the right stakeholders?
- Deciding on when to expand and limit partnerships?
- Precision medicine versus basic health care services?
- Administrative coordination and integration of groups and activities

MUSC TRANSDISCIPLINARY COLLABORATIVE CENTER IN PRECISION MEDICINE AND MINORITY MEN'S HEALTH

- Claudia Baquet, MD
- Bettina Beech, DrPH
- Kathleen Cartmell, PhD
- Ernestine Delmoor, MPH
- Richard Drake, PhD
- Sebastiano Gattoni-Celli, MD
- Stephen Ethier, PhD
- Chanita Hughes-Halbert, PhD
- Melanie Jefferson, PhD
- Les Lenert, MD
- Michael Lilly, MD
- Gaynell Magwood, PhD
- Cathy Melvin, PhD
- Raymond Samuel, MD
- Stephen Savage, MD
- Ian Thompson, MD
- Jennifer Wu, PhD

Advancing Trust to Engage Diverse Participation in Research: Community Dialogue and Partnerships

Karriem S. Watson

kswatson@uic.edu

Senior Research Scientist, University of Illinois at Chicago Cancer Center, Director of Community Engagement and Implementation Sciences

Chicago, Illinois

Abstract

In this presentation, Dr. Watson discusses the value of dialogue, conversation, and listening as he unpacks the precision engagement concept.

[*Karriem S. Watson*] Good afternoon. I have to give some disclosures. My name is Karriem Sedat, but somehow, I am the son of an Apostolic Deacon and a COGIC [*Church of God in Christ*] momma. If any of you know the black church you probably know where I am going with this. As I was thinking about this conversation, of course the title of my topic is "Advancing Trust to Engage Diverse Participation in Research: Dialogue and Partnerships, but in the tradition of the black church, if I had a sub-title, it would be, "Who goin' check me, boo?" And if I had to have a scripture, it would be Matthew 18 verse 20: "For where two or three are gathered in my name, I am in the midst of them." That's where I start my work, because when I think about the concepts of advancing trust, for us, the University of Illinois Cancer Center, it starts with a conversation, and the reason it starts with a conversation and dialogue at the University of Illinois Cancer Center is because we are made up of members where the conversation starts with us first. I stand here, yes, as a researcher, and yes, as a community-engaged researcher, very passionate about precision medicine, precision health, and precision engagement, but I am also the son of Bertha Watson, who lost her battle to breast cancer when I was seven years old. I am also the brother of Maryanne Landiham, who got breast cancer some 27 years later. I am also the brother of Tommy Duane Watson, who got colon cancer before the age of 50. I am also the son of Tommy Watson, who died of complications from colon cancer and diabetes. I bring all of that to the table, and that lends to the fact of us having this conversation about precision engagement. I have to give you those disclosures because that is where I start. I am from Chicago. I am from Michigan, but I live in Chicago, and that whole concept of, "Who goin' check me, boo?" is real in Chicago. When you do this work right, you get yourself some good community partners, and they will check you. Miss Regina Greer Smith is over there nodding her head. She don't even live in Chicago anymore and checkin' me, right!? We do dissemination. One of the things we do in this whole concept of dialogue is we do diverse dissemination - how we get the message out. We just bought some time on a radio show, so every first Sunday you can tune in on iHeart Radio, WVON 1690, and Regina listens and she emailing me – "Karriem, y'all ain't talkin' about the right thing! Y'all gotta talk about this! You gotta address this this way!" I am

making light of it, but I am serious. I can show you text messages of community partners right now texting me saying, "Hey, I am addressing this right now," letting me know they heard me in D.C. cause I'm a little out of the office, and let them know when I get back, ‘cause they want to talk to me about something.

[SLIDE 2] When I say, "Why dialogue?" Because you have to. Who should be at the table? I am going to talk about who should be at the table. I am going to talk about what has already been said and how can we keep the conversation going. When we talk about "precision engagement," I think we have lost the art of conversation. I think we have lost the art of listening. We have made it so academic that those natural tendencies that we naturally have as humans, we sometimes lose those. But I think with leaders like Al Richmond, he is bringing us back to what we need to do. (Another disclosure - I am Board member of the CCPH [*Community Campus Partnerships for Health; Al Richmond, Executive Director*]. Lots of disclosures.) And I am going to walk you through a brief story of what can happen when, as Usha [*Usha Menon, PhD, RN, FAAN Associate Dean for Research & Global Advances, UA College of Nursing*] said, you DREAM BIG. I am going to talk to you about what can happen when you have an amazingly bright and innovative young person who is not jaded by research and jaded by academia yet. So, yeah, I am going to speak that into existence, right? I told y'all I was apostolic. She is not jaded. I also want to tell you what happens when you pair her with a trusted community partner. What happens is that you get the story of Miss Rose Marie. [SLIDE 3] Miss Rose Marie is an over-27-year breast cancer survivor, and she has been at several tables. One of the tables she was fortunate to be at is the table with the DOD, the Department of Defense, Grant. If you have been a part of the Department of Defense Grants, you know that in order to get some of those research grants, you have to have community patients at the table to get those. As a result of being at that table, she immediately went from being a survivor to being an advocate. Now, I am fortunate enough to have Miss Rose Marie in my life to be one of those "who goin' check me" people, right? So, Dr. Winn, our cancer center Director [*Robert A. Winn, MD, Director, University of Illinois Cancer Center*], had this vision of a cancer ... we are changing the name of my office from The Office of Community-Engaged Research and Implementation Science ... we are taking out the "E" and adding an "I" to The Office of Community Involvement and Implementation Science. We are advancing "engagement" to "involvement," right? Miss Rose Marie's story was – “I was the only one at the table.” When we found out that we were one of the awardees for the "All of Us" research program, I knew that, while I was excited about precision medicine, there had to be some engagement way before. I had to capitalize on what we had already done in Chicago and leverage a lot of that trust that was already there, because we did not have time to do a lot of the catch-up work that we needed to do in our communities

around precision medicine. I went to our Community Advisory Boards at the UIC-CCTS [*University of Illinois at Chicago Center for Clinical and Translational Science*] and I ran this by them. One of our community partners, Marcus Murray, from Project Brotherhood, pulled me aside and said, "Yo, Karriem, now you mean to tell me you are going to have black men in Chicago to give their saliva to you?" I was like, "Yeah, Marcus, that's what I'm gonna do." He said, "You know that sound crazy, right?" I was like, "It sounds crazy, Marcus, but if I can get that saliva and I can connect that saliva with West African ancestry, and then I know that men with West African ancestry actually have a greater risk of prostate cancer, then I can connect that with some work that Dr. Rick Kittles, an African-American geneticist, has been doing, and we might be able to address this mortality gap that we have in African-American men." Marcus was like, "Aight, say that then." He said, "Lead with that, but don't lead with, 'I'm going to collect your saliva, and I'm going to do blood samples, and I may actually collect some hair.'" So, we have to acknowledge that.

We do this work, well , because we have to capitalize on precision engagement. We have to be asking questions that are relevant to community members, and at that pre-engagement, y'all. So, from that conversation was sparked in the "All of Us" research program. I would like to discuss the importance of beginning to catalog best practices. [SLIDE 4] One thing that we are hearing from community members is this concern of conversation fatigue, the same thing being said in different venues without moving the conversation forward. We must be better at noting who has been saying this and learning what we heard from them. Here are two pictures above as examples of two different groups, both community, and both initiated by community stakeholders and partnered with industry and academic partners similar conversations of trust and have convened community members with successful outcomes. In May 2017, CCPH partnered with UI Cancer Center and Northwestern University Cancer Center for Community Dialogue and in July 2017 we partnered with Sister's Network. We had an open, honest conversation about clinical trials with the Sisters Network Chicago These are examples of the accomplishments and instead of trying to invent new conversation, we may be able to be a part of those conversations with the community.

Now, this woman named Nicole Fisher, who is one of our patient stakeholders. They were worried about the precision engagement. Community members said that we had to organize a place. I'm thinking I'm doing the Town Hall meetings and at the most, we might show that there are really good people around you. Nicole Fisher, I know she found us, I said, "Girl, we ain't got no money for this!" We were fortunate to partner with the family of Henrietta Lacks [SLIDE 5]. The Lacks family was engaged and we had 659 people registered and more than 500 attendees at this event [SLIDE 6]. Representatives from elected officials in attendance, ; Gennifer Geer (Senator Durbin's office), Tran Nguyen (Senator Durbin's office) Nikki Harvey – Congressman

Davis's offices, Roland Vaughn (Congresswoman Kelly's office. The University of Illinois-Chicago leadership was in attendance and joined private lunch. Several Patient Brigade Members in were in attendance and City/academic/medical institutions including: Illinois Medical District, Rush , Northwestern University Cook County HHS, Harvard/Duke, Howard Brown, Elevate, AIDS Foundation of Chicago, Howard Brown City of Chicago and Pfizer were at this event. 85% of attendees said they knew the story of Henrietta Lack. We had 49% representation of African-Americans at the Town Hall [SLIDES 7-8]and then 8% Asian 18% Hispanic or Latino. The percentages reflect each question's total responses. The denominator varies per question as not everyone respondent answered each question. Of the respondents, 76% were women and 24% were male. The final Likert question was, "Will conversations and town halls like the one today will continue to advance research?" Of the respondents, 81% strongly agreed, 16% agreed, 1% disagreed and 2% strongly disagreed [SLIDE 9]. To quote Robert A. Winn, MD , "If loving science is wrong, then I don't want to be right. But in that science, there is a framework of social justice. Science devoid of social justice may even be considered bad science."[SLIDE 10]

The conversation at the Town Hall was about trust in medical research, attendees noted they were just as likely to go have a conversation with a family member, who is saying, "I think my aunt had cancer" or "I think my momma" ... Here are a few quotes community member: "Continue to promote community awareness and engagement. Trust will come when people know who you are and what you do. Make things are clear and easy to understand as possible. Appeal to the public interests. Thank you for all the great work that you do!!!"

[SLIDE 11]

The theme that echoed from panelists to audience members was that of community trust. In order to obtain and maintain trust we must continue to facilitate conversations such as these geared towards the community. Through feedback from the community after events such as the Henrietta Lacks (HeLA) Community Dialogue we can continue these conversations in ways that are directly informed by those it seeks to service. To quote, "In conferences like this, healthcare providers should step up, acknowledge and take responsibility and assure the communities that it wont happen again. In discussion please indicate health care providers who states what change they've done and what they're planning to do in order to get the community trust again." [sic]

[SLIDE 12]

Now, to share the story where these are the two parents that lost their son. Their son was a prominent DJ, named DJ Tim Buck2 in a clinical trial, and we thought we failed them but their story gave us hope because we didn't think there were going to be any options. We now know that we are a step closer for finding a cure for kidney cancer. So, thank you. So, I have this picture here that is training the next level of stakeholder. [SLIDE 14]. It is very important to have training programs that train and identify stakeholders. It is not rocket science. If people were trying to get those students from Inglewood to the University ... I also want to acknowledge Dr. Daryl Richardson-Heron of the *All of Us* Research Program , she actually came and moderated. I wanted to thank our patient brigade and The Lacks family [SLIDE 16] they advised us. [SLIDE 15] Lastly, I have to honor the life of a major "shero" champion and we do it on the shoulders of Dr. Cynthia "Cee" Barnes-Boyd, [SLIDE 16] and try to honor her spirit and her presence and thank her for her legacy. Thank you.

Declarations

- The presenter has no financial competing interests related to this presentation.
- All authors have approved the manuscript for submission
- The content of this presentation and transcript has not been published or submitted for publication elsewhere.
- This presentation was organized by the Meharry-Vanderbilt Alliance with support from NCATS and the NIMHD of the National Institutes of Health under award number R13TR001694

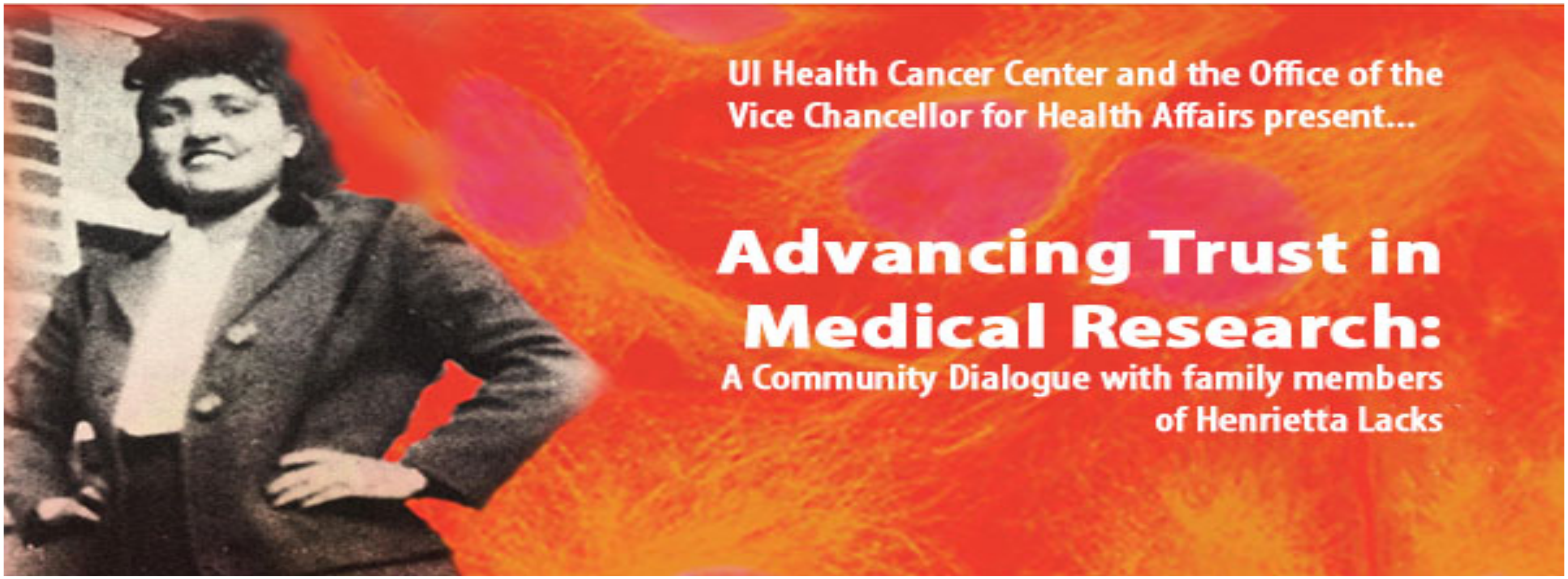

Advancing Trust to Engage Diverse Participation in Research: Dialogue and Partnerships

Dr. Karriem S. Watson, DHSc, MS, MPH-UI Cancer Center and UIC School of Public Health
Nicole Fisher-UI Cancer Center
Dr. Robert A. Winn, MD-Director UI Cancer Center and Assoc. Vice Chancellor Community Based Practice

Overview and Aims of the Conversation

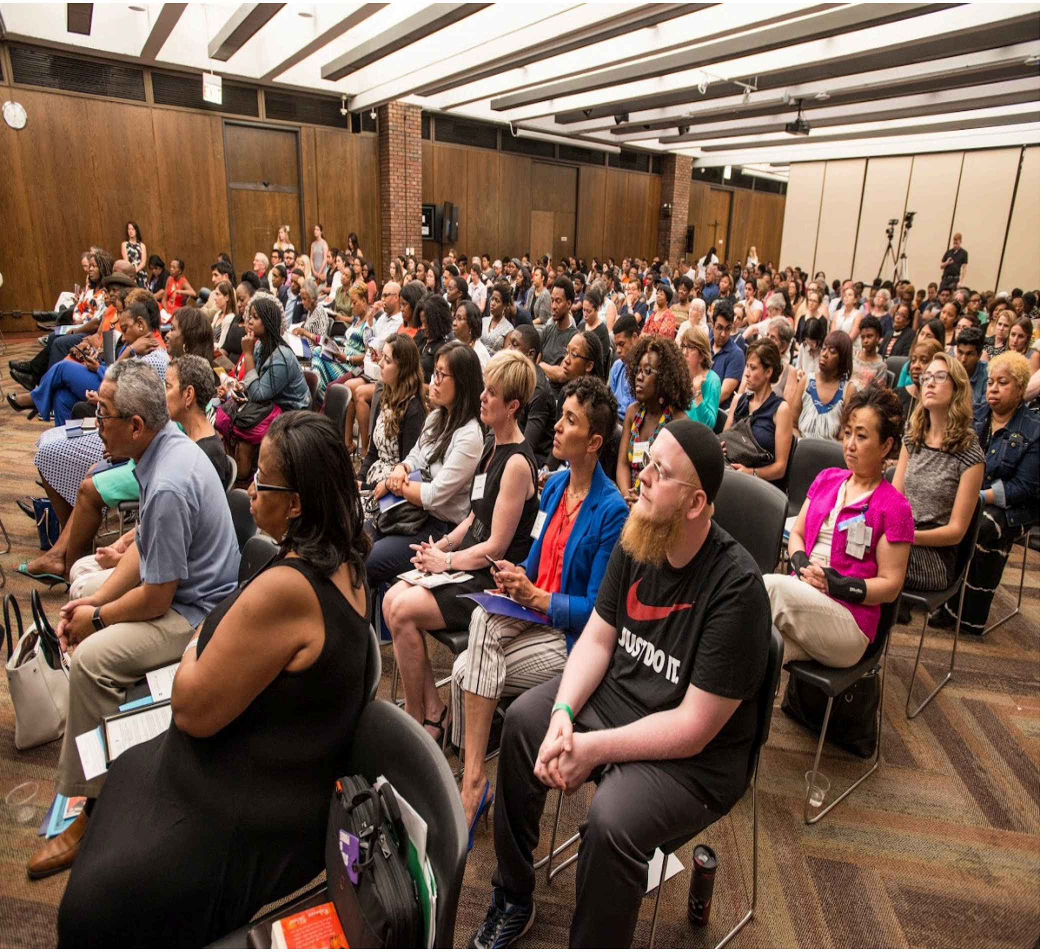

- Why Dialogue?
- Who should be at the table?;
- What has already been said?;
- How we can keep talking.

"Why" Dialogue: The Story of Ms. Rose Marie

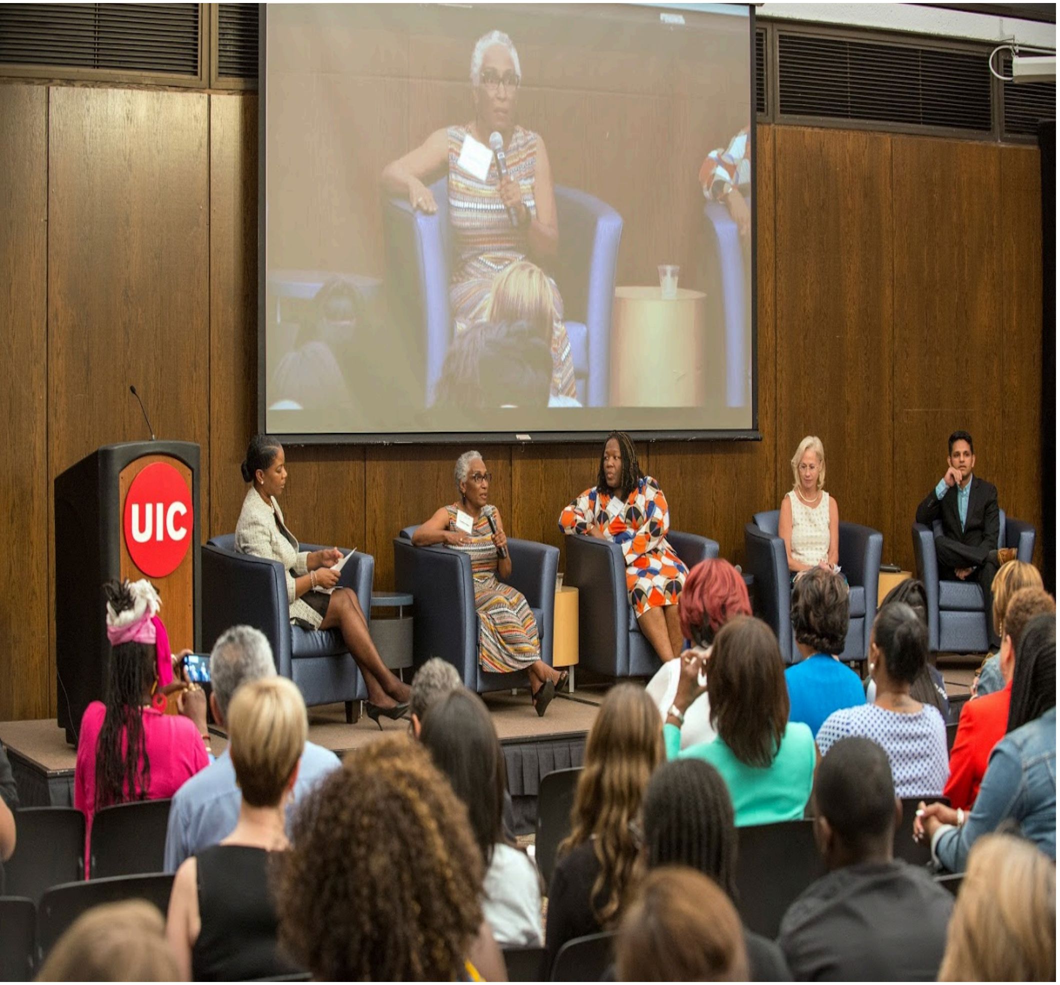

- UI Cancer Center "Patient Brigade"
- Vision of UI Cancer Center Director: Move beyond "engagement" to "involvement"
- Ms. Rose Marie's Concerns….. "*I was the only one at the table.*"

Best Practices: When Partners Talk, Trust is Built

- Community Campus Partnerships for Health: CCPH with PCORI

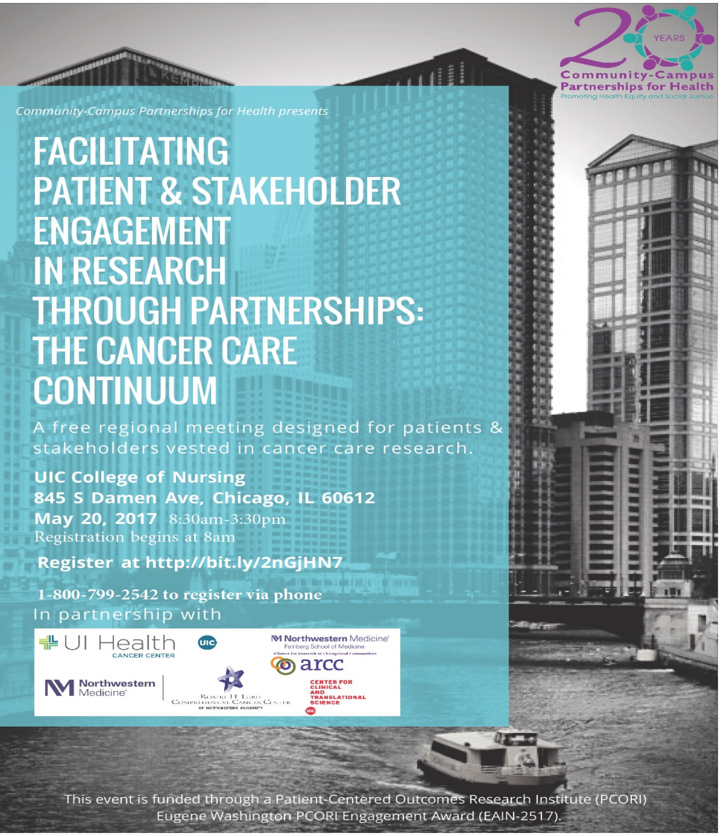

- September 2016: NIH Columbia university community workshop;
- March 2017: Engagement studio with Dr. Consuelo Wilkins' team and American Indian Health Center;
- March 2017: *On the Table* with Chicago Community Trust (20 conversations over 200 people);
- July 2017: Sisters Network Chicago: Discussion on Breast Cancer Clinical Trials in African American women

UI Cancer Center
Bench to Community

Advancing Trust by Acknowledging the Past to Inform the Future

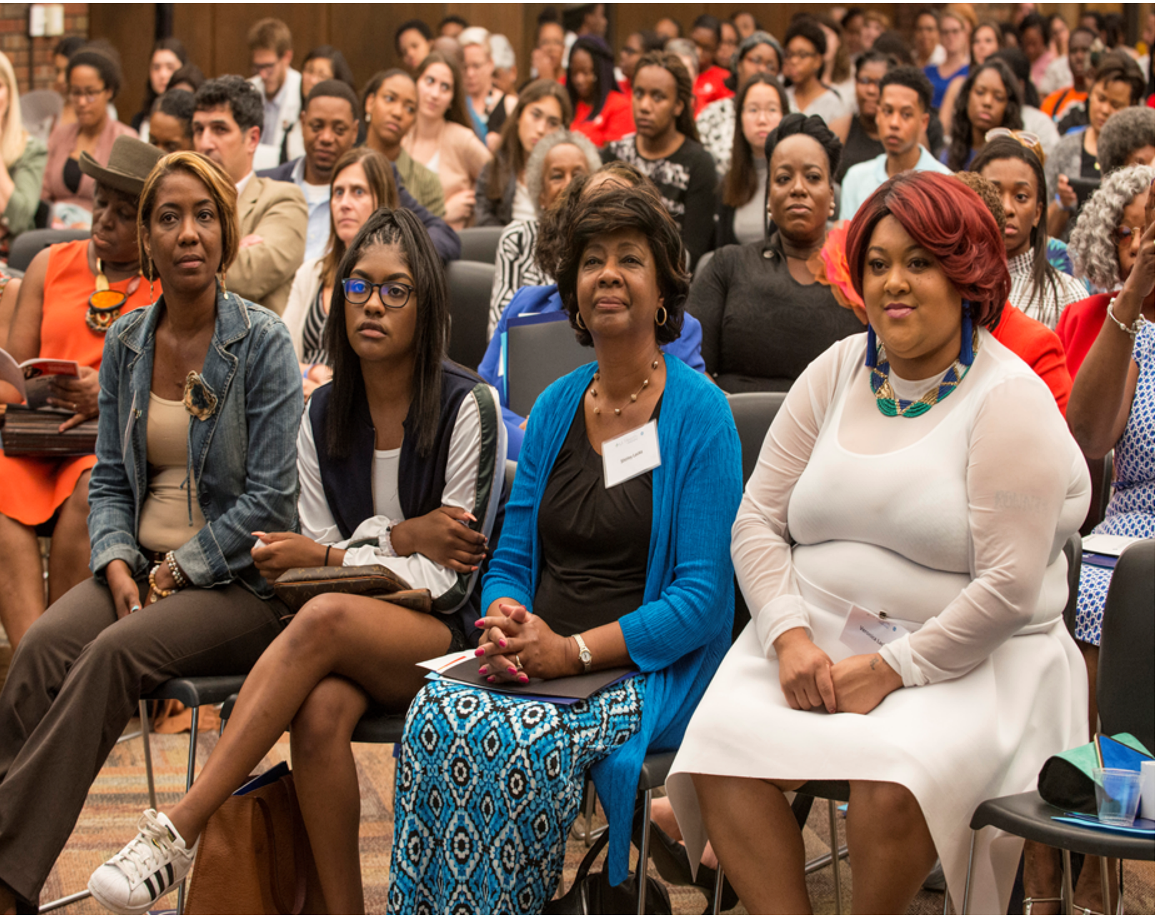

"Chicago, everything I've seen today, Mile Square, you are keeping the community engaged, you are exactly what we are about."

– Veronica Robinson, great granddaughter of Henrietta Lacks on WGN News

UI Cancer Center
Bench to Community

Diversity: Who is Invited to the Table Matters

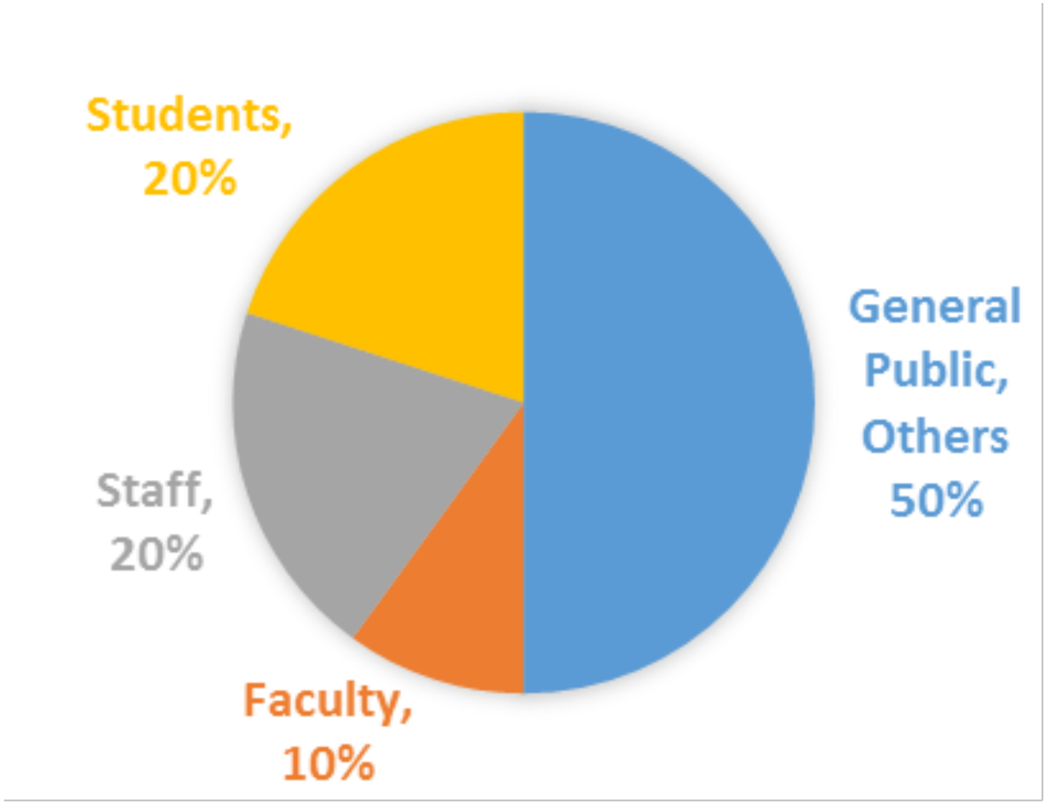

- 659 registered, approx. 518 attendees;
- 122 surveys completed (24% Response Rate); 85% Knew the Story of Henrietta Lacks.;
- 15% Introduced to the Story through the Event.

UI Cancer Center
Bench to Community

Diversity at the Table Can Advance Diversity in Research

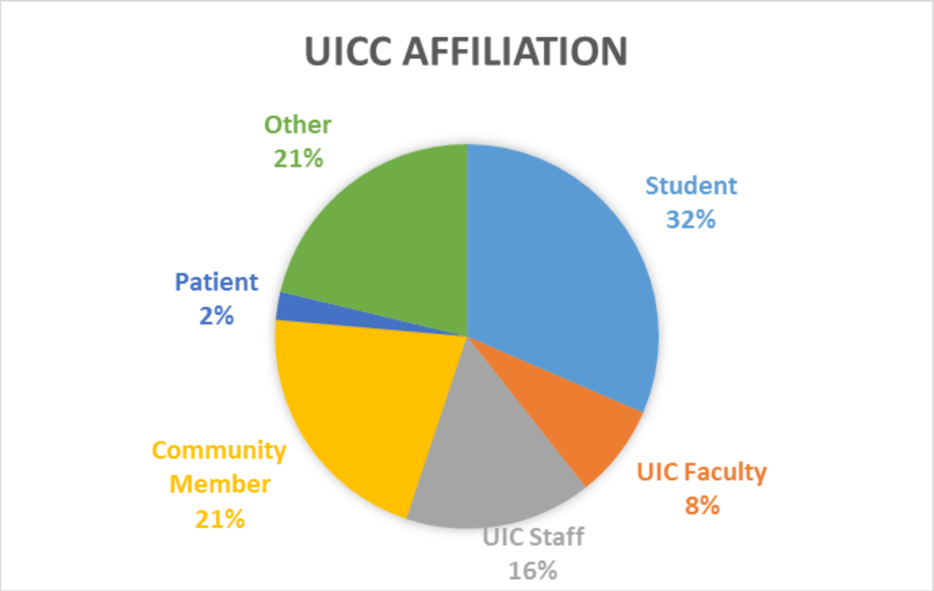

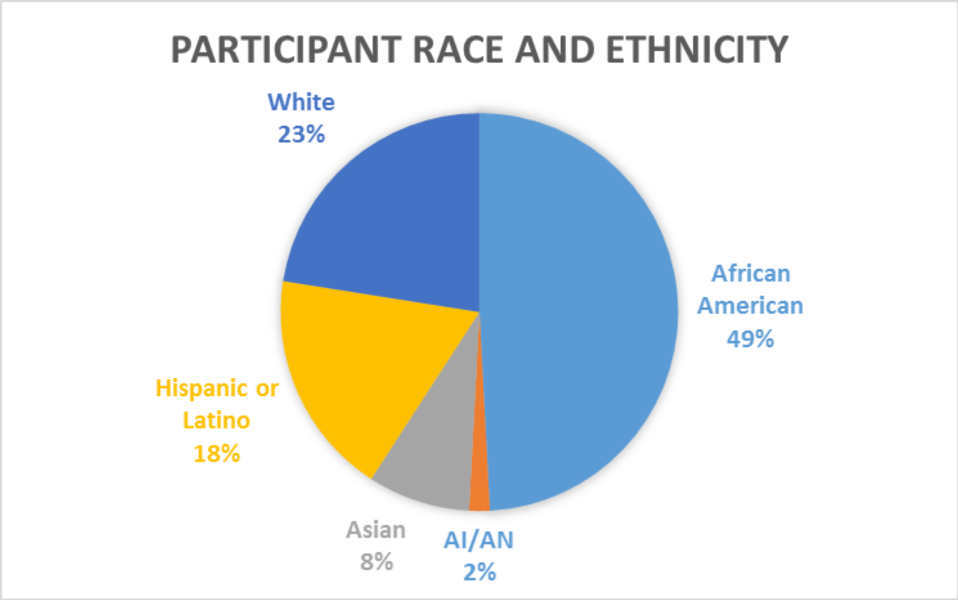

UI Cancer Center
Bench to Community

Understanding Where We are in Dialogue

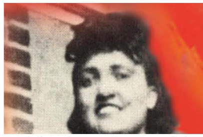

Advancing Trust in Medical Research: A Community Dialogue with the Family of Henrietta Lacks

Thank you for participating in *"Advancing Trust in Medical Research: A Community Dialogue with the Family Members of Henrietta Lacks."* The following is an optional survey regarding today's discussion. While your answers to this survey will not be directly used in research, they may be used in the future to inform as to how conversations can promote community trust in clinical research. Please only complete this survey if you are above the age of 18.

Please tell us about yourself:

Zip Code:	
Community Area:	□Back of the Yards □Cicero □Englewood □Humboldt Park □South Shore □Lower West Side □South Lawndale □North Lawndale □West Garfield Park □West Lawn □Chicago Lawn □Archer Heights □Brighton Park □Near West Side □University Village □Near South Side □West Town □River North □Near North Side □other:____________________
Race/ Ethnicity	□American Indian or Alaska Native □Asian □African American □ Hispanic or Latino □Native Hawaiian/ Pacific Islander □White
Gender	

Please describe your Affiliation (check all that apply):
□ Student; □UIC Faculty; □UIC Staff; □Community Member; □Patient; □Other______________________

1) Prior to today's conversation did you ever know the story of Henrietta Lacks?
□ Yes □ No

Please assist us in evaluating this event and planning future activities by completing this evaluation form.

		Rating scale				
		Strongly Agree	Agree	Neutral	Disagree	Strongly Disagree
2)	Today's Conversation positively informed my views on how trust in medical research can be advanced through community engagement.					
3)	As a result of today's conversation, I am more likely to talk to my family and community about the importance of clinical research.					
4)	Conversations and town halls like the one today will continue to advance research.					

5) Please indicate actions that the University of Illinois Health-Cancer Center can take to advance trust in medical research.

UI Health Cancer Center Henrietta Lacks Post Event Survey, V1.0 6/20/2017

"Please Assist us in evaluating this event and planning future activities by completing this evaluation form."

UI Cancer Center
Bench to Community

- Q2: Today's Conversation positively informed my views on how trust in medical research can be advanced through community engagement.
- Q3: As a result of today's conversation, I am more likely to talk to my family and community about the importance of clinical research.

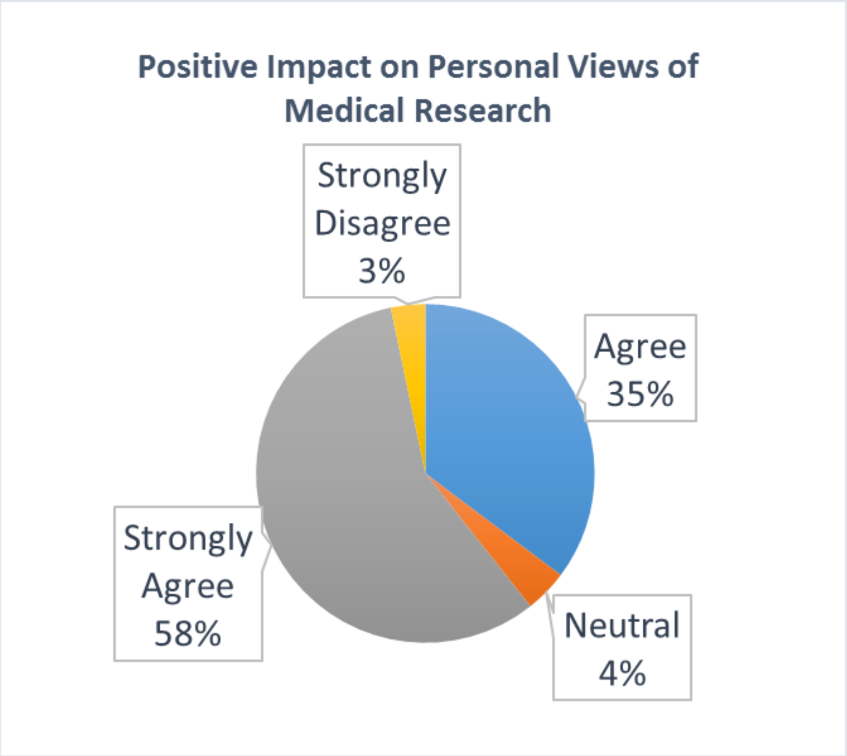

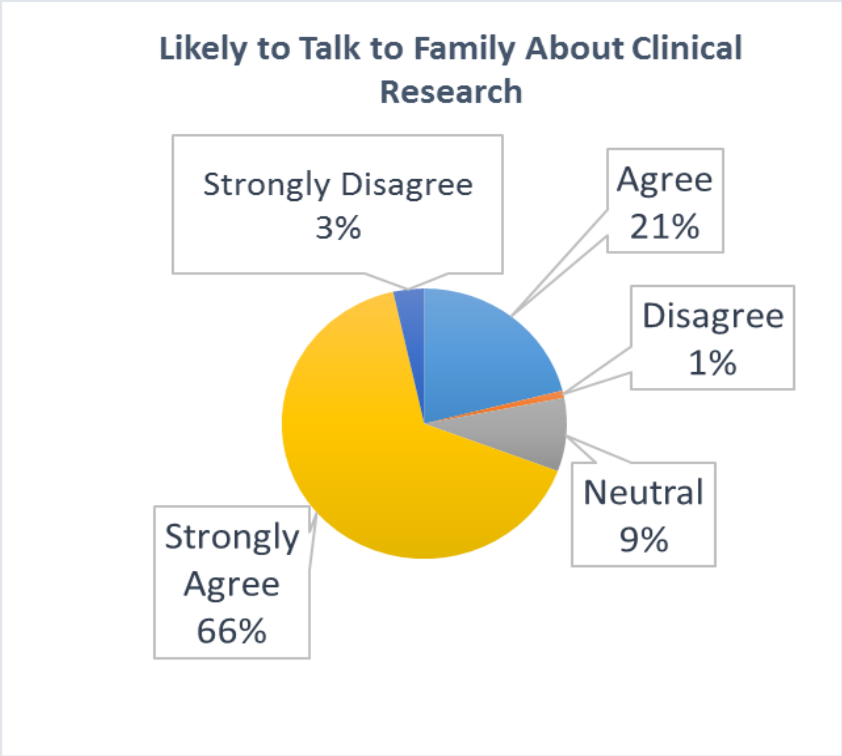

UI Cancer Center
Bench to Community

Leadership MUST Support the Partnership AND the Dialogue

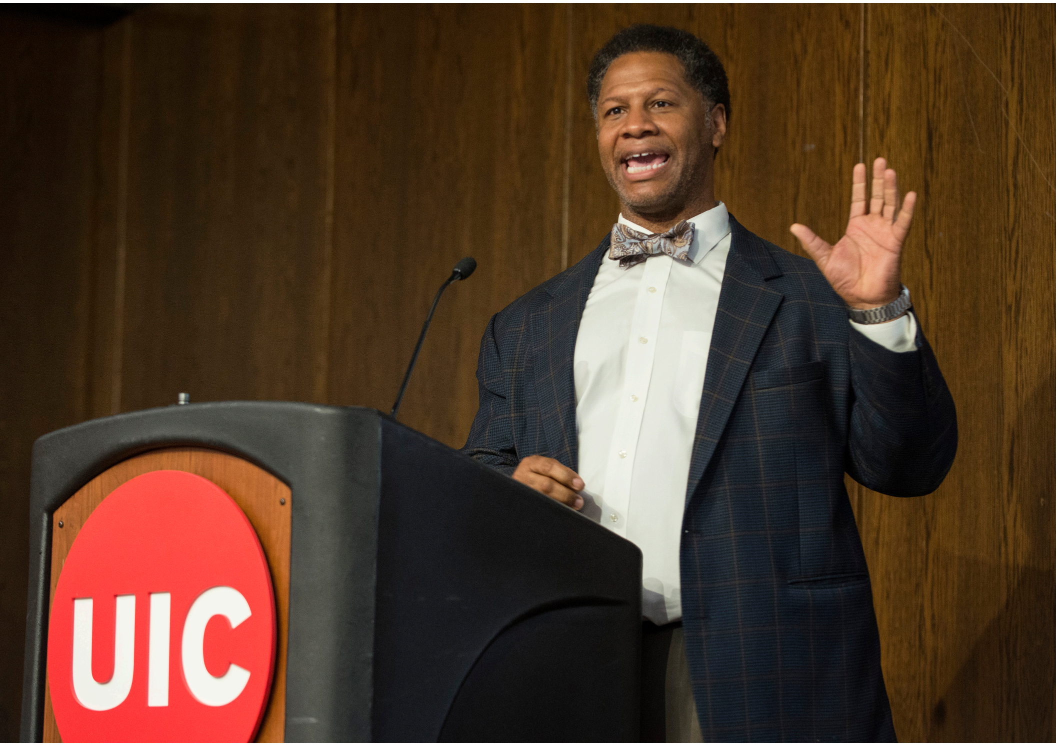

"If loving science is wrong, then I don't want to be right. But in that science there is a framework of social justice. Science devoid of social justice may even be considered bad science,"

– Robert A. Winn, MD, Chicago Magazine interview

How do we advance trust?: The Community Response

"Continue to promote community awareness and engagement. Trust will come when people know who you are and what you do. Make things are clear and easy to understand as possible. Appeal to the public interests. Thank you for all the great work that you do!!!" [sic]

- Community Member

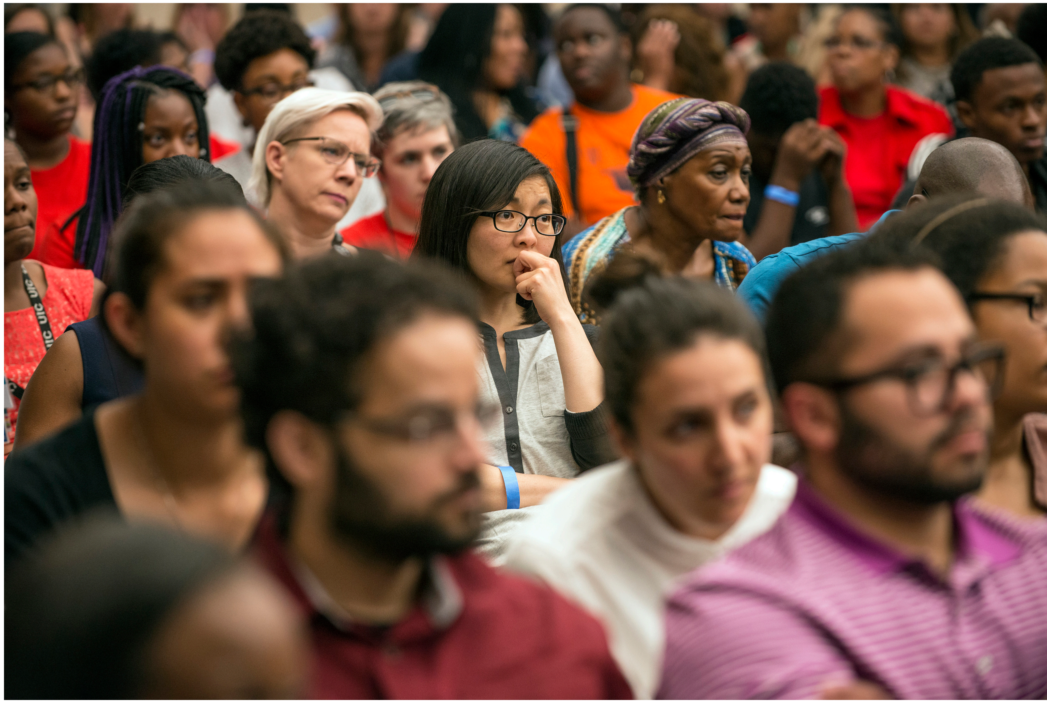

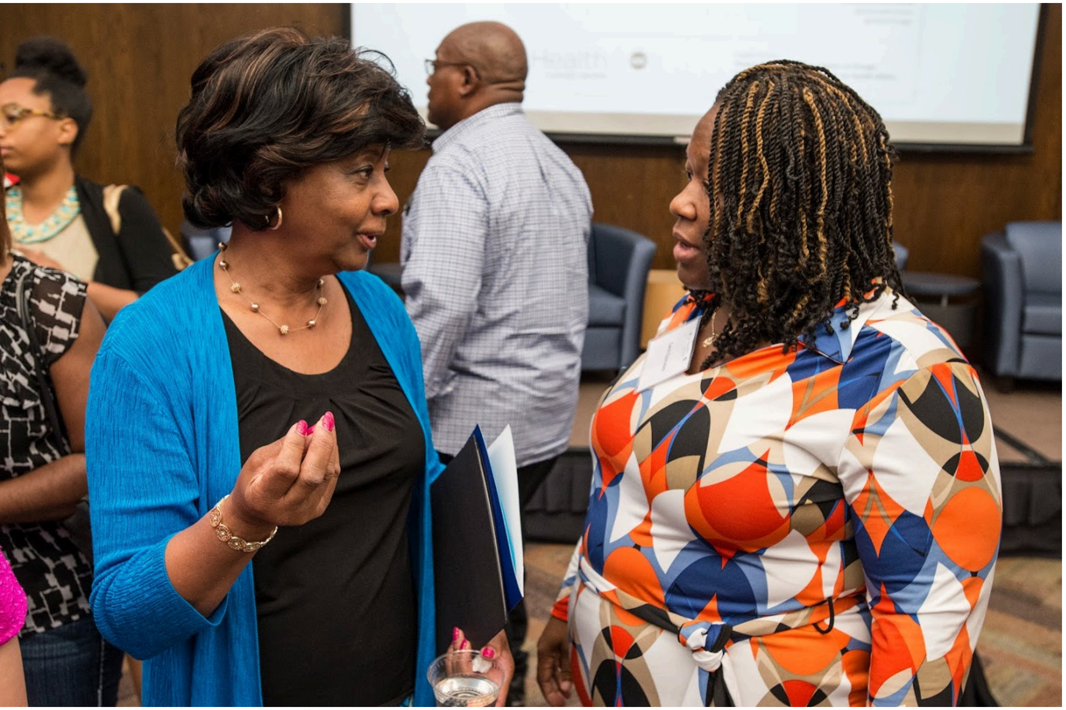

"In conferences like this, healthcare providers should step up, acknowledge and take responsibility and assure the communities that it wont happen again. In discussion please indicate health care providers who states what change they've done and what they're planning to do in order to get the community trust again." [sic]

– Community Member

Why Diversity in Clinical Trials Matters to Us: The Story of DJ Tim Buck2

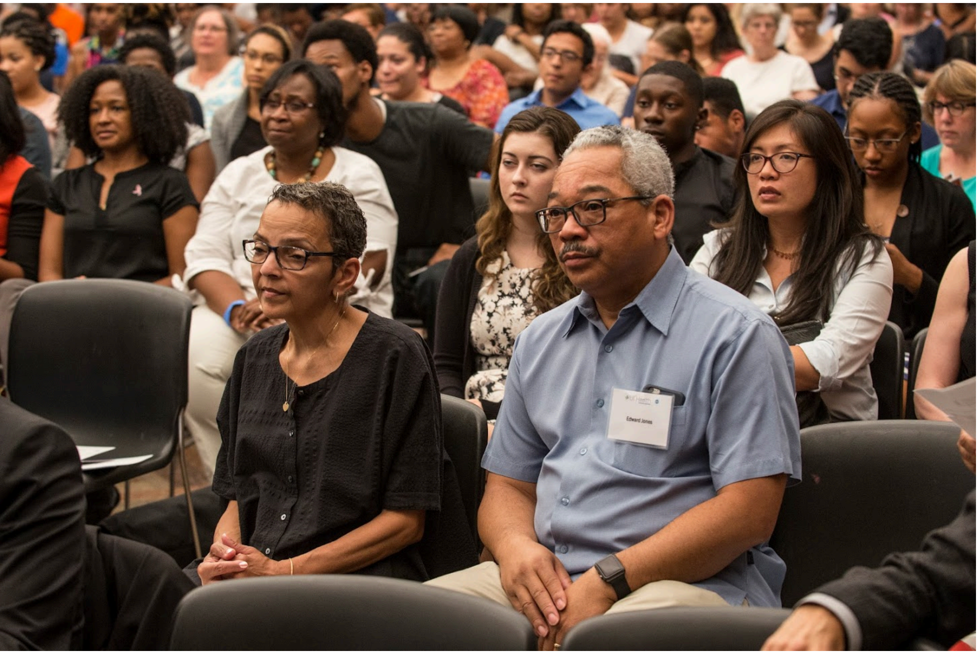

"Your struggle is my struggle, and I'm here to fight with you. To the ones with too much pride, and don't like going to the doctor because they are too proud and can handle it all, I have high hopes that my journey and story brings you back down to earth."

- Timothy Francis Jones
AKA DJ Tim Buck2

UI Cancer Center
Bench to Community

Advancing Trust Through Students, Training, Committed Leadership and Patient Partners

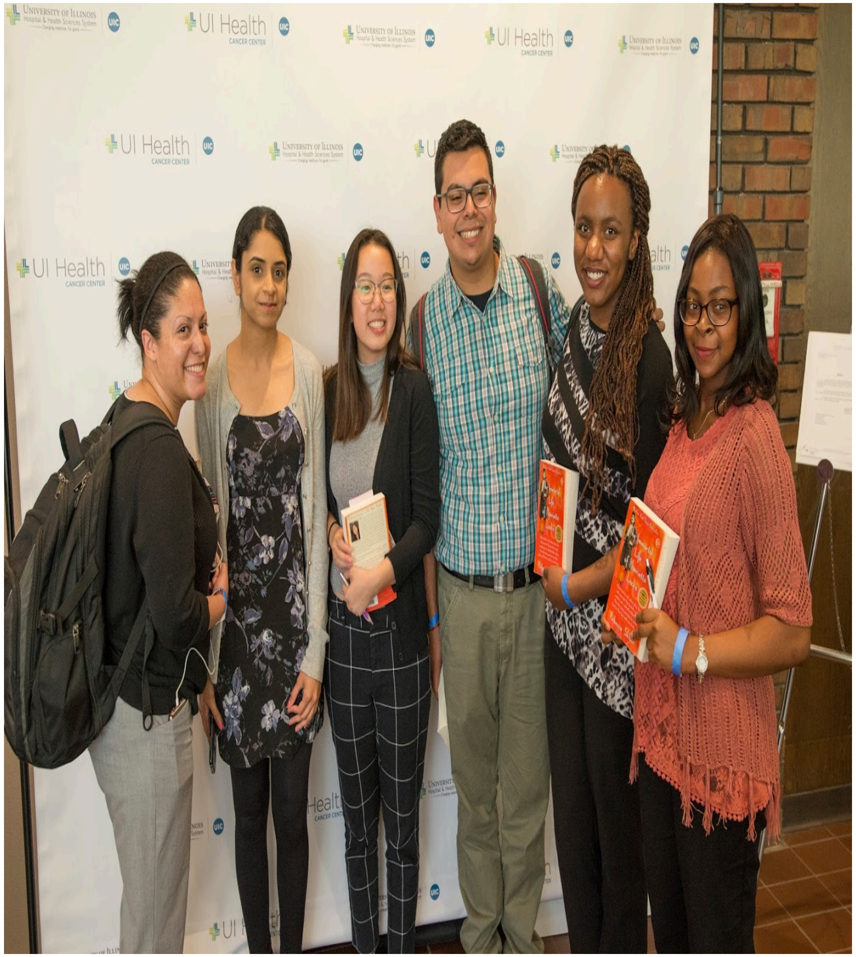

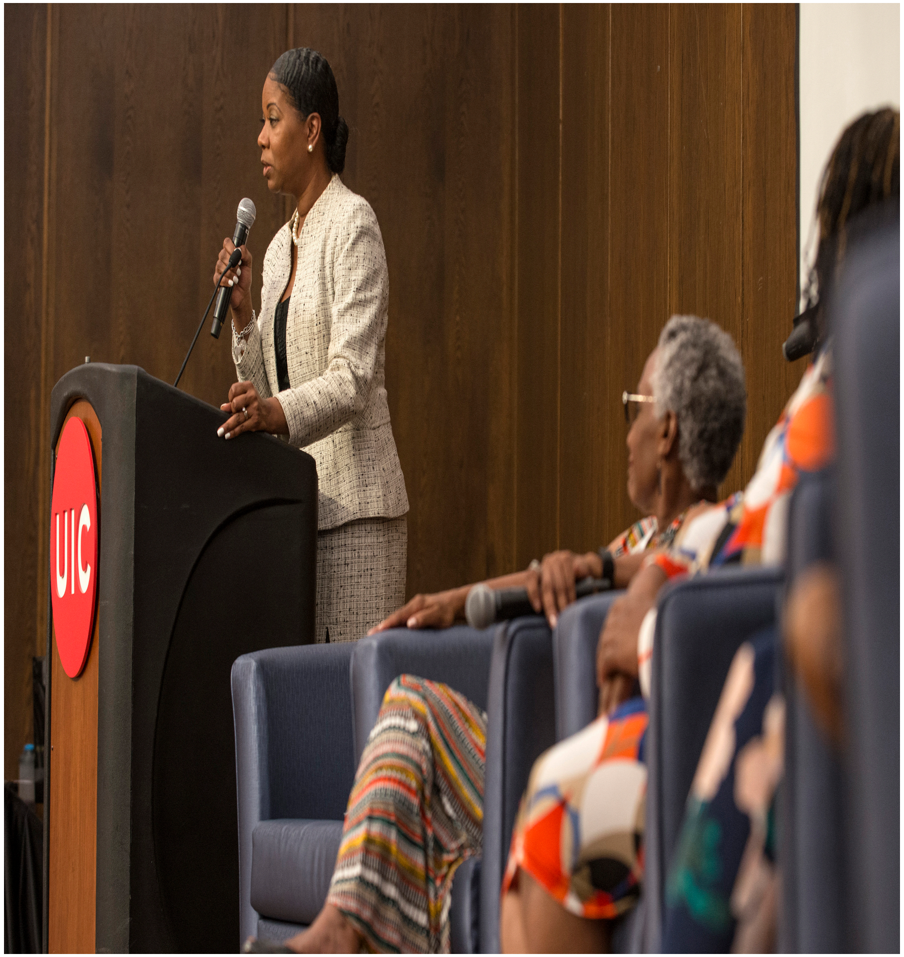

UI Cancer Center
Bench to Community

UICC Patient Brigade: At the Table and Determining the Menu

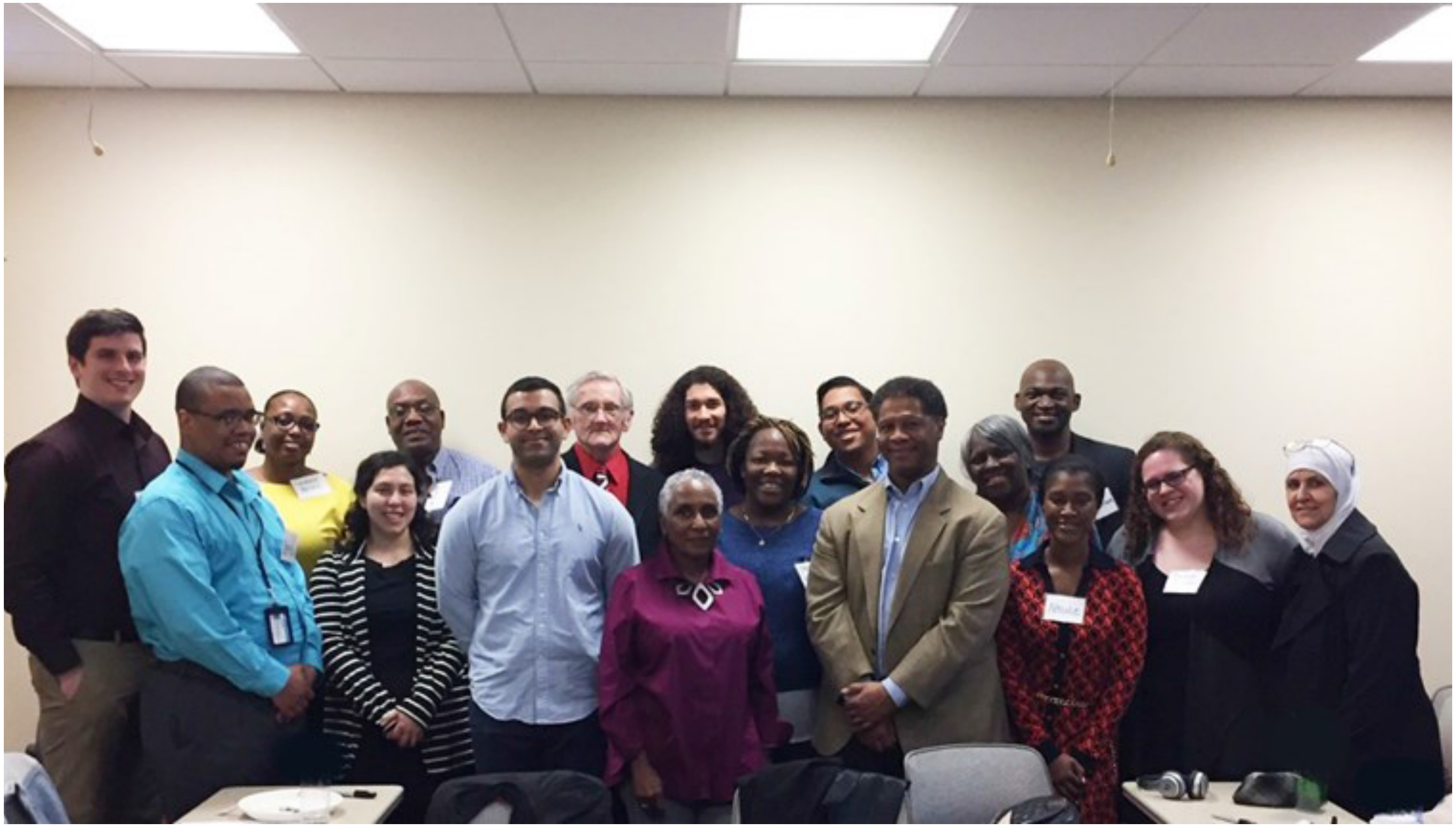

Thank you for your contributions as ambassadors and as reflections of UI Health Cancer Center's quality of care.

Henrietta Lacks Family Members

Shirley Lacks

Jeri Lacks-Whye

Veronica Robinson

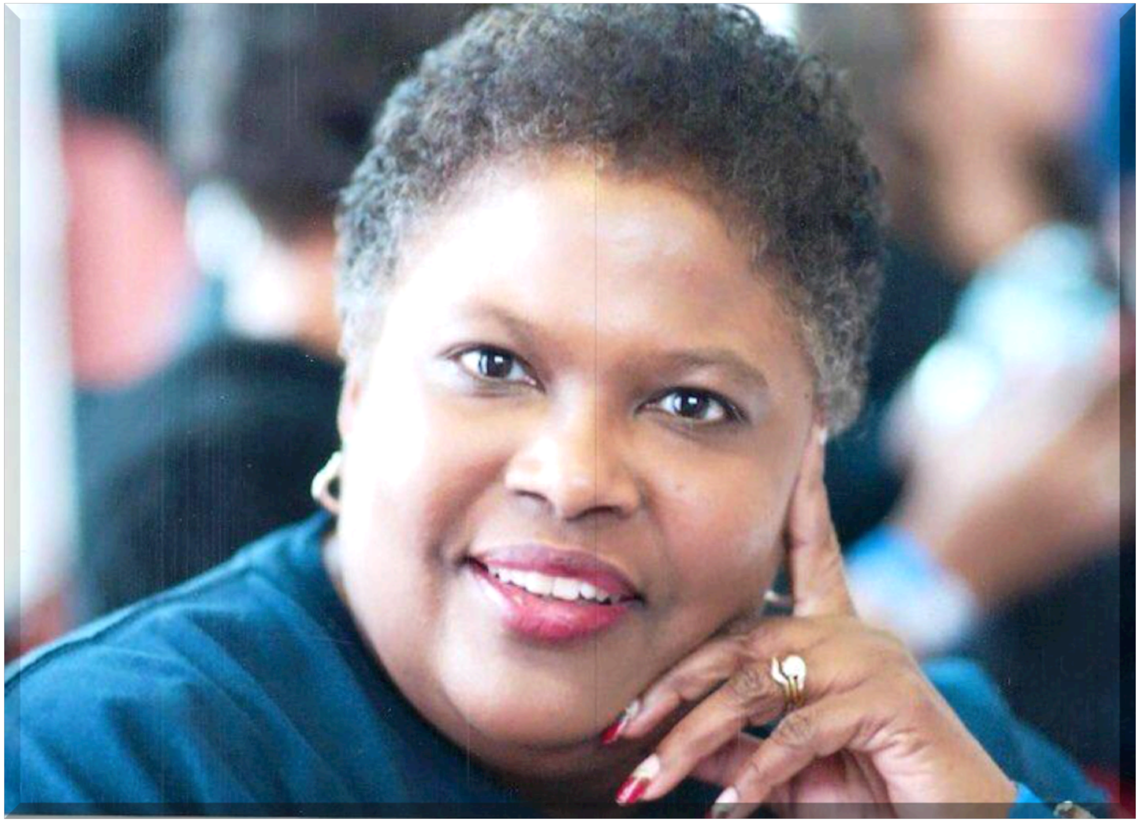

Honoring the Life and Legacy of Dr. Cynthia "Cee" Barnes-Boyd

Lisa Anderson-Shaw, DrPH, MA, MSN, ANP-BC
Clinical Assistant Professor of Medical-Surgical Nursing in Medical Education
College of Nursing, UIC
Director of the Clinical Ethics Consult Service, UI Health

Ashish Ansal, MD
Family Medicine, Mile Square Health Center, Englewood

Carol Gyimatey
UI Health Cancer Center Patient Brigade Member

RoseMarie Rogers
UI Health Cancer Center Patient Brigade Member

Big Data Research Ready Communities in Chicago

Regina Greer-Smith

healthcareresearch@sbcglobal.net
President, Healthcare Research Associates, LLC
Chicago, Illinois

Abstract

In this presentation, Ms. Greer-Smith presents the process through which her group, Pastors4PCOR, gathered community health data to serve as a resource for research. She provides an exemplar for how community members can learn "big data" and share that with researchers who can study and impact the health of that community. She noted that, in the past, researchers had approached the community for data but left the community nothing for their effort. In a testament to community-driven data collection, she said, "We got our own data, we did it our own way, and now we can share it with the world."

[*Ms. Greer-Smith*] How is everybody this morning? Can you see me in my loud dress? I am going to begin this presentation by thanking Dr. Consuelo Wilkins for finding me and inviting me to share our journey through "Big Data" here. [SLIDE 1]

I am going to begin this journey to share that my name is Regina Greer-Smith and I have a one-person organization called Healthcare Research Associates. I am the President, CEO, Chief Engagement Officer, and I call myself the "engagement activist," because I am the child of some very strong Civil Rights parents and family. I grew up in Chicago in Lawndale back in the early 50s. I sat at a table with some great African-American Catholics, which we call the "Three-Percenters" because there ain't that many black Catholics here in the country. I am working very much with my brothers and sisters in Chicago and around the country, but my real strong advocates and partners are faith-based communities, a group of African-American pastors that I have been working with for 20 years in the south side and south suburbs of Chicago called the Southland Ministerial Health Network. When I got called into service with PCORI back in 2012, the first group that I spoke to about PCORI was the Southland Ministerial Health Network. I found it kind of hard to believe that there was an organization funded by the Affordable Care Act that wanted patients to be the center of research and there was a strong focus on African-American communities and communities of color and underserved people. I told the pastors, "Maybe we need to look at this research thing because this is something that might help inform us and help us talk about why diseases are such a burden for us." I told the pastors that we had been doing health fairs and talking about the burdens in our communities, but now PCORI says we can design research and participate in research, so maybe we should look at this research and start getting involved with this. I said, "You could even be the 'Pastors4PCOR.'" They liked the name. We made a not-for-profit organization

about it. [SLIDE 2] I will tell you about our story from the South Side and south suburbs of Chicago on how we collectively developed a method to find out what our data was, learned about how to collect our own data and share with our communities, and determined how we could make data our own so we could find out the topics and questions we wanted to learn about so we could have a better health journey. We developed our program, "Pastors4PCOR." We made it a 501c3 with the help of Northwestern's Alliance for Research in Chicagoland Communities. They funded us with a grant, so we developed our not-for-profit and began the journey to find out who in our faith community wanted to be part of our program to develop. [SLIDE 3] We hadn't developed it yet. We were fortunate to be connected with a greater researcher that Melvin [*Melvin Thompson, Plenary Session III Moderator*] just mentioned, Dr. Rebecca Johnson at Northwestern. We actually sought her out. She didn't know us, but we were looking for our own researcher, somebody who could build a relationship. We achieved this by partnering with a researcher from an academic health center. They didn't come looking for us. We went looking for them. We wanted to foster relationships to build a robust research infrastructure in our communities and we wanted to build capacity for faith-based communities to engage in health research. [SLIDE 4] A couple of photos - the one on the far left of this and in the front is Dr. Rebecca Johnson and around the table are our pastor leaders from 20 churches in Chicago. Southland Ministerial Health Network made up the package for PCOR organizations. In the middle, me with a group of first ladies from Victory Christian Church. We did our first patient engagement cluster. We wanted to see if we could have conversations in the churches about healthcare. So, we did that. On the far left, there was a cold February of 2014 when the pastors decided they actually wanted to begin the research journey. So, we huddled around the table and they decided among themselves who was going to be the leader or the Chair of the program. The Chair turned out to be Bishop Simon Gordon, Senior Pastor of Triedstone Full Gospel Church and then Reverend Pastor Walter Turner of New Spiritual Light Baptist Church in South Shore. They are the Chair and Co-Chair. And you can see me far off on the left in that blue sweater. I was nervous waiting for them. They made their own decisions. The other pictures are when we were just sharing and talking about how we were going to really figure out how to do this research. And that picture of me in the red sweater, as you go through these pictures, you will see my hair change. I had lots of different hair-dos. I have grown hair and lost hair, but it turned out alright. [SLIDE 5] This is a couple of us, board members, of Pastors4PCOR. We are here in D.C. There is Bishop Simon Gordon on the left, Dr. Paris Davis, who was appointed the Executive Director of Pastors4PCOR, then me in the middle, and Pastor Turner on the left. We are in D.C. working with an organization and sharing Pastors4PCOR. That is our official logo and our button. [SLIDE 6] This slide will show you the organizations and board members of Pastors4PCOR. We are faith-based, community-based,

two academic medical centers, and a policy institute. We make up the Board for Pastors4PCOR and we do the guidance on how to develop our programming and just really making the decisions for the program. Dr. Rebecca Johnson is with the Osher Center of Northwestern and she is our lead investigator.

One of the things that we did to begin our journey was we had our first community day of research engagement where we invited community members to a church in the south suburbs, Victory Christian International, to talk about research and what research would actually look like in our communities. [SLIDE 7] We did some exercises. We did some facilitations. We did some table exercises. What is most important in the lower left-hand picture - we actually provided certification for Human Protections Training for all of the pastors in each of the 20 churches. That was facilitated by the Alliance for Research in Chicagoland Communities. All of the pastors got their IRBs. Very, very important. They still have those. That was the beginning of our research journey - get the pastors and the community to buy in and get our pastors certified. We did that with a community event. Now, our journey began in September 2015. [SLIDE 8] These are all of our Research Ministry Ambassadors. We gave them that title because our plan was to overlay research training in each of the health ministries in the 20 churches. So, we titled our ambassadors "Research Ministry Ambassadors," and that is something we kind of tagged and we kept that training. This is the beginning of our research training, our first picture, in September 2015. There I am in the middle with brown hair. I'm going to share now our roadmap. [SLIDE 9] We developed a five-module curriculum to train our Research Ministry Ambassadors how to get involved with research and specifically "big data." Today, I am going to talk about our journey through module 3, step 3, which is "Big Data." For step 2, all of our Research Ministry Ambassadors had a one-day training in Human Protections Training, so all of them also received their IRB training in addition to the pastors. That was important because they were going to do data collection and we all know in order to do data collection and research, we've got to be certified. If Jen Brown is here with the Alliance for Research in Chicagoland Communities, thank you very much. They came to our church, Triedstone, and they trained us that full day.

We are going to talk about step 3 today, big data. [SLIDE 10] We had three goals in our Big Data Training. Our first goal was we needed to know where the big data about health outcomes and relative factors comes from and feel competent engaging big data projects. We had to learn what big data was, where it came from, and how do we kind of transition into learning about our own data. Our second goal was we were going to have to develop a survey to identify the health conditions and factors which are priorities in our faith

communities. So, in order for us to get involved with the research, we had to determine what were the priorities in our communities and where did that data come from, especially in our faith communities. Then, goal three - we had to have the tools to conduct the survey on health conditions and factors impacting our faith-based communities. We were all on a journey to also develop our own surveys so we could determine how to collect our own data. [SLIDE 11] That is one of our training classes there - Triedstone Full Gospel Church in Beverley and south side of Chicago. This is Dr. [*Rebecca*] Johnson preaching to us about big data. What we did was we all got together and looked at some survey instruments and some reports and one of the ones we used was the American Community Survey. [SLIDE 12] We shared that with our Research Ministry Ambassadors so they could get the big picture of big data. Also, how and where is data collected and how are survey responses made? We used what was already out there and what people may already be familiar with to get them going on how to learn about big data. [SLIDE 13] What is big data used for? We showed them score cards, overall health outcomes, and health behavior. Then, we looked at the City of Chicago data. [SLIDE 14] We pulled down reports and shared with them how the city of Chicago collects data on a community-wide level and how it is measured to determine Chicago's Health Score Card. Then, we showed them specifically the zip codes that we work with. [SLIDE 15] We also did a survey on the health burdens and disparities in each of the zip codes in Chicago. Then, we also pulled out what we called the PCORI-style big data. We looked at some of the current PCORI projects going on in Chicago. We used a stroke program that is going on between Northwestern University and Rush and showed them how they are using data from Chicago to develop a PCORI project. Then, we also looked at PCORI's Patient-Powered Research Network. [SLIDE 16] We told them how patients around the country and in Chicago are actually joining these Patient-Powered Research Networks, PPRN's, that specifically invite patients to join who have that particular disease burden and learn and get information from them as well. So, we looked at the Healthy Heart Alliance and there is a large pragmatic study called "ADAPTABLE" that really manages that. We showed them that and we looked at the "ABOUT" network. In going through this journey, a couple of our research ministries at Baptist actually joined these big data projects and the PPRN [*Patient-Powered Research Network*].

Our second goal was to develop a survey we could share with the community. [SLIDE 17] We used a couple of tools to really outline the different disease burdens and chronic conditions that are happening around the body and then we made this little person and we did a couple of exercises on what diseases impact certain parts of the body so they could be familiar with where burdens lie in the human body. [SLIDE 18] We developed some survey templates so that people could understand when they share with their communities

who was doing the surveys, the health conditions that were important to them ... health factors of the demographics for each of their faith communities. [SLIDE 19] They did some of the survey instruments. Between our classroom inservice, which was every other month, we had exercises for them to do between those times. [SLIDE 20] There was three of us researchers and advocates. We served as mentors. We went to the churches and supported them as they were developing their surveys, learning to do the messaging, etc., so they could do it in their communities. It is one thing to do it in a classroom, but to really go to the churches and work with them is a different experience. So, we supported them with that. One of the things we had to do was help them develop some messaging. Even though we shared Pastors4PCOR, we had to deliver a message to the churches. First, we had to share who Pastors4PCOR was. You know - we are conducting the survey because ... (the message they wanted to deliver) ... who could take part in it (anyone who lives in the community and who prays in the suburbs and south side of Chicago can be part of the survey. You didn't have to be a church member. Anybody in the community or in that church's area could take the survey. What was very important was to let them know what was going to be happening with the data. That was extremely important. [SLIDE 21] Some of the demographic exercises in parts of the survey. Then, we did an exercise on the health burden for a particular area. [SLIDE 22] Many of our community people come from Harvey. I lived in the community right next to Harvey, so we demonstrated how Harvey fits into the full plan and scope of health data in Illinois. Health factors - we developed this so the Research Ministry Ambassadors could deliver it to their congregation. [SLIDE 23] Then, learn how to develop the survey. [SLIDE 24] After they developed the survey, then they went into their churches, and during Sunday service and Bible study and any other time community people were in the church, take a survey and deliver it. We collected about 638 surveys, which was pretty good the first time around. These were the survey instruments. [SLIDE 25] We also made sure our picture was on the survey so they could be familiar with us and the other Research Ministry Ambassadors for the program. They connected with us mentors. They delivered the surveys but I did the messaging. I had some great experiences in going to the pulpit delivering surveys. I had to first be vetted and approved by the Deacons to share the information and surveys while the Research Ministry Ambassador actually delivered the surveys and I did what was supposed to be a short sermon. I learned that in many of the churches when you hear the organ, you are supposed to stop. Me being a Catholic, when you hear the organ, that means you are supposed to sing. I got the two-minute warning a lot, and then I actually had a pastor who said, "Sister Greer-Smith, this church belongs to her, but we are glad she is here, but when you hear the music, you are supposed to slow down." I learned that lesson. Pastors are lovely people. They really helped me a lot and I learned a lot, too. [SLIDE 26] We

disseminated the surveys to our faith tradition and then we also had an evaluation for it. [SLIDE 27] These are the report cards that are the end result of our almost-year-long journeys for learning about big data, developing our surveys, and delivering them. We got our score cards and we used this information, not only to share amongst ourselves, but the Research Ministry Ambassadors got an individual score card to share with their pastors and their churches and their communities in addition to a Pastors4PCOR score card. They got a big one with everybody's information gathered and then there were individuals. These are some of the results of our year-long journey. One of the questions we asked our participants was - what are the resources your faith-based community needs to address the health conditions and factors you identified? [SLIDE 28] The most important things were education, programs, and research ... places and facilities that are needed in the communities (considered neighborhood factors), information and communications, access to affordable health, finances, and people. These were important factors that they said were priority for the community.

We had a graduation in October 2016. [SLIDE 29] There is me on the far right again with Bishop Gordon. He gave me a Certificate of Appreciation and I cried like a baby. I mean, it was very emotional, not to get just the certificate, but for the pastors to really embrace research ... and I will tell you, for researchers, PCORI is many times just a source of funding, but for our community, it was a lifeline and I will share that. This was the first opportunity we have had to be able to do research without fear and PCORI really brought us on that journey. We did this work under a Eugene Washington Engagement Award in 2015, and we were awarded another award in 2017 to disseminate our faith-based training. It is on PCORI's website, so we are sharing it freely. Pastor Barbara and I were taking selfies and stuff during the graduation ceremony. This is our graduation picture. [SLIDE 30] This is where we began in Triedstone Church and all of the Research Ministry Ambassadors who were certified during that process and delivered all the faith-based training. That is my journey. I hope it helps to really share how community people can learn how to do big data so that we can share with others so they can learn about us and really help us with research. What was important for us was that in this journey, the researchers weren't looking for us - we were looking for researchers. So, we really went out on faith and said, "We are going to try this and really activate what PCORI had promised with research done differently." It was really meaningful for us because we had researchers in the past come to the community and they left nothing. We got our own data, we did it our own way, and now we can share it with the world. Thank you for listening.

<u>Declarations</u>

- The presenter has no financial competing interests related to this presentation.
- All authors have approved the manuscript for submission
- The content of this presentation and transcript has not been published or submitted for publication elsewhere.
- This presentation was organized by the Meharry-Vanderbilt Alliance with support from NCATS and the NIMHD of the National Institutes of Health under award number R13TR001694

Pastors4PCOR

"To inform, inspire and engage congregations in research through collaboration".

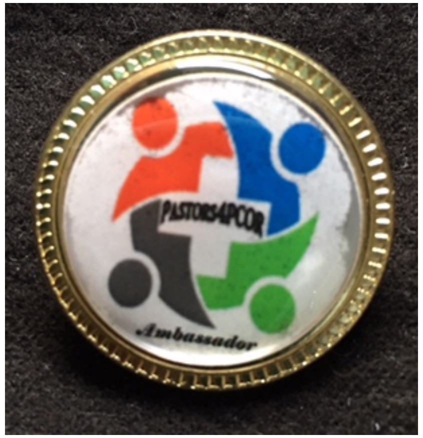

BIG DATA
Research-Ready Communities in Chicago
Advancing The Science of Community Engaged Research
September 14-15, 2017

Regina Greer-Smith MPH LFACHE

Healthcare Research Associates, LLC

President/Chief Engagement Officer/Engagement Activists

Pastors4PCOR

"To inform, inspire and engage congregations in research through collaboration".

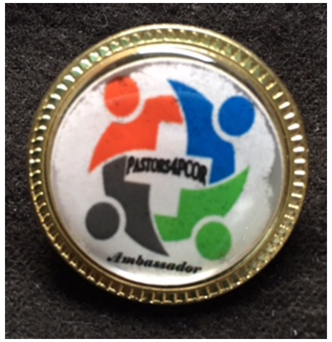

BIG DATA
Research-Ready Communities in Chicago

- **Our story from the south side and south suburbs of Chicago.**
- **How to we collectively developed our unique method for faith-based communities to engage, train and develop surveys to learn about our data.**
- **How we developed a method to share our data with our faith based communities and more.**
- **How We Will Use Our Data to Make our Communities Healthy thru Research.**

Pastors4PCOR

"To inform, inspire and engage congregations in research through collaboration".

BIG DATA

Research-Ready Communities in Chicago

Our Mission

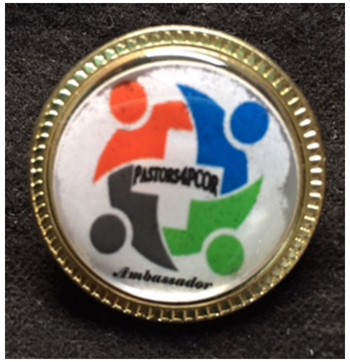

"To increase the participation of underserved communities of color in comparative effectiveness research and patient centered outcomes research through the design, development and implementation, reporting and dissemination of research"

We will achieve this by:

- Partnering with researchers from academic health research centers
- Fostering relationships to build a robust research infrastructure in the communities of the congregations
- Building the capacity of faith based communities to engage in health research

Pastors4PCOR

"To inform, inspire and engage congregations in research through collaboration".

BIG DATA
Research-Ready Communities in Chicago

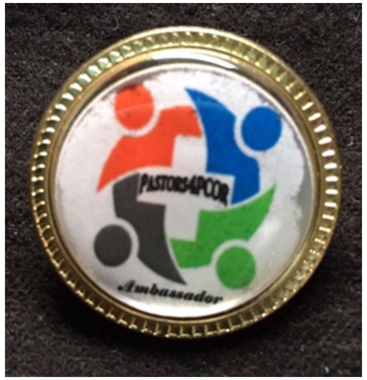

BUILDING TRUST

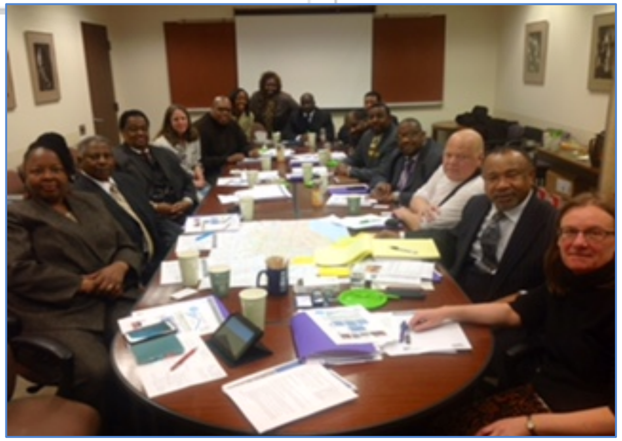

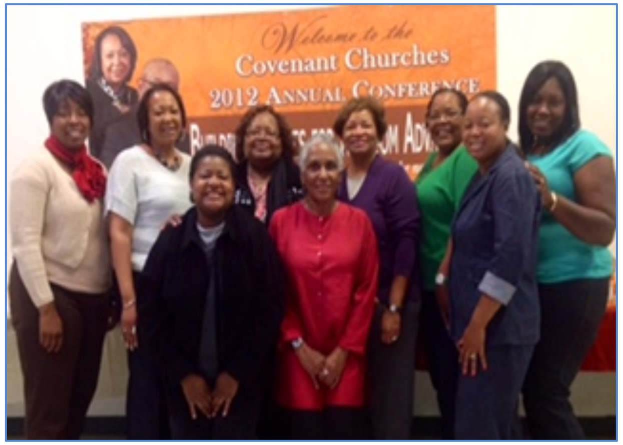

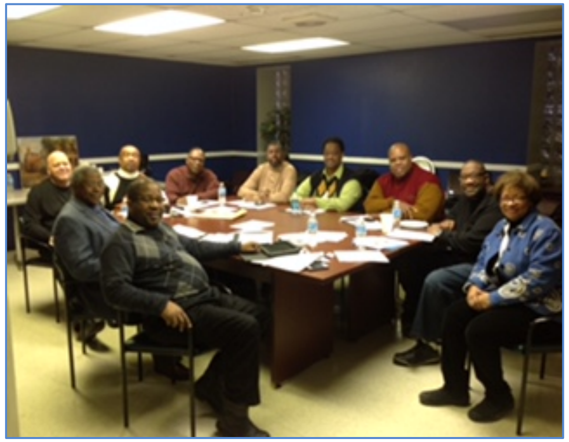

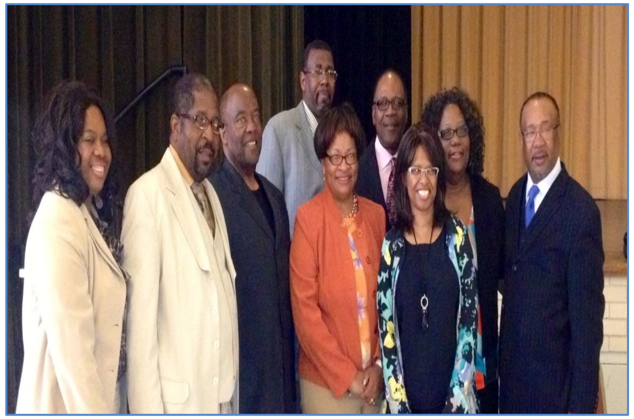

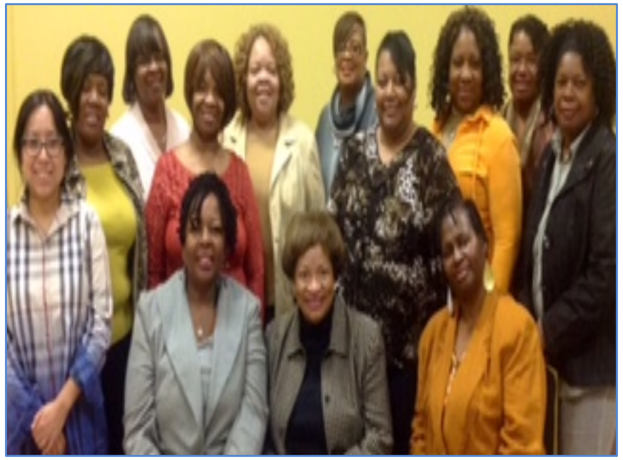

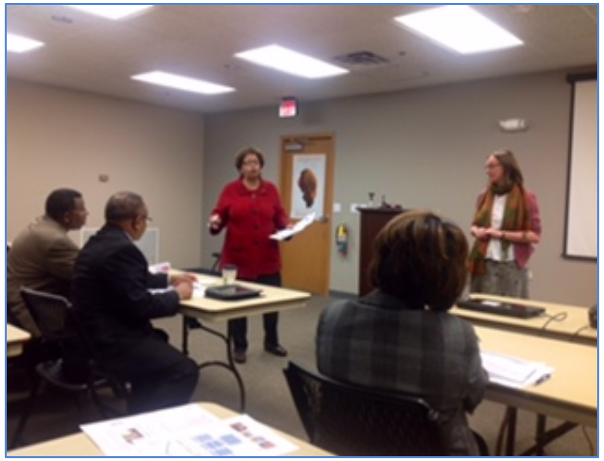

BIG DATA
Research-Ready Communities in Chicago

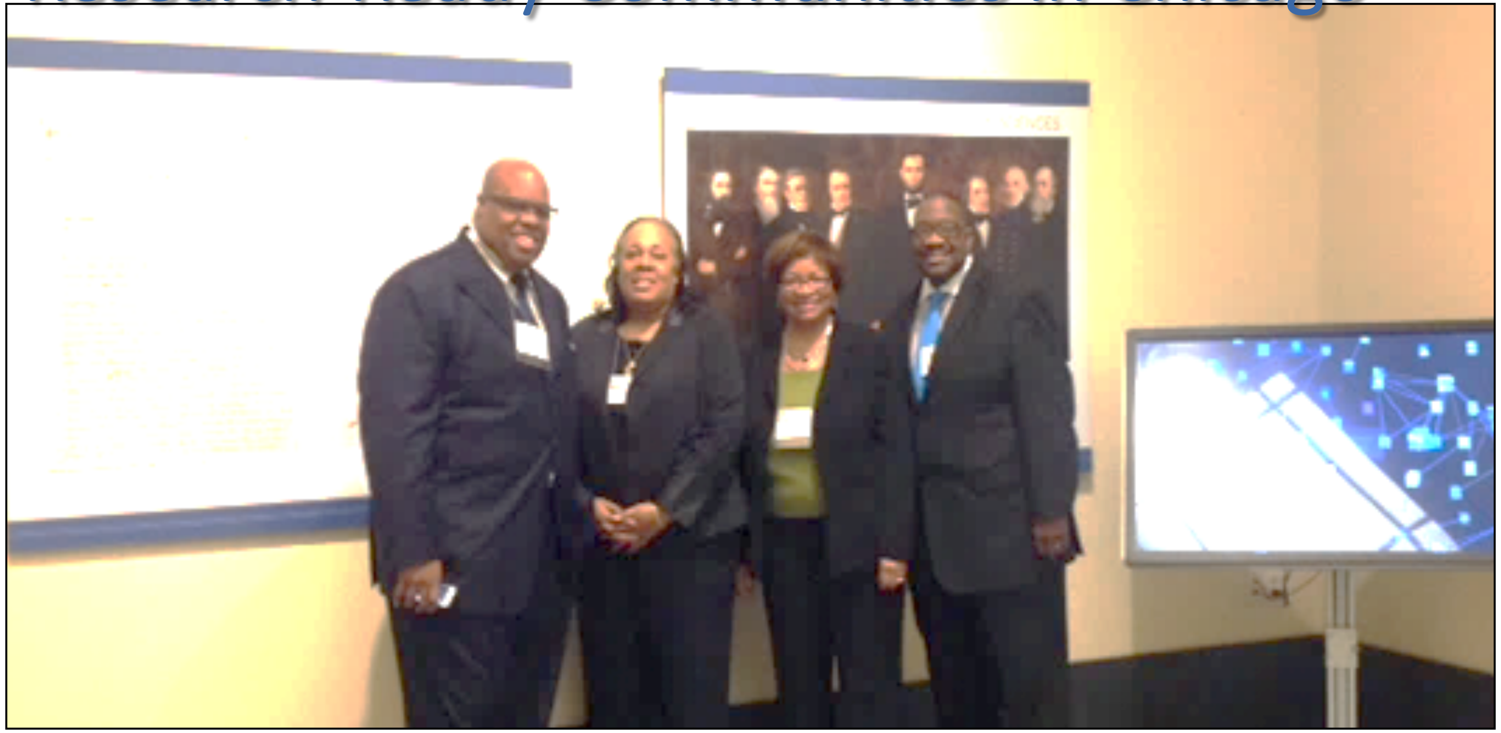

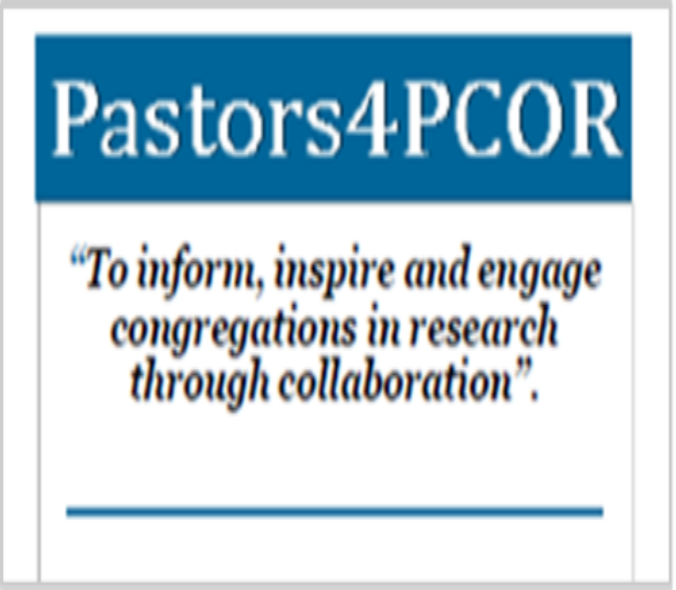

PASTORS4PCOR BOARD LEADERSHIP

- Bishop Simon Gordon – Board Chair – Senior Pastor/Triedstone Full Gospel Church – Chicago
- Dr. Paris Davis, MBA – ED – Pastors4PCOR – Elder-Triedstone Full Gospel Church/Total Resources Community Development Corporation (TRCDO)
- Regina Greer –Smith – Healthcare Research Associates, LLC/Co-founder of Pastors4PCOR/The S.T.A.R. Initiative
- Reverend Walter Turner III –Co-Chair-Pastors4PCOR - Senior Pastor/New Spiritual Light Baptist Church - Chicago

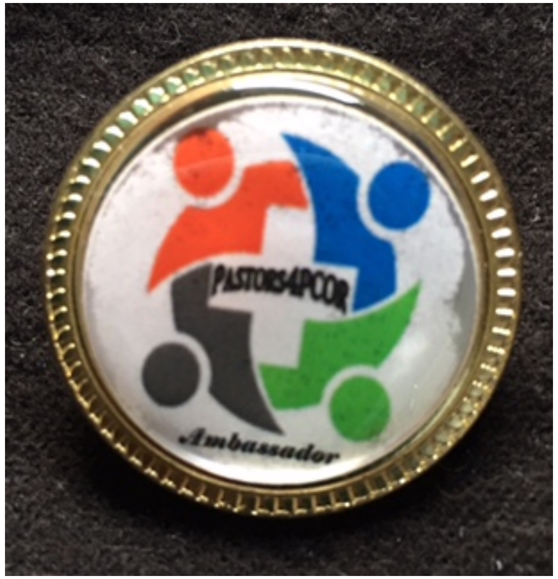

Pastors4PCOR

"To inform, inspire and engage congregations in research through collaboration".

BIG DATA
Research-Ready Communities in Chicago

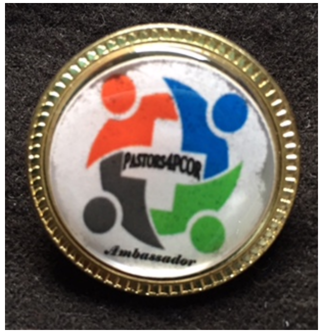

RUSH UNIVERSITY MEDICAL CENTER

South Chicago faith-based community network and research partners

Various congregations from Metro-Chicago participated

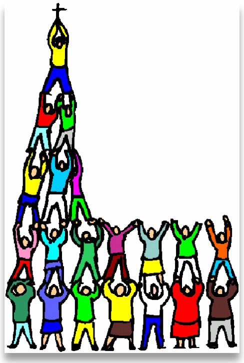

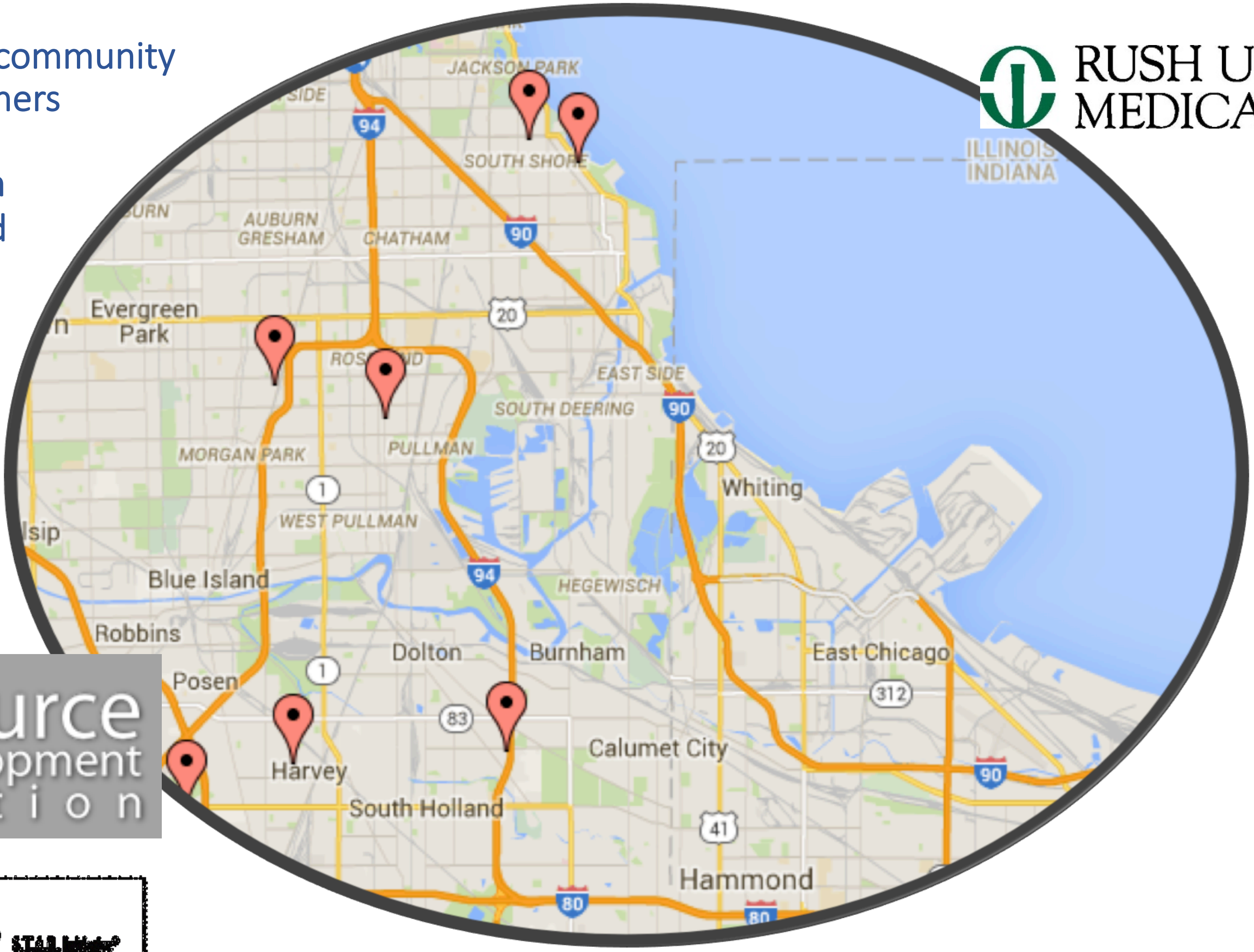

Buehler Center on Aging, Health & Society

COOK COUNTY HEALTH & HOSPITALS SYSTEM CCHHS

Total Resource Community Development Organization

Pastors4PCOR

"To inform, inspire and engage congregations in research through collaboration".

BIG DATA
Research-Ready Communities in Chicago

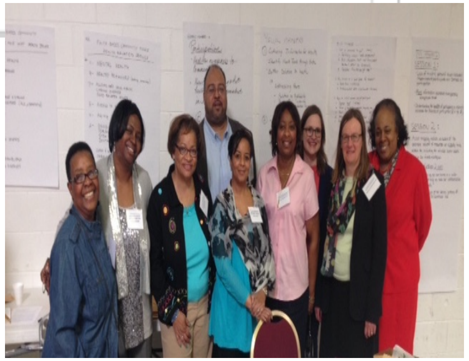

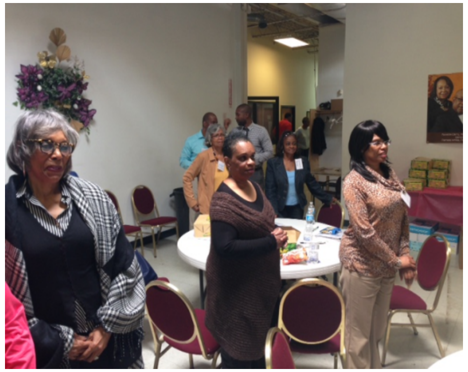

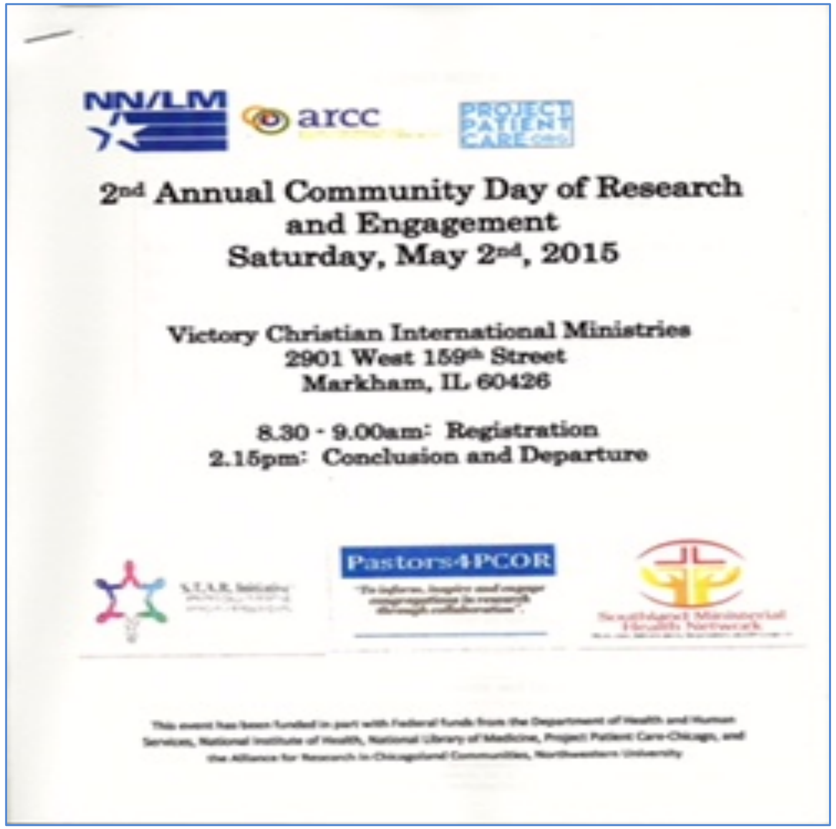
NN/LM
arcc
PROJECT PATIENT CARE

2nd Annual Community Day of Research and Engagement
Saturday, May 2nd, 2015

Victory Christian International Ministries
2901 West 159th Street
Markham, IL 60426

8.30 - 9.00am: Registration
2.15pm: Conclusion and Departure

Pastors4PCOR

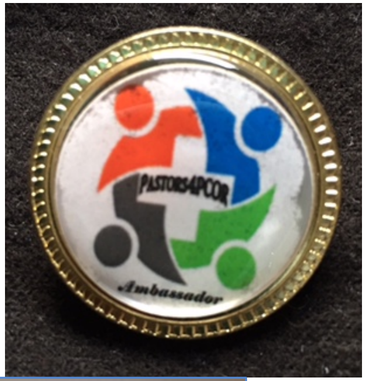

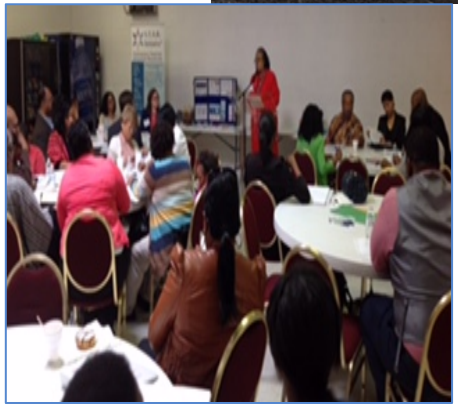

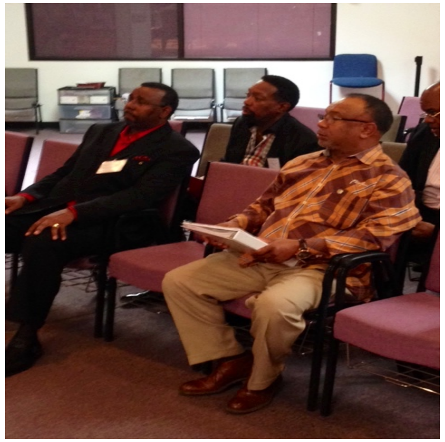

- Community engagement/orientation to research (i.e. what would research look like in a faith community
- Pastors Certified in Human Subjects Protections
- Learning if Faith Community had an "appetite" for research

Pastors4PCOR

"To inform, inspire and engage congregations in research through collaboration".

BIG DATA
Research-Ready Communities in Chicago

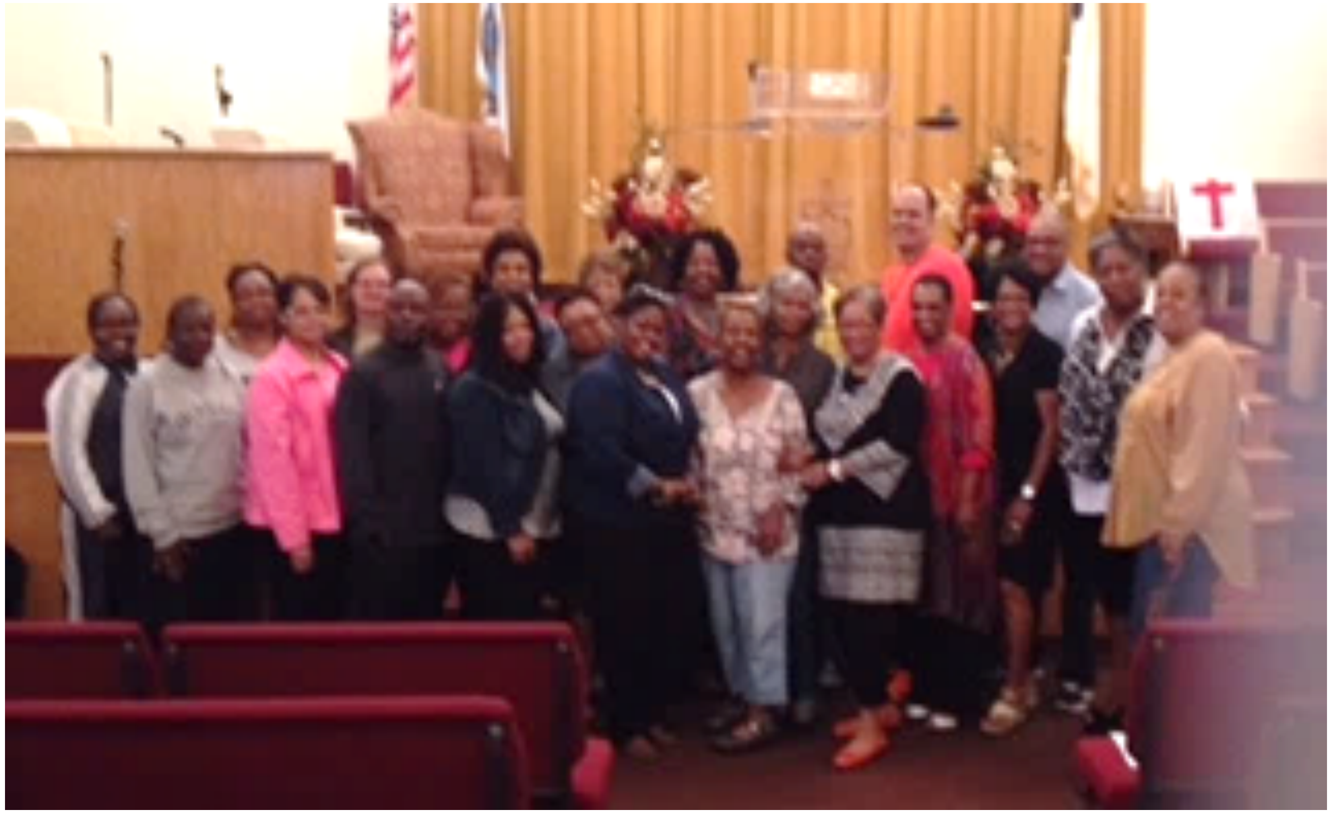

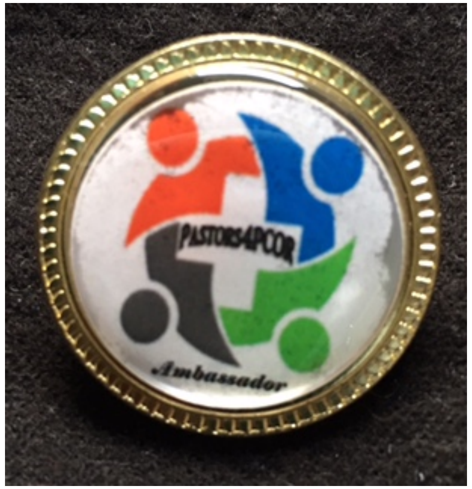

Pastors4PCOR Research Ministry Ambassadors and Team
September 2015
The Journey to Get Our Research Ministry Ambassadors "Research Ready"

Pastors4PCOR

"To inform, inspire and engage congregations in research through collaboration".

BIG DATA

Research-Ready Communities in Chicago

Faith based community research engagement roadmap

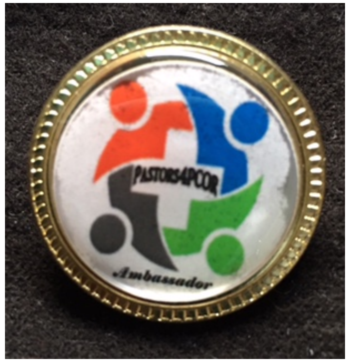

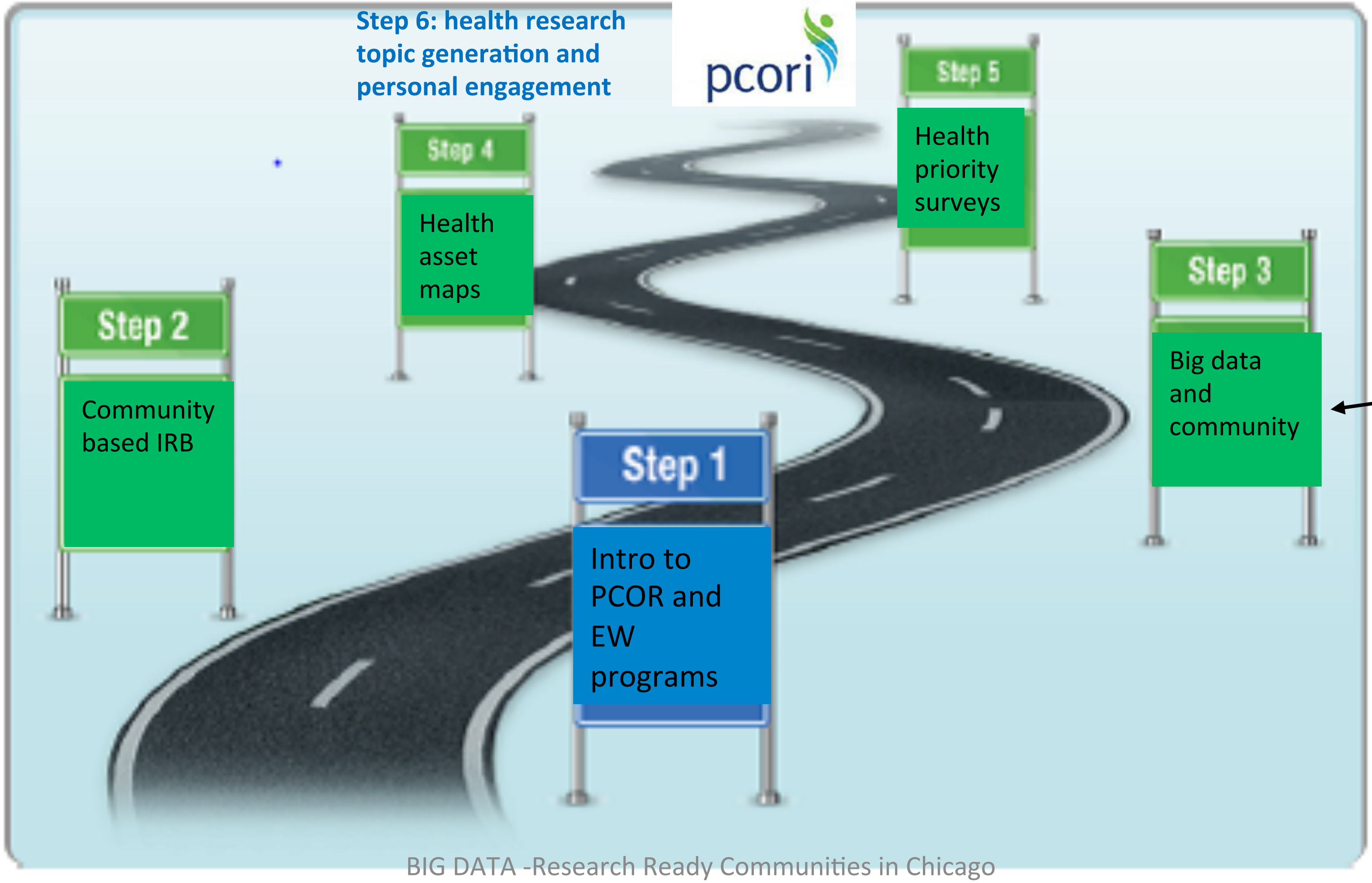

TODAY'S FOCUS
BIG DATA & COMMUNITY

Pastors4PCOR

"To inform, inspire and engage congregations in research through collaboration".

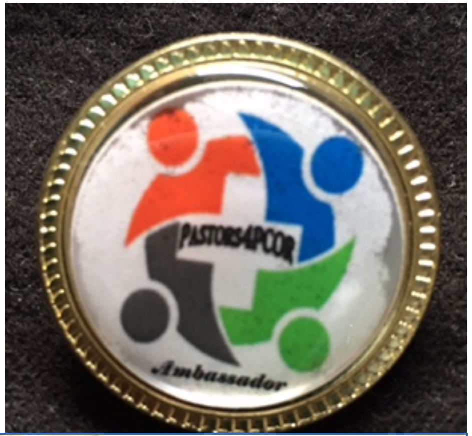

BIG DATA
Research-Ready Communities in Chicago

WE OWN OUR DATA

- ✓Goal A: Know where the big data about health outcomes and related factors comes from and feel confident in engaging with big data projects

- ✓Goal B: Have developed a survey to identify the health conditions and factors which are priorities in your faith based community

- ✓Goal C: Have the tools to conduct a survey on health conditions and factors impacting your faith based community

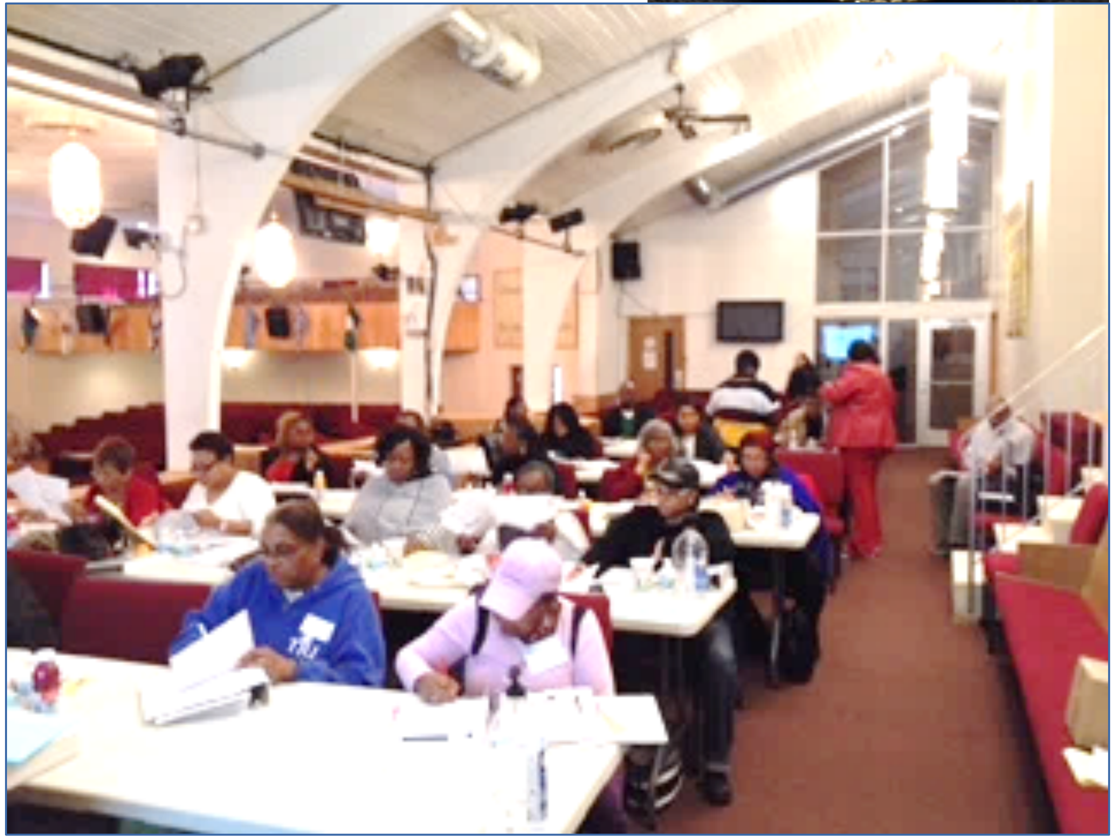

Pastors4PCOR

"To inform, inspire and engage congregations in research through collaboration".

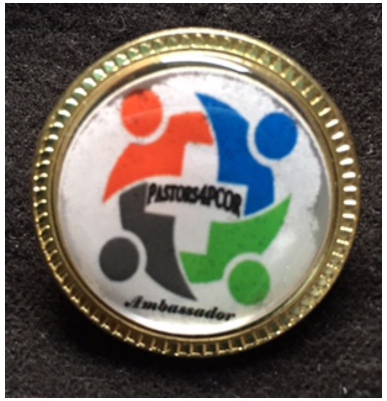

BIG DATA

Research-Ready Communities in Chicago

GOAL A:

Know where the big data about health outcomes and related factors comes from and feel confident in engaging with big data projects

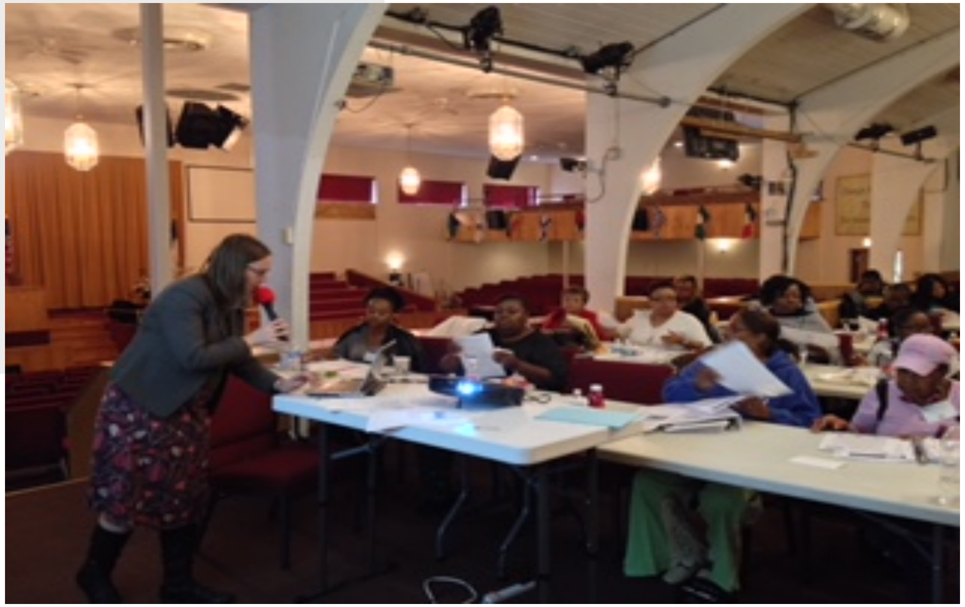

Pastors4PCOR

"To inform, inspire and engage congregations in research through collaboration".

BIG DATA

Research-Ready Communities in Chicago

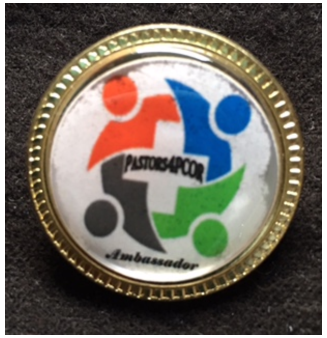

What is big data?

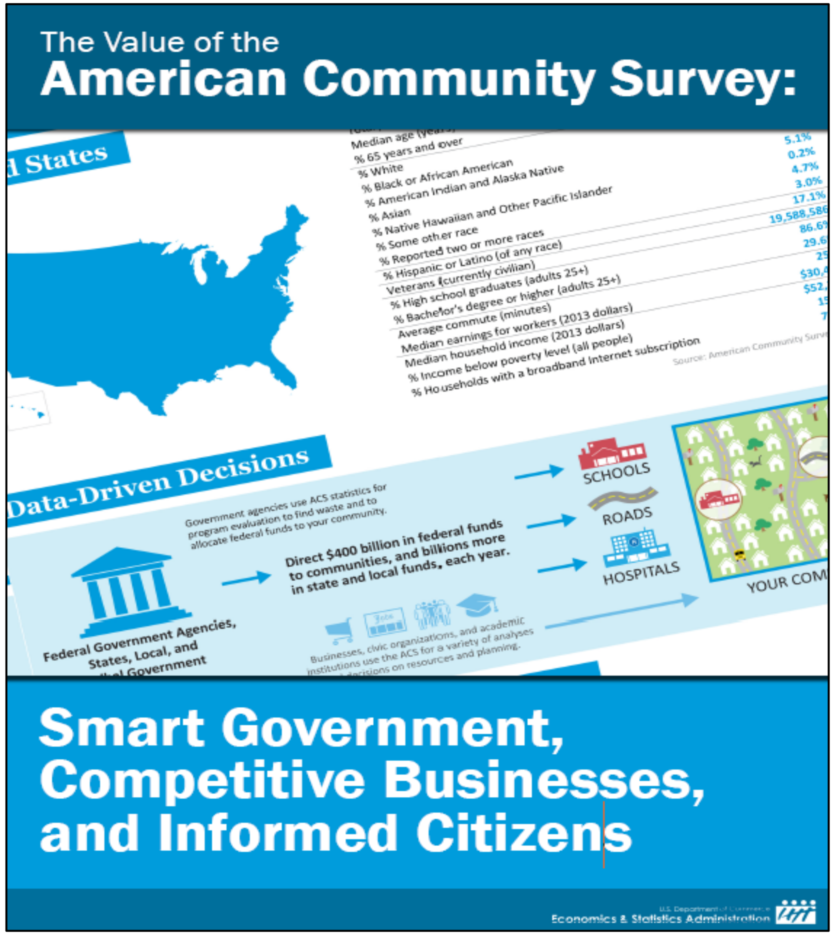

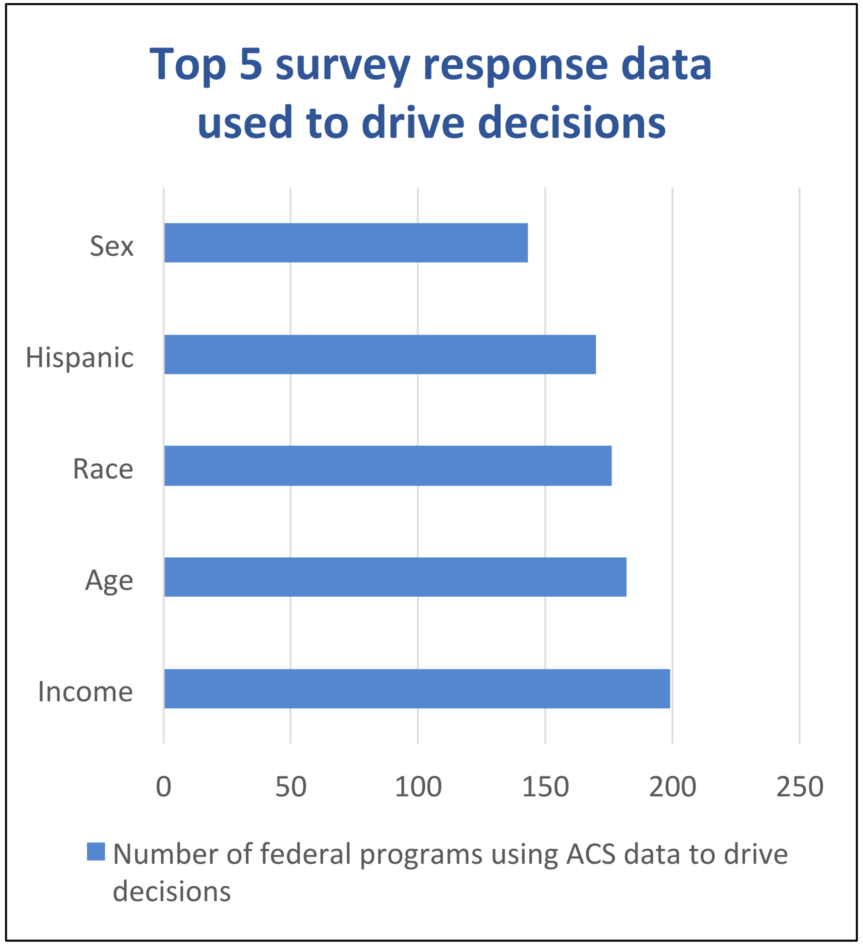

Pastors4PCOR

"To inform, inspire and engage congregations in research through collaboration".

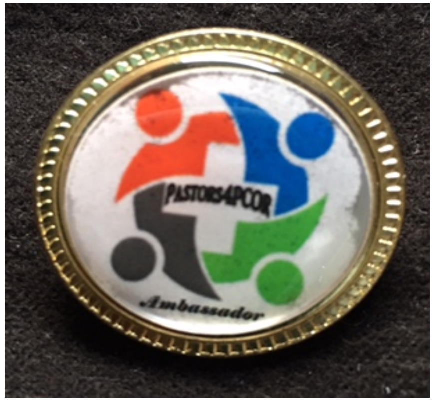

BIG DATA

Research-Ready Communities in Chicago

What is big data used for?

Health System score cards

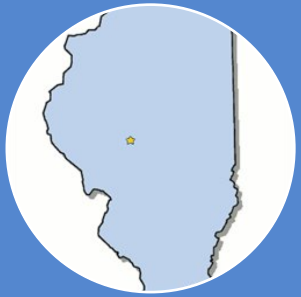

Overall Health Outcomes

Health Behaviors

Addressing Health Disparities

Pastors4PCOR

"To inform, inspire and engage congregations in research through collaboration".

BIG DATA

Research-Ready Communities in Chicago

What is happening at County and City level?

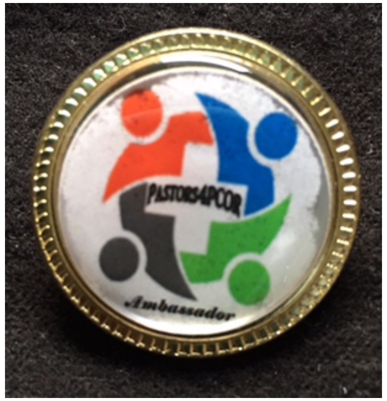

Health Impact Collaborative of Cook County

Working together for healthy communities.

Take the survey online:

https://www.surveymonkey.com/r/HealthImpactCook2015

Twenty-two (22) hospitals in Cook County, in partnership with health departments and many community organizations, are looking at health and wellness in neighborhoods across Cook County. Please take a few minutes to complete this survey. You will not be asked your name. The responses are confidential and will only be used to understand what the important health issues are in your community. If you have any questions about the survey, please contact: Kristin Monnard at Kristin.Monnard@iphionline.org or 312-850-4744.

1. Do you live in Chicago or suburban Cook County?

☐ Yes ☐ No ** if you do not live in Chicago or Cook County, you are not eligible to complete this survey **

2. Please enter the Zip Code in which you live: ________________

Part 1: Community Health

For this section of the survey, we are asking about the community where you live. Think about community in terms of your neighborhood, Chicago community area, or suburban city or village.

1. In general, how would you rate your community on each item below?

A. As a healthy place to live	Excellent	Good	Fair	Poor	Very Poor
B. As a place to raise children	Excellent	Good	Fair	Poor	Very Poor
C. As a place to work	Excellent	Good	Fair	Poor	Very Poor
D. As a place to grow old	Excellent	Good	Fair	Poor	Very Poor

2. How would you rate your community on each item below?

A. How adequate is adult education and job training in your community?	Extremely	Very	Somewhat	Not Very	Not at All	Don't Know
B. How available is good childcare in your community?	Extremely	Very	Somewhat	Not Very	Not at All	Don't Know
C. How good are the schools (Kindergarten through 12th grade) in your community?	Extremely	Very	Somewhat	Not Very	Not at All	Don't Know
D. How affordable is the housing in your community?	Extremely	Very	Somewhat	Not Very	Not at All	Don't Know
E. How available are healthy foods, including fresh fruits and vegetables, in your community?	Extremely	Very	Somewhat	Not Very	Not at All	Don't Know

1

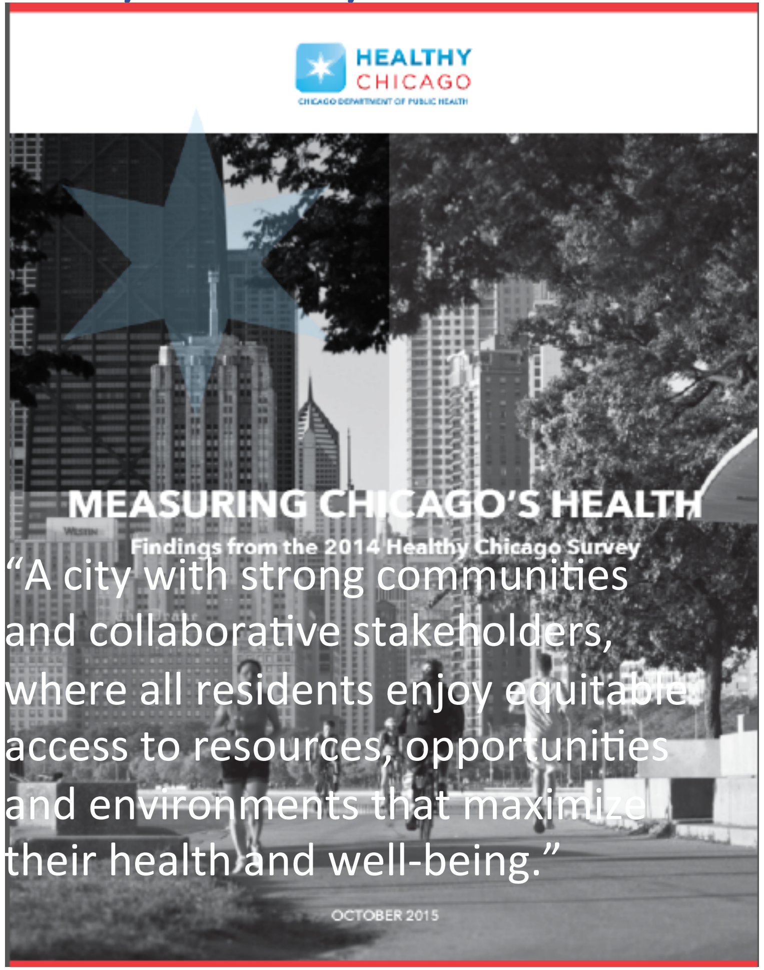

"A city with strong communities and collaborative stakeholders, where all residents enjoy equitable access to resources, opportunities and environments that maximize their health and well-being."

Pastors4PCOR

"To inform, inspire and engage congregations in research through collaboration".

BIG DATA

Research-Ready Communities in Chicago

Big Data PCORI style

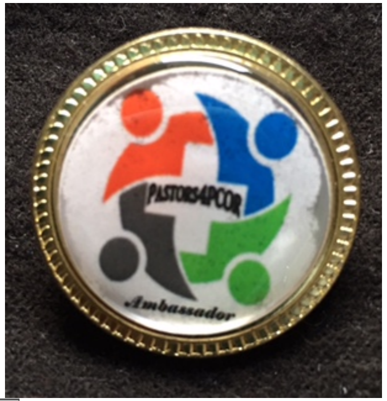

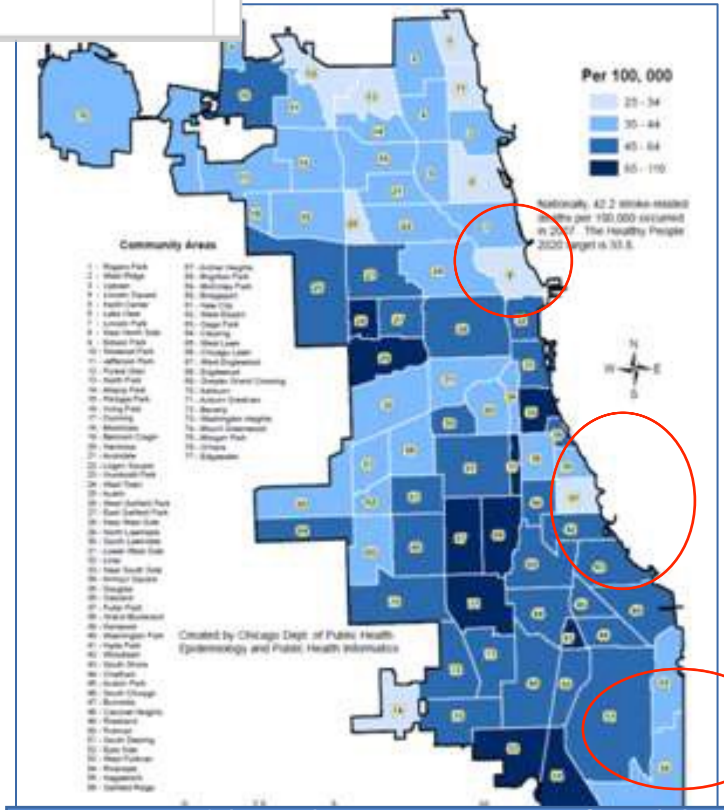

Created by Chicago Dept of Public Health

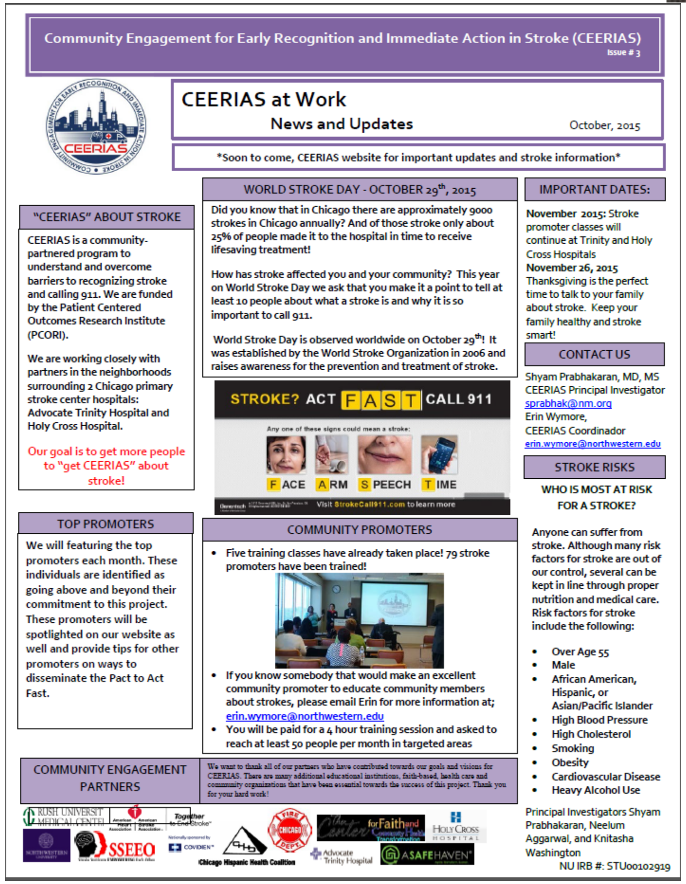

Community Engagement for Early Recognition and Immediate Action in Stroke (CEERIAS)
Issue # 3

CEERIAS at Work
News and Updates
October, 2015

Soon to come, CEERIAS website for important updates and stroke information

"CEERIAS" ABOUT STROKE

CEERIAS is a community-partnered program to understand and overcome barriers to recognizing stroke and calling 911. We are funded by the Patient Centered Outcomes Research Institute (PCORI).

We are working closely with partners in the neighborhoods surrounding 2 Chicago primary stroke center hospitals: Advocate Trinity Hospital and Holy Cross Hospital.

Our goal is to get more people to "get CEERIAS" about stroke!

WORLD STROKE DAY - OCTOBER 29th, 2015

Did you know that in Chicago there are approximately 9000 strokes in Chicago annually? And of those stroke only about 25% of people made it to the hospital in time to receive lifesaving treatment!

How has stroke affected you and your community? This year on World Stroke Day we ask that you make it a point to tell at least 10 people about what a stroke is and why it is so important to call 911.

World Stroke Day is observed worldwide on October 29th! It was established by the World Stroke Organization in 2006 and raises awareness for the prevention and treatment of stroke.

STROKE? ACT F A S T CALL 911
Any one of these signs could mean a stroke:
FACE ARM SPEECH TIME
Visit StrokeCall911.com to learn more

IMPORTANT DATES:

November 2015: Stroke promoter classes will continue at Trinity and Holy Cross Hospitals
November 26, 2015
Thanksgiving is the perfect time to talk to your family about stroke. Keep your family healthy and stroke smart!

CONTACT US

Shyam Prabhakaran, MD, MS
CEERIAS Principal Investigator
sprabhak@nm.org
Erin Wymore,
CEERIAS Coordinador
erin.wymore@northwestern.edu

STROKE RISKS

WHO IS MOST AT RISK FOR A STROKE?

Anyone can suffer from stroke. Although many risk factors for stroke are out of our control, several can be kept in line through proper nutrition and medical care. Risk factors for stroke include the following:

- Over Age 55
- Male
- African American, Hispanic, or Asian/Pacific Islander
- High Blood Pressure
- High Cholesterol
- Smoking
- Obesity
- Cardiovascular Disease
- Heavy Alcohol Use

TOP PROMOTERS

We will featuring the top promoters each month. These individuals are identified as going above and beyond their commitment to this project. These promoters will be spotlighted on our website as well and provide tips for other promoters on ways to disseminate the Pact to Act Fast.

COMMUNITY PROMOTERS

- Five training classes have already taken place! 79 stroke promoters have been trained!
- If you know somebody that would make an excellent community promoter to educate community members about strokes, please email Erin for more information at; erin.wymore@northwestern.edu
- You will be paid for a 4 hour training session and asked to reach at least 50 people per month in targeted areas

COMMUNITY ENGAGEMENT PARTNERS

We want to thank all of our partners who have contributed towards our goals and visions for CEERIAS. There are many additional educational institutions, faith-based, health care and community organizations that have been essential towards the success of this project. Thank you for your hard work!

Rush University Medical Center · American Heart Association · American Stroke Association · Together to End Stroke · Northwestern University · SSEEO · Covidien · Chicago Hispanic Health Coalition · Chicago Fire Dept. · The Center for Faith and Community Health Transformation · Holy Cross Hospital · Advocate Trinity Hospital · A Safe Haven

Principal Investigators Shyam Prabhakaran, Neelum Aggarwal, and Knitasha Washington
NU IRB #: STU00102919

Pastors4PCOR

"To inform, inspire and engage congregations in research through collaboration".

BIG DATA

Research-Ready Communities in Chicago

Joining a Big Data *Patient-Powered Research Network*

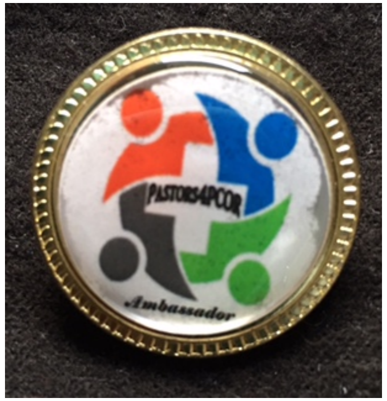

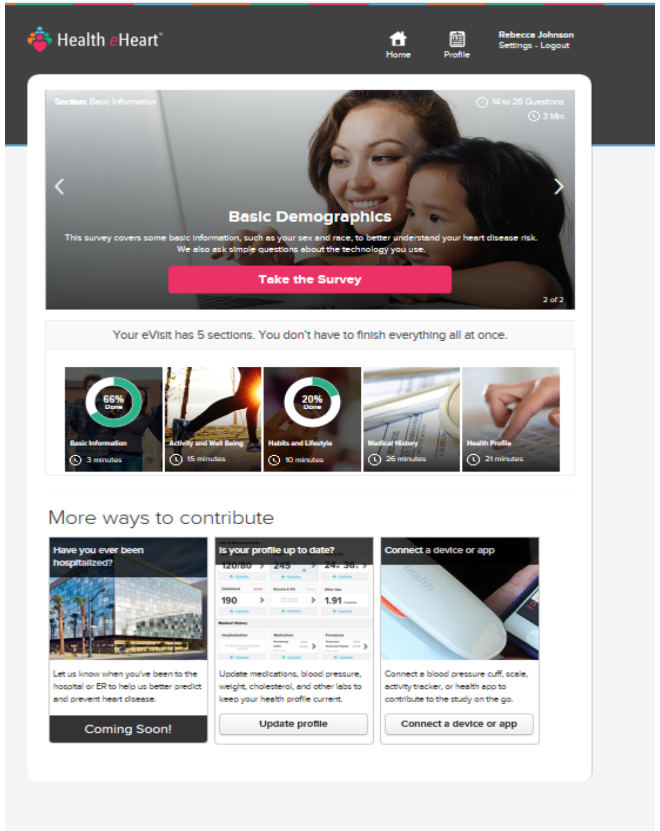

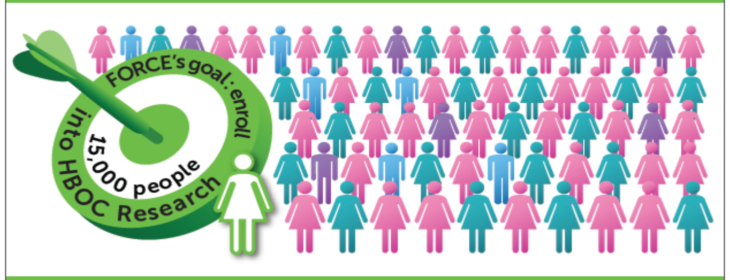

Help FORCE Accelerate Hereditary Cancer Research

www.ABOUTnetwork.org

Why is this important?
Research is the only way to improve prevention, detection, and treatment for cancer. Hereditary cancer research studies are in urgent need of participants.

How will we succeed?
FORCE is creating a research registry to improve patients' outcomes and match people to clinical trials.

What is the ABOUT Network?
ABOUT is the only network focused on hereditary cancer in the National Patient-Centered Research Network and the only research registry built by and for the HBOC community.

How can I join the ABOUT Network?
Visit: www.aboutnetwork.org for enrollment information.

FORCE Facing Our Risk of Cancer Empowered — ABOUT Research Registry

For more information contact us at:
(866) 632-2873 or
aboutnetwork@facingourrisk.org

Pastors4PCOR

"To inform, inspire and engage congregations in research through collaboration".

BIG DATA

Research-Ready Communities in Chicago

GOAL B:

"Have developed a survey to identify the health conditions and factors which are priorities in your faith based communities"

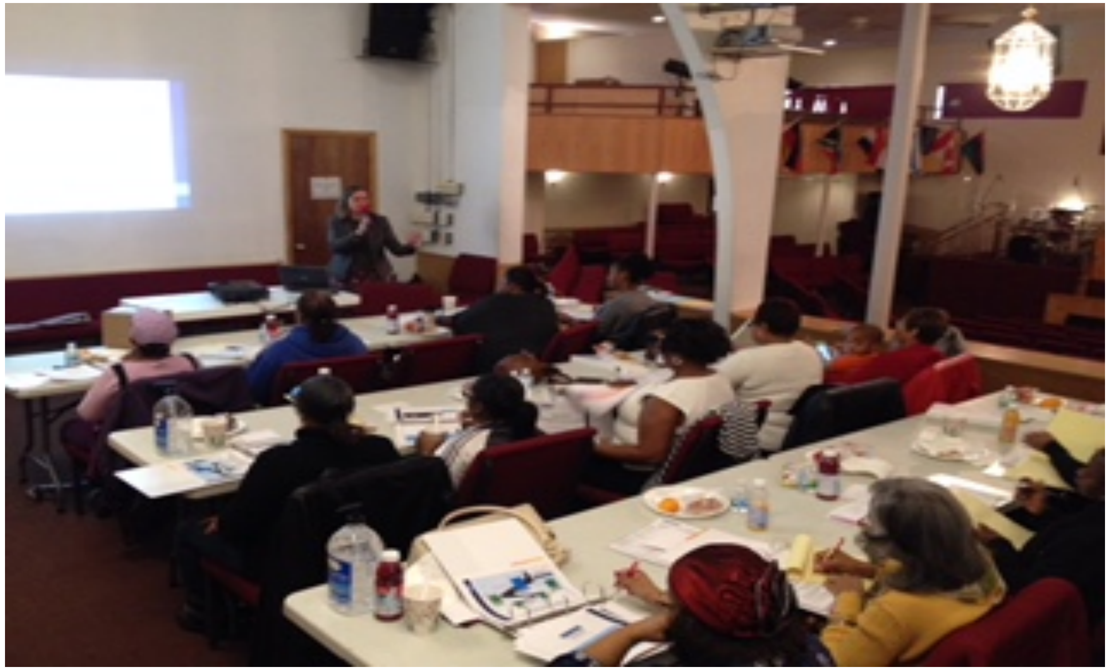

Pastors4PCOR

"To inform, inspire and engage congregations in research through collaboration".

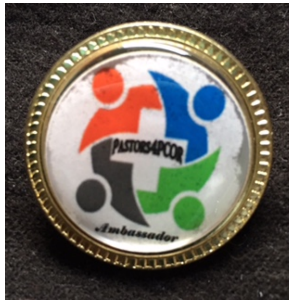

BIG DATA

Research-Ready Communities in Chicago

Building a faith-based community health survey

- **Health outcomes:**
- **Length of life**
- **Quality of life**

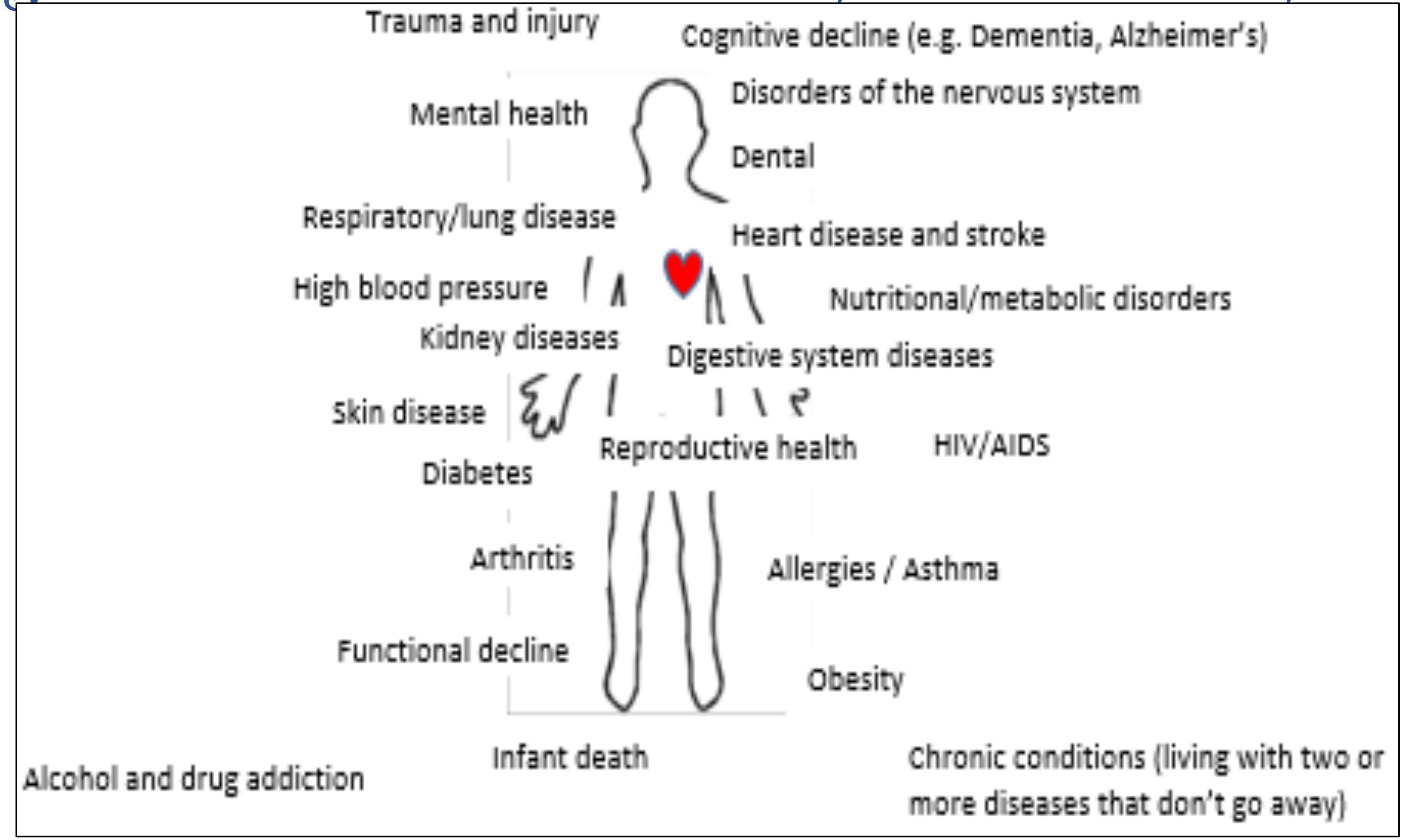

Pastors4PCOR

"To inform, inspire and engage congregations in research through collaboration".

BIG DATA

Research-Ready Communities in Chicago

Survey template

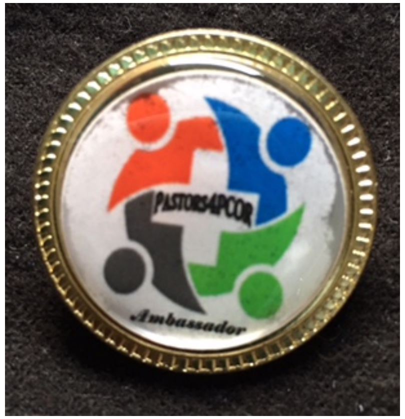

- Who is doing the survey?
- Health conditions
- Health factors
- Demographics

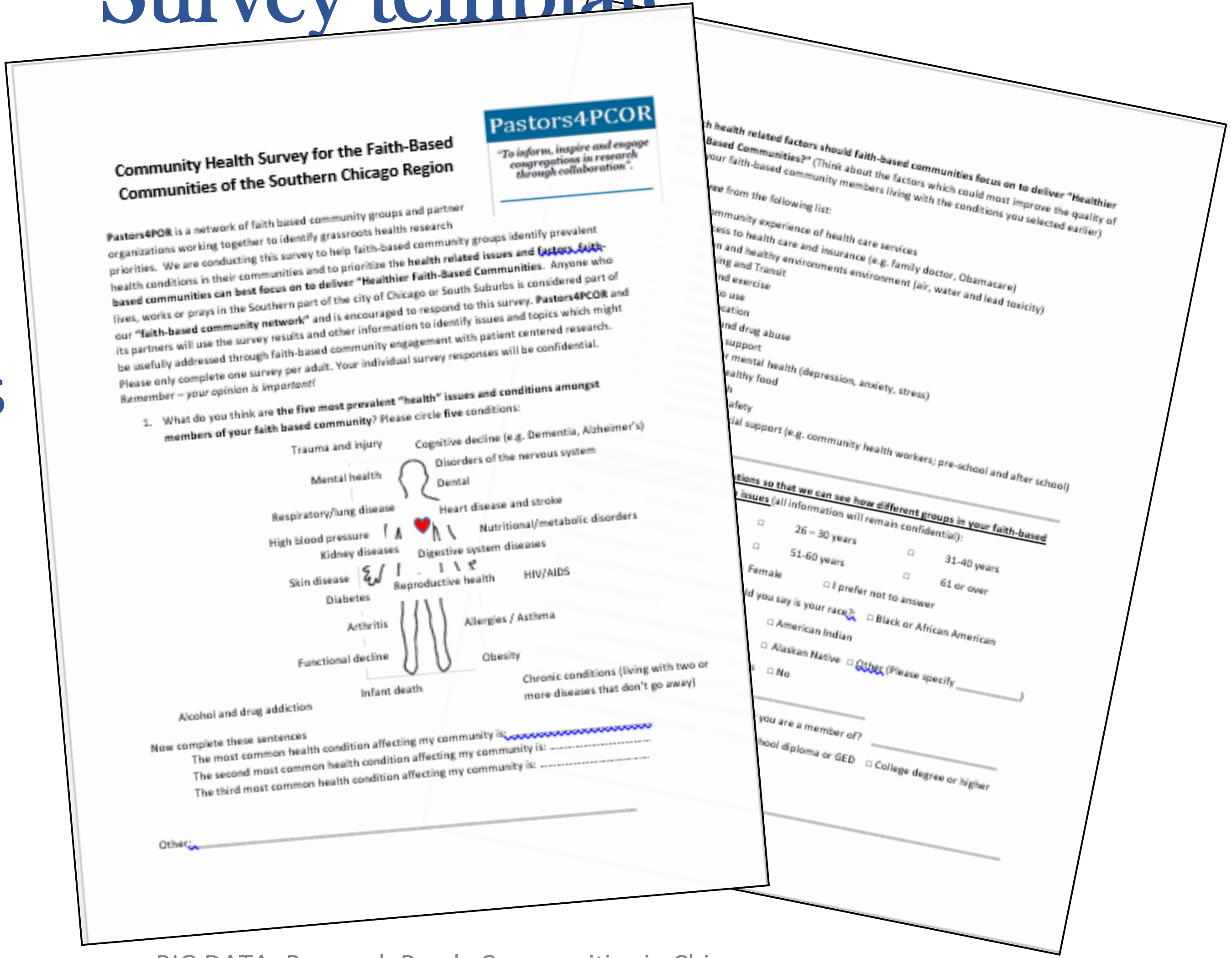

Pastors4PCOR

"To inform, inspire and engage congregations in research through collaboration".

BIG DATA

Research-Ready Communities in Chicago

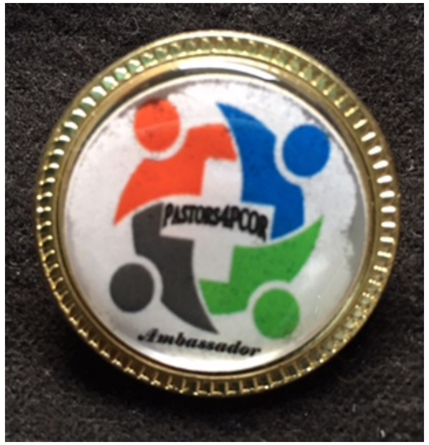

Exercise 1: Who is doing the survey?

Pastors4POR is ….

We are conducting this survey because …

Who can take part? Anyone who lives, works or prays in the Southern part of the city of Chicago or South Suburbs is considered part of our "faith-based community network" and is encouraged to respond to this survey.

What happens to my data? Pastors4PCOR and its partners will use the survey results and other information to …

Pastors4PCOR

"To inform, inspire and engage congregations in research through collaboration".

BIG DATA

Research-Ready Communities in Chicago

Exercise 3 demographics check

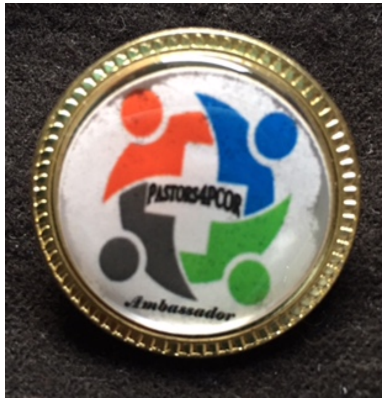

- Now take the demographic survey questions yourself. Are there categories which need changing?
- Will your community be comfortable providing this data?

4. **Please answer the following questions so that we can see how different groups in your faith-based community feel about local health issues** (all information will remain confidential):

Age: □18 – 25 years □26 – 30 years □31-40 years □41-50 years □51-60 years □61 or over

What is your gender? □ Male □ Female □ I prefer not to answer

Which one or more of the following would you say is your race?: □ Black or African American

□ White (non-Hispanic) □ Asian □ American Indian

□ Native Hawaiian or other Pacific Islander □ Alaskan Native □ Other (Please specify ___________)

Are you Hispanic or Latino? □ Yes □ No

What is the zip code of where you reside? ______________________

What is the name of the faith-based community you are a member of? ______________________

Education: □ Less than high school □ High school diploma or GED □ College degree or higher

Pastors4PCOR

"To inform, inspire and engage congregations in research through collaboration".

BIG DATA

Research-Ready Communities in Chicago

What health factors in B impact A?

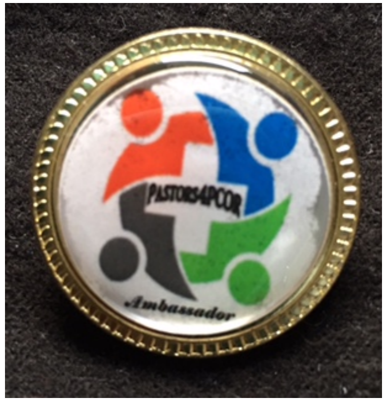

Map A: Illinois 2015: overall county ranking for health factors

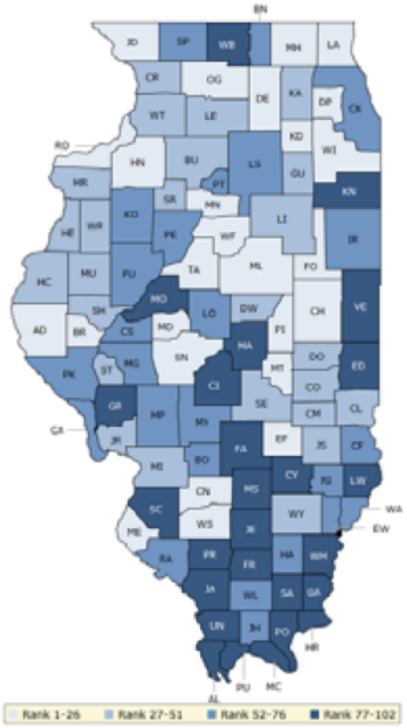

Map B:

Harvey 2015

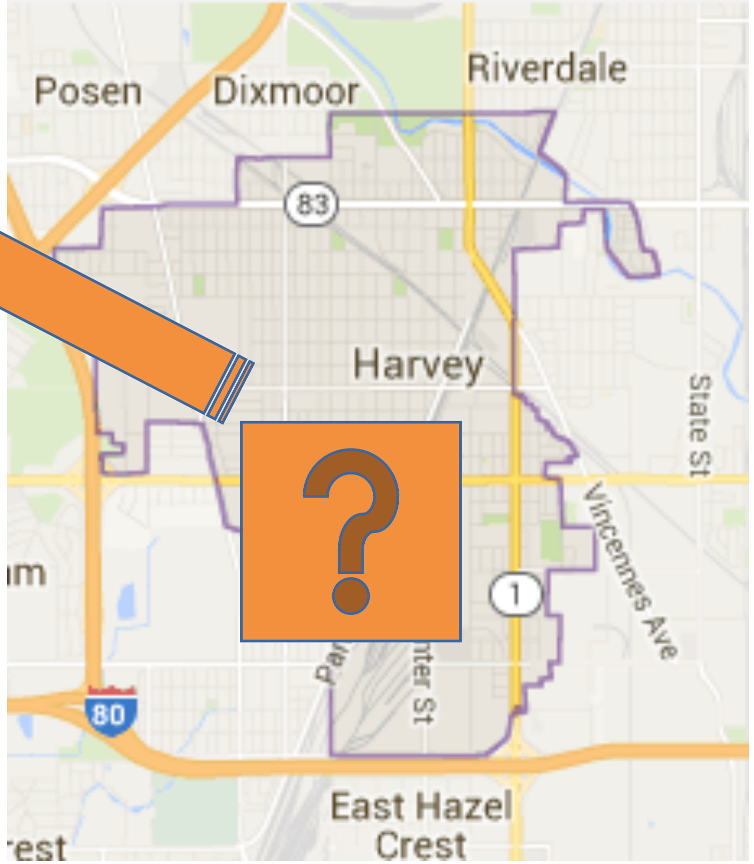

http://www.countyhealthrankings.org/app/illinois/2015/overview

Pastors4PCOR

"To inform, inspire and engage congregations in research through collaboration".

BIG DATA

Research-Ready Communities in Chicago

Survey factors

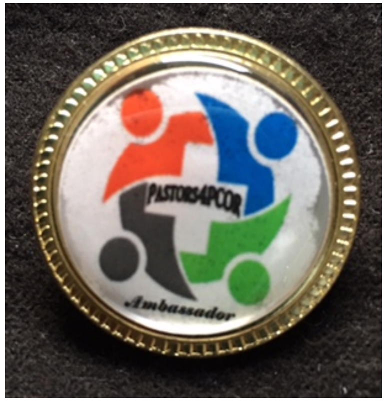

2. **Which health related factors should faith-based communities focus on to deliver "Healthier Faith-Based Communities?"** (Think about the factors which could most improve the quality of life of your faith-based community members living with the conditions you selected earlier)

   Check ***three*** from the following list and **underline** the most important:

   - ☐ Community experience of health care services
   - ☐ Access to health care and insurance (e.g. family doctor, Obamacare)
   - ☐ Clean and healthy environments environment (air, water and lead toxicity)
   - ☐ Housing and Transit
   - ☐ Diet and exercise
   - ☐ Tobacco use
   - ☐ Sex education
   - ☐ Alcohol and drug abuse
   - ☐ Caregiver support
   - ☐ Support for mental health (depression, anxiety, stress)
   - ☐ Access to healthy food
   - ☐ Dental health
   - ☐ Community Safety
   - ☐ Family and social support (e.g. community health workers; pre-school and after school)

3. In your opinion, what resources or assistance are needed for your community to address the health issues and conditions that you selected? (Please give examples)

____

____

Pastors4PCOR

"To inform, inspire and engage congregations in research through collaboration".

BIG DATA

Research-Ready Communities in Chicago

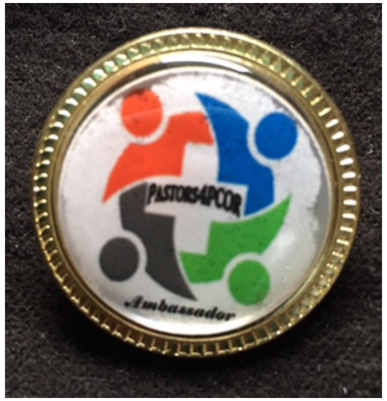

GOAL C:

"Learn how to conduct a survey on health conditions and factors impacting your faith based community"

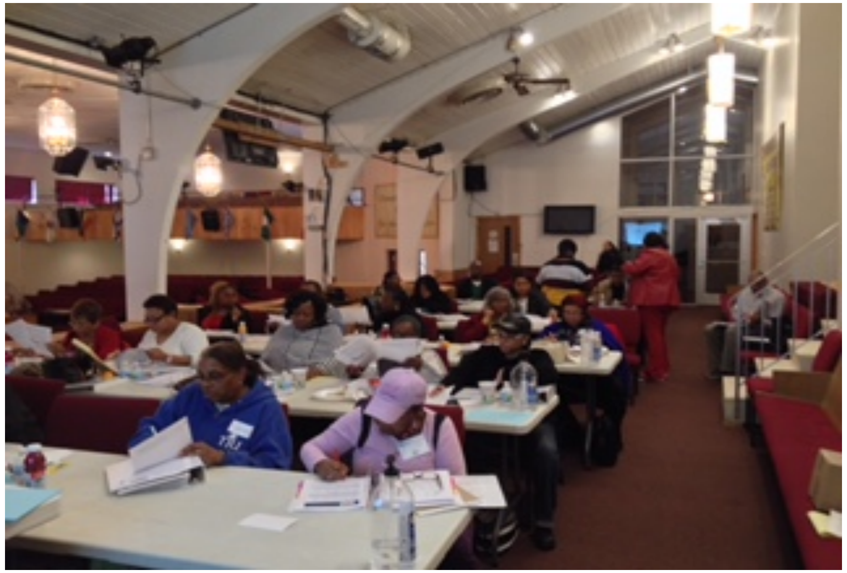

BIG DATA

Research-Ready Communities in Chicago

Activities for You and for Research Ministry

Pastors4PCOR

"To inform, inspire and engage congregations in research through collaboration".

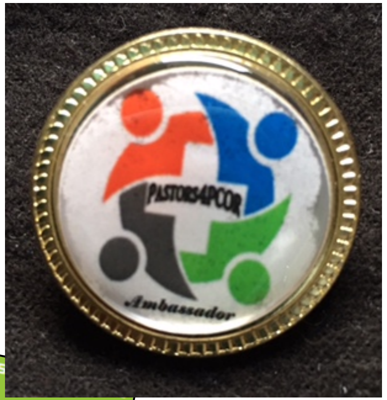

Activity sheet 6: Record sheet for recording dates and type

This activity sheet is designed to help health ambassadors record all the activity you
faith based communities and encourage members of your community to complete th
Please complete and return to your survey mentor.

Name of Church:
Zip code of Church:
Name of health ambassador:
Name of survey mentor:

Activity completed	Date	Estimated numbers reached
[e.g. advert in newsletter; announcement by pastor on church; announcement at event; email; tweet; conversation with friends and family]		

Appendix 2: Activity Sheet 5: survey process and support.

Activity Sheet 5: The survey process and survey mentor support.

This activity sheet is designed to help you work through the process of advertising the survey to your faith based communities and encouraging members of your community to complete the survey. It also outlines the role of your survey mentor in supporting you throughout this process.

Step 1: Make sure you know who your survey mentor is and have provided a current email address. Survey mentors will be matched with ambassadors at session 3 on 23 January 2016. Mentors will reach out by email to you the week after session 3 and agree support call via email, phone or face to face to talk through four steps.

Step 2: Your survey mentor will contact you at an agreed time during **February 2016** to discuss with you the best ways to advertise the survey to your faith based community and ensure community members complete the online or hard copy versions. In preparation for this conversation ask yourselves: *How does my church currently communicate with its members? What is the most effective way to communicate the survey?* It may be that the most effective way of engaging members of your community is an advert in a newsletter (like figure 1), or public announcement or a combination of both.

To help you reach out, we have created a poster template using WORD. (see reverse of this sheet). Ask your mentor if you would like to receive the WORD template. Feel free to adapt the template. You should use Activity Sheet 6 to record all the activities you do **during March** to disseminate the survey as well as the number of times and dates completed.

Figure 1: example advert for newsletter

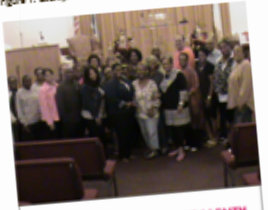

Step 3: When you speak to your mentor they will ask you to identify **an event in your faith based community in March** where you will be able to hand out the survey and collect it in. Your mentor should attend this event. To support you they will bring along the hard copies of the survey you need plus pens and be available to field questions. At the end of the March event survey mentors will collect completed surveys and your completed Activity Sheet 6 records.

Step 4: Early **in April** your mentor will arrange a follow up call or email to ask you about your experience of disseminating the survey and to share comments made by community members. Keep a note of the questions people asked you throughout the process or the reasons they gave for completing or not completing the survey. Think about whether you would be prepared to repeat the process in two years' time. Come along to session 4 at the end of April prepared to discuss your experience. Survey results will also be presented at session 4.

7

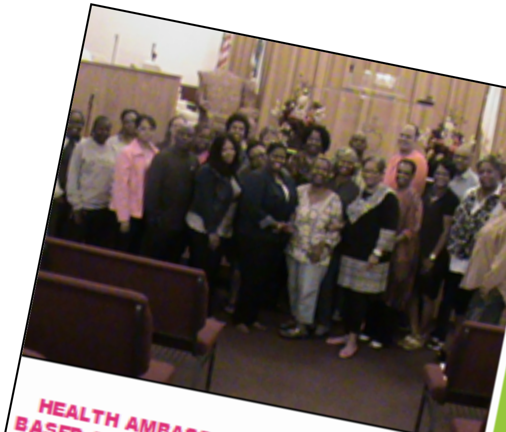

HEALTH AMBASSADORS FROM 14 FAITH BASED COMMUNITIES INVITE YOU TO TAKE THE FAITH BASED COMMUNITY SURVEY.

WWW.FAITHBASEDCOMMUNITYSURVEY

Compete the survey at the {insert event}
Date:
Time:
Venue:

members of faith based communities have created their own based survey as part of their training to become health ssadors. The goal of the short survey is to ask you to identify alth conditions and factors which impact your community" nswers will help the Health ambassadors and Pastors4PCOR orative partnership of churches, university health rs, and health professionals) to partner with researchers improve health outcomes.

does not share any rmation ealth

Pastors4PCOR

pcori

What's

You tell us about the health issues affecting your faith based community

So that health researchers understand the health conditions and factors that impact your community

And research can answer questions that matter to you

FOR MORE INFORMATION AND A HARD COPY OF THE SURVEY CONTACT:
Ambassadors name

Pastors4PCOR

"To inform, inspire and engage congregations in research through collaboration".

BIG DATA

Research-Ready Communities in Chicago

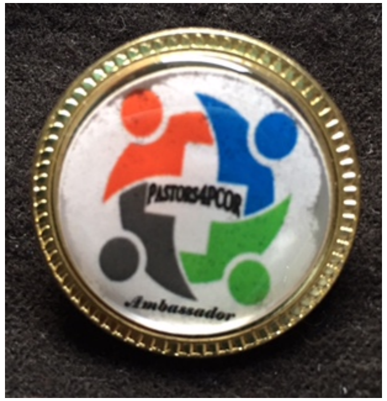

Our Next Steps

- ✓ Connect with your survey mentor
- ✓ Inform and inspire your faith based communities to engage with research
- ✓ Disseminate the faith based community health survey
  - Read info in your folder,
  - Use the guidelines in Activity Sheets 5 and 6 to engage your communities
- ✓ Complete the session evaluation form

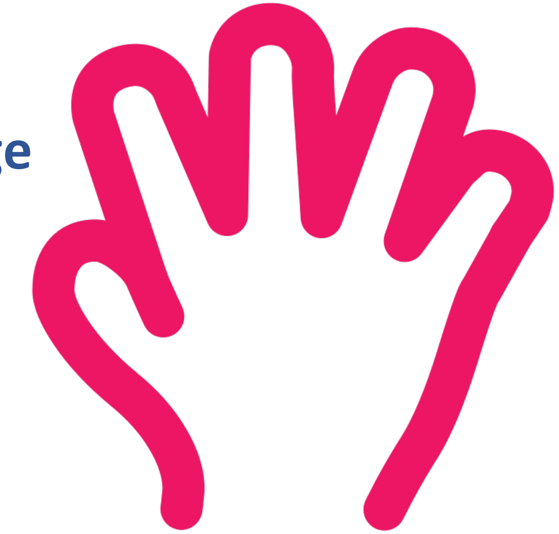

BIG DATA
Research-Ready Communities in Chicago

Pastors4PCOR

"To inform, inspire and engage congregations in research through collaboration".

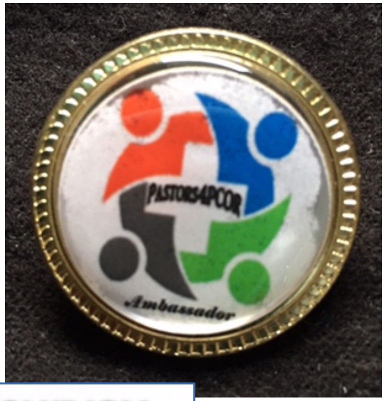

OUR DATA – INFORMATION WE CAN USE & SHARETO MAKE OUR COMMUNITIES HEALTHIER

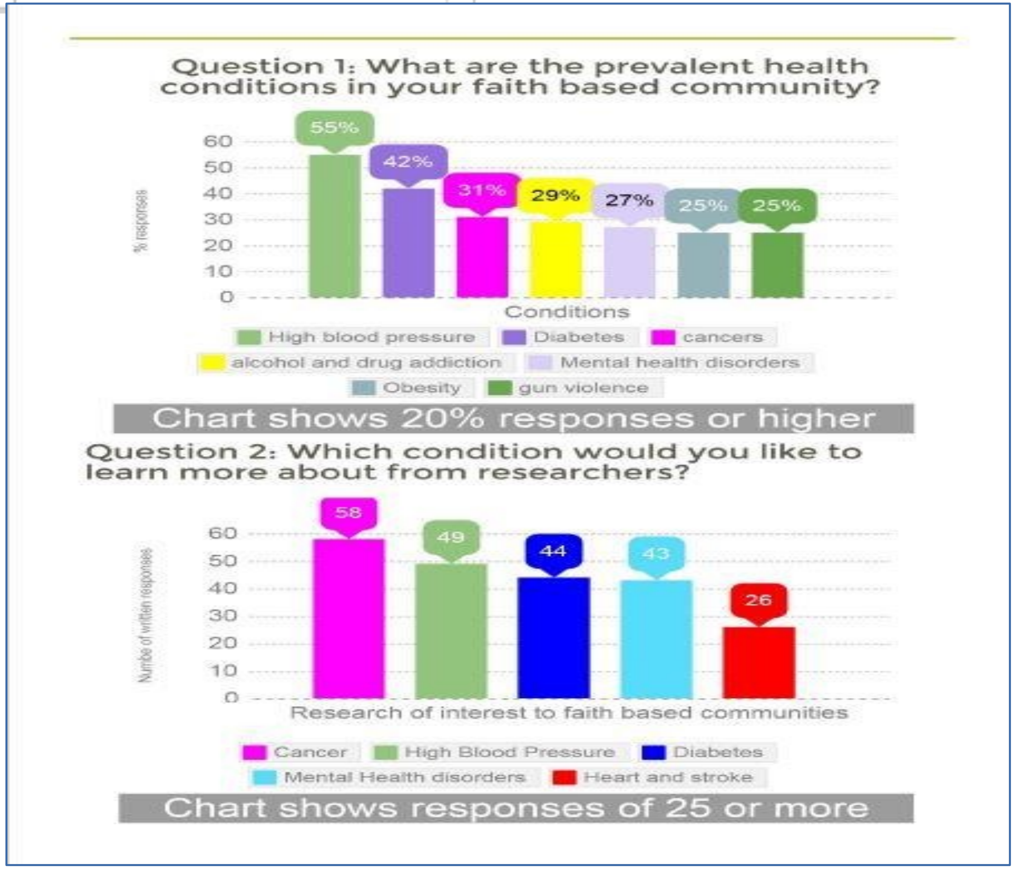

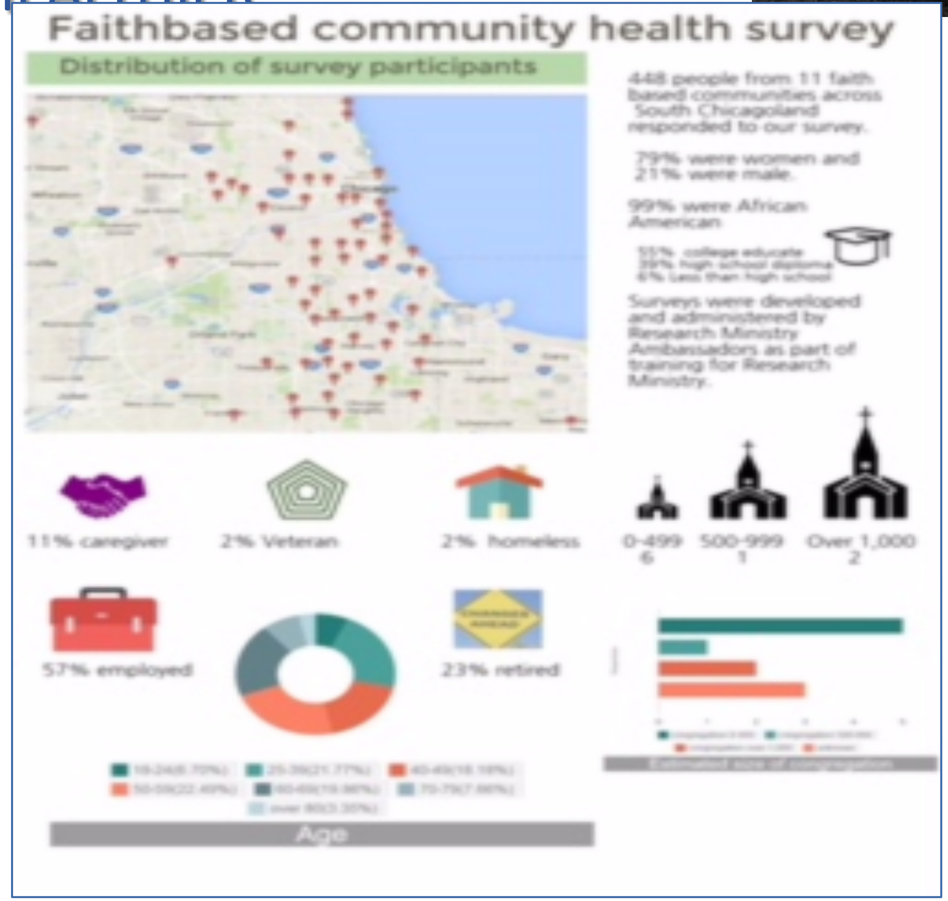

Pastors4PCOR

"To inform, inspire and engage congregations in research through collaboration".

BIG DATA

Research-Ready Communities in Chicago

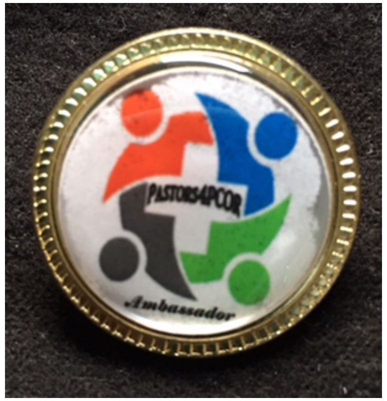

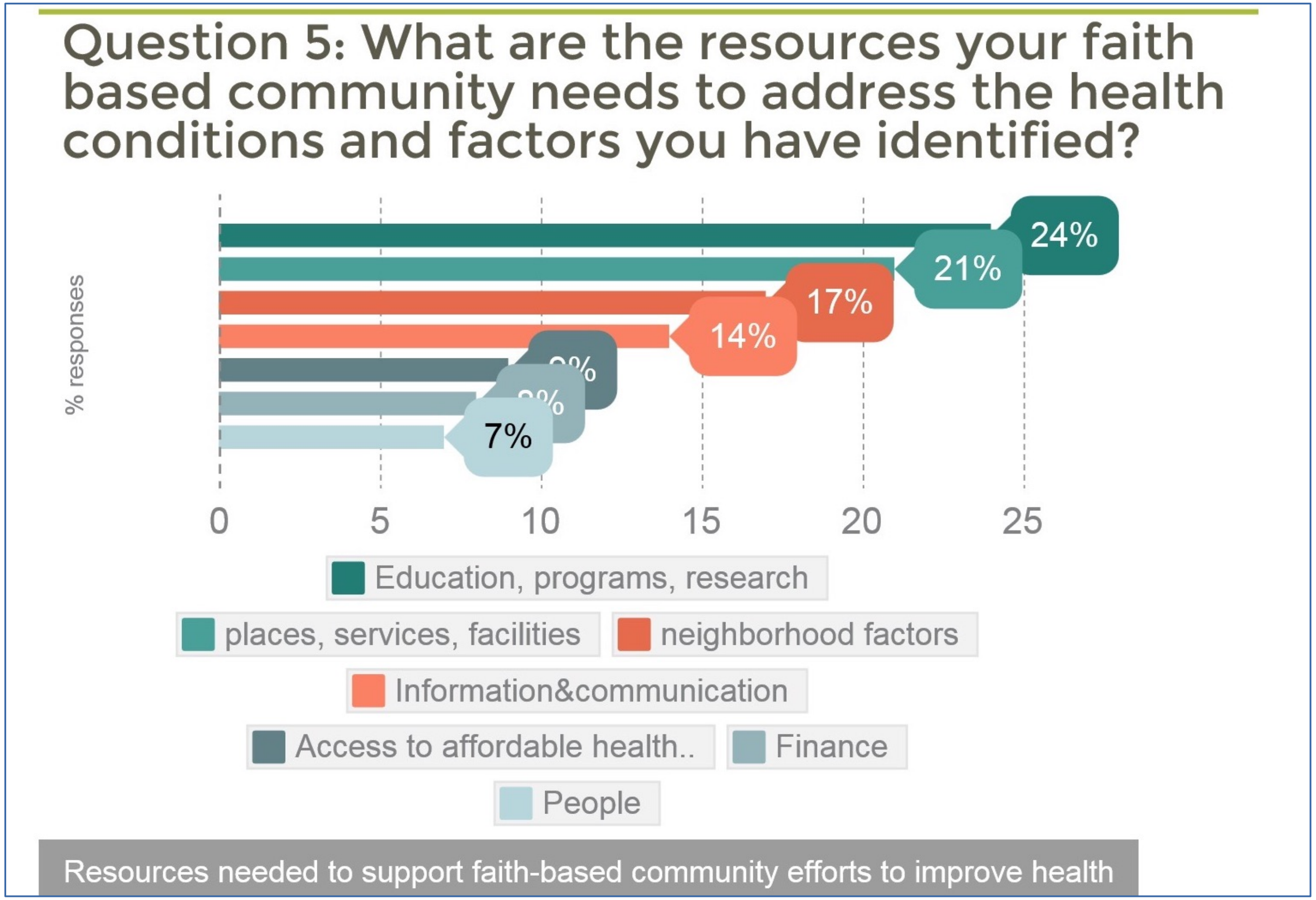

Pastors4PCOR

"To inform, inspire and engage congregations in research through collaboration".

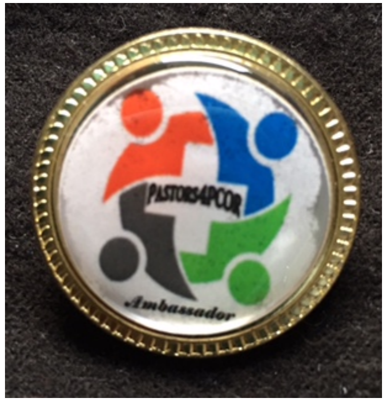

BIG DATA
Research-Ready Communities in Chicago

Graduation, Celebration, Appreciation, Affirmation
October 2016

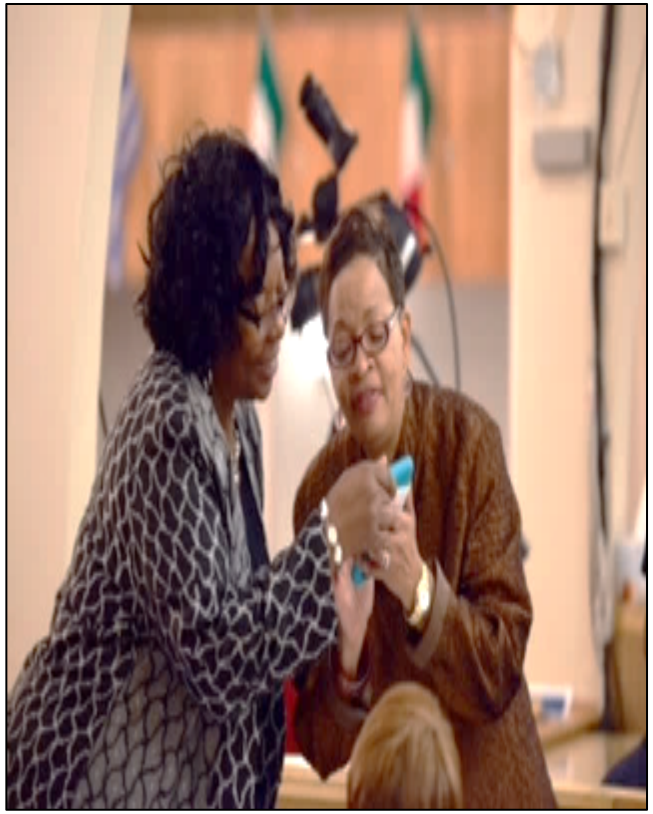

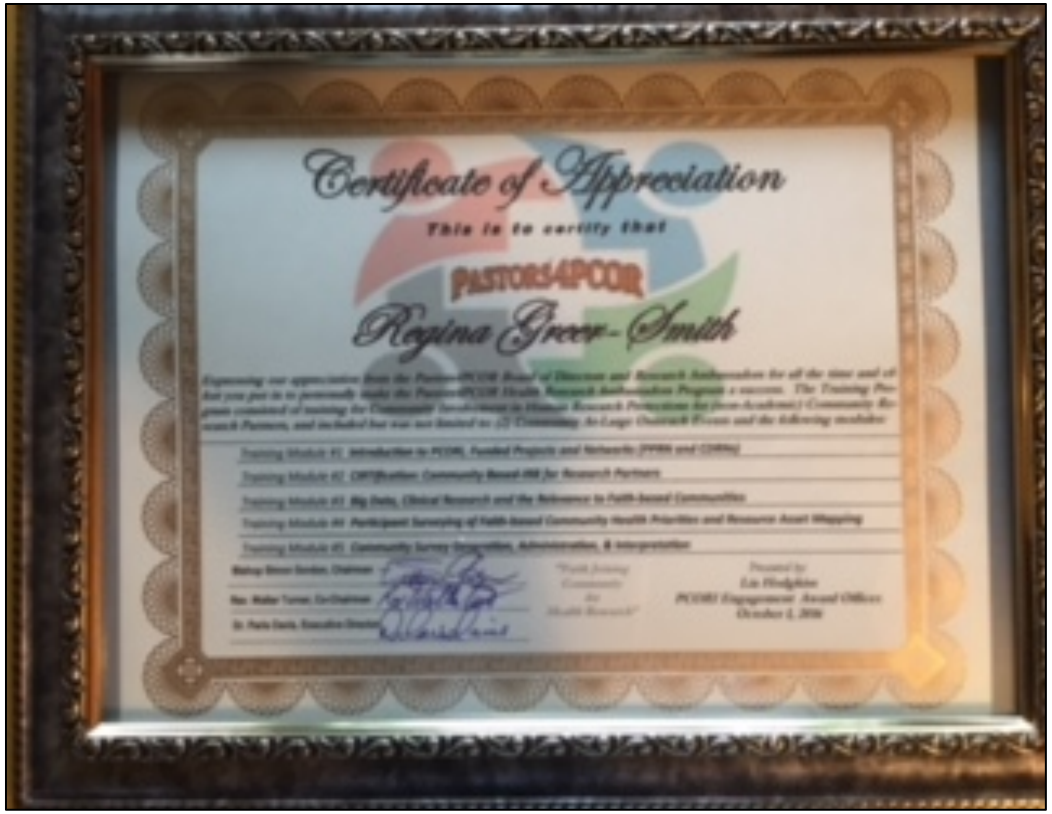

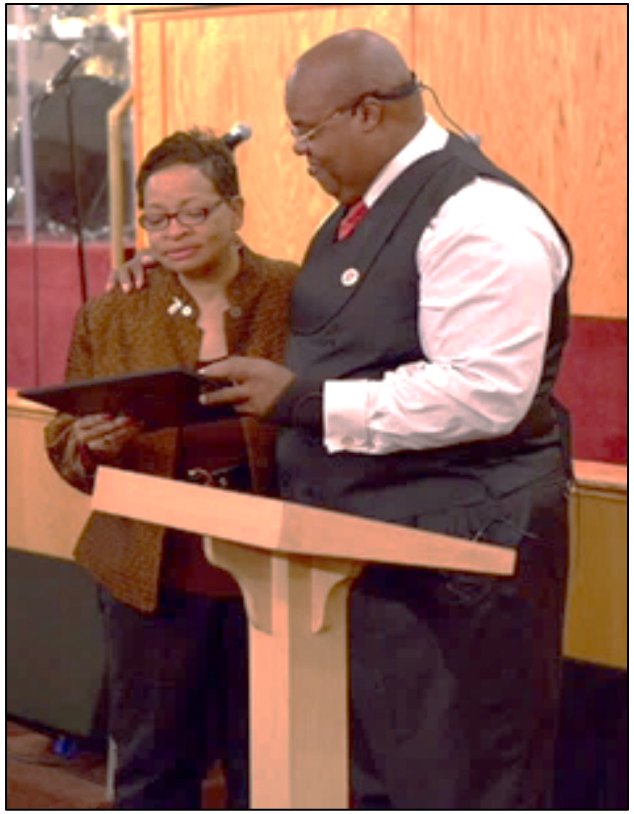

Pastors4PCOR

"To inform, inspire and engage congregations in research through collaboration".

BIG DATA

Research-Ready Communities in Chicago

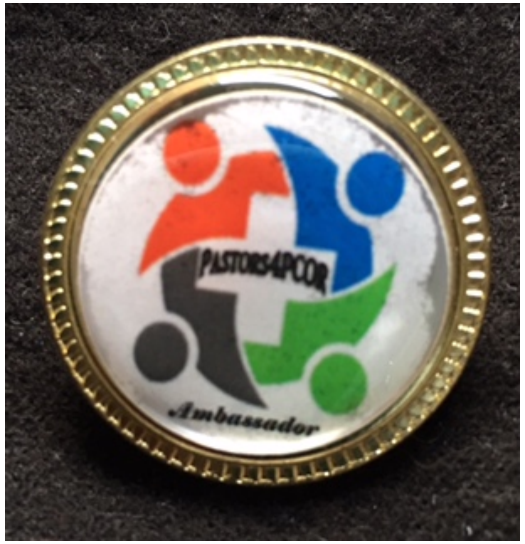

Pastors4PCOR Research Ministry Ambassadors
First Faith-Based Research Ministry Organization
Graduation- Cohort I October 2016
"Our Journey Continues"

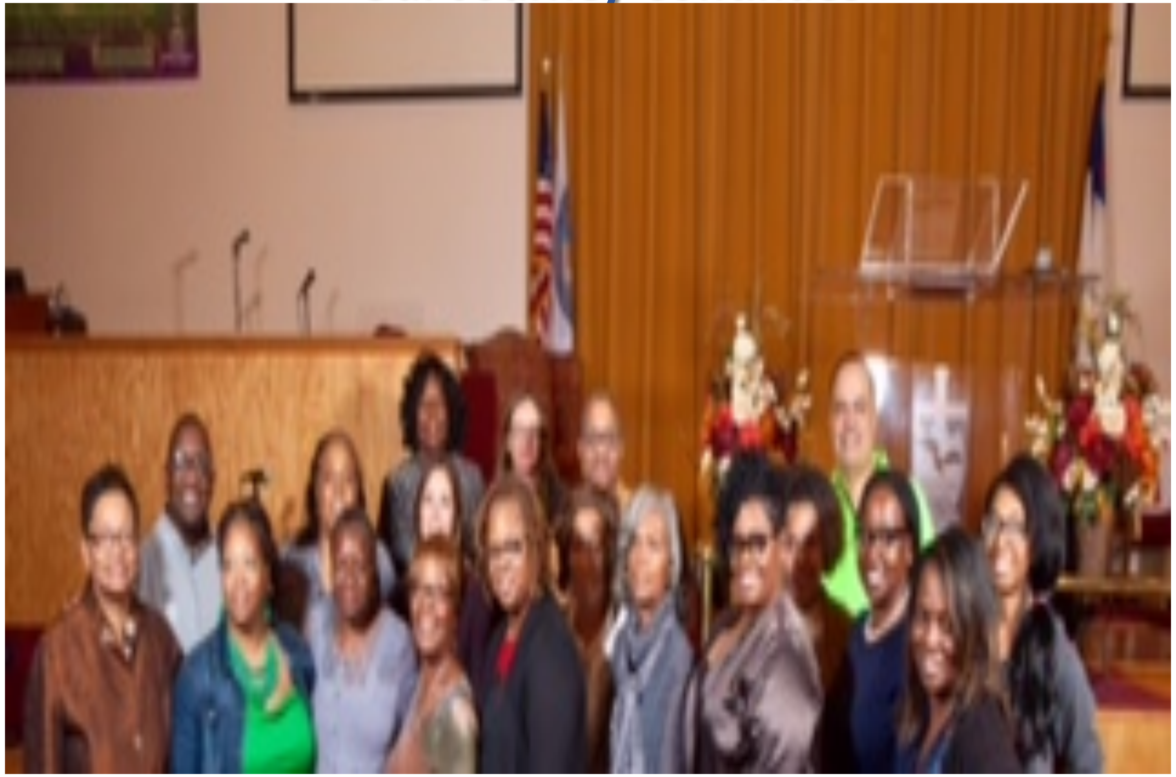

Plenary Session III: Data as the Driver – and Passenger – for the Community-Engaged Research Vehicle

Data as the Passenger: The Importance of Identifying What Data Matters to the Community

Courtney Clyatt
cclyatt@pcori.org
Program Officer, Patient-Centered Outcomes Research Institute, Pipeline to Proposal Awards/Engagement Awards
Washington, DC

There are currently two programs at the Patient-Centered Outcomes Research Institute (PCORI) that provide funding for communities to help shift the research paradigm to ensure communities have a voice and input into the research process. These programs allow the communities, particularly patients, to drive the research with data as the passenger. Factual data (such as measurements and statistics) allows people to make informed decisions. [Slide 1] Health information is particularly meaningful to individuals in the community, though that data is often complicated and must be refined. [Slide 2] Unless the data is both pertinent and digestible, it is of little value to target communities. [Slide 3]

This is one of the reasons communities need to be a part of driving research to facilitate their own informed decisions about healthcare. [Slide 4] PCORI programs aim to engage patients, clinicians, and caregivers in the entire research process, [Slide 5] from topic prioritization to proposal review with the Merit Review Program to dissemination and implementation of results. [Slides 6, 7] PCORI funds research focused on comparative effectiveness: comparing two treatments or two diagnostic tools to aid patients in identifying which option will work best for them based on information provided regarding medication side effects, potential benefits and/or harm associated with the chosen treatment, optimizing individual health outcomes, and exploring how clinicians and care delivery systems can work together to assist in the patient's decision-making process.

There are two funding programs at PCORI. [Slide 8] These awards support projects that bring together patients, caregivers, and clinicians, allowing them to collectively engage in research. Some projects have focused on informing, training, and developing community involvement in health research.

Another program, the Pipeline to Proposal Awards, seeks to build a national community of patients, stakeholders, and research partnerships with the expertise and the drive to involve their communities in the research development process. [Slide 9]

Several projects were designed that allowed patients to drive the data and the development of a research study. [Slide 10] One of these was in New Jersey and focused on sickle cell anemia. Outreach efforts were made via Facebook and through support groups in order to engage people and identify the data they considered important. One common issue involved pain management and the necessary pain medications. Another project called "Cystic Life" focused on cystic fibrosis and looking at cardiovascular exercise as a treatment option, and this effort was patient-led. Project participants engaged their community through message posts, blog posts, and Twitter. A third project called “Sole Survivors” focused on melanoma research. The project grew out of a melanoma patient support system and was started by a 20-something female who realized she might be considered an anomaly. She realized that, as a patient, she had the information that researchers might need in order to direct additional research, and she shared several stories about knocking on doors to find the right partner. Funding from PCORI is one of the things that opened doors because she came with the money, and researchers were intent on hearing from her. A fourth project, #LCSM, focuses on lung cancer and lung cancer treatment after surgery. This is a clinician-led project where the project lead basically sourced his research question as well as some data from using a Twitter chat. Other methods for accessing the the community and obtaining data [Slide 11] include message boards, Twitter chats, Facebook, Town Halls, World Cafes, surveys, and conferences. Familiarity with the target community and understanding each community’s unique health concerns and priorities facilitiate engagement which further dictates the research focus. [Slide 12] In the pre-planning stage before a research project is developed or even before a research question is identified, engaging with the community in capacity-building is more likely to involve patients and community partners who not only help drive the research, but also help disseminate study findings and implement programs based on those results. [Slide 13] It is important to consider that if the data system does not reflect the full patient experience and relevant health issues, that data system may not be fully integrated without patient perspective. To maximize the impact that research can have on a given community, patient input must be integrated into the process. Only then will researchers truly optimize the likelihood of improving human health.

Declarations

- The presenter has no financial competing interests related to this presentation.
- All authors have approved the manuscript for submission
- The content of this presentation and transcript has not been published or submitted for publication elsewhere.
- This presentation was organized by the Meharry-Vanderbilt Alliance with support from NCATS and the NIMHD of the National Institutes of Health under award number R13TR001694

Data as the Passenger: The Importance of Identifying What Data Matters to the Community

Courtney Clyatt
Program Officer, Pipeline to Proposal Awards/Engagement Awards

CEnR - Advancing the Science of Community Engaged Research
September 15, 2017

Definition of Data

- 1 :factual information (such as measurements or statistics) used as a basis for reasoning, discussion, or calculation
- 2 :information output by a sensing device or organ that includes both useful and irrelevant or redundant information and must be processed to be meaningful

What makes data useful to patients when making healthcare decisions?

- Data is useful to patients when it provides factual information (such as measurements or statistics) that patients can use as a basis for reasoning, discussion, or determining if a test or treatment is right for them and their health issue.

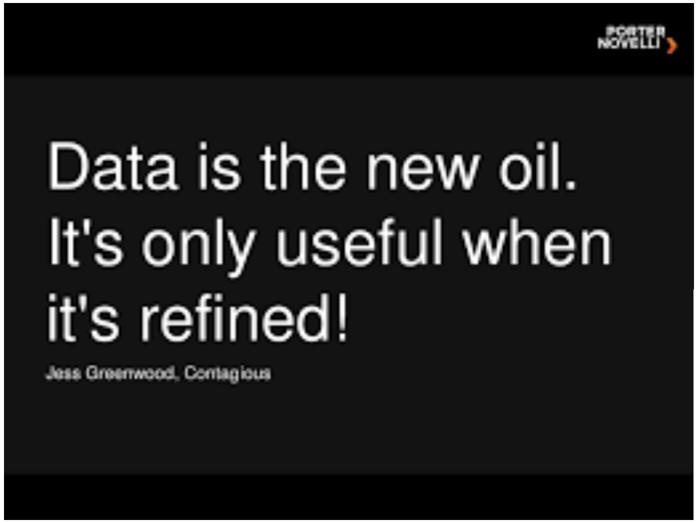

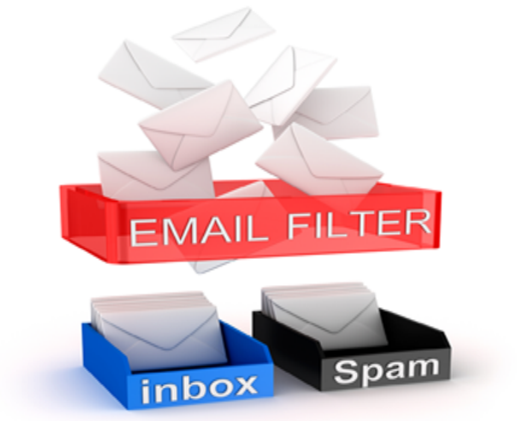

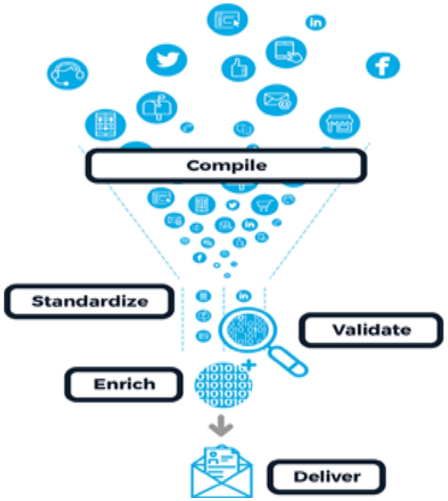

PCORI Vision & Engagement

- Vision
  - Patients and the public have information they can use to make decisions that reflect their desired health outcomes.

- PCORI Engagement
  - We engage patients, caregivers, clinicians, insurers, employers and other stakeholders throughout the research process

Engagement as a Path to Useful, High-Quality Research

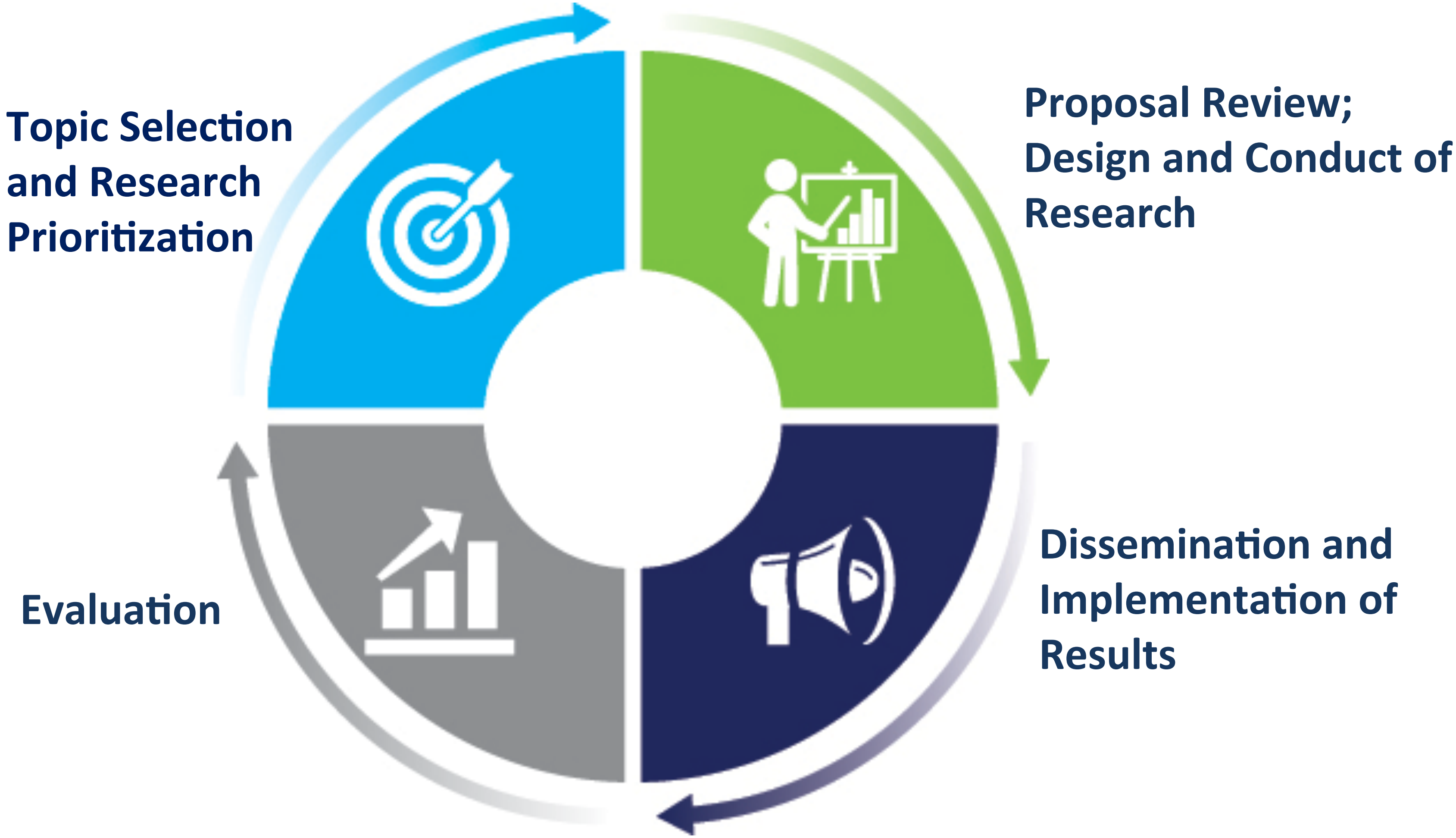

PCORI Vocabulary

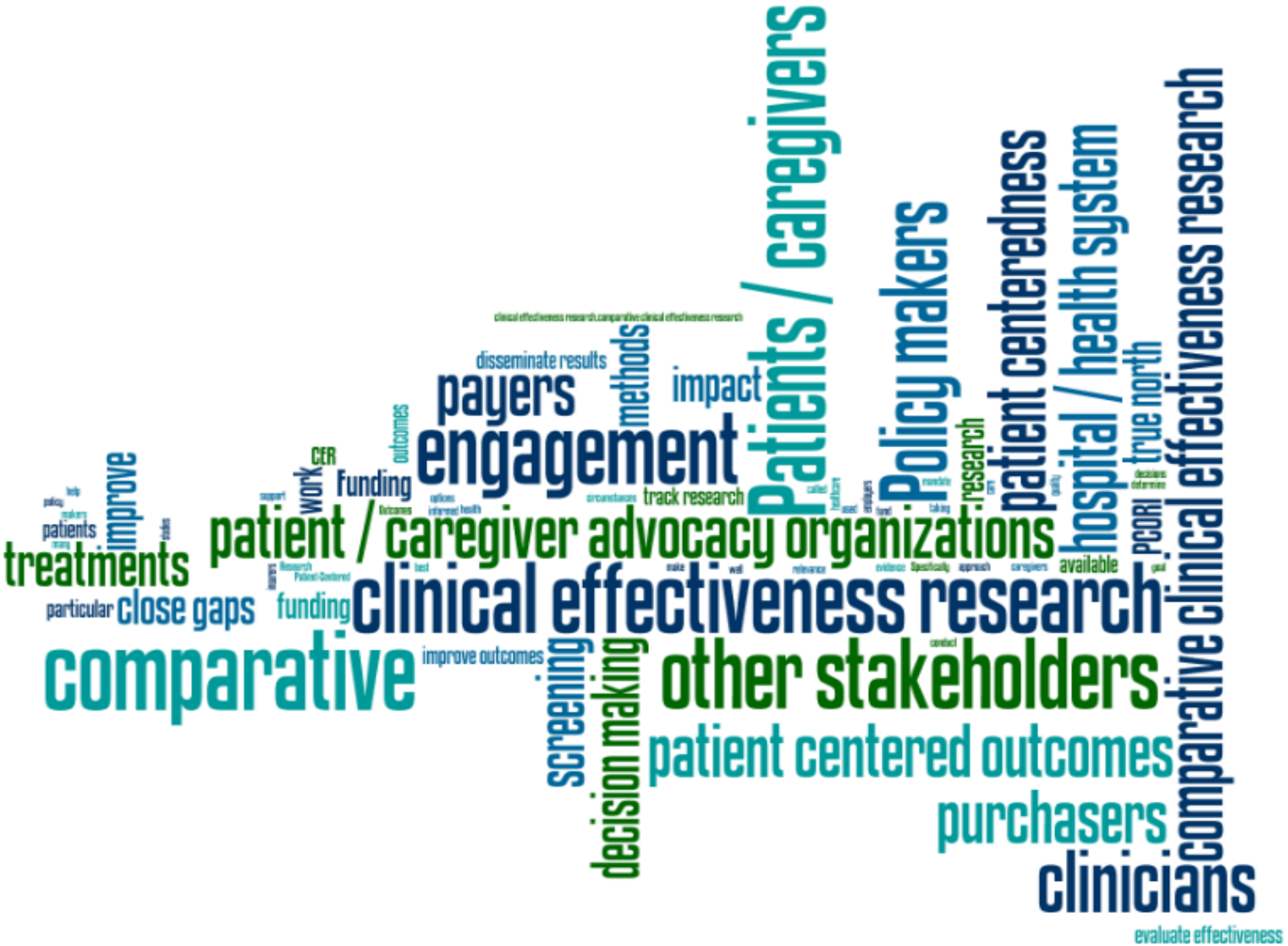

PCORI's Work Answers Patients' Questions

Given my personal characteristics, conditions and preferences…

"What should I expect will happen to me?"

"What are my options and what are the potential benefits and harms of those options?"

"What can I do to improve the outcomes that are most important to me?"

"How can clinicians and the care delivery systems they work in help me make the best decisions about my health and healthcare?"

Two funding programs that prepare communities for research

- **Eugene Washington Engagement Awards**
  - These awards support projects that encourage active integration of patients, caregivers, clinicians, and other healthcare stakeholders as integral members of the patient-centered outcomes research/clinical effectiveness research (PCOR/CER) enterprise.

- **Pipeline to Proposal Awards**
  - Build a national community of patient, stakeholder, and researcher partnerships that have the expertise and passion to participate in patient-centered outcomes research within their communities
  - Build capacity and cultivate the development of proposals with sound scientific rigor and robust patient engagement.

Just as Demographic and Socioeconomic Factors are Major Determinants of Health…

- The type of data that is important to the community will vary by access to healthcare, socio-economic status, and demographics.
  - to determine what information the community values as important, you may need to think outside of the box.

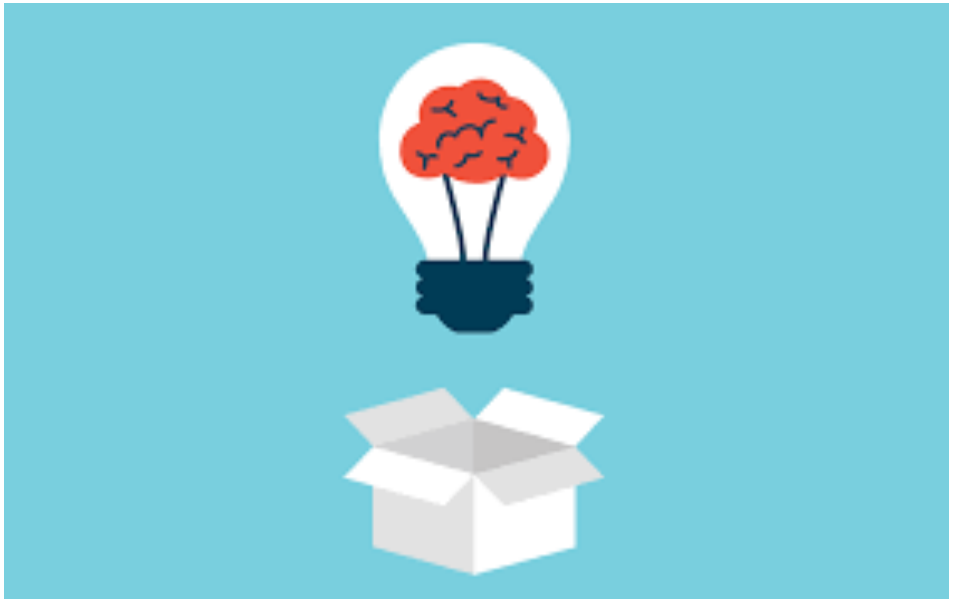

Examples of Projects Where Patients Drive Data in the Development of a Research Study

- We'll take the Village – Sickle Cell Association of NJ is conducting outreach via Facebook and support groups
- Cystic Life – patient led project focusing on Cystic Fibrosis and cardio exercise as a treatment
- SolSurvivors – patient/survivor-led project focusing on melanoma research
- #LCSM – clinician-led project focusing on lung cancer treatment, conducting outreach, sourcing data and determining a research question via Twitter

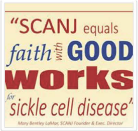

The Sickle Cell Association of New Jersey
@sicklecellnewjersey

September is National Sickle Cell Disease Awareness Month

SOL SURVIVORS

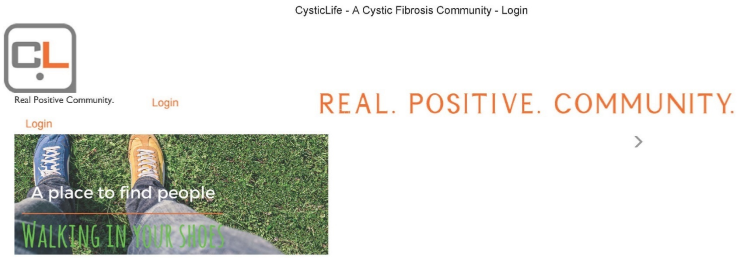

#LCSM Chat

Strategies that Pipeline and Engagement Awardees Use to Share and Receive Data from their Community of Stakeholders

- Message Boards
- Twitter Chat
- Facebook
- Town Halls
- World Cafes
- Surveys
- Conferences

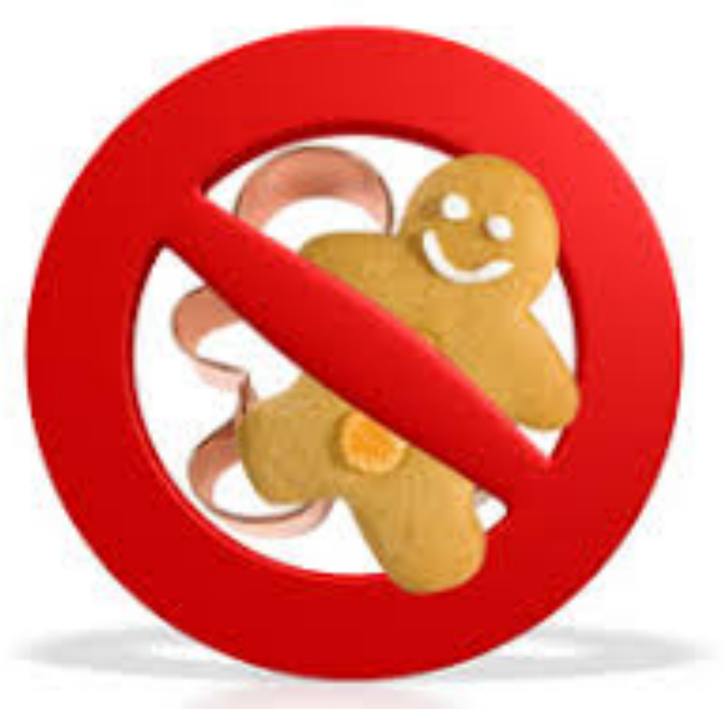

Community Engagement Strengthens the Research Enterprise

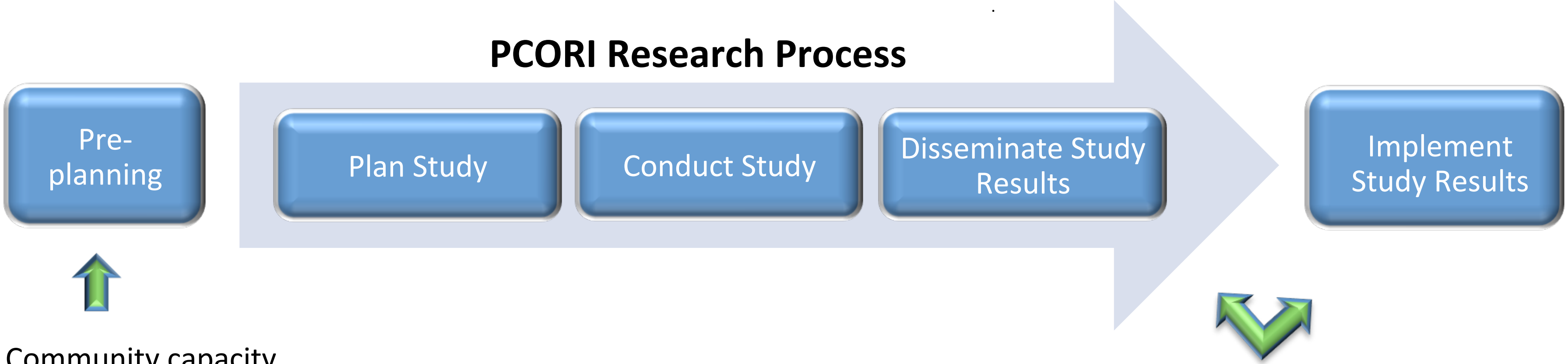

Community capacity building before a study is planned enables patients and the community to drive the research.

2) It has been shown that when patient and community partners are engaged early on and throughout the research process, they are more likely to help in the implementation and dissemination of study results in their communities.

Importance of Including the Community Voice and Lived Experience in the Development of Data Systems

- If the data system isn't reflecting the full patient experience living with that health issue, then is it a fully integrated data system?

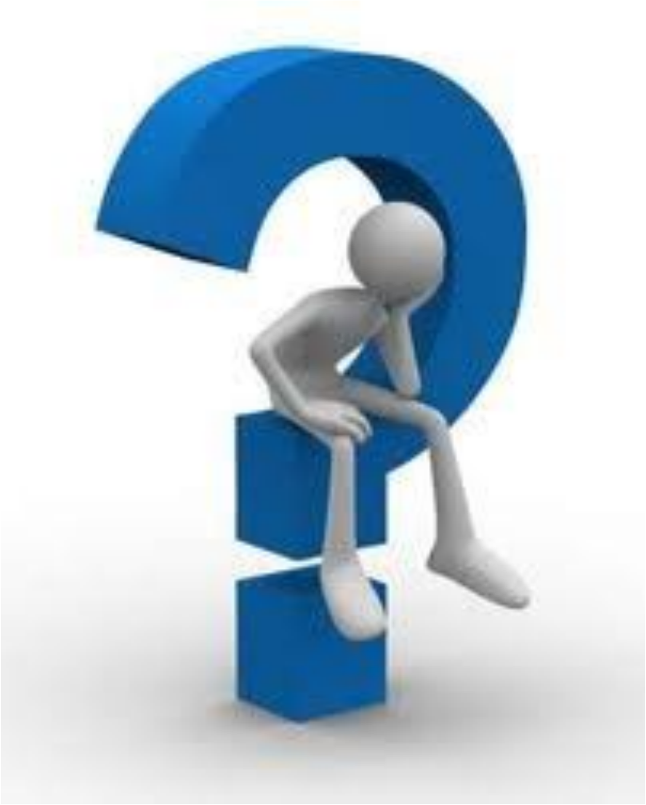

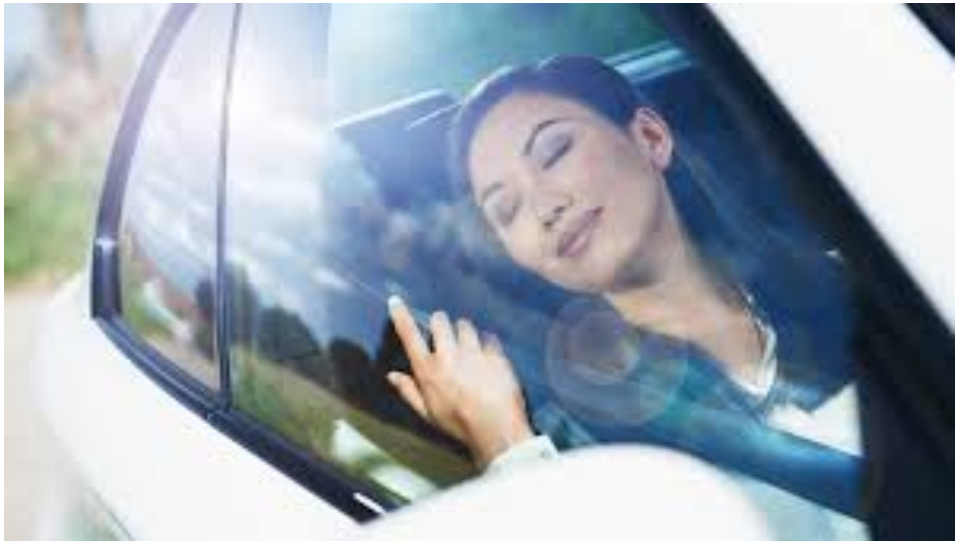

Little Data, Big Data: Translational Research Partners Across the Spectrum

Jonathan N. Tobin
jntobin@cdnetwork.org
President/CEO, Clinical Directors Network, Inc.
New York, New York

Abstract

Dr. Tobin presents translational research reliant on "small data" (a few participants engaged in a behavioral intervention implemented by community health workers) and "big data" (an observation in electronic health record data over 10s of thousands of pregnant women of the life course and trans-generational patterns of metabolic disease). He observes that translational research critically depends on listening closely to the needs of all stakeholders — priorities, obligations, and driving incentives — which entails deep engagement. He emphasizes the need to define outcomes across the full biomedical research spectrum, from the biological, through the clinical and patient-centered, and to the health services public health measures. He and his colleagues enable deep engagement in grant writing, for example, using an approach that involves discussion teams consisting of a laboratory investigator, a health services researcher, a clinician, and a patient. Topics the groups discuss include patient engagement, patient recruitment, incorporating patient needs, defining patient outcomes. He concludes that this allows research to embed biological measures within well-characterized clinical populations.

[*Jonathan Tobin*]

Good morning, everybody. I want to thank Consuelo [*WIlkins*] and Charles [*Mouton*] and Rhonda [*Kost*] and my other colleagues on the Planning Committee for giving me the opportunity to tell some stories, and what I would like to do this morning is tell two stories through their data, hence the title "Little Data, big Data." These stories are about the translational research partners across the spectrum. I'm going to do it a little bit in reverse from conventional science, because typically we would talk about the methods and then the results, but I'm going to talk about the results first, and then go back to some of the methods and try to get some insights on what we can learn about translational research partnerships by looking at these two cases, a study in little data and a study in big data. I want to acknowledge funding from NCATS, PCORI, The Sackler Foundation, and AHRQ all of whom contributed to the data that I am going to be sharing. [SLIDE 2]

This translational research spectrum in a number of different manifestations should be pretty familiar to everybody. [SLIDE 3] It runs from T0 to T7, and every talk I go to adds another level of T, but for the purposes of this discussion, we will end at T4 with translation to community with population level impact.

How did we do this? [SLIDE 4] We began with two different infrastructures, the practice-based research network infrastructure of the Clinical Director's Network, and the CTSA infrastructure, The Rockefeller University Center for Clinical and Translational Science. Those two infrastructures have some common elements in them in terms of training and in terms of research, but they have some real differences in terms of focus. The work in the practice-based research network is really focused on clinical outcomes and comparative effectiveness research and training investigators who are practicing clinicians, whereas with our CTSA engagement award, we are training patients to become investigators and colleagues in that process, and the CTSA academic training focuses on clinical scholars, postdocs, medical students, physician scientists. My colleague, Dr. Kost [*Rhonda G. Kost, MD*, *Clinical Research Officer at The Rockefeller University*] has designed and described in her publication, "The Community-Engaged Research Navigation Process," an open cycle that allows investigators and teams to join in the research enterprise at any one of these difference stages and move into a collaboration that, in this instance, produces community engagement, community-engaged research, and then comparative effectiveness research, and patient-centered outcomes research. I think one of the unique additions to the model that I am going to discuss is actually embedding T0 or mechanistic questions within the framework of comparative effectiveness research and clinical outcomes study.

Let me move into these two different case studies. [SLIDE 5] The first is a study called "The Patient-Centered Comparative Effectiveness Study of Home-Based Interventions to Prevent Community-Associated MRSA Infection Recurrence." It is a mouthful to say, so we call it "CAMP." We have a very wide range of stakeholders [SLIDE 6]: patients, caregivers, family members of patients, community health workers, practicing clinicians, academic and laboratory investigators, statisticians, and informaticians. We have representation from several large public hospitals in New York City and several large federally-qualified health centers, our colleagues at Rockefeller and CBN, as well as a number of different academic stakeholders [SLIDE 7]. We had this one instance that, I think, really articulates the value of patient engagement. At every one of our monthly meetings, we have case presentations by a clinician, and in this instance, one of our most active clinicians was presenting a case that he had actually shared with us previously, and he shared with us that his patient in this case is very upset with him now because she has had yet another recurrent infection despite incision and drainage, despite antibiotics, and she doesn't want this to come back. Further, her sister was visiting and came down with the same infection. So, that really galvanized the group to try to understand the predictors of recurrence, and we realized that it was probably the most important area of focus that we, as a research

team, could address. This question really derives from a single patient expressing her frustration with her care experience to a very concerned clinician, who then shared that frustration, and it generated this intense conversation. When we looked at the CAMP data, we saw that this really was quite significant in the outcome study. In the observational cohort, 40% of patients, in spite of most of them receiving and following CDC-defined treatment guidelines, were developing recurrent infections if they had methicillin-resistant Staph or methicillin-sensitive Staph infections. [SLIDE 8 and 9] So, obviously, this required our focus. We looked at the literature and identified a study conducted in the hospital intensive care setting that identified one strategy that was associated with significant reductions in an ICU setting of MRSA infection. So, we asked the question - could we train promotoras and community health workers to provide home visits and during those home visits, could they, in fact, train patients to implement the same protocol that worked so well in the ICU setting? [SLIDE 10] Our control condition is usual care, so everybody in these participating sites [SLIDE 11] across New York City and Westchester County received the CDC guidelines care. Everybody had a home visit where we conducted surveillance measures. [SLIDE 12] The experimental group, however, received materials related to decolonization (that is removing evidence of the bacteria from parts of the body that are not infected) and decontamination (removing the bacteria from surfaces in the household to reduce the overall risk of infection). We went into the households of patients (278 are randomized in this PCORI-supported study) [SLIDE 13] and we surveyed 13 surfaces that are high-touch areas and we ask all of the household members if they would be willing to provide us with swabs of their nares, their axilla, and their groin so that we could determine who in the household is colonized in addition to the patient. Now, I am going to tell the story through the results. We have a colleague across the street at another CTA-funded institution, Weill Cornell, who is interested in meta-genomics. [SLIDE 14] We have our colleagues at Rockefeller who are interested in characterizing the molecular epidemiology of the specimens that have come through from these household studies. [SLIDE 15] Remembering the translational research spectrum, this research question started in the T0-T1 space with the investigator interested in mechanisms of recurrent furunculosis (that is the skin and soft tissue infection that keeps coming back). We moved into the T3 space to try to understand what are the clinical predictors that are responsible for this patient's frustrating recurrence experience. In fact, it is the patient who really generated the research question.

The study is currently underway, and we have enrolled 135 of the 270, so we are at the halfway mark. We are behind in our recruitment, but this is a challenging disorder to identify and recruit. [SLIDE 16] The key findings here are that 65% of patients in this study with the skin and soft tissue infection that has been diagnosed

microbiologically to be caused by Staph aureus are also colonized in one or more locations and 33% of the people they share their home with are colonized. Perhaps most strikingly, 60% of the households are contaminated (have evidence of Staph aureus on the high-touch surfaces), most frequently on the kitchen floor, on the toilet seat, and on the bedroom floor. So, maybe it is true - you can catch this from the toilet. We don't know that yet, but we will know when we do the final analysis. It is a reminder that this kind of infection really is a family affair. It is a household matter. It is not the problem of an individual patient, so it needs a solution and a strategy that addresses the entire household. So, this takes us into the T3-T4 space, where the clinician investigator becomes involved. [SLIDE 17] We showed him pictures of MRSA to a community leader who happens to be a barber, and he said, "Well, I see these things on the scalp and the neck all the time - are they contagious?" We realized in that brief conversation that we really do need to talk with people outside the four walls of the healthcare delivery system. If you look at the Google maps (*lower left, slide 17*), around three partner health centers, those dots represent just some of the barber shops, beauty salons, and nail salons in the area. Through the community leader, we had the opportunity to go into and provide essentially a continuing barber education course, CBE, on infection, infection prevention, and control, and we found statistically significant increases at one month and three months in what people learned and what they retained and what they told us they planned to do in terms of preventing infection.

The currency is always education. While barber education is new to us, continuing medical education has been a major engagement currency that we have used across all of our research projects and we feel that is a very, very critical dissemination strategy. I will share with you a flyer where you can find all of those CME-accredited projects. [SLIDE 18]

That was the little data case. Let me take you now to the big data case - Obesity, Cardiometabolic Risk, and Adolescent Pregnancy: Building a De-Identified Electronic Clinical Database to Examine Biological and Social Determinants of Nutritional Status, Pregnancy, and Birth Outcomes. [SLIDE 19] This project grew out of an NIMH-funded clinical trial that randomized 14 hospitals and FQHCs in New York City to usual care prenatal care (which is individual care) or group prenatal care, using a program called "Centering." The intervention group, (the group prenatal care) had significantly reduced the incidents of pre-term birth, low birth weight, and small for gestational age for these young mothers. [SLIDE 20] We also learned that the younger mothers who were in the intervention group gained less weight during pregnancy and returned to their pre-pregnancy weight a lot sooner than women in the control condition. This group of stakeholders includes OB/GYNs,

pediatricians, family medicine physicians, midwives, nurses, nutritionists, our laboratory investigators, IT analysts, statisticians, and our funders. [SLIDE 21 and 22] One point of feedback from the prior study was that the research sometimes got in the way of patient care, so we needed to find a more streamlined way of conducting research that didn't distract busy clinicians and teams from delivering care to their patients. [SLIDE 23] We asked the question - could we leverage the resources of the electronic health records and limit our focus of attention to measures that are part of routine clinical care so that we didn't increase any of the burden of clinical research. We were interested in building a de-identified, multi-site EHR database to demonstrate this feasibility. [SLIDE 24] We took the same population definition as the prior study, young women age 12-21, and we were interested in looking at their electronic health records to characterize the care and conditions that they experienced during the preconception, prenatal, postnatal, and early pediatric phases. We identified 85,000 records of eligible women from seven of the eight clinical sites that provided data. When we applied further eligibility restrictions related to height, weight, and BMI, we have a cohort of 55,000 of those women, hence the "big data" component. [SLIDE 24] Again, this is the geographic distribution and the Bronx is heavily represented in our population.

This [table] describes three cohorts [SLIDE 26]: all women, the subgroup of women who become pregnant and those who don't become pregnant, and the babies of the women who are born in this interval, and we will hope to have five years of followup data for these cohorts by the end of 2017. We identified a long list of clinical variables of interest to the practicing clinicians and investigators on the study. [SLIDE 27] We divided those into three separate extractions and transmissions and that was the site staff and the PBRN staff working together, and then we combined that with the expertise of the biostatisticians and the bioinformaticians at Rockefeller, who confirmed that the data were in fact de-identified, underwent cleaning analysis, reporting, and I am here to disseminate to you some of the results of our early cardiometabolic analysis. [SLIDE 28]

This particular slide [SLIDE 29] categorizes women as underweight, normal weight, overweight, or obese, and then shows their systolic and diastolic blood pressure at the first visit during the inception cohort. What is striking here is that each systolic and diastolic blood pressure monotonically increases as you move from the underweight through the obese. The trends are very statistically significant. The numbers suggest that we are just within the range of normotensive, although one could predict that the incidence of hypertension will be faster and sooner among the obese and the overweight. In fact, if we take the same breakdowns of underweight, normal weight, overweight, and obese, [SLIDE 30] and we look at a definition of hypertension

based on three measures from the chart on three different occasions, we find that the prevalence among overweight women was 11%, among obese was 19%, and those figures are two to four times higher than the prevalence of normal weight women and statistically significant. We have a very disturbing picture emerging of the life course and the cardiometabolic course. That picture is virtually identical if you look at the laboratory data from the electronic health record. [SLIDE 31] So, if you look at cholesterol, total cholesterol, triglycerides, blood glucose, and hemoglobin A1c, the trends are all highly-statistically significant and the pattern is virtually identical to what we saw for blood pressure and hypertension.

I will take this a step further and if we look at the incidence of low birth weight, which you will recall was the focus of our intervention study, the incidence of low birth weight is inversely proportional to the weight of the mothers, and in fact, that is a similar finding to a large meta-analysis that included over a million young women, published in 2010. [SLIDE 35] Our two estimates of the relative risk are virtually identical. The confidence intervals are quite similar. It is clear that birth weight is inversely associated with maternal weight. That may be related to a cycle of low birth weights, growth failure, low weight in teens, contributing to low weight in adults, and then repeating that cycle over and over again. If we look at the other end of the spectrum, we also see that all of the large birth weight babies were born to the obese and overweight mothers. So, the cycle here of maternal overweight and obesity contributes to child overweight and obesity, in part due to dietary patterns, feeding environment, etc. But if we take this together, what we have here is a picture of an association that we can demonstrate now from the electronic health records, extracted from routine clinical care. This is not a research setting. These are sites that deliver care, federally-qualified health centers and community hospitals, that really lead us to raising the question of these multigenerational effects and I would speculate that unless we can bend the cardiometabolic curve and do it very soon, if we follow these babies for 20 years or so, we will find the same association in the grandchildren that are going to be born to these babies in the next two decades association - the cardiometabolic associations and cardiometabolic risks. If we could look back to the grandmothers, the mothers of the mothers we are studying, we would probably see the same association. We really have a lifespan model in place here of these multigenerational effects that remind us that we really aren't treating individual patients. We are treating families and communities and generations. This is absolutely essential work for us to try to intervene and intervene using the most effective methods, and soon, in order to avoid the continuation of these disparities.

We are in the relatively early phases of this project's dissemination [SLIDE 36] I want to now reflect back on some of the principles that gave rise to these projects. [SLIDE 37] Both of these studies have monthly team meetings. What has been really critical has been the engagement of the clinicians and the patients in defining the measures and the study parameters and in really understanding what kinds of questions can be structured from these observations. This is an example of the meeting template that we use for every meeting we have by phone or in person and what is really critical is that everybody participates. [SLIDE 39] We ask the clinicians to provide a case report. We have one of our academic colleagues provide an update from generally a single publication from the literature and we focus on opportunities for dissemination (conferences such as this, Town Halls, community health fairs in the neighborhood and everything in between). We have engagement opportunities in grant writing where we break the large group into smaller teams – [SLIDE 40] this is an example of an approach that we followed in both of those studies. Each group is broken up into a team that includes a laboratory investigator, health services researcher, clinician (in the MRSA case), and a patient. Examples of topics include Patient engagement, patient recruitment, incorporating patient needs, defining patient outcomes. Each group has a recorder and text is presented and edited in real-time. We finish those meetings with very complete, usable text for our grant proposals.

I mentioned before that dissemination is a really critical part of this and I hope you'll have the opportunity to look at the CDNetwork.org Rockefeller web page to see some of the programs in adolescent health that we have been presenting as part of this project.

[SLIDE 42] I think one of the key components of this model of embedding mechanistic questions in outcome studies is to ensure that there are variables and hypotheses that are related to each end of the translational spectrum, hence the Partners Along the Translational Research Spectrum. We have several cases under development right now to extend and hopefully replicate this approach.

To summarize, [SLIDE 43] I would say the critical issue here is you have to listen to each stakeholder's needs, priorities, their obligations, and understand their rewards systems. We have to elicit and include the specific priorities for measurement and assessment and make sure that the outcomes are meaningful. We have to align those measures and priorities. We have to be practical and pragmatic about this. Extensive questionnaires and burdensome surveys are just not going to be feasible in this kind of setting. We need to be able to define the outcomes across the full spectrum, starting with the biological, moving into the clinical and

patient-centered, and then into health services measures and public health measures. In this way, we are able to embed these biological measures within well-characterized clinical populations. I think this is what the picture really looks like. As a reminder, [SLIDE 44] when we are alone, we may be harmless, but get them together for a research grant, and watch out! So, thank you for the opportunity to drive and be a passenger. [SLIDE 45]

Declarations

- The presenter has no financial competing interests related to this presentation.All authors have approved the manuscript for submission
- The content of this presentation and transcript has not been published or submitted for publication elsewhere.
- This presentation was organized by the Meharry-Vanderbilt Alliance with support from NCATS and the NIMHD of the National Institutes of Health under award number R13TR001694

Little Data, Big Data
Translational Research Partners Across the Spectrum

Jonathan N. Tobin, PhD
President/CEO
Clinical Directors Network, Inc. (CDN), New York NY
Co-Director, Community Engaged Research
The Rockefeller University Center for Clinical and Translational Science, New York NY
Professor, Department of Epidemiology & Population Health
Albert Einstein College of Medicine/Montefiore Medical Center, Bronx NY

Presented at:
Meharry – Vanderbilt Community Engaged Research Core
Advancing the Science of Community Engaged Research (CEnR)
Association of American Medical Colleges
Washington, DC
September 15, 2017

CDN
CLINICAL·DIRECTORS·NETWORK
www.CDNetwork.org

DISCLOSURE

- I do not have financial or other relationships with the manufacturer(s) of any commercial product(s) or provider(s) of any commercial service(s) discussed in this educational activity.
- In the last 12 months, I have received funding from:
  - NHLBI, NIDDK, NIMH, **NCATS**
  - CDC, **AHRQ**, PCORI
  - NYS Department of Health & NYC Department of Health & Mental Hygiene
  - Chronic Liver Disease Foundation (CLDF)
  - Hepatitis Foundation International (HFI)/Merck
  - Leukemia and Lymphoma Society (LLS)
  - March of Dimes (MoD)
  - **The Rockefeller University Sackler Center for Biomedicine and Nutrition**
  - **New York Academy of Sciences/Sackler Institute for Nutrition Science**
  - Opioid Post-Marketing Requirements Consortium (OPC)/Campbell Alliance (Purdue Pharma, LP; Actavis; Endo Pharmaceuticals; Janssen Pharmaceuticals; Mallinckrodt, LLC; Pfizer; Rhodes Pharmaceuticals, LP; Roxane Laboratories; and Zogenix)

SPECTRUM OF TRANSLATIONAL RESEARCH

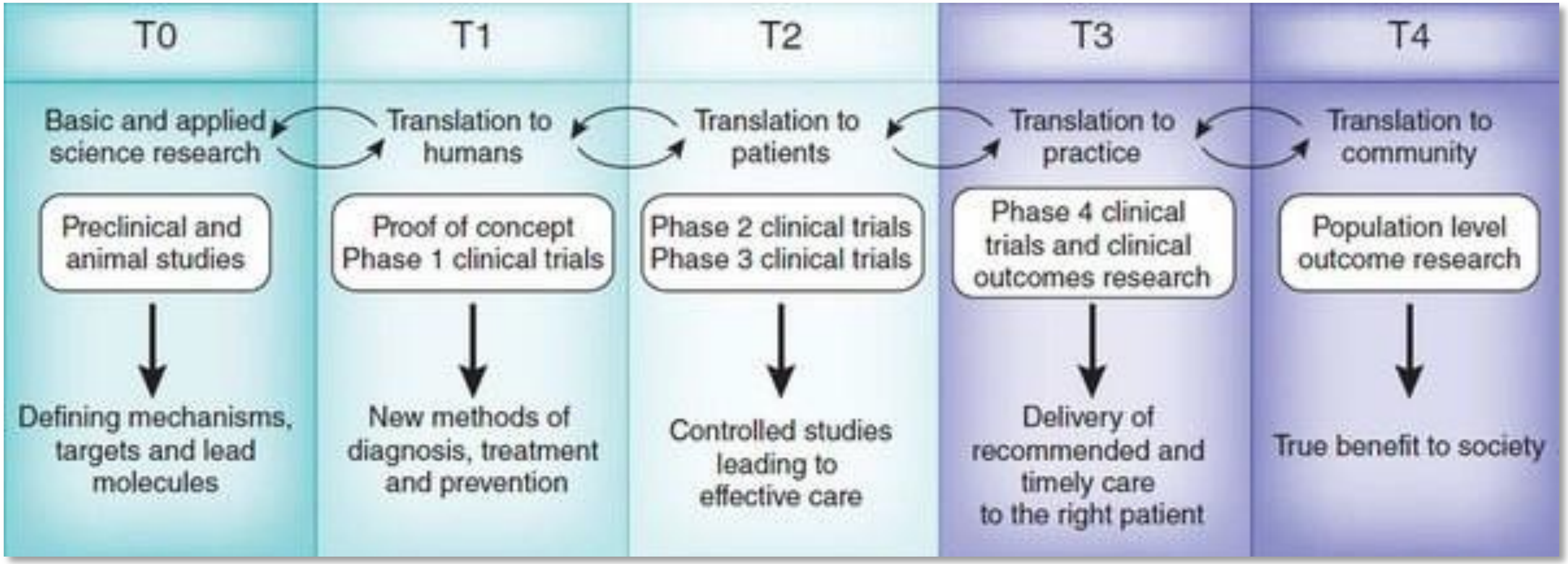

BUILDING COMMUNITY-ACADEMIC TRANSLATIONAL RESEARCH PARTNERSHIPS

CDN/N² = PBRN INFRASTRUCTURE[1]

- Quality Improvement
- Clinical Outcomes
- Comparative Effectiveness Research
- Training Clinician Investigators
- Implementation Science
- Disseminating Methods & Clinical Outcomes Results

+

ROCKEFELLER = CTSA INFRASTRUCTURE

- Laboratory Investigation
- Mechanistic Questions
- Protocol Navigation
- Clinical Scholars
- Bioinformatics/ Phenotyping
- Disseminating Translational Research Methods

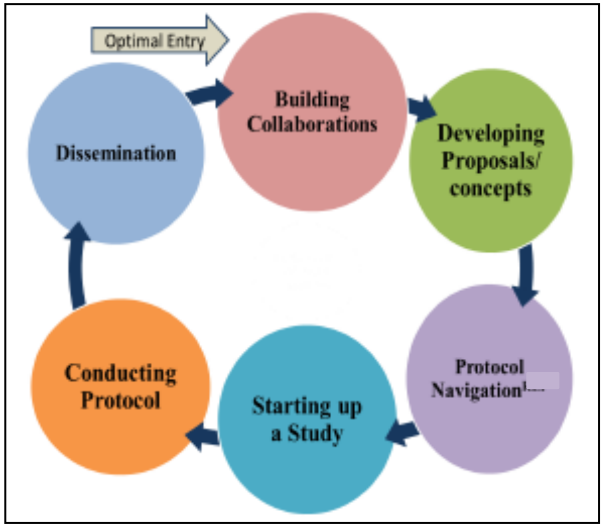

CEnR-Navigation Process (CEnR-Nav)[2]
[Investigators and partners may enter at any stage]

=

CE

CEnR

CER/PCOR

+

Embedded Mechanistic Studies

[1] N²- Building a Network of Safety Net PBRNS, https://www.pbrn.ahrq.gov/sites/default/files/docs/page/N2.pdf
[2] Kost, R.G., Leinberger-Jabari, A., Evering, T.H., Holt, P.R., Neville-Williams, M., Vasquez, K.S., Coller, B.S., Tobin, J.N., "Helping Basic Scientists Engage with Community Partners to Enrich and Accelerate Translational Research." Academic Medicine. 2017;92(3):374.

Little Data, Big Data

CASE STUDY 1

Patient-Centered CER Study of Home-based Interventions to Prevent CA-MRSA Infection Recurrence: CA-MRSA Project 2 (CAMP2)

Patient Centered Outcomes Research Institute (PCORI), Grant # CER-1402-10800

The Rockefeller University Clinical and Translational Science Award Program (CTSA) and an Administrative Supplement and Pilot Project Awards (NIH-NCATS Grant #UL1-TR-000043)

N^2-PBRN: Building a Network of Safety Net PBRNs (AHRQ Grant #1 P30-HS-021667)

TYPES OF STAKEHOLDERS

- Community members
  - Patients
  - Caregiver
  - Family members of patient
- Community Health Workers
- Clinicians
- Academic Stakeholders
- Laboratory Investigators
- Biostatisticians
- Bioinformaticians

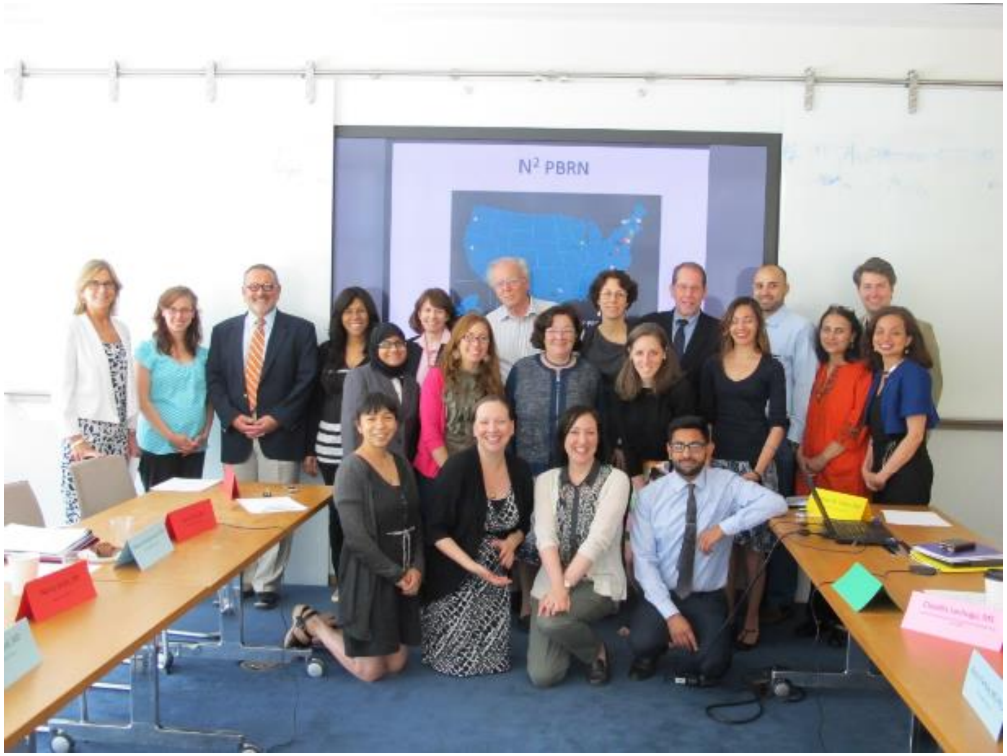

CAMP1 (Cohort) & CAMP2 (CER/PCOR RCT) Stakeholders and Partners

The Rockefeller University

Barry Coller, MD
Rhonda G. Kost, MD
Alexander Tomasz, PhD
Herminia de Lencastre, PhD
Maria Pardos de la Gandara, MD, PhD
Marilyn Chung, BA
Cameron Coffran, MS
Joel Correa da Rosa, PhD
Kimberly Vasquez, MPH
Teresa Evering, MD, MS
Mina Pastagia, MD, MS
Maija Neville-Williams, MPH

CDN

Jonathan N. Tobin, PhD
Chamanara Khalida, MD, MPH
Brianna D'Orazio, BA
Tameir Holder, MPH
Musarrat Rahman, BS
Sisle Heyliger, BA
Anthony Rhabb
Cynthia Mofunanya, MD
Jessica Ramachandran, MD
Uma Siddiqui

Metropolitan Hospital Center

Getaw Worku Hassen, MD, PhD
Jessica Ramachandran, MBBS
*Van Johnson

Coney Island Hospital

Regina Hammock, DO
Slava Gladstein, DO
Rosalee Nguyen, DO, MS
*Ronnett Davis

Community Healthcare Network

Satoko Kanahara, MD
Katrina Adams

Academic Stakeholders

Christopher Frei, PharmD, MSc, FCCP, BCPS
South Texas Ambulatory Research Network/UTHSCSA

Christopher Mason, PhD
Weill Cornell Medical College

Eric Lofgren, PhD
Washington State University College of Veterinary Medicine

Susan Huang, MD, MPH
University of California Irvine

*Patient/Community Stakeholders

NYU Lutheran Family Health Centers

William Pagano, MD, MPH
Paula Clemons, PA
Jason Hyde, MA
Jasbir Singh, MBBS
*Keenan Millan

Open Door Family Medical Center

Daren Wu, MD
Asaf Cohen, MD

Urban Health Plan

Samuel DeLeon, MD
Franco Barsanti, PharmD
Shirish Balachandra, MD
Claude Parola, MD
Tracie Urban, RN
*Brenda Gonzalez

Denny Moe's Superstar Barbershop

*Dennis "Denny Moe" Mitchell

PCORI Project Officers

Anne Trontell, MD, MPH
Jess Robb

Funded by:
Patient Centered Outcomes Research Institute (PCORI, CONTRACT # CER-1402-10800)

The Rockefeller University Center for Clinical and Translational Science (CCTS)
Pilot Grant and Administrative Supplement (NIH-NCATS Grant # 8-UL1-TR000043)

AHRQ Grant # P30 HS 021667

CAMP1 Qualitative Findings:

Convergence of CER/PCOR Interests

Patients: Responses from the RPPS patient focus group indicated that many patients participated in the CAMP study in order to contribute to knowledge about CA-MRSA transmission and recurrence. Outcomes that patients were most concerned about include: **recurrence,** pain and inability to work. Among Research Town Hall participants, 78% ranked "**Reducing the spread of MRSA in your household**" as being "most important" on a scale of 1-5.

Clinicians: "[It is assumed that] colonization is ongoing, because we've had **patients return with recurrent infections.** …If you just use systemic antibiotics, the nasal colonization persists. Another question to consider is if the source is in the house. We can take all measures to decolonize the person but if the infection is still in the house (pet, towel, sheets, etc), then it's a huge factor." – Dr. Balachandra

Laboratory Investigators: "Does the MRSA **recurrent phenotype** reflect single or multiple genotypes?

Clinical Investigators: **31%** of MRSA+ wounds and **28%** of MSSA+ wounds are **recurrent**

THIS CONVERGENCE OF INTERESTS LED US TO FOCUS ON
LABORATORY & CLINICAL CORRELATES
OF INFECTION RECURRENCE
AND TO PRIORITIZE THE STUDY OF
PREVENTION OF INFECTION RECURRENCE

CAMP1 Clinical Outcomes *(n=153)*

S. aureus Recurrence Cases (n=91)

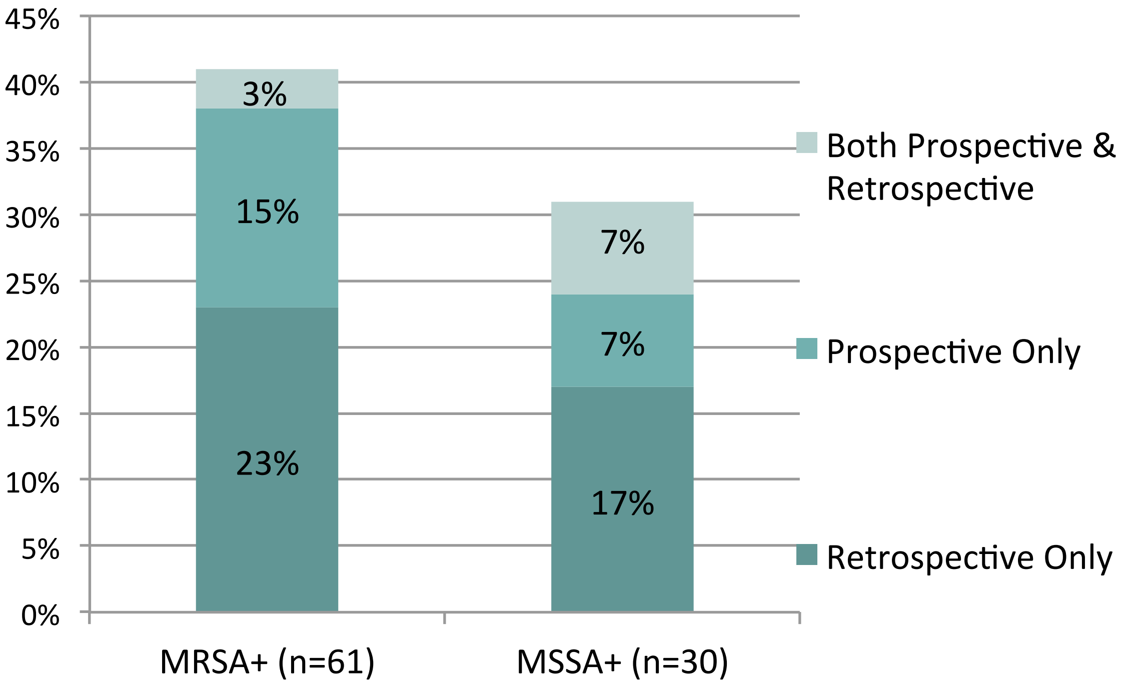

Retrospective Recurrence: History of SSTIs/MRSA Before Enrollment

- **T1:** Recurrent infection
- **T2:** Received treatment of this lesion previously
- **T3:** Previously documented MRSA infection or colonization

Prospective Recurrence: Subsequent Incident of SSTIs/ MRSA After Enrollment

- **T3:** Re-occurring complaint of SSTI at more recent primary care visit

OBJECTIVES

To evaluate the comparative effectiveness of a CHW/Promotora-delivered home intervention (Experimental Group) as compared to Usual Care (Control Group) on the primary patient-centered and clinical outcome (SSTI recurrence rates) and secondary patient-centered outcomes (pain, depression, quality of life, care satisfaction) and public health outcomes (household transmission) using a two-arm randomized controlled trial (RCT).

CLINICAL-DIRECTORS-NETWORK
www.CDNetwork.org

CAMP2 Participating Sites

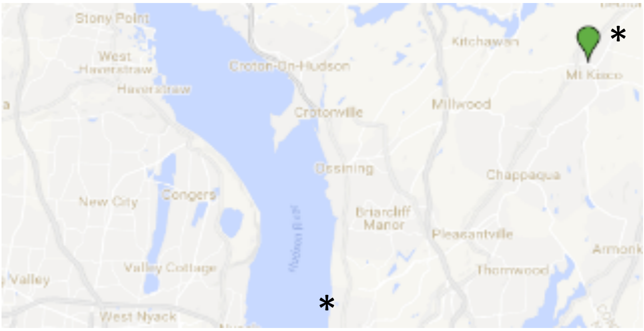

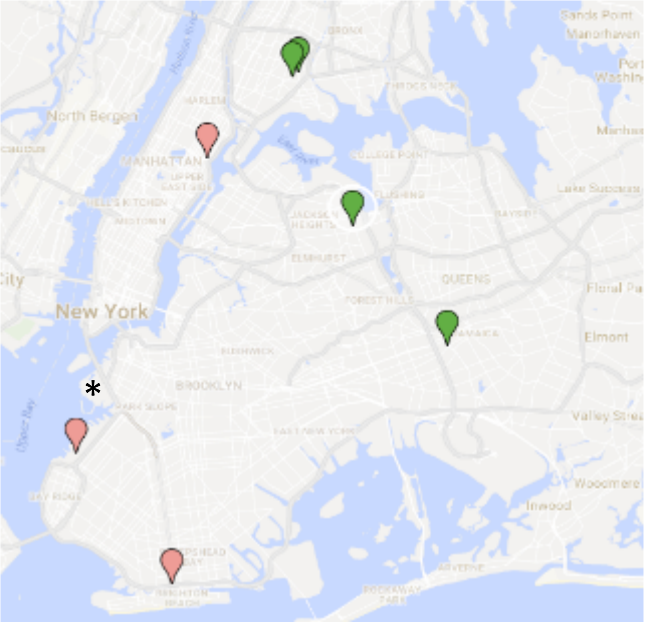

Community Health Centers
Community Hospitals
* Participated in Previous MRSA Studies

Participating Sites	Clinician Stakeholders
Open Door Family Medical Center<br>*Mt. Kisco, NY*	Daren Wu, MD<br>Asaf Cohen, MD
Urban Health Plan<br>*Bronx, NY*<br>*Corona, NY*	Claude Parola, MD<br>Tracie Urban, RN, BSN
Community HealthCare Network<br>*Bronx, NY*<br>*Jamaica, NY*	Satoko Kanahara, MD
Metropolitan Hospital<br>*New York, NY*	Getaw Worku Hassen, MD, PhD
Lutheran Medical Center<br>*Brooklyn, NY*	Barry Kohn, MD<br>Paula Clemons, PA
Coney Island Hospital<br>*Brooklyn, NY*	Regina Hammock, DO<br>Slava Gladstein, DO<br>Jessica Padron, PA<br>Glenn Donovan, DPM<br>Mark Trezia, DPM<br>Rosalie Nguyen, DO, MS

CAMP2 Research Design

CDC Guidelines Concordant Care:
Incision & Drainage
<u>+</u> Oral Antibiotics

Assessment of Household Environmental Contamination & Household Members Colonization

Patient & Household Members
- Decolonization

Home Environment
- Environmental Decontamination

(after S. Huang, 2014)
1) Nasal Mupirocin
2) Chlorhexidine Baths on Skin
3) Chlorine Bleach Cleaning of Household Surfaces

Usual Care (CDC Guidelines) Control Intervention (n=139)	Usual Care (CDC Guidelines) + Experimental Intervention (n=139)
+	+
+	+
-	+

CAMP2 Home Visit Assessment: Household Surface Sampling

Collected at Baseline and 3 Months Post Intervention from:

- Index patients (n=278)
- Consenting household members
- Home Environment Surfaces

Index Patients and Household Members
(n=3 per participant)
Baseline and 3-Months

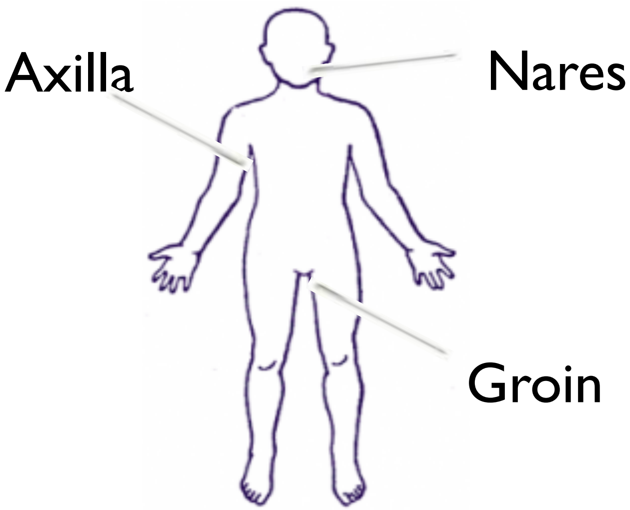

Environment
(n=13 surfaces per household)

Surface to Swab	
Front doorknob	Kitchen floor
TV remote	Bathroom sink handle
Telephone	Hair brush
Kitchen light switch	Toilet seat
Kitchen countertop	Bedroom floor
Refrigerator door handle	Favorite child's toy (non-plush)
Kitchen sink handle	

CAMP2 Household Specimens Meta-Genomics

CDN CLINICAL•DIRECTORS•NETWORK
www.CDNetwork.org

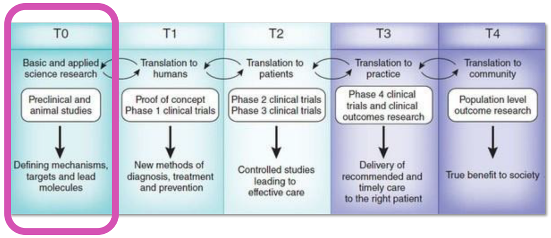

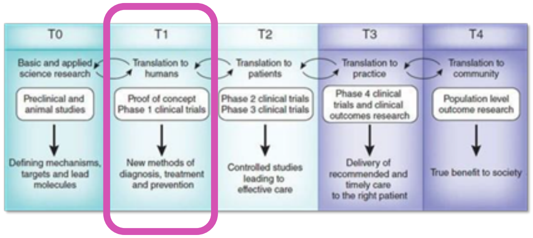

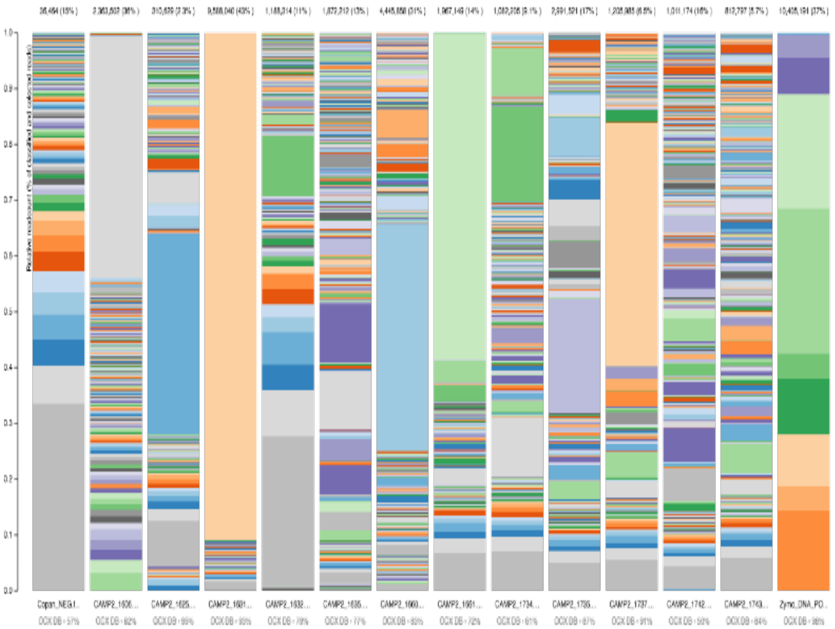

Molecular profile of USA 300 MRSA wound isolates

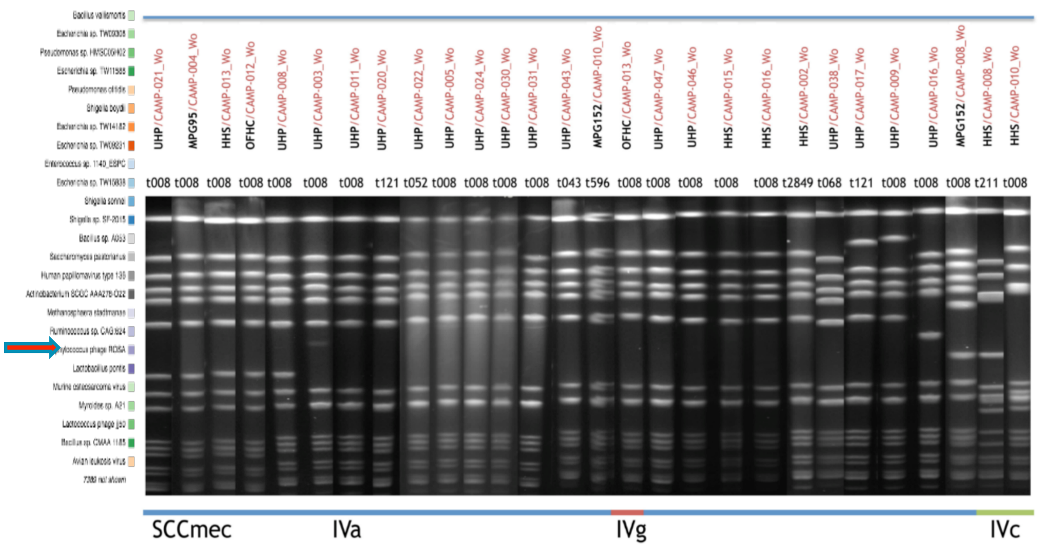

All MRSA wound isolates belonging to the USA 300 clone (ST 8) were:
- *pvl* +
- ACME type I

Weill Cornell Medicine

One Codex: A Sensitive and Accurate Data Platform for Genomic Microbial Identification, Samuel S Minot, Niklas Krumm, Nicholas B Greenfield
bioRxiv 027607; doi: https://doi.org/10.1101/027607

JCM

Molecular Types of Methicillin-Resistant *Staphylococcus aureus* and Methicillin-Sensitive *S. aureus* Strains Causing Skin and Soft Tissue Infections and Nasal Colonization, Identified in Community Health Centers in New York City

Shirish Balachandra, MD (CDN/Urban Health Plan FQHC)
Maria Pardos, MD PhD (Rockefeller)
Case Study of MRSA Infection Recurrence

MICROBIAL DRUG RESISTANCE
Volume 21, Number 2, 2015
© Mary Ann Liebert, Inc.
DOI: 10.1089/mdr.2014.0283

(T3 Clinician Investigator Expertise/Interest)

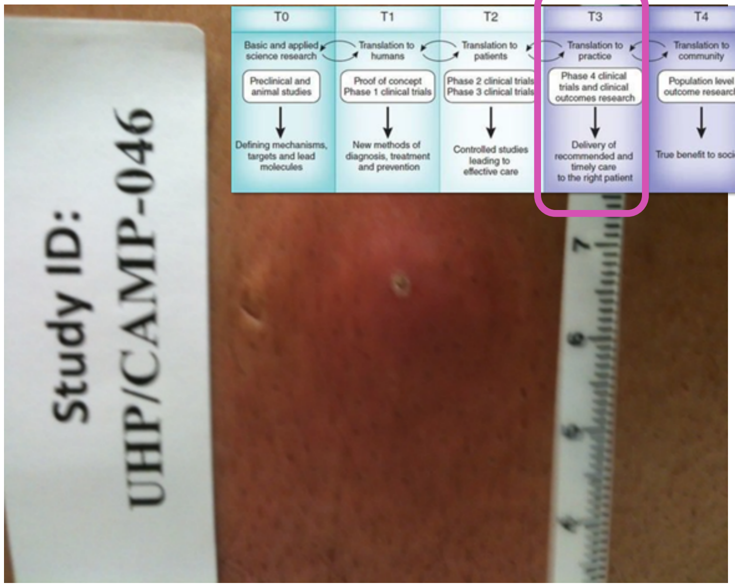

Recurrent Furunculosis Caused by a Community-Acquired *Staphylococcus aureus* Strain Belonging to the USA300 Clone

Shirish Balachandra,[1,*] Maria Pardos de la Gandara,[2,*] Scott Salvato,[1] Tracie Urban,[1] Claude Parola,[1] Chamanara Khalida,[3] Rhonda G. Kost,[4] Teresa H. Evering,[4] Mina Pastagia,[4] Brianna M. D'Orazio,[3] Alexander Tomasz,[2] Herminia de Lencastre,[2,5] and Jonathan N. Tobin[3,4]

Background: A 24-year-old female with recurrent skin and soft tissue infections (SSTI) was enrolled as part of a multicenter observational cohort study conducted by a practice-based research network (PBRN) on community-acquired methicillin-resistant *Staphylococcus aureus* (CA-MRSA). ***Methods:*** Strains were characterized by pulsed-field gel electrophoresis (PFGE), *spa* typing, and multilocus sequence typing. MRSA strains were analyzed for SCC*mec* type and the presence of the Panton-Valentine leukocidin (PVL) and arginine catabolic mobile element (ACME) using PCR. ***Results:*** In the first episode, *S. aureus* was recovered from the wound and inguinal folds; in the second, *S. aureus* was recovered from a lower abdomen furuncle, inguinal folds, and patellar fold. Molecular typing identified CA-MRSA clone USA300 in all samples as *spa*-type t008, ST8, SCC*mec*IVa, and a typical PFGE pattern. The strain carried virulence genes *pvl* and ACME type I. Five SSTI episodes were documented despite successful resolution by antibiotic treatment, with and without incision and drainage. ***Conclusions:*** The source of the USA300 strain remains unknown. The isolate may represent a persistent strain capable of surviving extensive antibiotic pressure or a persistent environmental reservoir may be the source, possibly in the patient's household, from which bacteria were repeatedly introduced into the skin flora with subsequent infections.

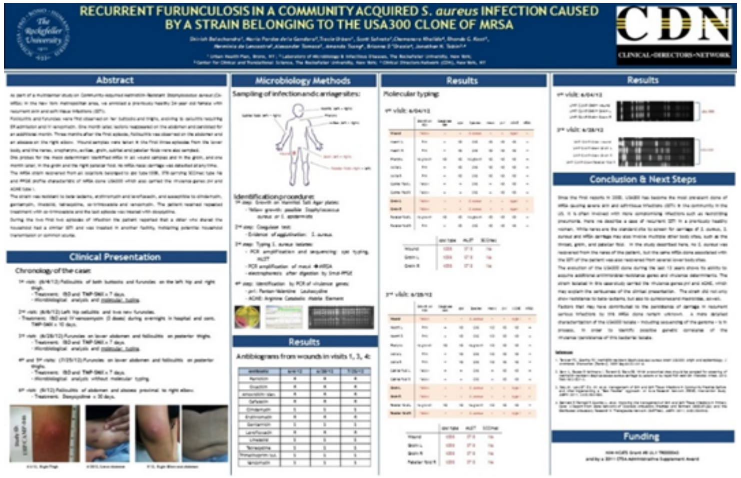

(T3/T4 Clinician & Public Health Investigators Expertise/Interest)

CAMP2 Baseline Results *(4/17/17)*

Surveillance Site	Patient Colonization (n=135)	Household Member Colonization (n=40)
Nares	51.9%	10.0%
Axilla	17.8%	17.5%
Groin	34.1%	25.0%
0 Colonized sites	33.3%	67.5%
1 Colonized site	35.6%]	15.0%]
2+ Colonized sites	29.7%] **65%**	17.5%] **33%**

Household Surface Site	Household Surface Contamination (n=52)
**Kitchen floor**	**19.2%**
**Toilet seat**	**23.1%**
**Bedroom floor**	**21.2%**
Refrigerator handle	13.5%
TV remote	11.5%
Telephone	11.5%
Bathroom sink handle	11.5%
Kitchen countertop	9.6%
Kitchen light switch	5.8%
Front doorknob	5.8%
Child's toy	3.8%
Hairbrush	3.8%
Kitchen sink handle	1.9%
No Contamination (0 surfaces)	40.4%
Moderate Contamination (1-3)	48.1%]
High Contamination (> 4)	11.5%] **60%**

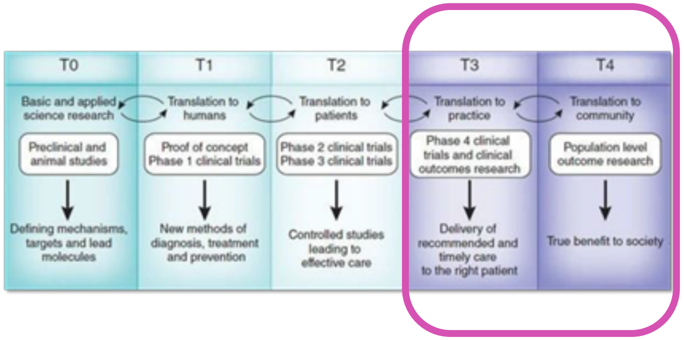

(T4 Clinician Investigator Interest)

Community-Engaged Research Pilot Project:

Expanding the Study of SSTIs/CA-MRSA to Barbershops and Beauty Salons in NYC

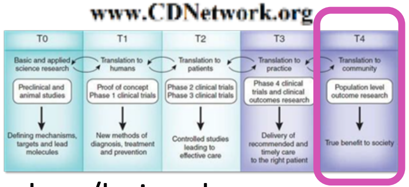

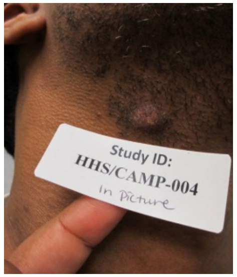

- The CA-MRSA Project (CAMP1) extended its learning collaborative to the barbershop/hair salon settings to increase awareness and prevention of CA-MRSA and other infections.
  - Previous studies have shown the receptiveness of male barbershop **owners, employees and patrons** to learning more about disease prevention and **occupational safety and health.**
  - **SSTIs/lesions are often observed on face, scalp, head, neck, arms, hands.**
  - **About 20% of CAMP participants' lesions presented in these locations.**
- Barbers and their clients were highly receptive to inquiries and information about MRSA.
- Barbers welcome an in-depth public health education in the barbershop setting.

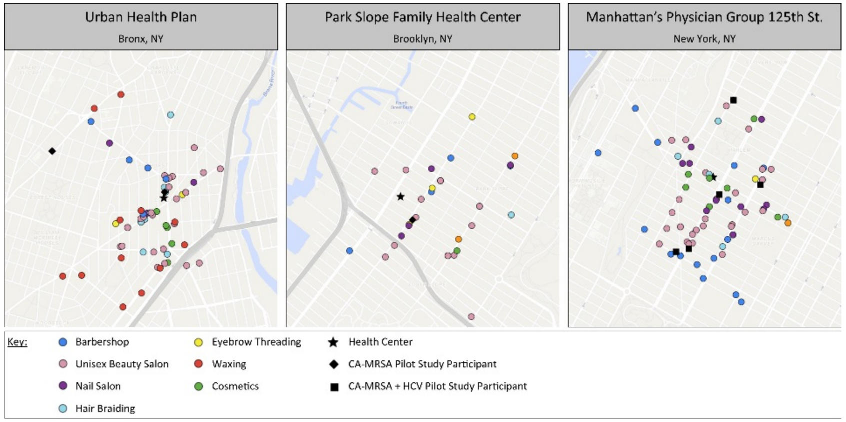

Pre-Post Test: Percent Correct T1-T2

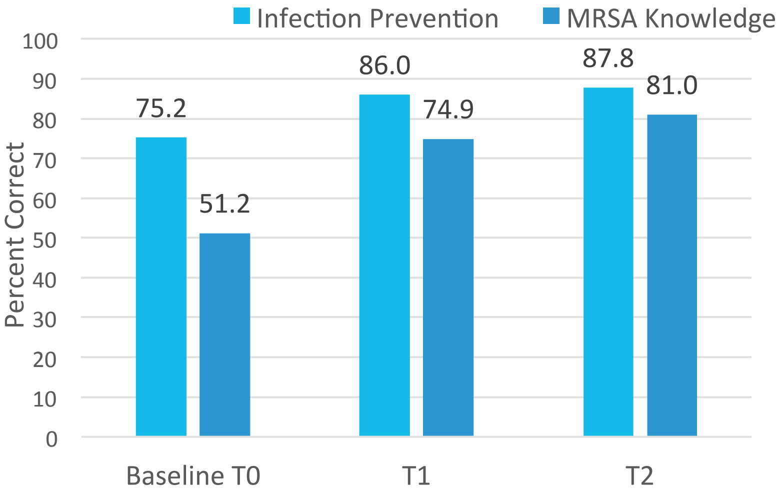

MRSA Infection Prevention
Baseline to T1: p=0.0003
Baseline to T2: p=0.0001

MRSA Knowledge
Baseline to T1: p<0.0001
Baseline to T2: p<0.0001

Rockefeller Webcasts Serire

cdnetwork.org

Search

ABOUT US | RESEARCH | EDUCATION | DISSEMINATION | LIBRARY | PARTNERSHIPS | CONTACT US

EDUCATION

CDN is an AHRQ designated Center of Excellence for Primary Care Practice-Based Research and Learning (2012).

LEARN MORE

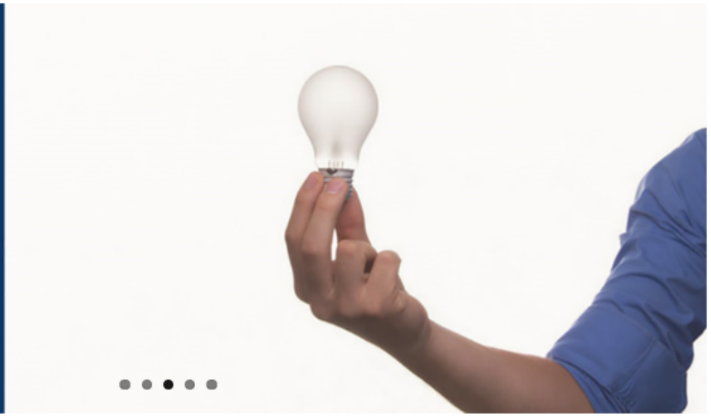

Clinical Directors Network, Inc. (CDN) is a not-for-profit clinician membership organization, practice-based research network (PBRN), and clinician training organization, founded to provide peer-initiated activities for clinicians practicing in low income, minority, and other underserved communities. CDN's overall goal is to translate clinical research into clinical practice for the enhancement of health equity and improvement of public health. MORE

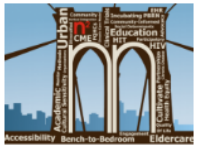

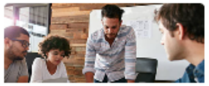

Research

CDN was designated a "Best Practice" Clinical Research Network by the NIH (2006).

We accelerate research translation. CDN has over 25 years of experience developing, conducting, implementing and evaluating practice-based research with Community Health Centers and other safety-net practices. MORE

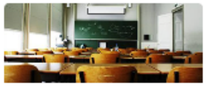

Education

CDN is an AHRQ designated Center of Excellence for Primary Care Practice-Based Research and Learning (2012).

We provide peer support through training and education that integrates online and on-site didactic and experiential learning. Collaborate with us to meet your training needs. MORE

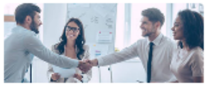

Partnership

CDN has an extensive network of multidisciplinary researchers, clinicians, clinical leaders and policy-makers.

We conduct research and educational activities in partnership with government, academic, not-for-profit, and for profit organizations. Our national network represents an enormous resource for change. MORE

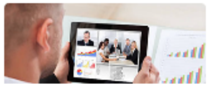

Dissemination

CDN has extensive experience disseminating research and training programs to our extensive network of multidisciplinary researchers, clinicians, clinical leaders and policy-makers.

We provide dissemination services through webcasts for public health and clinical research projects. MORE

Little Data, **Big Data**

CASE STUDY 2

Obesity, Cardiometabolic Risk and Adolescent Pregnancy:

Building a De-Identified Electronic Clinical Database to Examine the Biological and Social Determinants of Nutritional Status, Pregnancy and Birth Outcomes

FUNDED BY:

The Sackler Center for Biomedicine and Nutrition (SCBN) Research at The Rockefeller University; The Sackler Institute for Nutrition Science at The New York Academy of Sciences; (3) N^2: Building a Network of Safety-Net PBRNs (AHRQ 1-P30-HS-021667); (4)The National Center for Advancing Translational Sciences/ The Rockefeller University Center for Clinical and Translational Science (NIH-NCATS Grant #UL1-TR-000043)

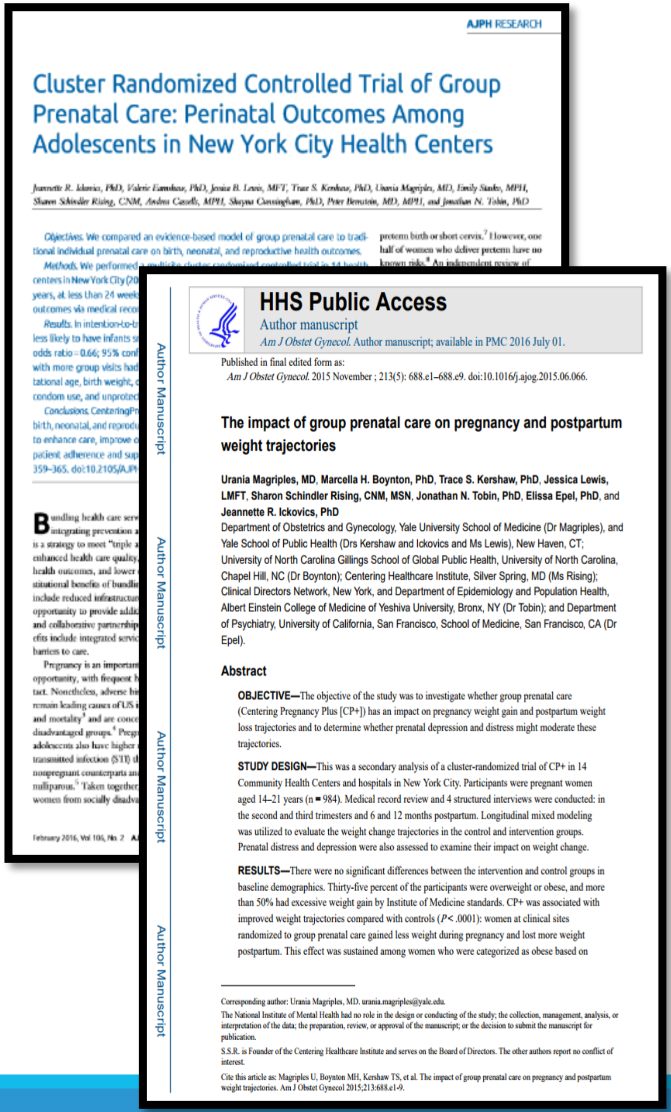
AJPH RESEARCH

Cluster Randomized Controlled Trial of Group Prenatal Care: Perinatal Outcomes Among Adolescents in New York City Health Centers

Jeannette R. Ickovics, PhD, Valerie Earnshaw, PhD, Jessica B. Lewis, MFT, Trace S. Kershaw, PhD, Urania Magriples, MD, Emily Stasko, MPH, Sharon Schindler Rising, CNM, Andrea Cassells, MPH, Shayna Cunningham, PhD, Peter Bernstein, MD, MPH, and Jonathan N. Tobin, PhD

HHS Public Access
Author manuscript
Am J Obstet Gynecol. Author manuscript; available in PMC 2016 July 01.

Published in final edited form as:
Am J Obstet Gynecol. 2015 November ; 213(5): 688.e1–688.e9. doi:10.1016/j.ajog.2015.06.066.

The impact of group prenatal care on pregnancy and postpartum weight trajectories

Urania Magriples, MD, Marcella H. Boynton, PhD, Trace S. Kershaw, PhD, Jessica Lewis, LMFT, Sharon Schindler Rising, CNM, MSN, Jonathan N. Tobin, PhD, Elissa Epel, PhD, and Jeannette R. Ickovics, PhD
Department of Obstetrics and Gynecology, Yale University School of Medicine (Dr Magriples), and Yale School of Public Health (Drs Kershaw and Ickovics and Ms Lewis), New Haven, CT; University of North Carolina Gillings School of Global Public Health, University of North Carolina, Chapel Hill, NC (Dr Boynton); Centering Healthcare Institute, Silver Spring, MD (Ms Rising); Clinical Directors Network, New York, and Department of Epidemiology and Population Health, Albert Einstein College of Medicine of Yeshiva University, Bronx, NY (Dr Tobin); and Department of Psychiatry, University of California, San Francisco, School of Medicine, San Francisco, CA (Dr Epel).

Abstract

OBJECTIVE—The objective of the study was to investigate whether group prenatal care (Centering Pregnancy Plus [CP+]) has an impact on pregnancy weight gain and postpartum weight loss trajectories and to determine whether prenatal depression and distress might moderate these trajectories.

STUDY DESIGN—This was a secondary analysis of a cluster-randomized trial of CP+ in 14 Community Health Centers and hospitals in New York City. Participants were pregnant women aged 14–21 years (n = 984). Medical record review and 4 structured interviews were conducted: in the second and third trimesters and 6 and 12 months postpartum. Longitudinal mixed modeling was utilized to evaluate the weight change trajectories in the control and intervention groups. Prenatal distress and depression were also assessed to examine their impact on weight change.

RESULTS—There were no significant differences between the intervention and control groups in baseline demographics. Thirty-five percent of the participants were overweight or obese, and more than 50% had excessive weight gain by Institute of Medicine standards. CP+ was associated with improved weight trajectories compared with controls (*P* < .0001): women at clinical sites randomized to group prenatal care gained less weight during pregnancy and lost more weight postpartum. This effect was sustained among women who were categorized as obese based on

Corresponding author: Urania Magriples, MD. urania.magriples@yale.edu.
The National Institute of Mental Health had no role in the design or conducting of the study; the collection, management, analysis, or interpretation of the data; the preparation, review, or approval of the manuscript; or the decision to submit the manuscript for publication.
S.S.R. is Founder of the Centering Healthcare Institute and serves on the Board of Directors. The other authors report no conflict of interest.
Cite this article as: Magriples U, Boynton MH, Kershaw TS, et al. The impact of group prenatal care on pregnancy and postpartum weight trajectories. Am J Obstet Gynecol 2015;213:688.e1-9.

COMPARISONS

Usual Care Individual prenatal care

Experimental Centering Pregnancy Plus (Group prenatal care)

Outcome	AOR[a] (95% CI)
Birth or neonatal	
Preterm birth at < 37 wk	0.76 (0.69, 0.84)
Low birth weight, < 2500 g	0.81 (0.73, 0.89)
Small for gestational age	0.91 (0.85, 0.99)

WEIGHT CHANGE OVER TIME BY INTERVENTION CONDITION AND OBESE GROUP STATUS

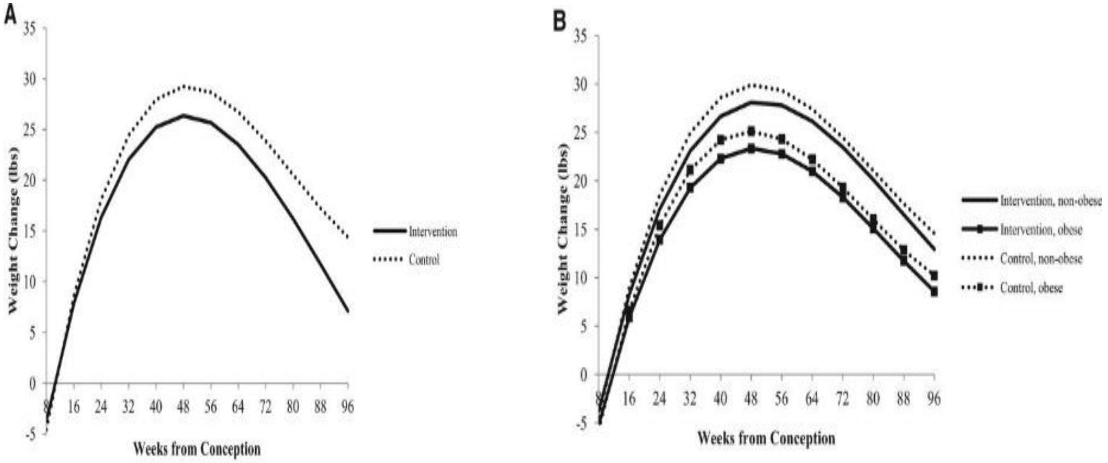

A, Weight change over time as predicted by intervention condition. **B**, Weight change over time as predicted by intervention condition × obese group status.

COLLABORATORS

The Rockefeller University
- **Jan L. Breslow, MD***
- Peter R. Holt, MD
- Caroline S. Jiang, MS
- Bruce S. McEwen, PhD
- Rhonda G. Kost, MD
- Kimberly S. Vasquez, MPH
- Joel Correa da Rosa, PhD
- Cameron Coffran, MS
- Donna Brassil, MA, RN, CCRC

The Sackler Institute for Nutrition Science/The New York Academy of Sciences
- **Mireille McLean, MA MPH***
- Julie Shlisky, PhD
- Gilles Bergeron, PhD
- Megan Bourassa, PhD

Clinical Directors Network, Inc. (CDN)
- **Jonathan N. Tobin, PhD****
- Amanda Cheng, MPH
- Dena Moftah, BA
- Julie Wilcox, MFA

Albert Einstein College of Medicine/ Montefiore Medical Center
- Peter S. Bernstein, MD, MPH
- Rebecca Mahn, MD/MS candidate
- Siobhan Dolan, MD
- Stephanie Morgan, MS

Clinician Advisory Committee
- Tyler Evans, MD
- Elizabeth Dubois, MSN, FNP-BC, AAHIVS
- Mayer Sagy, MD
- William Pagano, MD, MPH
- Barry Kohn, MD
- Rabih Nemr, MD
- Abbe Kirsch, CNM, MSN, MPH
- Daryl Wieland, MD, MSMI
- Peter Bernstein, MD, MPH
- Siobhan Dolan, MD
- Isaac Dapkins, MD, FAAP

***Project Officers** ****Principal Investigator**

The Rockefeller University

THE SACKLER INSTITUTE *for* NUTRITION SCIENCE

a program of The New York Academy of Sciences

CDN CLINICAL·DIRECTORS·NETWORK www.CDNetwork.org

TYPES OF STAKEHOLDERS

- Physicians
  - Pediatrics
  - OBGYN
  - Family Medicine
  - Bariatric Surgery
- Midwives
- Nurses

- Nutritionists
- Researchers
- IT Analysts
- Biostatisticians
- Bioinformaticians
- Basic Scientists
- Funders
- Scientific Publishers

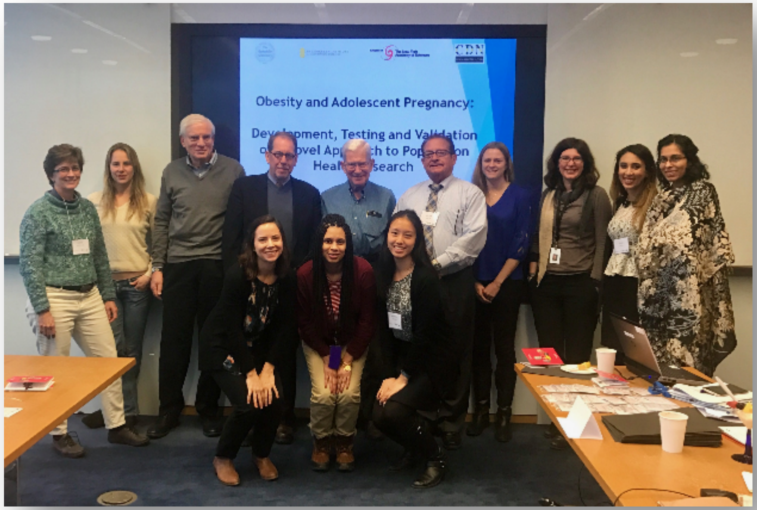

OBJECTIVES

This community-academic partnership involves the creation of a **multisite de-identified Electronic Health Records (EHR) database** that will demonstrate the feasibility of using available measures conducted as part of routine clinical care to explore associations and identify targets for future interventions that address adolescent nutritional and pregnancy outcomes.

This “Big Data” EHR-based study addresses the disproportionate health burdens experienced by overweight and obese adolescents and their infants up to the age of 24 months.

METHODS/STUDY POPULATION

Partnering New York City Community Health Centers (n=4) and Hospitals (n=4) are working with The Rockefeller University, the Sackler Institute for Nutrition Science and the Clinical Directors Network (CDN) to build a de-identified database extracted from EHR data from all **female adolescents, aged 12-21 years and their offspring through 24 months**, who received their primary care at these eight (8) clinical sites between 2011-2015.

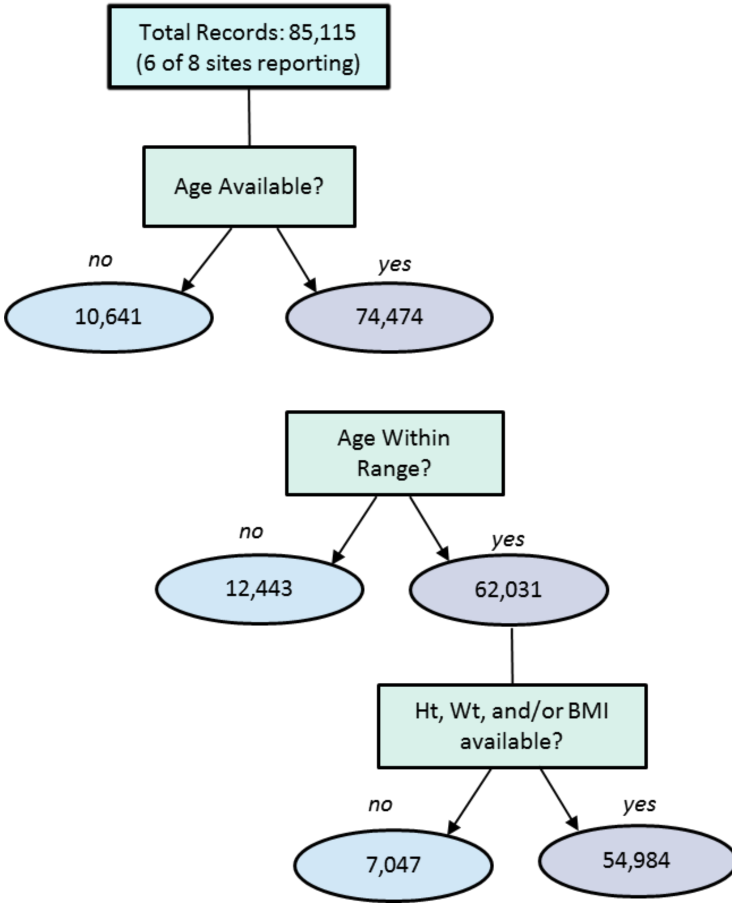

This time period covers all care visits:

- **Preconception**
- **Prenatal**
- **Postnatal**
- **Pediatric**

COMMUNITY HEALTH CENTERS/COMMUNITY HOSPITALS

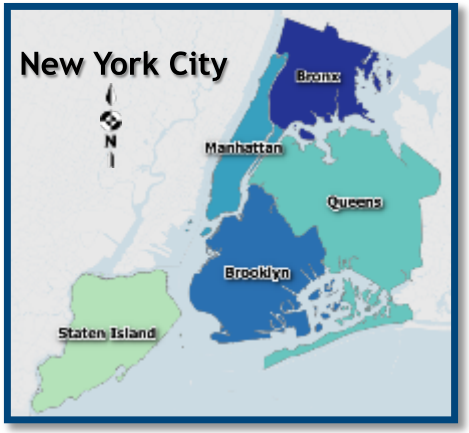

Borough (n=84,714)*	Percent
Bronx	68.5%
Brooklyn	15.4%
Manhattan	5.0%
Queens	5.3%
Staten Island	0.4%
Westchester	1.6%
Yonkers	1.1%
Other	1.2%

*Reflects patients who provided a zip code

Community Health Center/Hospital	Location(s)	Co-Investigators	IRB
Community Healthcare Network	Bronx, NY<br>Brooklyn, NY<br>New York, NY<br>Queens, NY	Tyler Evans, MD<br>Elizabeth Dubois, MSN, FNP-BC, AAHIVS	Clinical Directors Network (CDN)
Morrisania Diagnostic and Treatment Center	Bronx, NY	Mayer Sagy, MD	BRANY
Gouverneur Health	New York, NY	Mayer Sagy, MD	BRANY
NYU Family Health Centers	Brooklyn, NY	William Pagano, MD, MPH<br>Barry Kohn, MD<br>Rabih Nemr, MD	BRANY
Bronx Lebanon Hospital Center	Bronx, NY	Abbe Kirsch, CNM, MSN, MPH	BRANY
Jacobi Medical Center	Bronx, NY	Daryl Wieland, MD, MSMI	BRANY
North Central Bronx Hospital	Bronx, NY	Daryl Wieland, MD, MSMI	BRANY
Montefiore Medical Center	Bronx, NY	Peter Bernstein, MD, MPH<br>Siobhan Dolan, MD	BRANY

COHORTS AND TIMELINE

- Start: 1/1/2011
- End: 12/31/2012
- Last Birth: 8/31/2013
- 12 months: 8/31/2014
- 24 months: 8/31/2015

Time	2011	2012	2013	2014	2015	2016
Adolescent	Adolescents: Females, Ages 12-21		Adolescents: Females, Ages 12-21 (Continued follow-up)			Data Analysis
Birth at the end of enrollment period	N/A		Adolescents' Offspring: Postpartum Follow-up for 24 months			Data Analysis
Adolescent	Adolescents: Females, Ages 12-21		Adolescents: Females, Ages 12-21 (Continued follow-up)			Data Analysis
Birth at the beginning of enrollment period	Adolescents' Offspring: Postpartum Follow-up for 24 months		Adolescents' Offspring: Postpartum Follow-up > 24 months			Data Analysis
Never pregnant	Adolescents: Females, Ages 12-21					Data Analysis
Time	2011	2012	2013	2014	2015	2016

Key: Adolescents Offspring Data Analysis *Age during enrollment (1/1/11-12/31/12)

The Rockefeller University
THE SACKLER INSTITUTE for NUTRITION SCIENCE
a program of The New York Academy of Sciences
CDN CLINICAL•DIRECTORS•NETWORK www.CDNetwork.org

SACKLER – FROM BIG PICTURE TO IMPLEMENTATION

List of 100+ Variables

Type	Variable	Categories (if applicable)
Demographics	Age (years)	
	Gender	Female
	Race	American Indian/Alaska Native, Asian, Native Hawaiian or Other Pacific Islander, Black or African American, White
	Ethnicity	Hispanic or Latino, Not Hispanic or Latino
	Zip Code of Residency *	
Vitals	Height (inches)	
	Weight (lbs)	
	BMI	
	Systolic Blood Pressure (mm Hg)	
	Diastolic Blood Pressure (mm Hg)	
Laboratory	Glucose (mg/dL)	
	Total Cholesterol (mg/dL)	
	Triglycerides (mg/DL)	
	HbA1c (%, mmol/mol)	
Psychosocial: Cognitive	Positive PHQ2	[illegible]
Psychosocial: Healthcare Utilization	OBGYN History	[illegible]
	Gravidity [illegible]	[illegible]
	Parity [illegible]	[illegible]
[illegible]	[illegible]	1st, 2nd, 3rd
[illegible]	[illegible]	Yes (dates on following screening A1C, Fasting Plasma Glucose or Oral Glucose Tolerance Test), No
[illegible]	[illegible] Weight Gain	Weight/Time (e.g. kg/week), Dates**
[illegible]	[illegible]	Yes (Add Date), No**
[illegible]	[illegible] Delivery Modality	Type (e.g. Vaginal, Caesarian Section), [illegible], Date**
[illegible]	Medical Visits (counts)	
Offspring: Ne[illegible]	Live Birth Counts, Dates, Week of Delivery**	
	Non-live Birth (e.g. Stillbirths) Counts, Weeks of Delivery**	
	Birth Weight	
	Gender	Female, Male
	Gestational Age at Delivery (Age and Weeks)	
	NICU Admission	Yes (Dates and Length of Stays), No**
Offspring: Pediatric Follow-up	Weight and Date**	
	Breastfeeding	Immediate (e.g. Within First Two Hours of Delivery), 6 Months, 1 Year
	Immunization Record Completed by 24 Months of Age	Yes, No (Reason Not Completed)

Primary Investigator and Clinicians

3 separate extractions & transmissions

1 2 3

Site IT and PBRN Staff

Combining data from multiple transmissions

PBRN Staff Biostatistician/ Bioinformatician

Confirmation of De-Identification

Data Cleaning

Data Analysis

Reporting

Dissemination

PBRN Staff Biostatistician/ Bioinformatician

CARDIOMETABOLIC ANALYSIS

Blood Pressure
(Sites A, B, C, D, E; n = 45,541)

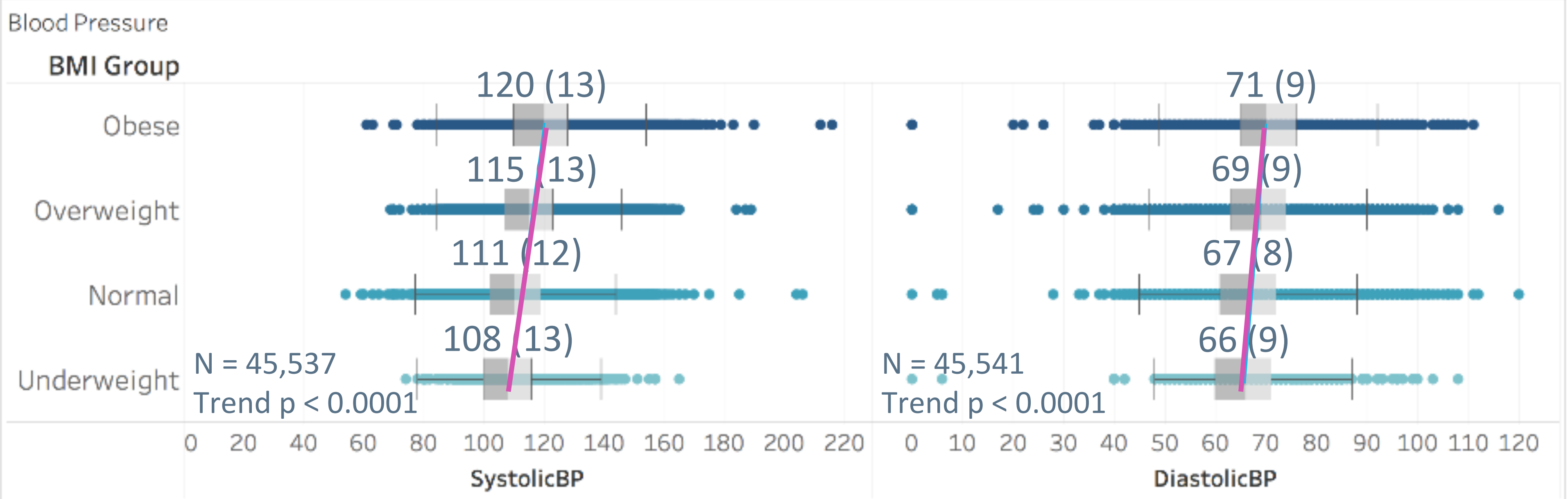

* Mean (SD)

THE SACKLER INSTITUTE *for* NUTRITION SCIENCE

a program of The New York Academy of Sciences

CDN CLINICAL•DIRECTORS•NETWORK www.CDNetwork.org

ABNORMAL BLOOD PRESSURE BY BMI PERCENTILE

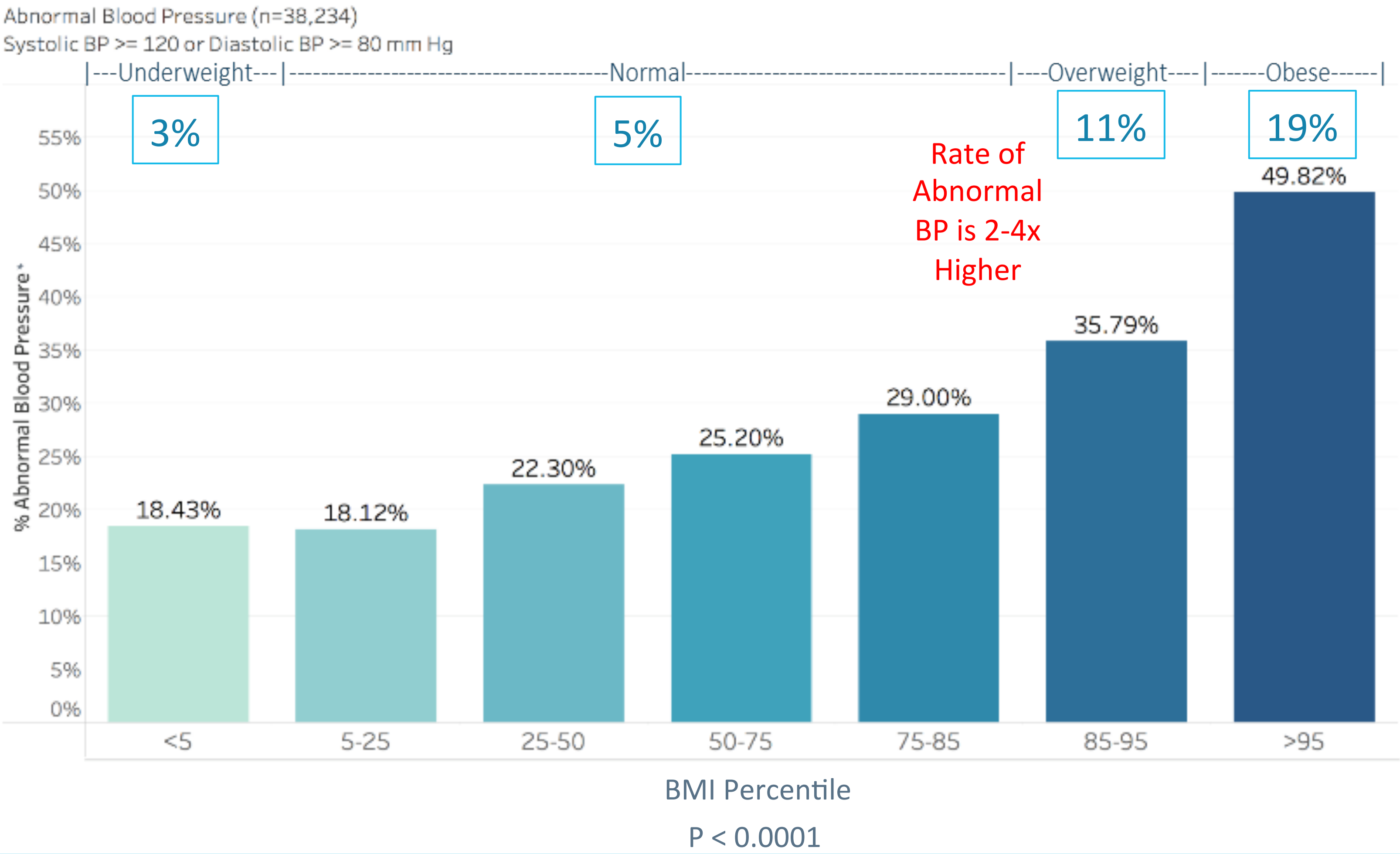

*3 measurements with systolic BP ≥ 120 or diastolic BP ≥ 80 mmHg per Cheung et al. 2017

Cardiometabolic Measures (Sites A, B, C) (n = 3,569 – 5,576)

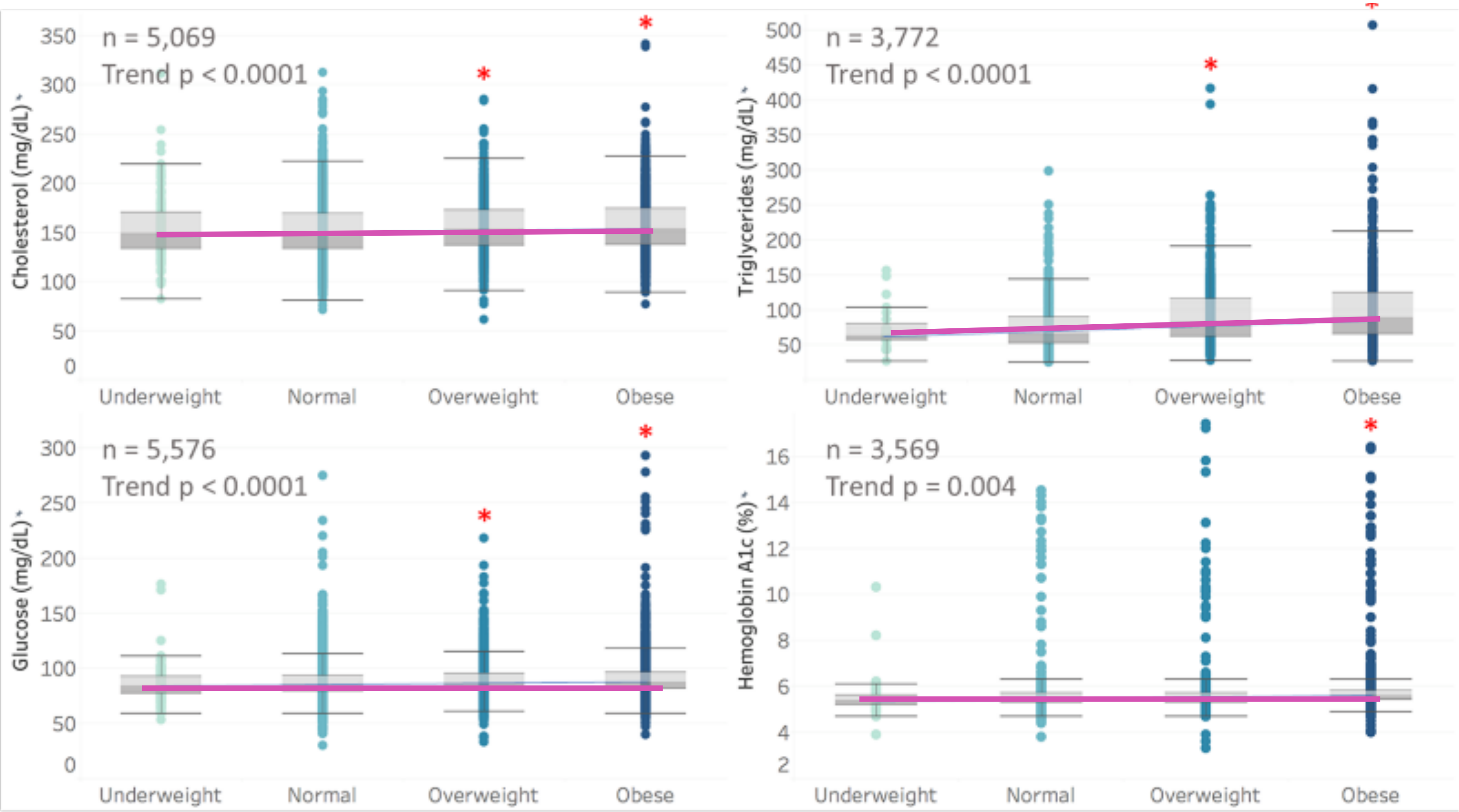

PREGNANCY & BIRTH OUTCOMES ANALYSES

Intergenerational Cycle of Growth Failure

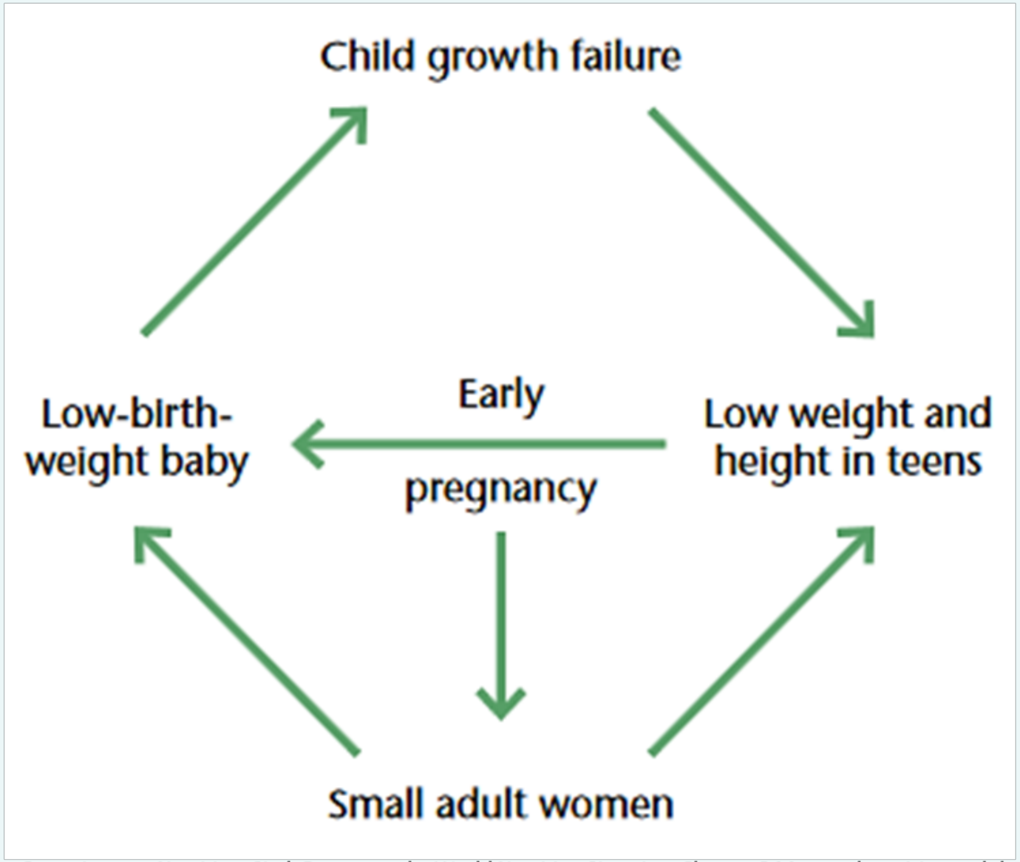

Source: United Nations System – Standing Committee on Nutrition, Sixth Report on the World Nutrition Situation: Chapter 3 Maternal nutrition and the intergenerational cycle of growth failure" (2013).

McDonald et al 2010	RR = 0.84	(0.75, 0.95)

Maternal Obesity - Feeding Behaviors and Child Obesity Risk

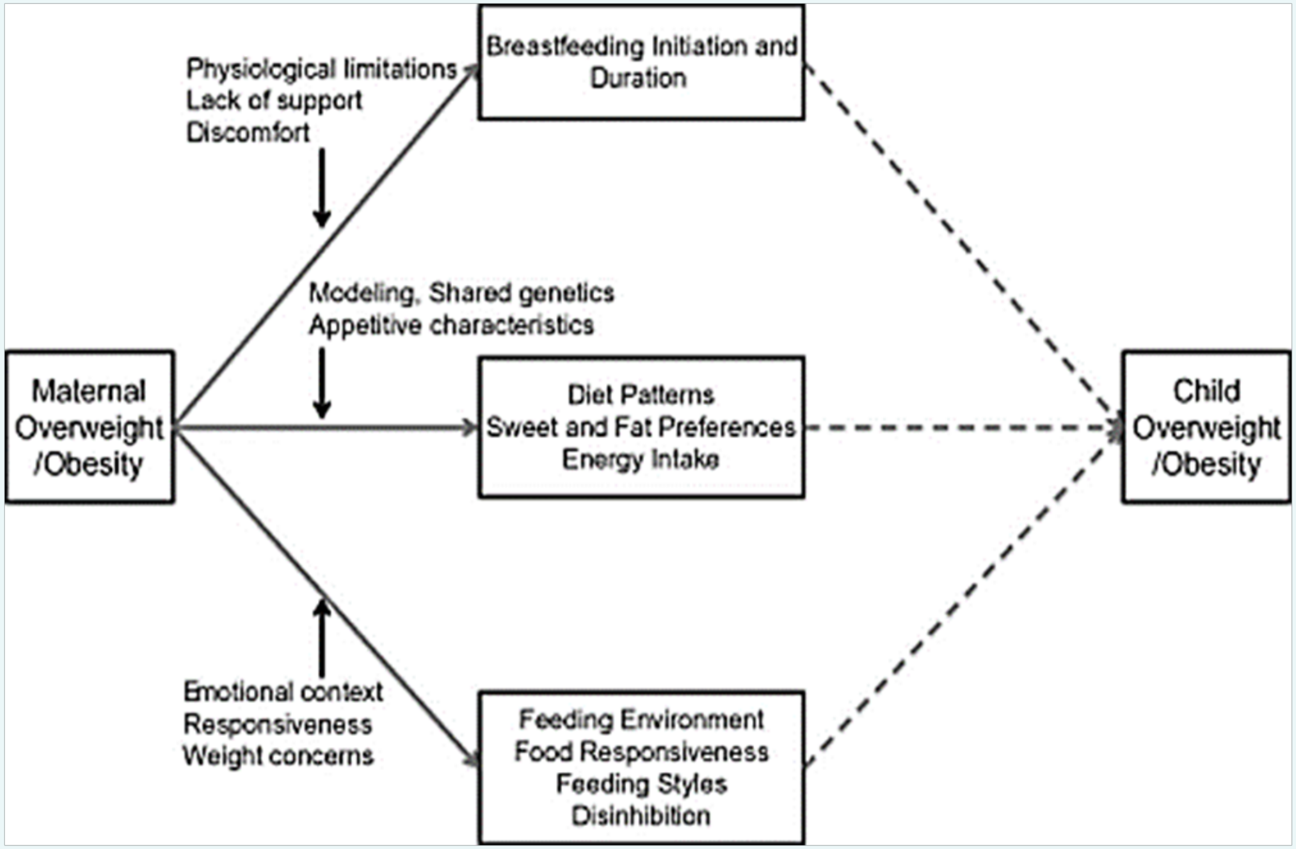

Source: Thompson AL. Intergenerational impact of maternal obesity and postnatal feeding practices on pediatric obesity. Nutrition reviews. 2013 Oct 1;71(suppl 1):S55-61.

Baby Birth Weight by Maternal BMI Group for Pregnant Adolescents (Sites A, B, C, D: n=2,866)

Birth Weight Group	Underweight (n=94) (3%)	Normal (n=1,278) (45%)	Overweight (n=809) (29%)	Obese (n=685) (23%)	Total (n=2,866) (100%)	P-value
Extremely LBW	**0%**	**0.5%**	0.7%	0.9%	0.6%	0.001*
Very LBW	**1.06%**	**0.7%**	0.5%	0.3%	0.6%	
LBW	**8.5%**	**7.4%**	4.9%	3.8%	5.9%	
Normal	90.4%	90.3%	92.6%	91.7%	91.3%	
Large	0%	1.2%	**1.2%**	**3.4%**	1.7%	

*P-value from logistic regression after combining [ELBW, VLBW, LBW] and [Normal, Large] with BMI group as a continuous variable for trend testing and site as a fixed effect.

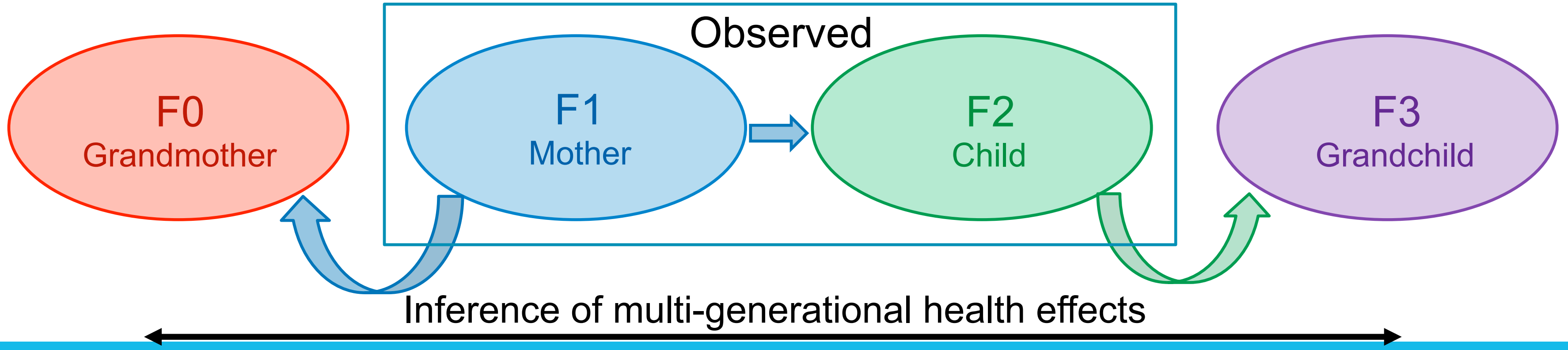

The Rockefeller University

THE SACKLER INSTITUTE *for* NUTRITION SCIENCE

a program of The New York Academy of Sciences

CDN CLINICAL-DIRECTORS-NETWORK www.CDNetwork.org

SACKLER DISSEMINATION

1. NIH-NIMHHD/Weill Cornell-Hunter CTSA Conference: "Stress & Resilience: The Science of Adapting to a Challenging World Symposium – 30th Annual Symposium of the Center for Translational and Basic Research Conference" (NYC May 15, 2017) - Received Best Poster Award: 1st Place (out of 81 posters)

2. NIH-NCATS article entitled "NCATS Enables Scientists, Community Clinicians to Collaborate on Health Initiatives" (Posted August 2017 at https://ncats.nih.gov/pubs/features/rockefeller)

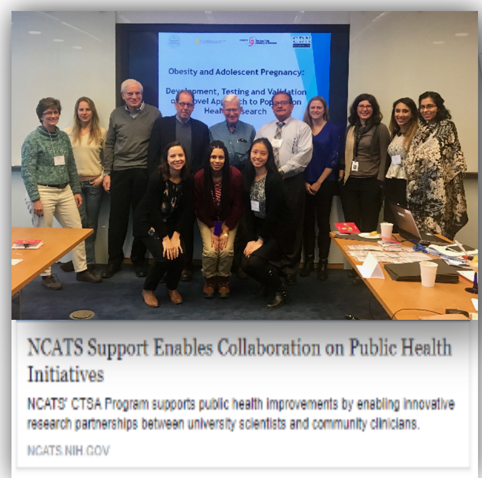

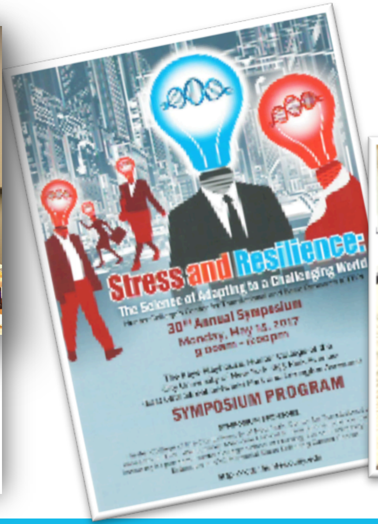

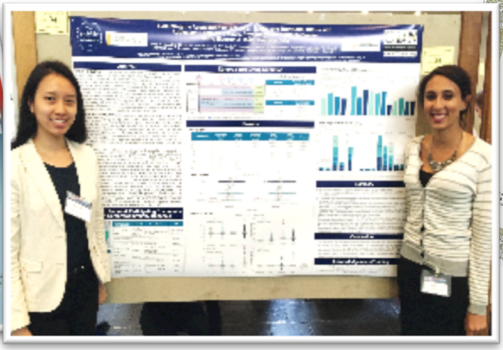

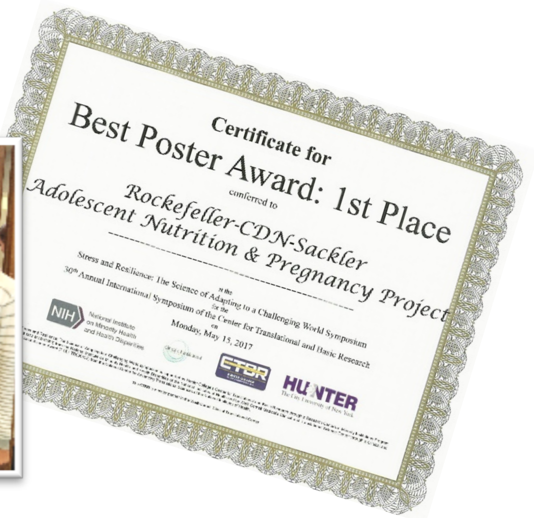

HOW DID WE DO THIS?

Team Science Principles
of Community-Academic
Research-to-Practice Partnerships

COMPARISON OF CAMP2 & SACKLER STUDIES

Project Components	CAMP2	SACKLER
**Study Design**	**RCT**	**Observational Cohort**
**Start Year**	**2010**	**2014**
**Monthly team meetings *(alternating teleconference & in-person)***	+	+
**Collaborative development and testing of hypothesis**		
• Patient defined measures and study parameters	+	-
• Clinician defined study parameters	+	+
**Stakeholders**		
• Clinicians	+	+
• Academic researchers	+	+
• Patients and community members	+	-
**Data type**		
• Many patients, few data points available *(Big Data)*	-	+
• Few patients, many clinical data points available *(Little Data)*	+	-

Team Meeting Agenda Template

TOPIC	PRESENTER
Welcome & Introductions	Principal Investigator
Progress Update	Project Manager
Site Reports	Clinicians and Staff
Hand-On Exercise Breakout Session	All
Case Report	Clinician Investigator
Literature Update	Academic Investigator
Dissemination	All
Next Steps	Principal Investigator
Wrap Up & Adjourn	Principal Investigator

Sample Breakout: Collaborative Grant Writing Activity

Group 1:	Group 2:	Group 3:	Group 4:
Creating Meaningful Patient Engagement in Planning the Study Design and Protocol	Patient Recruitment and Baseline Assessments	Incorporating Patient Needs in the Methodology	Defining Study Outcomes

CDN
CLINICAL•DIRECTORS•NETWORK
www.CDNetwork.org

tors Network | Rockefeller Webcasts Se

cdnetwork.org/rockefeller

CDN
CLINICAL•DIRECTORS•NETWORK
www.CDNetwork.org

Search

ABOUT US | RESEARCH | EDUCATION | DISSEMINATION | LIBRARY | PARTNERSHIPS | CONTACT US

THE ROCKEFELLER UNIVERSITY
Science for the benefit of humanity

The Rockefeller University

Center for Clinical and Translational Science (CCTS)

ctors Network | Rockefeller Webcasts Se

cdnetwork.org/rockefeller

Age Differences and Consequences for Mental Health" & "Maternal Experiences with Everyday Discrimination and Infant Birth Weight: A Test of Mediators and Moderators Among Young, Urban Women of Color"

Presenters:

Lisa Rosenthal, PhD, Assistant Professor, Psychology Department, Pace University

Valerie Earnshaw, PhD, Instructor in Pediatrics, Harvard Medical School, Associate Scientific Researcher, Division of General Pediatrics, Boston Children's Hospital

Sponsored By: The Sackler Institute for Nutrition Science, a program of the New York Academy of Sciences, The Rockefeller University Sackler Center for Biomedicine and Nutrition, Clinical Directors Network, Inc. (CDN), and the N2 PBRN Virtual Training Series (AHRQ, Grant No.1P30-HS-021667)

Click Here to View

► ARCHIVED WEBCAST

2016 Beatrice Renfield Lecture: Advancing Science, Improving Lives

Presented by: **Patricia A. Grady, PhD, RN, FAAN,** Director, National Institute of Nursing Research

Sponsored By: The Beatrice Renfield Foundation and The Rockefeller University Center for Clinical and Translational Science.

Click Here to View

► ARCHIVED WEBCAST

Beyond the Cross-sectional: Neighborhood Poverty Histories and Preterm Birth

Presented by: **Catherine Cubbin, PhD,** Associate Professor, School of Social Work, Faculty Research Associate, Population Research Center University of Texas at Austin

Sponsored By: The Sackler Institute for Nutrition Science, a program of The New York Academy of Sciences, The Rockefeller University, Sackler Center for Biomedicine and Nutrition Research, and N2 PBRN Virtual Training Series (AHRQ,Grant No.1P30-HS-021667)

Sponsors:

The Beatrice Renfield Foundation

The Rockefeller University Center for Clinical and Translational Science.

Date/Time:

February 9th, 2016, 6:00-7:00 pm EST

Click Here to View

► ARCHIVED WEBCAST

Emerging Concepts in Human Miscarriage

Presented by: **Zev Williams, MD, PhD,** Assistant Professor, Department of Obstetrics & Gynecology and Women's Health, Department of Genetics, Albert Einstein College of Medicine of Yeshiva University

Sponsored By: The Sackler Institute for Nutrition Science, a program of The New York Academy of Sciences, The Rockefeller University, Sackler Center for Biomedicine and Nutrition Research, and N2 PBRN Virtual Training Series (AHRQ,Grant No.1P30-HS-021667)

Click Here to View

► ARCHIVED WEBCAST

Patients in Context: EHR Capture of Social and Behavioral Determinants of Health: Meet the Author

Presented By: **Nancy Adler, PhD,** Professor of Medical Psychology, Director, Center for Health and Community University of California, San Francisco School of Medicine

Click Here to View

SPECTRUM OF TRANSLATIONAL RESEARCH

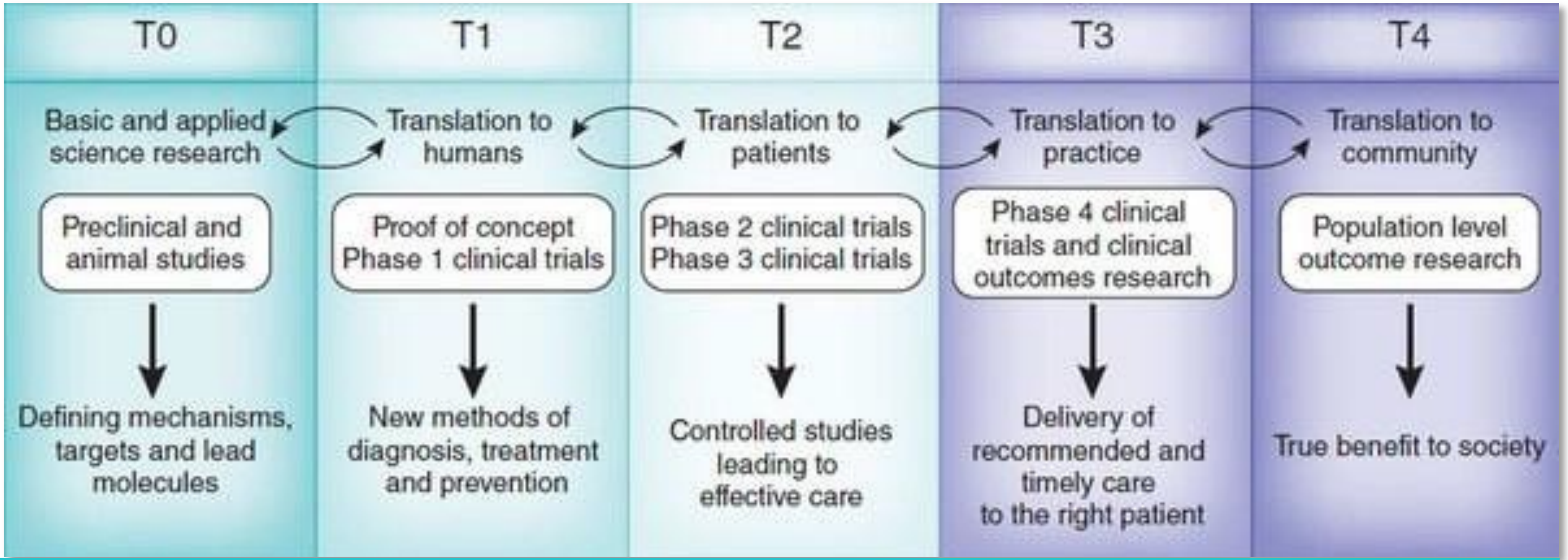

**CAMP2**	Metagenomics	Molecular Epidemiology/ Genotyping	Incision & Drainage Antibiogram	CDC Guidelines Dissemination and Implementation	Prevention of Recurrence and Transmission
**Sackler**	Micronutrient & Macronutrient	Allostatic Load Index	**Efficacy and Effectiveness Studies of Health Care:** Preconception Prenatal Postnatal Pediatric	**Implementation and Dissemination Studies of Health Care:** Preconception Prenatal Postnatal Pediatric	Validation with NCHS Surveys and Meta-Analysis

CRITICAL STEPS FOR PARTNERSHIP DEVELOPMENT FOR LITTLE DATA & BIG DATA STUDIES

- Listen to each stakeholders' needs, priorities, obligations and reward systems
- Elicit each stakeholders' specific priorities for measurement/assessment and intervention/outcome
- Align each set of measures and priorities
- Focus on pragmatic (> explanatory) interventions and designs
- Define outcomes in terms of:
    - Laboratory/Biological
    - Clinical
    - Patient-Centered
    - Health Services
    - Public Health
- Conduct Embed Mechanistic Studies with
    - Biological Measures (T0, T1) from clinical specimens
    - Well-characterized clinical phenotypes (T2, T3)

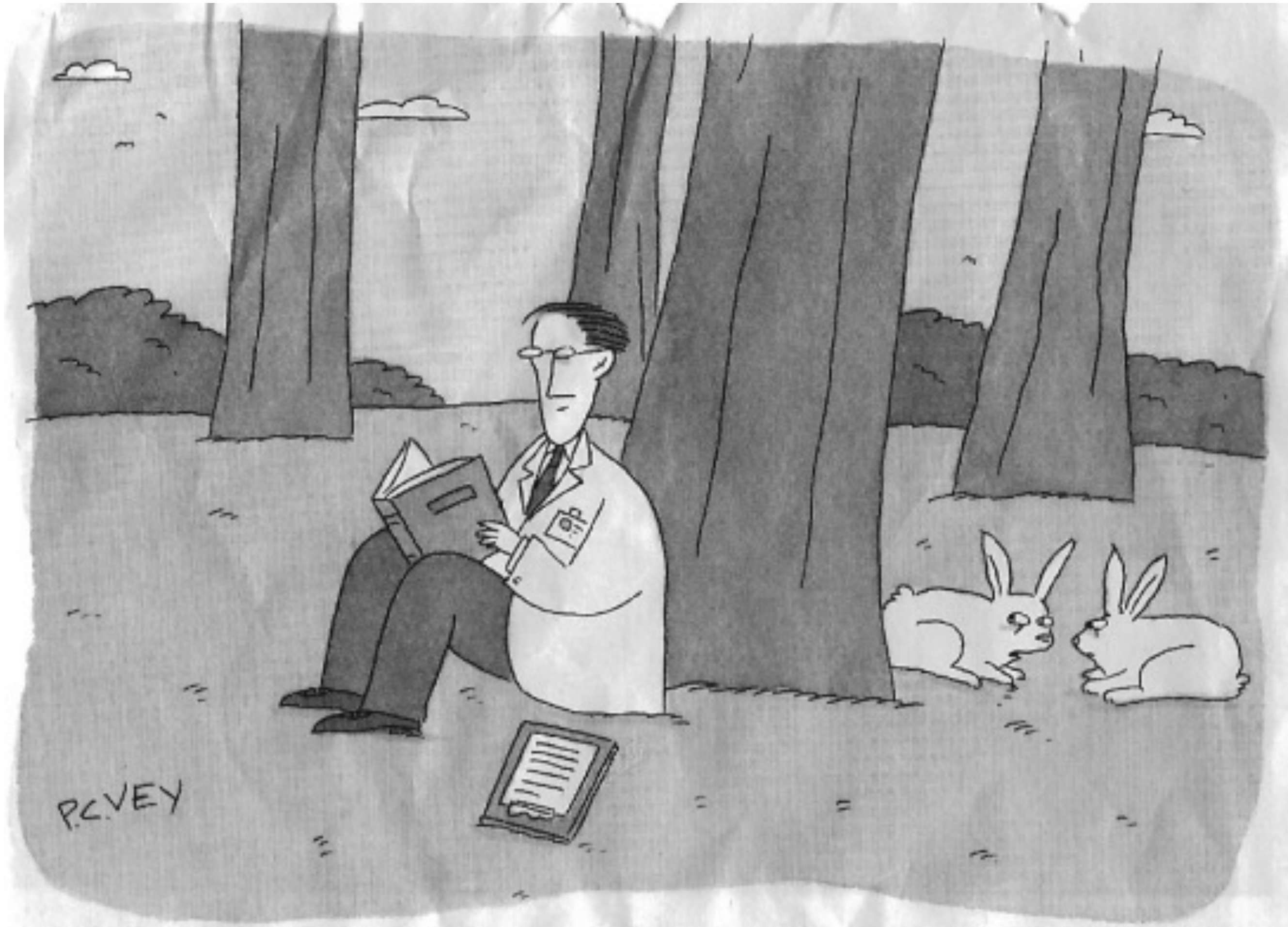

"They're harmless when they're alone, but get a bunch of them together with a research grant and watch out."

THANK YOU!

Jonathan N. Tobin, PhD
5 West 37th Street – 10th FL
New York NY 10018 USA
Tel 212-382-0699 x234
JNTobin@CDNetwork.org

THANK YOU!

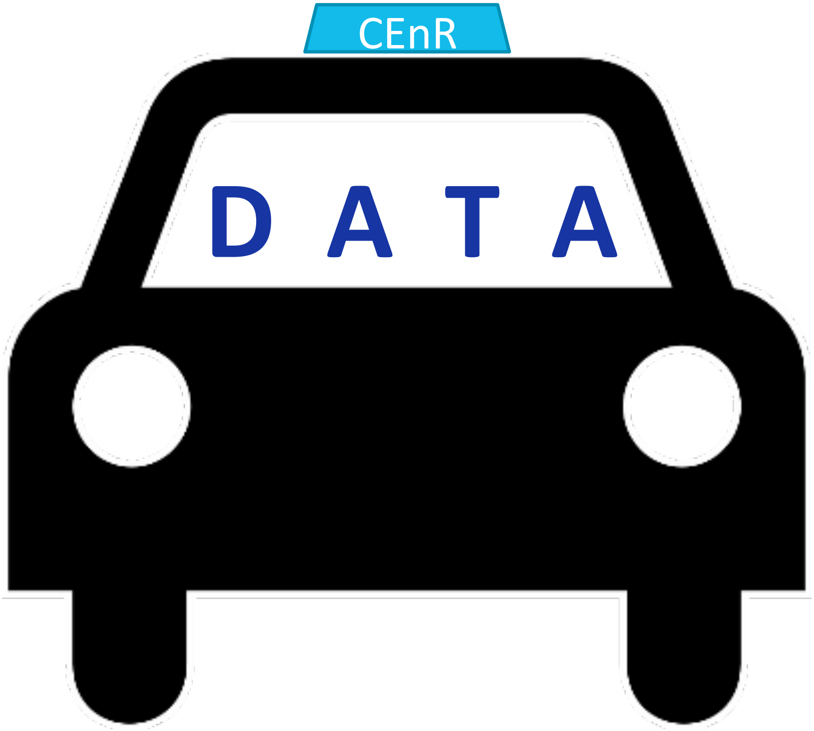

Pragmatic-Explanatory Continuum Indicator Summary (PRECIS) Tool

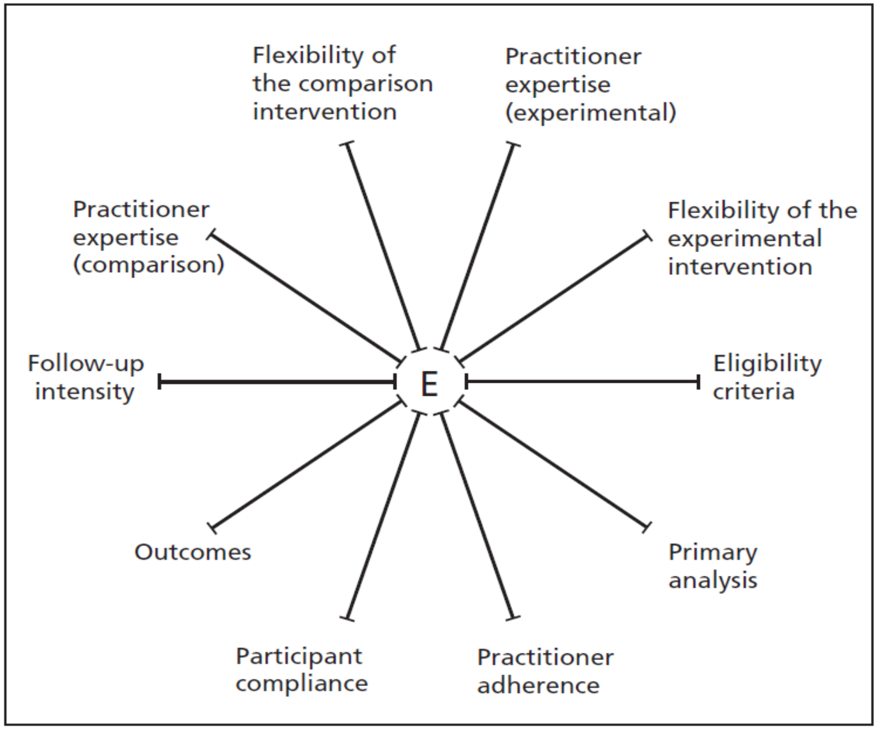

Figure 1: The blank "wheel" of the pragmatic–explanatory continuum indicator summary (PRECIS) tool. "E" represents the "explanatory" end of the pragmatic–explanatory continuum.

Thorpe KE et al. A pragmatic-explanatory continuum indicator summary tool (PRECIS): a tool to help trial designers. *CMAJ* 2009; 180(10):E47-57.

EFFECTIVENESS-IMPLEMENTATION HYBRID DESIGNS

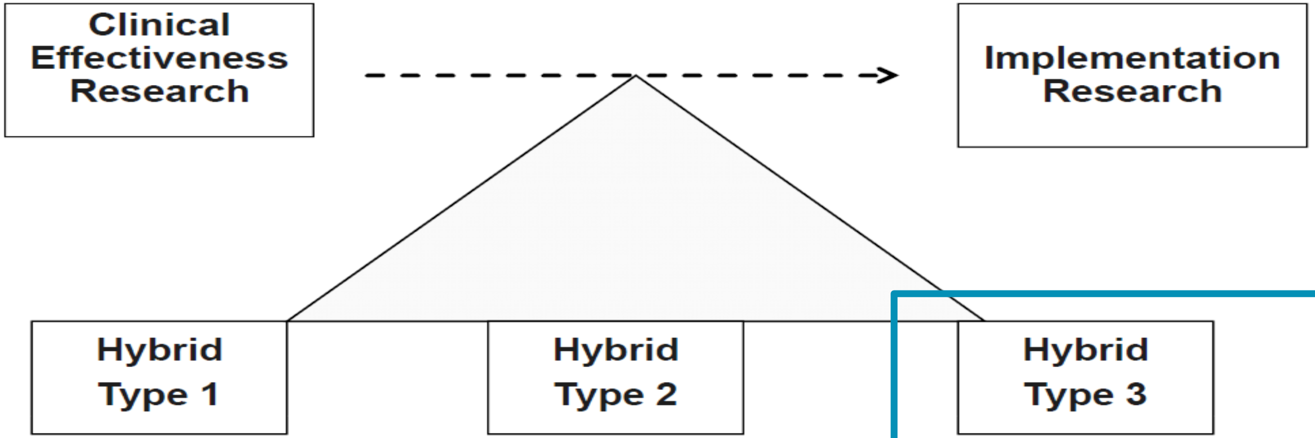

Hybrid Type 1: test clinical intervention, observe/gather information on implementation

Hybrid Type 2: test clinical intervention, test implementation intervention

Hybrid Type 3: test implementation intervention, observe/gather information on clinical intervention and outcomes

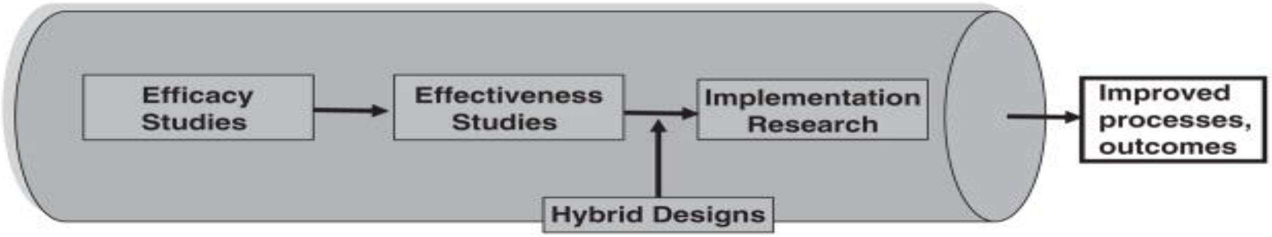

Curran, G. M., Bauer, M., Mittman, B., Pyne, J. M., & Stetler, C. (2012). Effectiveness-implementation Hybrid Designs: Combining Elements of Clinical Effectiveness and Implementation Research to Enhance Public Health Impact. *Medical Care*, *50*(3), 217–226.
